# Fluorinated Azobenzenes Switchable with Red Light

**DOI:** 10.1002/chem.202005486

**Published:** 2021-05-01

**Authors:** Anna‐Lena Leistner, Susanne Kirchner, Johannes Karcher, Tobias Bantle, Mariam L. Schulte, Peter Gödtel, Christian Fengler, Zbigniew L. Pianowski

**Affiliations:** ^1^ Institut für Organische Chemie Karlsruher Institut für Technologie Fritz-Haber-Weg 6 76131 Karlsruhe Germany; ^2^ Institut für Technische Chemie und Polymerchemie Karlsruher Institut für Technologie (KIT) Engesserstraße 18 76128 Karlsruhe Germany; ^3^ Institute of Biological and Chemical Systems – FMS Karlsruhe Institute of Technology Hermann-von-Helmholtz Platz 1 76344 Eggenstein-Leopoldshafen Germany

**Keywords:** azobenzene, photoswitches, red-light photoisomerization, therapeutic window

## Abstract

Molecular photoswitches triggered with red or NIR light are optimal for photomodulation of complex biological systems, including efficient penetration of the human body for therapeutic purposes (“therapeutic window”). Yet, they are rarely reported, and even more rarely functional under aqueous conditions. In this work, fluorinated azobenzenes are shown to exhibit efficient *E→Z* photoisomerization with red light (PSS_660nm_ >75 % *Z*) upon conjugation with unsaturated substituents. Initially demonstrated for aldehyde groups, this effect was also observed in a more complex structure by incorporating the chromophore into a cyclic dipeptide with propensity for self‐assembly. Under physiological conditions, the latter molecule formed a supramolecular material that reversibly changed its viscosity upon irradiation with red light. Our observation can lead to design of new photopharmacology agents or phototriggered materials for in vivo use.

## Graphical abstract

**Molecular photoswitches** find rapidly growing applications in biocompatible materials and photopharmacology. *ortho*‐Fluorinated azobenzenes bearing conjugated sp^2^ substituents can be effectively photoisomerized with light wavelengths above 630 nm, within the “therapeutic window”, which enables deep penetration of soft human tissues. This observation may therefore facilitate the design of new‐generation stimuli‐responsive therapeutics.

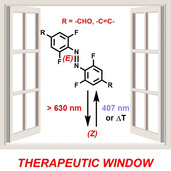

Molecular photoswitches undergo reversible light‐induced transformations between two forms that differ in their absorption spectra (photochromism) and molecular properties, such as geometry, polarity, or rigidity.^[1]^ They found a vast number of applications, ranging from material sciences^[2]^ to biological systems.^[3]^ A few selected examples are: photocontrol over molecular movement,^[4]^ assembly of nanoparticles^[5]^ and soft materials,^[6]^ or activity of biopolymers^[7]^ and pharmacologically relevant substances.^[8]^

The structures of established photoswitches, like azobenzenes,^[9]^ spiropyrans,^[1^°^]^ or diarylethenes, as well as emerging scaffolds, like indigoids,^[11]^ or Stenhouse adducts,^[12]^ are constantly modified in order to tune their photophysical properties to particular applications. While the majority of reported photochromes is triggered with UV light, photocontrol of biological systems is more optimal with visible wavelengths.[^9^, ^13^] Photoswitches triggered with light within so‐called “therapeutic window” (600‐900 nm) are particularly attractive for applications in photopharmacology, as these wavelengths enable deep penetration of soft tissues in human organism.^[14]^ This property has been reported for alkoxy‐, chloro‐[^14c^, ^15^] and – recently – mixed dichlorodifluoro^[15a]^ azobenzenes, as well as for diazocines,^[16]^ arylhydrazones,^[17]^ Stenhouse adducts (DASA),^[18]^ and hemithioindigos.^[19]^ However, due to synthetic difficulties, or incompatibility with physiological conditions their use remains limited,^[14a,2^°^]^ and novel photochromic scaffolds operational in this range of light are of high demand.

Azobenzene derivatives modified with fluorine atoms adjacent to the azo bond (“*ortho‐*fluoroazobenzenes”) belong to the most thermostable photochromic systems triggered with visible light (*E→Z* 530 nm, *Z→E* 410 nm). They combine efficient synthesis with pronounced geometry changes upon isomerization,^[21]^ and biological stability.^[6b]^ This chromophore found numerous applications in photocontrol of materials^[22]^ and biological systems^[23]^ with visible light. However, efficient (>50 % *Z‐*isomer) switching with light above 600 nm was not yet described for purely fluorinated azobenzenes, with exception of a two‐photon excitation process with high‐power NIR‐laser beam performed on a sensitizer‐coupled derivative.^[24]^

Here we report that functionalization of the tetra‐*ortho*‐fluoroazobenzene (TFAB) chromophore with sp^2^‐hybridized conjugated substituents leads to efficient *E*→*Z* isomerization upon irradiation with red light (>630 nm), and that the red light‐induced photoisomerization can be used to elicit a macroscopic effect – namely a reversible viscosity change ‐ in a supramolecular material under physiological conditions.

Our group has previously investigated drug‐releasing supramolecular hydrogels consisted of cyclic dipeptide‐based gelators bearing non‐fluorinated (**1 a**)^[2g]^ and fluorinated (**1 b‐c**)[^6b^, ^25^] azobenzenes. The cargo release was triggered, respectively, with UV, or with green light. In search for new phototriggered supramolecular materials, we wanted to exchange one of the stereocenters in **1 c** for a double bond. For that, we planned to couple a protected cyclo(Lys‐Gly) with the aldehyde **4**, previously synthesized by oxidation of the TFAB‐bearing alcohol **2** (Figure 1). As expected, the alcohol **2** photoisomerized (*E→Z*) upon irradiation with green light (λ_max_=530 nm) (Table 1).

**Figure 1:**
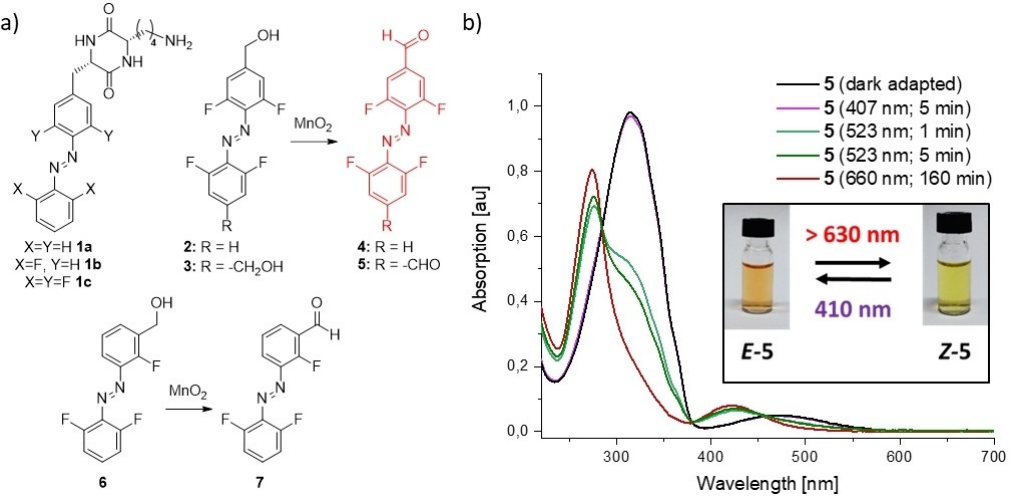
a) Photochromic compounds **1 a‐c** are efficient low‐MW supramolecular hydrogelators (as *E‐*isomers). Upon optimization of their structure we discovered that compounds **4** and **5** are capable of photoisomerization with red light (>630 nm), which expands the scope of their potential *in vivo* applications; b) UV‐Vis spectra of the bis‐aldehyde **5** (38 μM in MeCN). The *E→Z* photoisomerization is more efficient with red (λ_max_=660 nm, cut‐off filter <630 nm PSS_660_=82 % *Z‐* **5**) than with green light (λ_max_=532 nm, PSS_523_=67 % *Z‐* **5**). (see also Table 1); *inset*: optical demonstration of the photochromism of **5**; 407 nm–9 mW/cm^2^, 523 nm–7 mW/cm^2^, 660 nm (with filter)–56 mW/cm^2^.

**Table 1:** The percentage of *Z* photoisomers in photostationary states (PSS) obtained upon irradiation with violet, green, or red light of the compounds **2–7** determined by ^1^H NMR spectroscopy.

Compound	% of *Z‐* **x** in PSS λ_max_ 407 nm	% of *Z‐* **x** in PSS λ_max_ 523 nm	% of *Z‐* **x** in PSS λ_max_ 660 nm
TFAB	15 %	94 %	20 %
**2**	15 %	92 %	19 %
**3**	15 %	91 %	24 %
**4**	10 %	83 %	**75 %**
**5**	15 %	67 %	**82 %**
**6**	18 %	85 %	29 %
**7**	16 %	87 %	26 %
**8**	29 %^[a]^	55 %	**61 %**

[a] measured in PSS upon blue light irradiation (λ_max_ 470 nm), as decomposition was observed upon prolonged exposure of **8** on violet light (λ_max_ 407 nm).

But the respective aldehyde **4** exhibited unusual behavior. We observed that *E*‐**4** undergoes substantial *E→Z* photoisomerization (PSS_660 nm_=75 % *Z‐* **4**) upon irradiation with red light (λ_max_=660 nm) (Table 1). To assure that the emission shoulder of our light source does not overlap with the green light range, we used a 630 nm‐cutoff filter SCHOTT RG‐630.

Based on analogous reports for other classes of photoswitches, we hypothesized that the bathochromic shift in the absorption range can be attributed to the extended conjugated π‐electron system of the chromophore. To check this hypothesis, we have prepared two further aldehyde derivatives – the *bis*‐aldehyde **5** from the alcohol **3**, and the non‐conjugated aldehyde **7** from the alcohol **6**. The conjugated *bis*‐aldehyde **5** showed even more pronounced photoisomerization (PSS_660 nm_=82 % *Z‐* **5**) under red light irradiation (λ_max_=660 nm, filter cut‐off <630 nm) (Figure 1), while the non‐conjugated aldehyde **7**, as well the alcohols **2**, **3**, or **6**, did not switch significantly under these conditions, as compared with the photostationary states (PSS) achieved with the reverse isomerization elicit by violet light (PSS_407 nm_) (Table 1).

At higher concentrations (*e. g*. 1.5 mM in DMSO), the absorption peak tail above 600 nm, responsible for the photoisomerization of *E‐* **4** and *E‐* **5** with red light, is clearly visible (Figure 2). The S_0_‐S_1_ absorption bands of both photoisomers, which in azobenzene systems are commonly identified as n‐π* transitions, exhibit in our case sufficient separation (from 25 nm for **6** to 51 nm for **5**) to selectively address each photoisomer with visible light. This is important, as the distribution of photoisomers in PSS at a given wavelength is governed by the ratio of the products of molar attenuation coefficients and quantum yields for each isomer. For example, at 630 nm the ϵ_630 nm_ of *E‐* **5** is 11.5 M^−1^ cm^−1^, while the ϵ_630 nm_ of *E‐* **5** is 1.47 M^−1^ cm^−1^. The ratio of both values satisfyingly corroborates with the observed *Z/E‐*photoisomer ratio in PSS (82 % of *Z‐* **5**) obtained upon irradiation with the red LED, and indicates that the difference in quantum yields for both photoisomers is not critical in the demonstrated case.

**Figure 2:**
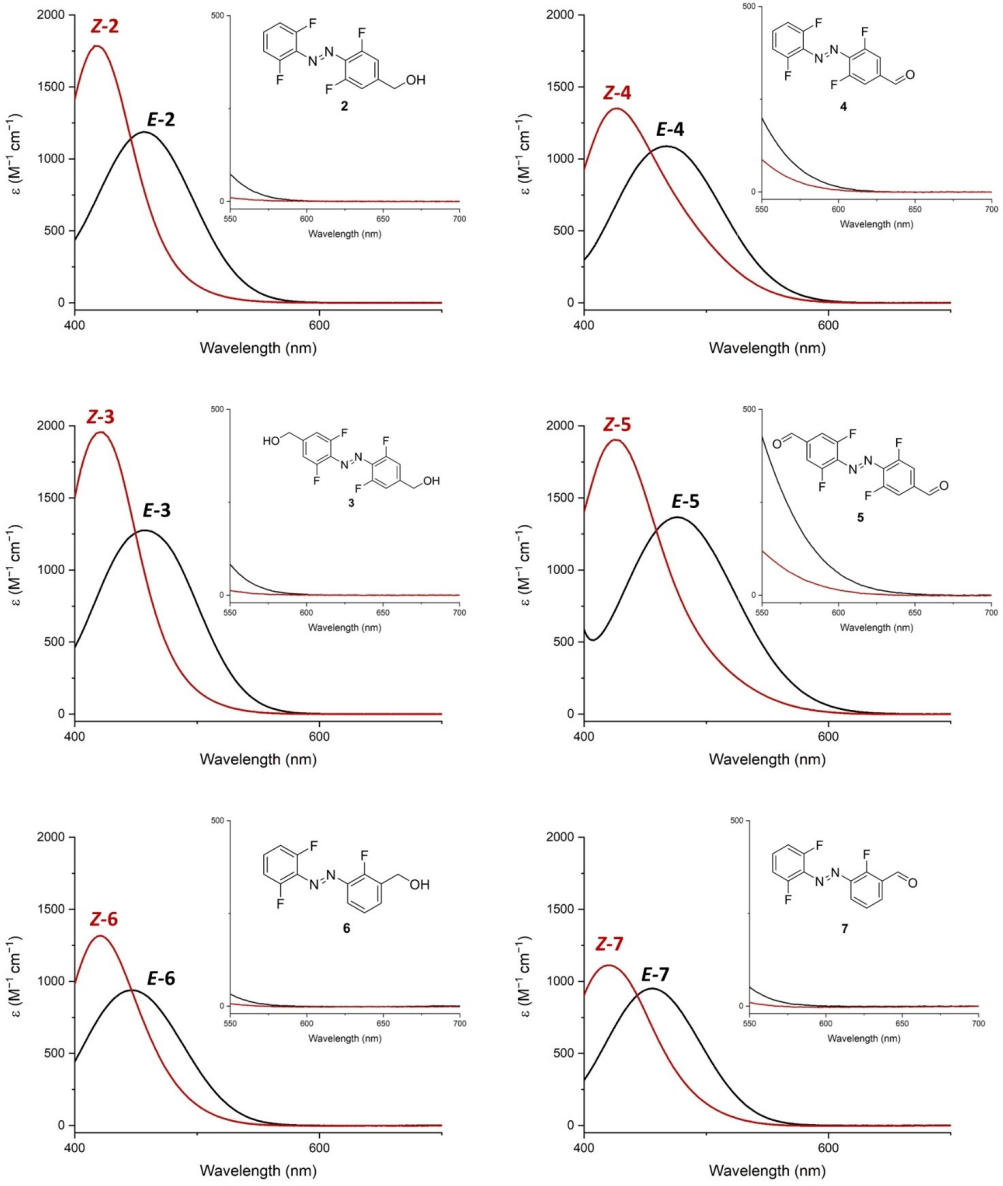
Band separation of the *E*‐isomers (black lines) and *Z‐*isomers (red lines) of the compounds **2–7** in the visible light range (1.5 mM solutions in d_6_‐DMSO). Insets show the magnified respective band separation in the range 550–700 nm. In all cases, the y‐axis depicts the molar attenuation coefficient ϵ (M^−1^ cm^−1^). The spectra of pure *Z*‐isomers (red lines) have been calculated from spectra registered for samples irradiated 15 min with 523 nm LED with concomitant determination of the *E/Z*‐ratio by ^1^H NMR (procedure described in Supporting information, pages S77‐S92, Figures S12–S40).

Next, we have investigated thermal stability of the aldehydes *Z*‐**4** and *Z*‐**5** in MeCN at 60 °C. The half‐life of *Z‐* **4** under these conditions – 10.8 h – was comparable to other TFAB derivatives,^[21b]^ while the half‐life of *Z*‐**5** – 3.2 h – was similar to the value of the unsubstituted azobenzene (Figure S5).^[21b]^

To gain more understanding on the observed photophysical properties, we have corroborated our experimental results with theoretical calculations on all *E*‐ and *Z*‐isomers, respectively. The results of ground state optimization show that aldehyde substituents in *para* position lead to an extended π‐conjugation, which causes a shift of both the HOMO (n) and LUMO (π*) levels to lower energies. The decrease in the energy level of the LUMO (π*) is more pronounced than for the HOMO (n), which results in a significantly lower HOMO‐LUMO gap for the compounds **4** and **5** compared to the non‐conjugated aldehyde **7**, unsubstituted TFAB, or *sp* ^3^‐substituted derivatives **2**, **3** or **6** (Figure 3, Table S3). Based on the optimized structures, time‐dependent calculations were performed, applying the polarizable continuum model (PCM) for the solvent MeCN. Bathochromic shifts of the calculated excitation maxima for *E‐* **4** and *E*‐**5** versus the other compounds (Table S19) are in qualitative agreement with the experimentally observed shifts in the measured UV‐Vis spectra (Figure S9).

**Figure 3:**
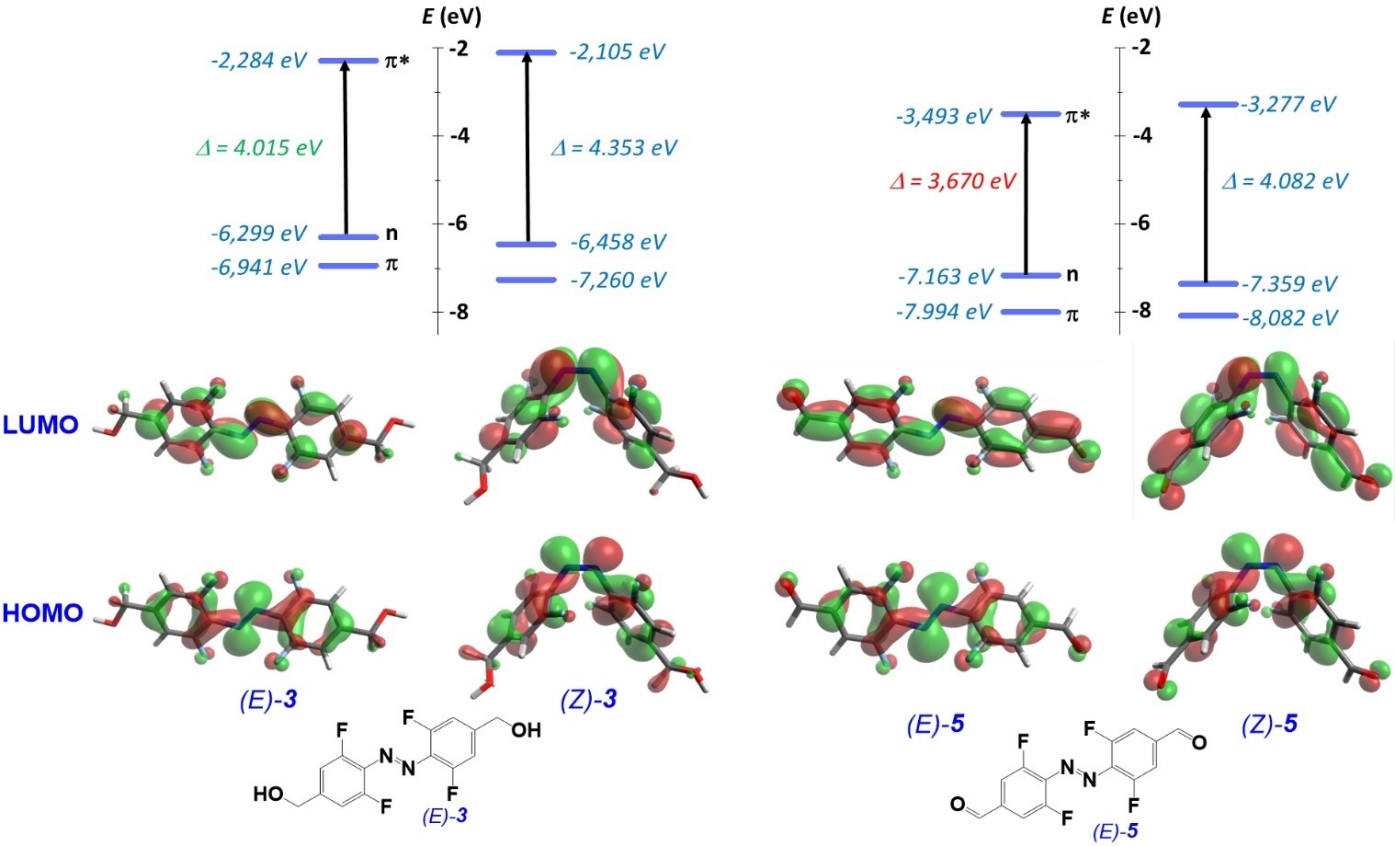
Molecular modelling of the HOMO‐LUMO gap of the bis‐alcohol **3** and the corresponding bis‐aldehyde **5** (PBE0‐D3/def2‐TZVP level of theory). The HOMO‐LUMO gap of 3.670 eV for the *E*‐**5** vs. 4.015 eV for the *E‐* **3** (see Table S3) corroborates with the experimentally observed bathochromic shift of the absorption maximum in the *E*‐**5**. EWG aldehyde substituents in **5** stabilize all orbitals (π, n, and π*) in comparison to **3**. However, the stabilization is most pronounced for the π* (LUMO) orbital, most likely due to the extended conjugation in the π‐orbital system depicted in the orbital contour.

Then we wondered, if our observation can be implemented into a material, which properties are reversibly triggered with red light. Incorporation of aldehydes, like **4** or **5**, into smart materials or systems of biological interest would be complicated. Thus we hypothesized that similar bathochromic shift may occur for other conjugated *sp^2^‐*substituents, more suitable as linkers, – in particular C=C bonds. To verify that, we have coupled the *bis*‐aldehyde **5** upon base‐catalyzed condensation with two equivalents of a Boc‐protected cyclic dipeptide cyclo(Gly‐Lys), followed by complete removal of the residual Boc groups.

The resulting symmetric unsaturated TFAB derivative **8** also photoisomerized upon exposure on red light (λ_max_=660 nm, >630 nm) (PSS_660 nm_=61 % (*Z*)‐**8**) (Table 1), with the *Z*‐**8** half‐life of 17 min at 60 °C (Figure S6, left). This is considerably shorter than the aldehydes **4** or **5**. Nonetheless, the half‐life of 14.8 h for the same *Z‐* **8** measured at 20 °C (Figure S6, right) is still comparable with other red light‐switchable azobenzenes. The compound **8** remained stable during 10 cycles of forth‐and‐back switching with alternate 660 nm and 470 nm irradiation (Figure S2–S4). In presence of 10 mM reduced glutathione (a standard mimic of intracellular reduction potential) under physiological conditions at 25 °C roughly half of the compound **8** was degraded within 10 hours (Figure S41). Therefore, it has to be kept in mind, that eventual intracellular applications of this chromophore have to be time‐limited.

Next, we wanted to investigate self‐assembly properties of **8** in aqueous solutions, and potential macroscopic effects which can be triggered upon its exposure on red light. Compound **8** suspended in water, 200 mM aq. NaCl, or Ringer solution under physiological conditions, and shortly boiled, yielded gel‐like opaque viscous material at the concentration range between 10 g/L and 20 g/L (1–2 wt%) of *E‐* **8** (Table S20–S22). The presence of salts significantly enhanced its melting temperature.

However, its low mechanical stability ‐ quantified with rheological experiments (Figure S26) – was different from the stability of hydrogels formed by the gelators **1 a–c** under analogous conditions. Upon irradiation with red light (660 nm, with filter >630 nm, 56 mW/cm^2^), this material rapidly (5 min) dissipates to non‐viscous, opaque fluid (Figure 4). This fluid returns to the initial viscosity upon short boiling, which thermally restores the *E‐*isomer.

**Figure 4:**
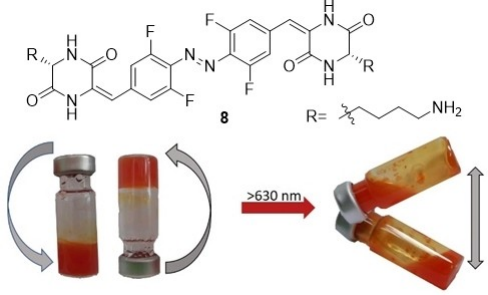
The molecule **8** consisted of two gel‐forming cyclic dipeptide motifs linked with the conjugated TFAB photochromic motif. The gel‐like material formed from *E‐* **8** in aqueous conditions can be reversibly dissipated to non‐viscous liquid with red light. This, in turn, solidifies upon thermally induced back‐isomerization.

The structure of this material has been investigated with electron microscopy techniques (Figure 5, Figure S42‐S56). In the non‐irradiated material, we observed mostly μm‐long thick structures (Figure 5A), contrary to previously investigated hydrogels formed from **1 a–c**, consisted of a dense network of small fibers. Upon irradiation with red light, along with the viscosity change, we observed almost total decay of the thick structures (Figure 5B). They were, however, restored upon thermally induced back‐isomerization (Figure 5C). The morphology of freeze‐dried SEM and air‐dried TEM samples was also characterized for various solvent compositions (Figure 5E–F).

**Figure 5:**
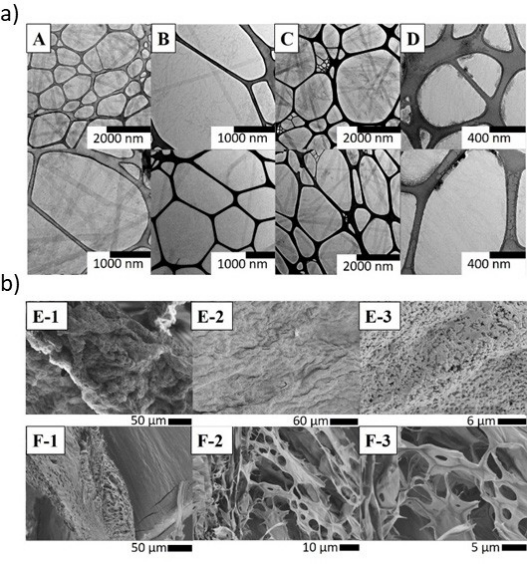
Electron microscopy of the supramolecular assemblies composed from **8** (1 wt%) under aqueous conditions (Ringer's solution+1 % lead citrate). a) (A) the structure visible as multiple μm long and nm thick fibers; (B) after irradiation at 660 nm (50 min) ‐ the quantity of the described fibers decreases and smaller structures in the nm‐scale are formed; (C) regeneration of fibers occurs after boiling the irradiated gel; (D) upon 1 : 10 dilution of (B) and irradiation at 660 nm – only small fibers visible. b) freeze‐dried samples of hydrogels prepared from 1 wt% **8** in Ringer's solution (E) (no network visible), or from 2 wt% **8** in in water (F) (sponge‐like network revealed); Scalebars: (A) top – 2 μm, bottom 1 μm, (B) – 1 μm, (C) – 2 μm, (D) – 400 nm, (E),(F) – see also Figures S42‐S56.

In addition, ^1^H‐NMR transverse relaxation (*T*_2_) measurements were used to investigate the microstructure of the gel as it is directly linked to the molecular mobility (1–10 nm) of the components. The mobility can be quantitatively assessed by the decay of the transverse magnetization using time‐domain NMR techniques. Herein, a magic sandwich echo (MSE) pulse sequence was used to refocus the initial transverse magnetization (100 ms) of rigid components in combination with a CPMG (Carr‐Purcell‐Meiboom‐Gill) pulse sequence to refocus the magnetization of more mobile components up to 1 s. As shown in Figure S58a distinct decay of the FID by 80 % in the first 100 ms is observed, which indicates a highly polycrystalline nature of the microstructure and corroborates with the structures observed with electron microscope (Figure 5a).

Comparison between **8** and the hydrogelators **1 a–c** indicates the crucial role of a flexible linker between the azobenzene and the peptide fragment for efficient self‐assembly of fibrous structures and the resulting hydrogel formation. A similar issue has been already discussed for hydrogelation of short linear photochromic peptides decorated with azobenzenes.^[26]^

In conclusion, we demonstrated the efficient *E→Z* photoisomerization of tetra‐*ortho‐*fluoroazobenzenes substituted with sp^2^‐hybridized conjugated substituents with red light within the “therapeutic window” (>630 nm). These results were successfully corroborated with calculations. Importantly, TFAB derivatives with saturated substituents, or a fluorinated azobenzene substituted with an sp^2^‐hybridized carbon at the *meta*‐position were essentially inert to red light within our experimental setup.

The new chromophore has been coupled with a cyclic dipeptide motif. The resulting compound, structurally analogous to previously described hydrogelators, formed a viscous material in aqueous media under physiological conditions. This material was reversibly dissipated to non‐viscous fluid with red light, and recovered its initial form upon thermal equilibration. The microscopic structure indicated its polycrystalline nature, which is reversibly dismantled upon photoisomerization. This example demonstrated that the red light‐switchable conjugated TFAB photochrome can be incorporated into larger molecules, using a C=C bond linker, remains stable under physiological conditions, and can elicit macroscopic effects upon irradiation with red light. Thus, this photochrome is suitable for applications in new biocompatible materials, or in photopharmacology agents, comprising these operational inside human organisms, with reservation to its slow decomposition upon exposure to glutathione.

## Conflict of interest

The authors declare no conflict of interest.

## Supporting information

As a service to our authors and readers, this journal provides supporting information supplied by the authors. Such materials are peer reviewed and may be re‐organized for online delivery, but are not copy‐edited or typeset. Technical support issues arising from supporting information (other than missing files) should be addressed to the authors.

Supplementary
