# Supplementary material for: Fluorinated Azobenzenes Switchable with Red Light
Source: Chemistry. 2021 May 1;27(31):8094–9. doi: 10.1002/chem.202005486 (PMC8252058; doi:10.1002/chem.202005486)
Supplement: Supplementary file 1 — Supplementary [file CHEM-27-8094-s001.pdf]

Chemistry–A European Journal

Supporting Information

Fluorinated Azobenzenes Switchable with Red Light

Anna-Lena Leistner, Susanne Kirchner, Johannes Karcher, Tobias Bantle, Mariam L. Schulte, Peter Gödtel, Christian Fengler, and Zbigniew L. Pianowski*

Author Contributions

A.-L.L. Data curation:Lead; Investigation:Lead; Writing – original draft:Supporting; Writing – review & editing:Equal
S.K. Data curation:Equal; Software:Lead
J.K. Data curation:Supporting; Investigation:Supporting
T.B. Data curation:Supporting
M.S. Data curation:Supporting
P.G. Data curation:Supporting
Z.P. Conceptualization:Lead; Funding acquisition:Lead; Project administration:Lead; Resources:Lead; Supervision:Lead; Writing – original draft:Lead; Writing – review & editing:Lead

Table of contents:

1. General Information: 2
2. Synthesis: 4
3. Photophysical properties of the compounds 2-8 18
4. Computations 22
5. UV/Vis isomerization experiments 75
6. Calculation of the attenuation coefficients 77
7. Biological stability 93
8. Viscosity experiments 94
9. Microscopy images 95
10. Rheology and NMR relaxometry of 8 103
11. NMR spectra of the synthesized compounds 106
12. Photoisomerization Experiments 137
13. Relative spectral power distribution of the used LED light sources 158
14. References 159

1. General Information:

All reagents and starting materials are commercially available (Sigma-Aldrich, Fluorochem, chemPur, Alfa Aesar or Bepharm) and were used as supplied unless otherwise indicated. All experiments were conducted in air and in deionized water (Millipore) unless otherwise noted. All experiments with molecules that can photoisomerize with visible light frequencies, namely the azobenzene derivatives containing fluorine atoms, were performed in absence of sunlight (brown glassware, or colorless glassware wrapped with aluminium foil, working in a room with dimmed light in a fume hood equipped with red light). All reactions containing air- and moisture-sensitive compounds were performed under argon using oven-dried glassware applying common Schlenk-techniques. Liquids were added via steel cannulas and solids were added directly as powders.
Column chromatography was performed on Silica gel 60 Å (40-63 µm particle size) (Sigma). **NMR spectra** were recorded using the following devices: ^{1}H NMR: Bruker 300 (300 MHz), Bruker Avance 400 (400 MHz), Bruker Ascend 500 (500 MHz), ^{13}C NMR: Bruker 300 (75 MHz), Avance 400 (101 MHz), Ascend 500 (126 MHz), ^{19}F NMR: Avance 400 (377 MHz) or Ascend 500 (471 MHz). The following solvents from Eurisotop were used: chloroform-d_1 ($CDCl_3$), acetic acid-d_4, DMSO-d_6, and D_2O. Chemical shifts δ were expressed in parts per million (ppm) and referenced to $CDCl_3$ (^{1}H: δ=7.26 ppm, ^{13}C: δ=77.16 ppm), acetic acid-d_4 (^{1}H: δ=2.04 ppm, ^{13}C: δ=178.99 ppm), DMSO-d_6 (^{1}H: δ=2.50 ppm, ^{13}C: δ=39.52 ppm) and D_2O (^{1}H: δ=4.79 ppm). [1] ^{19}F-NMR were not referenced. **Mass spectra** were recorded on a Finnigan MAT 95 mass spectrometer using electron ionization-mass spectrometry (EI-MS) or fast atom bombardment-mass spectroscopy (FAB-MS). For FAB measurements *m*-nitrobenzyl alcohol (3-NBA) was used as the matrix. The software of FAB and EI adds the mass of one electron. Electrospray ionization–mass spectrometry (ESI-MS) spectra were recorded on a Thermo Fisher Scientific Q Exactive mass spectrometer. Calibration was carried out using premixed calibration solutions (Thermo Fisher Scientific). The molecular fragments are stated as ratio of mass per charge *m/z*. **UV-Vis spectra** were recorded on a Lambda 750 (PerkinElmer) UV-Vis spectrophotometer at 20 °C, slit=2 nm. Quartz cuvettes of 10 mm optical path length were used. **IR spectra** were recorded on a Bruker IFS 88 using ATR (Attenuated total reflection). The intensities of the absolute peaks are given as follows: vs=very strong 0-9% T, s=strong 10-39% T, m=medium 40-69% T, w=weak 70-89% T, vw=very weak 90-100% T. All spectroscopy samples were taken at room temperature. **Analytical High Performance Liquid Chromatography (HLPC)** was performed using a Thermofisher UltiMate 3000 system containing a degaser, pump, autosampler, column compartment and diode array detector. The flow rate was 1 mL/min on a stationary *PerfectSil Target* (MZ-Analytik) C_{18} column (3-5 µm, 4.0 mm × 250 mm). Chromeleon 7 software was used for data extraction. **Preparative HPLC** separation was performed with a LC-2000Plus series from Jasco with a VDSpher column with C18-M-SE, 250 × 20 mm and 10 µm from VDSoptilab.

Analytical thin layer chromatography was carried out using silica coated aluminium plates (silica 60, F_{254}, layer thickness: 0.25 mm) with fluorescence indicator by Merck. Detection proceeded under UV light at λ=254 nm.

Sample irradiation for measurements of photostationary states was performed using LED diodes with following emission maxima: 3 W LED diode 365 nm, and 10 W LED diodes: 407, 523, 623, 640 and 660 nm from LED Engin. For the time of irradiation, samples were maintained at constant temperature (22 ± 2 °C) using a metal cooling block unless otherwise noted.
For the irradiation process (NMR experiments for quantification of the PSS) with the red-light-emitting diodes (λ_{max} of 623 nm, 640 nm and 660 nm) a cut-off filter (SCHOTT RG-630, 50mm DIA, from "EDMUND Optics, Inc.") was applied, which eliminates light frequencies below 630 nm. It assured that no residual emission shoulder, especially in the green light area (below 570 nm) could cause the reported effect, which we assigned exclusively to the absorption of red light frequencies by examined molecules.

Using the PowerMax USB (type PS19Q) sensor device (Coherent®) we have measured the irradiation intensity for the particular diodes used in our experiment (5 independent measurements, the detector (diameter 19 mm) was located at the distance of 55 mm from the light source, identical as the position of irradiated samples) (**Table S1**).

Table S 1: Irradiation intensity of LED light sources used in experiments described in this report.

λ_{max} of the LED diode	Light intensity (mW/cm^2) without filters	Light intensity (mW/cm^2) with the filter RG-630	Average power (W)	Variance (W)	SD (W)
365 nm	0.56	-	$1.59*10^{-3}$	$7.64*10^{-9}$	$8.74*10^{-5}$
407 nm	9.07	-	$2.57*10^{-2}$	$2.72*10^{-7}$	$5.52*10^{-4}$
523 nm	7.08	-	$2.01*10^{-2}$	$3.48*10^{-6}$	$1.87*10^{-3}$
623 nm	-	27.2	$7.71*10^{-2}$	$2.05*10^{-5}$	$4.53*10^{-3}$
640 nm	3.54	-	$1.00*10^{-2}$	$7.02*10^{-7}$	$8.38*10^{-4}$
640 nm	-	2.22	$6.28*10^{-3}$	$6.40*10^{-8}$	$2.53*10^{-4}$
660 nm	68.0	-	$1.93*10^{-1}$	$2.81*10^{-3}$	$5.30*10^{-2}$
660 nm	-	56.1	$1.59*10^{-1}$	$1.16*10^{-3}$	$3.41*10^{-2}$

2. Synthesis:

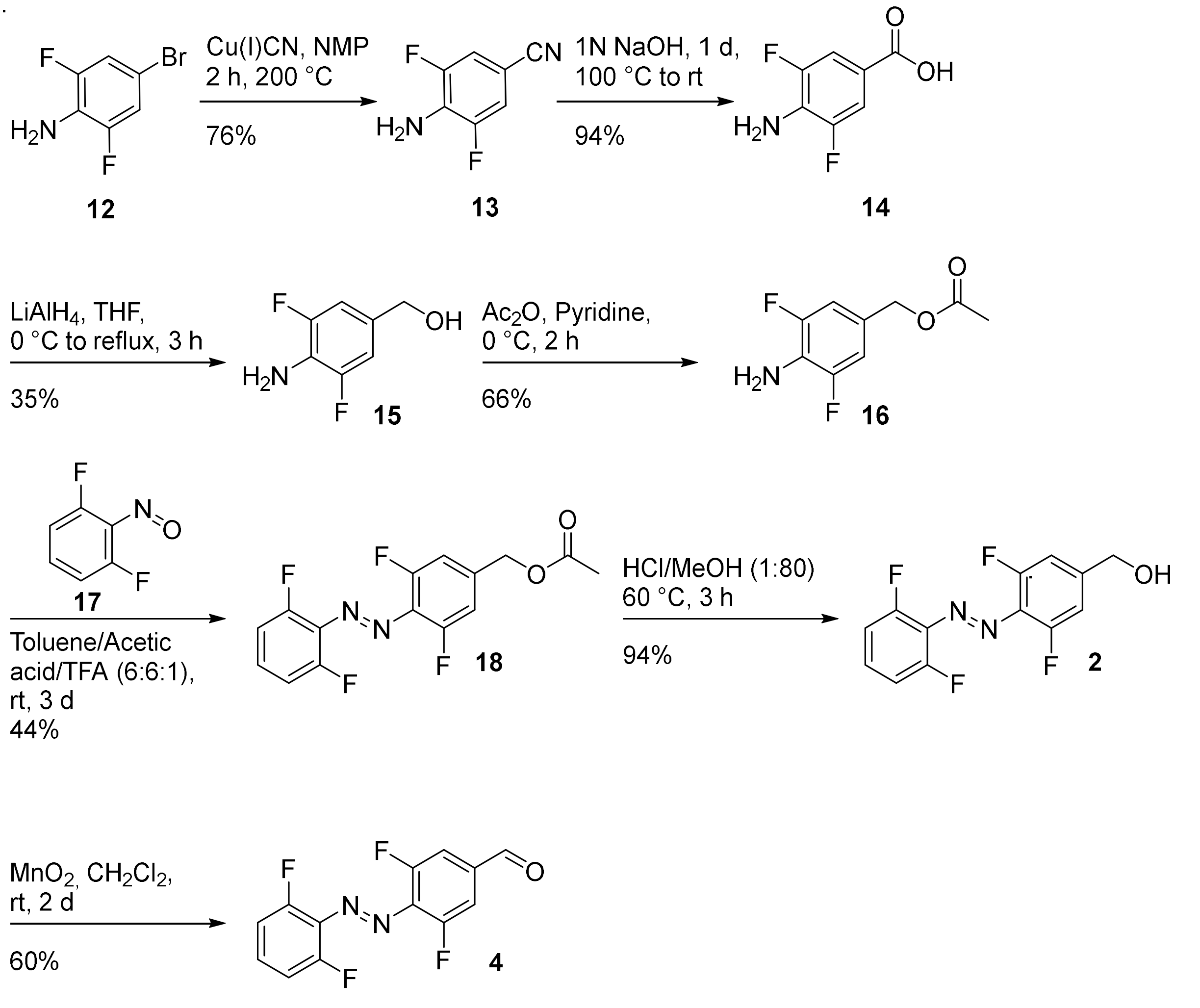

*Scheme S 1: Synthesis of (E)-4-((2,6-difluorophenyl)diazenyl)-3,5-difluorobenzaldehyde) (**4**) (**TFAB-4-aldehyde**). The acetylation (**15**→**16**) with following deprotection (**18**→**2**) was necessary for the Mills reaction. Otherwise, reaction of **15** and **17** directly aiming at **2** was accompanied with acetylation side reaction, low efficiency and multiple by-products lowering the final yield.*

4-Amino-3,5-difluorobenzonitrile (13)

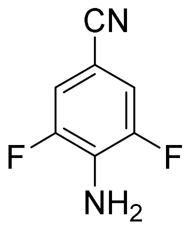

A mixture of 4-bromo-2,4-difluoroaniline **12** (48.7 g, 234 mmol, 1.00 eq.) and copper(I) cyanide (25.2 g, 281 mmol, 1.20 eq.)[5] in NMP (100 mL) was refluxed at 200 °C for 1.5 h (preheated silicone

oil bath). After cooling down to rt, the reaction mixture was poured into an aqueous solution of 12% ammonium hydroxide (1000 mL), CH_2Cl_2 (300 mL) and active coal (5.00 g). The blue mixture was filtrated over Celite® (h=4 cm, Ø=4 cm) and the Celite® pad was washed with CH_2Cl_2 (2×50 mL). The solution was extracted with CH_2Cl_2 (3×200 mL). After the separation of the layers, the organic phase was washed with water (2×200 mL) and sat. aqueous $NaHCO_3$ (100 mL) and dried over Na_2SO_4. The solvent was removed under reduced pressure. The crude product was purified by recrystallization: First active coal (5.0 g) and water (100 mL) was added and the mixture was refluxed for 10 min (oil bath, 120 °C). Then more water was added (ca. 100 mL) until the product was dissolved. The hot solution was filtered off to remove undesirable side products and the active coal. The clear solution was cooled overnight in the fridge (4 °C).The crystalline product was filtered off, washed with water (2×20 mL), then dried under high vacuum to yield 27.2 g 4-amino-3,5-difluorobenzonitrile **13** (17.7 mmol, 76%) as a white solid.

TLC: R_f = 0.25 (CH_2Cl_2:cH 1:1). **^{1}H NMR (400 MHz, $CDCl_3$):** δ = 7.15 (dd, *J* = 6.0 Hz, 2.3 Hz, 2H), 4.27 (s, 2H) ppm. **^{13}C NMR (101 MHz, $CDCl_3$):** δ = 152.2 (d, *J* = 9 Hz), 149.7 (d, *J* = 8.9 Hz), 130.1 (t, *J* = 15.7 Hz), 118.4 (t, *J* = 3.5 Hz), 116.1 – 115.7 (m), 98.8 (t, *J* = 11.0 Hz) ppm. **^{1}H NMR (300 MHz, DMSO-d$_6$):** *δ* = 7.15 (dd, *J* = 6.0, 2.2 Hz, 2H), 4.27 (br s, 2H) ppm. **^{13}C NMR (101 MHz, DMSO-d$_6$):** *δ* = 150.6 (dd, *J* = 243.5, 8.9 Hz), 129.7 (t, *J* = 15.7 Hz), 118.1 (s), 115.6 (dd, *J* = 16.1, 8.5 Hz), 98.5 (t, *J* = 11.1 Hz) ppm. **^{19}F NMR (377 MHz, $CDCl_3$):** *δ* = −135.08 (s) ppm. **HRMS (EI+):** *m/z* calcd for $C_7H_4F_2N_2$: 154.0343 Da [M], found: 154.0344 Da (Δ = 0.6 ppm). **IR (ATR):** ṽ = 3375 (w), 3314 (w), 3204 (w), 2225 (w), 1648 (w), 1608 (w), 1579 (m), 1524 (m), 1511 (m), 1443 (m), 1337 (m), 1163 (m), 1120 (w), 977 (m), 876 (m), 862 (m), 728 (w), 664 (m), 614 (m), 537 (m), 470 (w), 458 (m), 413 (w) cm^{-1}. **elemental analysis calcd for $C_7H_4F_2N_2$ (%):** C: 54.55, H: 2.62, F: 24.65, N: 18.18, found: C: 54.56, H: 2.44, N: 18.29.

4-Amino-3,5-difluorobenzoic acid (14)

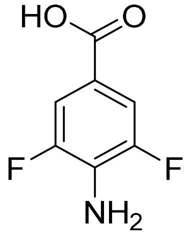

The substrate 4-amino-3,5-difluorobenzonitrile **13** (9.11 g, 59.1 mmol, 1.00 eq.) was suspended in NaOH (313 mL, 313 mmol, 5.30 eq.) and refluxed for 1 h to dissolve the substrate and stirred further at rt for 1 d. After cooling down to rt, conc. HCl was added until the solution reached pH 1. The precipitate was filtered off and dried by lyophilization, yielding 9.45 g 4-amino-3,5-difluorobenzoic acid **14** (55.7 mmol, 94%).

TLC: R_f = 0.33 (1% formic acid, 30% cH in EtOAc). **^{1}H NMR (400 MHz, DMSO-d$_6$):** *δ* = 12.71 (s, 1H), 7.40 (d, *J* = 9.7 Hz, 2H), 6.06 (s, 2H) ppm. **^{13}C NMR (101 MHz, DMSO-d$_6$):** *δ* = 166.5 (t, *J* = 3.2 Hz), 151.4 (d, *J* = 9.3 Hz), 149.0 (d, *J* = 9.5 Hz), 131.1 (t, *J* = 16.6 Hz), 116.1 (t, *J* = 7.7 Hz), 112.7 (dt, *J* = 15.7, 7.8 Hz) ppm. **^{19}F NMR (377 MHz, DMSO-d$_6$):** *δ* = −135.99. **HRMS (EI+):** *m/z* calcd for

$C_7H_5O_2N_1F_2$: 173.0288 Da [M], found: 173.0290 Da (Δ = 1.0 ppm). **IR (ATR):** ṽ = 3497 (vw), 3450 (vw), 3400 (w), 2836 (w), 2605 (w), 1862 (vw), 1693 (m), 1632 (m), 1587 (m), 1537 (w), 1449 (m), 1419 (m), 1336 (m), 1275 (m), 1239 (m), 1147 (w), 1082 (vw), 962 (w), 953 (w), 890 (w), 765 (m), 716 (w), 628 (w) 556 (w), 456 (w), 385 (vw) cm^{-1}.

(4-Amino-3,5-difluorophenyl)methanol (15)

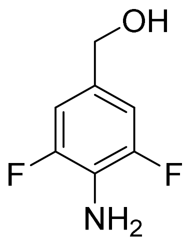

$LiAlH_4$ (2.61 g, 68.7 mmol, 1.20 eq.) was added to anhydrous THF (72 mL) in a two-neck flask under an argon atomsphere. The substrate 4-amino-3,5-difluorobenzoic acid **14** (12.0 g, 57.3 mmol, 1.00 eq.) was dissolved in anhydrous THF (72 mL) and was added slowly at 0 °C. The mixture was stirred for 1 h at rt. Then additional $LiAlH_4$ (2.61 g, 68.7 mmol, 1.20 eq.) was added and the mixture was refluxed for 2 h. The reaction was quenched by addition of EtOAc (50 mL) and H_2O (10 mL) under argon stream. Aqueous solution of NaOH (1.0 M) was added until the solution reached pH 8-10 and the undesired precipitated salts were filtered off and washed with EtOAc (3×50 mL). The liquid was extracted with EtOAc (5×100 mL), dried over Na_2SO_4 and evaporated under reduced pressure. The crude product was purified by silica gel column chromatography with 1% Et_3N in EtOAc/cH 1:1 to obtain 7.38 g of (4-amino-3,5-difluorophenyl)methanol **15** (46.4 mmol, 81%) were obtained.[6]

TLC: R_f = 0.35 (EtOAc/cH 1:1 + 1% Et_3N). **^{1}H NMR (400 MHz, DMSO-d$_6$):** δ = 6.84 (d, *J* = 9.7 Hz, 2H), 5.13 (t, *J* = 5.8 Hz, 1H), 5.04 (s, 2H), 4.34 (d, *J* = 5.8 Hz, 2H) ppm. **^{13}C NMR (101 MHz, DMSO-d$_6$):** δ = 151.5 (dd, *J* = 238.4, 9.3 Hz), 130.2 (t, *J* = 7.2 Hz), 124.2 (t, *J* = 16.8 Hz), 109.7 – 109.4 (m), 62.3 – 61.7 (m) ppm. **^{19}F NMR (376 MHz, DMSO-d$_6$):** δ = −135.90 ppm. **HRMS (FAB+):** *m/z* calcd for $C_7H_7O_1N_1F_2$: 159.0496 Da [M], found: 159.0.498 Da (Δ = 1.3 ppm). **IR (ATR):** ṽ = 3414 (w), 3176 (w), 2945 (w), 1645 (vw), 1594 (m), 1519 (m), 1472 (w), 1447 (m), 1361 (w), 1322 (m), 1158 (m), 1124 (w), 1033 (m), 1001 (m), 954 (m), 845 (m), 774 (m), 720 (m), 667 (m), 568 (m), 511 (w), 478 (w), 385 (w) cm^{-1}

4-Amino-3,5-difluorobenzyl acetate (16)

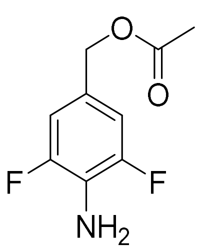

Ac_2O (2.57 mL, 27.2 mmol, 1.00 eq.) was added to a solution of (4-amino-3,5-difluorophenyl)methanol **15** (5.20 g, 32.7 mmol, 1.20 eq.) in pyridine (20 mL) and the reaction was stirred at 0 °C for 2 h. The mixture was extracted with EtOAc (3×100 mL) and washed with brine (2×10 mL), then with $NaHCO_3$ (2×10 mL) and dried over anhydrous Na_2SO_4. The solvent was removed under reduced pressure. The product was purified by column chromatography with 20% EtOAc in cH (R_f = 0.25) and 1.42 g (4-amino-3,5-difluorophenyl)methanol (8.93 mmol, 33%) were recovered. The product 4-amino-3,5-difluorobenzyl acetate **16** (3.55 g 17.6 mmol, 64%) was obtained as a white solid.

^{1}H NMR (400 MHz, DMSO-d$_6$): δ = 6.94 (m, 2H), 5.29 (s, 2H), 4.89 (s, 2H), 2.03 (s, 3H) ppm. **^{13}C NMR (101 MHz, DMSO-d$_6$):** δ = 170.3, 151.8 (d, *J* = 9.5 Hz), 149.5 (d, *J* = 9.6 Hz), 125.5 (t, *J* = 16.6 Hz), 122.3 (t, *J* = 8.3 Hz), 111.4 (m), 64.7 (t, *J* = 2.0 Hz), 20.8 ppm. **^{19}F NMR (376 MHz, DMSO-d$_6$):** δ = −135.81 ppm. **HRMS (EI+):** *m/z* calcd for $C_9H_9O_2N_1F_2$: 201.0601 Da [M], found: 201.0602 Da (Δ = 0.5 ppm). **IR (ATR):** ṽ = 3369 (w), 1731 (m), 1646 (w), 1592 (m), 1527 (m), 1451 (m), 1381 (w), 1363 (m), 1228 (m), 1150 (m), 1024 (m), 953 (m), 847 (w), 722 (w), 665 (w), 633 (w), 601 (w), 573 (w), 452 (w) cm^{-1}.

1,3-Difluoro-2-nitrosobenzene (17)

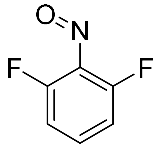

Oxone® (6.54 g, 21.3 mmol, 4.00 eq) was dissolved in 106 mL of diH_2O. 2,6-difluoroaniline (761 mg, 5.32 mmol, 1.00 eq) was dissolved in 27 mL of CH_2Cl_2. The solutions were combined and stirred for 2 h at rt. The phases were separated, and the organic phase was washed with 1 M HCl solution, half saturated NaCl solution and was dried over Na_2SO_4. The solvent was removed under reduced pressure. The product **17** was obtained as a grey solid (622 mg, 4.35 mmol, 82%), which was used without further purification.[3]

HRMS (EI+): *m/z* calcd for $C_6H_3ONF_2$ [M+H] = 143.0177 Da, found: 143.0176 Da (Δ = 1.0 ppm).

***(E)*-4-((2,6-difluorophenyl)diazenyl)-3,5-difluorobenzyl acetate (18)**

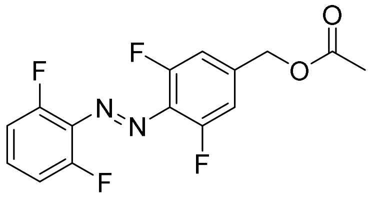

Freshly prepared 1,3-difluoro-2-nitrosobenzene **17** (405 mg, 2.83 mmol, 1.90 eq) was added to a solution of 4-amino-3,5-difluorobenzyl acetate **16** (300 mg, 1.49 mmol, 1.00 eq) in 7.5 mL AcOH/toluene/TFA (6:6:1). The mixture was stirred for 3 d at rt. After removing the solvent under reduced pressure, column chromatography was used to purify the crude product (0.5% CHCOOH,

10% EtOAc in cH). 215 mg of *(E)*-4-((2,6-difluorophenyl)diazenyl)-3,5-difluorobenzyl acetate **18** (0.660 mmol, 44%) were obtained as red solid.

TLC: R_f= 0.20 (developed in 0.5% FA, 10% EtOAc in cH) **^{1}H NMR (300 MHz, CDCl$_3$):** δ = 7.44 – 7.32 (m, 1H), 7.11 – 7.01 (m, 4H), 5.13 (s, 2H), 2.17 (s, 3H). ppm. **^{13}C NMR (101 MHz, CDCl$_3$)**: δ = 170.4, 170.4, 157.2 – 156.7 (m), 154.5 – 153.9 (m), 153.2 – 152.6 (m), 150.7 – 150.1 (m), 141.1 – 140.4 (m), 139.3 – 138.5 (m), 132.1 – 131.0 (m), 129.9 (t, *J* = 9.5 Hz), 112.8 – 112.7 (m), 112.6 – 112.5 (m), 112.3 – 112.2 (m), 112.1 – 112.0 (m), 111.8 – 111.6 (m), 111.6 – 111.4 (m), 111.2 – 111.0 (m), 111.0 – 110.8 (m), 64.6 – 64.3 (m), 64.3 – 64.1 (m), 20.8 ppm. The occurrence of multiplets can be traced back to the C-F coupling and additional peaks can be assigned to the *Z*-isomer (62%).**^{19}F NMR (376 MHz, CDCl$_3$):** δ = -122.90, -123.76, -124.49, -125.43. ppm. Additional peaks can be assigned to the *Z*-isomer (62%). **HRMS (FAB):** calcd. for $C_{15}H_{10}F_4N_2O_2$ [M+H] = 327.0757 Da, found 327.0756 Da (Δ= 0.18 ppm). **IR (ATR):** ṽ = 3444 (vw), 3342 (vw), 3221 (vw), 3098 (vw), 2944 (vw), 1948 (vw), 1741 (m), 1703 (w), 1690 (w), 1679 (m), 1628 (m), 1609 (m), 1577 (m), 1541 (w), 1490 (vw), 1473 (w), 1465 (m), 1448 (m), 1431 (w), 1378 (w), 1361 (w), 1349 (w), 1329 (vw), 1278 (w), 1235 (m), 1211 (m), 1149 (w), 1123 (w), 1060 (w), 1043 (m), 1028 (m), 1003 (w), 969 (w), 924 (m), 884 (w), 849 (m), 790 (m), 745 (m), 714 (w), 663 (vw), 645 (w), 602 (w), 592 (w), 579 (w), 538 (w), 526 (w), 506 (w), 472 (w), 452 (w), 419 (vw), 384 (w) cm^{-1}.

(E)-(4-((2,6-difluorophenyl)diazenyl)-3,5-difluorophenyl)methanol (2)

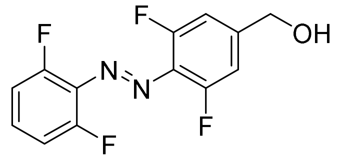

(E)-4-((2,6-difluorophenyl)diazenyl)-3,5-difluorobenzyl acetate **18** (200 mg, 0.613 mmol, 1.00 eq) was dissolved in 61 mL conc. HCl/MeOH 1:80. The solution was stirred at 60 °C for 3 h. Subsequently, the solvent was removed under reduced pressure and the residual oil was purified by column chromatography (30% EtOAc in cH). The product *(E)*-(4-((2,6-difluorophenyl)diazenyl)-3,5-difluorophenyl)methanol **2** (164 mg, 0.577 mmol, 94%) was obtained as red solid.

TLC: R_f= 0.25 (developed in 30% EtOAc in cH) **^{1}H NMR (400 MHz, DMSO-d$_6$):** δ = 7.66-7.58 (m, 1H), 7.39 – 7.33 (m, 2H), 7.29-7.26 (m, 2H), 5.63 (t, *J* = 5.8 Hz, 1H), 4.60 (d, *J* = 5.8 Hz, 2H). **^{13}C NMR (101 MHz, DMSO-d$_6$)**: δ = 156.0 (d, *J* = 4.4 Hz), 155.9 (d, *J* = 4.1 Hz), 153.5 (d, *J* = 4.9 Hz), 153.3 (d, *J* = 4.2 Hz), 150.1 (t, *J* = 9.2 Hz), 132.9 (t, *J* = 10.5 Hz), 130.8 (t, *J* = 10.1 Hz), 128.9 (t, *J* = 9.8 Hz), 113.3 (d, *J* = 3.5 Hz), 113.1 (d, *J* = 3.6 Hz), 110.2 (d, *J* = 3.3 Hz), 110.0 (d, *J* = 3.0 Hz), 61.7 ppm. **^{19}F NMR (376 MHz, DMSO-d$_6$):** δ = −121.21, −122.14 ppm. **HRMS (EI+):** *m/z* calcd. for $C_{13}H_8F_4N_2O$ [M+H] = 284.0573 Da, found 284.0572 Da (Δ= 0.14 ppm). **IR (ATR):** ṽ = 3293 (vw), 3186 (vw), 3177 (vw), 3109 (vw), 3068 (vw), 2945 (vw), 2897 (vw), 2856 (vw), 1625 (w), 1612 (w), 1575 (w), 1469 (w), 1445 (w), 1429 (w), 1358 (w), 1329 (vw), 1295 (vw), 1281 (vw), 1242 (w), 1221 (vw), 1200 (vw), 1126 (vw), 1069 (w), 1064 (w), 1047 (w), 1024 (w), 986 (vw), 972 (w), 887

(vw), 880 (vw), 846 (w), 812 (vw), 788 (w), 742 (w), 715 (w), 666 (vw), 606 (w), 577 (vw), 537 (vw), 524 (vw), 507 (vw), 475 (vw), 422 (w), 409 (vw), 382 (vw) cm^{-1}

(*E*)-4-(2,6-difluorophenyl)diazenyl-3,5-difluorobenzaldehyde (4)

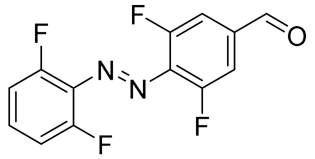

(*E*)-(4-((2,6-Difluorophenyl)diazenyl)-3,5-difluorophenyl)methanol **2** (150 mg, 0.528 mmol, 1.00 eq) was dissolved in 2.6 mL of CH_2Cl_2. Activated MnO_2 (298 mg, 3.43 mmol, 6.5 eq) was added subsequently and the mixture was stirred for 48 h at rt. The residual dark red oil was purified by column chromatography (CH_2Cl_2) to yield 90.0 mg (0.319 mmol, 60%) of 4-(2,6-difluorophenyl)diazenyl-3,5-difluorobenzaldehyde **2** as pale-red solid, 23% of the starting material were recovered.

^{1}H NMR (400 MHz, $CDCl_3$): δ = 9.98 (s, 1H), 7.65 – 7.54 (m, 2H), 7.45 (tt, *J* = 8.5, 5.9 Hz, 1H), 7.15 – 7.05 (m, 2H) ppm. **^{13}C NMR (101 MHz, $CDCl_3$)**: δ =188.9 (t, *J* = 2.0 Hz), 157.3 (d, *J* = 3.7 Hz), 156.9 (d, *J* = 3.7 Hz), 154.7 (d, *J* = 3.8 Hz), 154.2 (d, *J* = 4.0 Hz), 137.6 (t, *J* = 7.6 Hz), 135.7 (t, *J* = 11.0 Hz), 132.9 (t, *J* = 10.7 Hz), 131.7 (t, *J* = 9.4 Hz), 113.7 – 113.5 (m), 113.5 – 113.3 (m), 113.1 (d, *J* = 4.0 Hz), 112.9 (d, *J* = 3.7 Hz) ppm. **^{19}F NMR (376 MHz, $CDCl_3$):** δ = −119.09, −119.82. **HRMS (EI+):** *m/z* calcd. for $C_{13}H_6F_4N_2O$ [M+H] = 282.0416 Da, found 282.0417 Da (Δ= 0.18 ppm). **IR (ATR):** ṽ = 3105 (vw), 2883 (vw), 2752 (vw), 1698 (w), 1612 (w), 1587 (vw), 1572 (w), 1483 (vw), 1466 (w), 1445 (w), 1383 (w), 1322 (w), 1313 (w), 1299 (vw), 1281 (vw), 1239 (w), 1220 (w), 1200 (w), 1153 (vw), 1116 (w), 1051 (w), 1026 (w), 989 (w), 882 (vw), 861 (w), 813 (vw), 793 (w), 747 (w), 722 (w), 703 (w), 608 (w), 582 (w), 543 (w), 521 (w), 506 (w), 482 (w), 459 (vw), 443 (vw), 428 (vw), 416 (w), 395 (vw), 382 (w) cm^{-1}

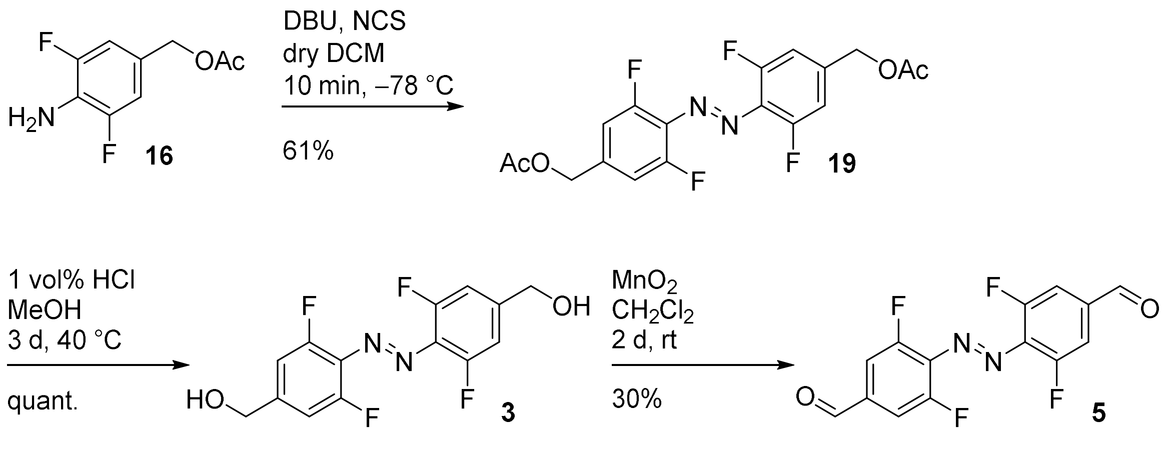

*Scheme S 2: Synthesis of (E)-4,4'-(diazene-1,2-diyl)bis(3,5-difluorobenzaldehyde) (**5**) (**TFAB-4,4'-bisaldehyde**).*

(E)-(diazene-1,2-diylbis(3,5-difluoro-4,1-phenylene))bis(methylene) diacetate (19)

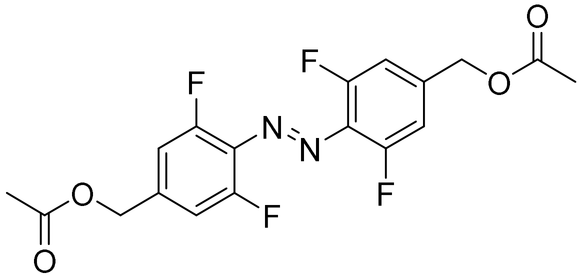

DBU (4.94 mL, 33.1 mmol, 2.00 eq.) was added to a solution of 4-amino-3,5-difluorobenzyl acetate **16** (3.33 g, 16.5 mmol, 1.00 eq.) in dry CH_2Cl_2 (248 mL) under argon atmosphere. The solution was stirred at rt for 5 min before being cooled down to −78 °C. Powdered NCS (4.42 g, 33.1 mmol, 2.00 eq.) was added to the reaction mixture. The orange solution was stirred for 10 min at −78 °C before quenching by addition of a sat. $NaHCO_3$. The organic layer was separated, washed sequentially with water (100 mL) and diluted aqueous solution of HCl (1.0 mol/L, 100 mL), dried over anhydrous Na_2SO_4, and concentrated to dryness *in vacuo*. The crude product was purified by silica gel column chromatography in 1% EtOAc in CH_2Cl_2 (R_f = 0.35) and 2.00 g *(E)*-(diazene-1,2-diylbis(3,5-difluoro-4,1-phenylene))bis(methylene) diacetate **19** (5.03 mmol, 61%) was obtained as an orange solid. (*symmetric DBU/NCS coupling: cf.*[6])

^{1}H NMR (400 MHz, DMSO-d$_6$): δ = 7.38 (d, *J* = 10.1 Hz, 4H), 5.17 (s, 4H), 2.13 (s, 6H) ppm. **^{13}C NMR (101 MHz, DMSO-d$_6$):** δ = 170.1, 155.8 (d, *J* = 4.6 Hz), 153.3 (d, *J* = 4.8 Hz), 142.7, 129.7, 111.7 (dd, *J* = 21.4, 3.1 Hz), 63.7, 20.6 ppm. **^{19}F NMR (376 MHz, DMSO-d$_6$):** δ = −125.37 ppm. **HRMS (FAB+):** *m/z* calcd for $C_{18}H_{15}O_4N_2F_4$: 399.0968 Da [M+H], found: 399.0970 Da (Δ = 0.5 ppm). **IR (ATR):** ṽ = 2936 (vw), 1738 (w), 1627 (w), 1577 (w), 1438 (w), 1383 (w), 1362 (w), 1234 (m), 1040 (m), 972 (w), 923 (w), 850 (m), 752 (vw), 665 (w), 586 (w), 529 (w), 455 (w), 386 (vw) cm^{-1}. **UV-Vis (MeCN):** λ_{max} = 229, 315, 454 nm.

(*E*)-(diazene-1,2-diylbis(3,5-difluoro-4,1-phenylene))dimethanol (3)

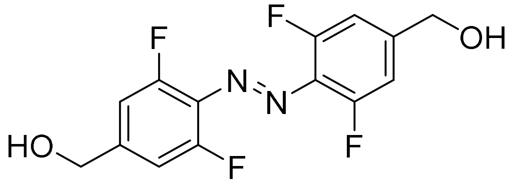

(E)-(diazene-1,2-diylbis(3,5-difluoro-4,1-phenylene))bis(methylene) diacetate **19** (1.98 g, 4.97 mmol, 1.00 eq.) was dissolved in a solution of conc. HCl (37%):methanol 1:100 (500 mL)[8] and the resulting solution stirred at 40 °C for 72 h. The reaction was diluted with PhMe (100 mL) and the solvent was removed under reduced pressure. The crude product was purified by silica gel column chromatography and the 1.56 g *(E)*-(diazene-1,2-diylbis(3,5-difluoro-4,1-phenylene))dimethanol **3** (4.96 mmol, quant.) was obtained as an orange solid.

TLC: R_f = 0.20 in 20% EtOAc in CH_2Cl_2 **^{1}H NMR (400 MHz, DMSO-d$_6$):** δ = 7.26 (d, *J* = 11.3 Hz, 4H), 5.63 (s, 2H), 4.60 (s, 4H) ppm. **^{13}C NMR (101 MHz, DMSO-d$_6$):** δ = 155.3 (d, *J* = 4.5 Hz), 152.8 (d, *J* = 4.6 Hz), 149.0 (t, *J* = 9.2 Hz), 128.4 (t, *J* = 9.9 Hz), 109.4 (dd, *J* = 20.8, 2.9 Hz), 61.0 ppm. **^{19}F NMR (376 MHz, DMSO-d$_6$):** δ = −121.4 ppm. **HRMS (FAB+):** *m/z* calcd for $C_{14}H_{11}O_2N_2F_4$: 315.0757 Da [M+H], found: 315.0756 Da (Δ = 0.3 ppm). **IR (ATR):** ṽ = 3286 (w), 1623 (m), 1574 (m), 1441 (m), 1359 (w), 1297 (w), 1200 (w), 1125 (w), 1067 (m), 1041 (m), 971 (m), 881 (w), 845 (m), 748 (w), 658 (w), 580 (w), 543 (w), 520(w) cm^{-1}. **UV-Vis (MeCN):** λ_{max} = 230, 318, 454 nm.

(*E*)-4,4'-(diazene-1,2-diyl)bis(3,5-difluorobenzaldehyde) (5)

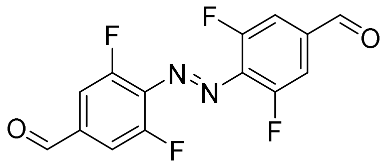

(E)-(diazene-1,2-diylbis(3,5-difluoro-4,1-phenylene))dimethanol **3** (100 mg, 0.318 mmol, 1.00 eq.) was dissolved in 13 mL of MeCN. Activated MnO_2 (1.09 g, 12.6 mmol, 10.0 eq) was added subsequently. The mixture was stirred for 8 h at rt. The residual dark red oil was purified by filtration column chromatography (CH_2Cl_2) to yield (*E*)-4,4'-(diazene-1,2-diyl)bis(3,5-difluorobenzaldehyde) **5** as purple crystals (30 mg, 0.097 mmol, 30%).

^{1}H NMR (400 MHz, DMSO-d$_6$): δ = 10.04 (s, 2H), 7.94 – 7.91 (m, 4H) ppm. **^{13}C NMR (101 MHz, DMSO-d$_6$):** δ = 190.5 , 156.1 (d, *J* = 3.6 Hz), 153.5 (d, *J* = 3.6 Hz), 139.0 (t, *J* = 7.9 Hz), 133.8 (t, *J* = 10.3 Hz), 113.8 (dd, *J* = 20.9, 3.3 Hz)ppm. **^{19}F NMR (376 MHz, DMSO-d$_6$):** δ = −119.29 ppm. **HRMS (FAB+):** *m/z* calcd for $C_{14}H_6O_2N_2F_4$: 310.0365 Da [M+H], found: 310.0363 Da (Δ = -0.65 ppm). **IR (ATR):** ṽ = 3074 (w), 2873 (w), 1688 (s), 1617 (w), 1569 (s), 1443 (s), 1388 (m), 1323 (m), 1307 (m), 1204 (m), 1117 (m), 1053 (s), 1000 (m), 988 (s), 895 (w), 869 (s), 788 (m), 755 (w), 714 (m), 613 (s), 588 (m), 532 (m), 489 (m), 456 (m), 456 (w) cm^{-1}. **UV-Vis (MeCN):** λ_{max} = 196, 314, 475 nm.

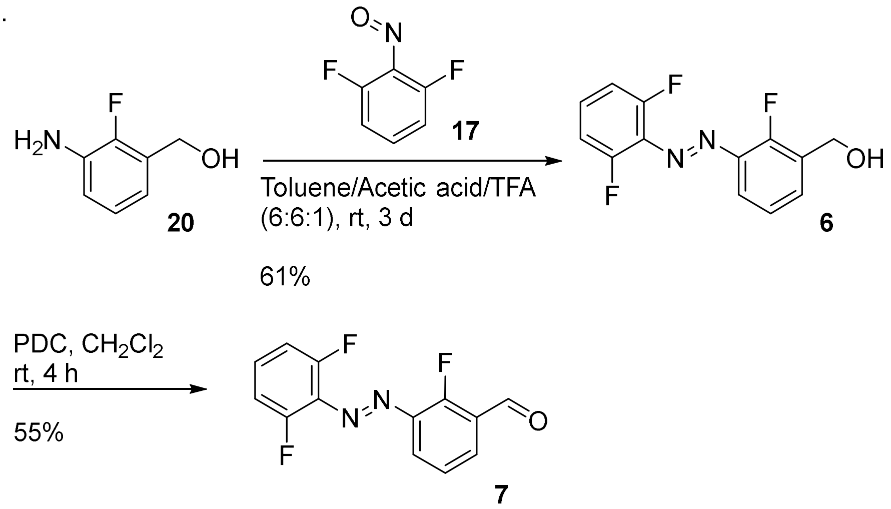

*Scheme S 3: Synthesis of (E)-3-((2,6-difluorophenyl)diazenyl)-2-fluorobenzaldehyde (**7**). This time the acetylation side reaction was not observed in the reaction of **20** and **17** leading to **6**.*

(E)-(3-((2,6-difluorophenyl)diazenyl)-2-fluorophenyl)methanol (6)

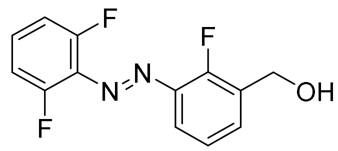

1,3-difluoro-2-nitrosobenzene **17** (2.50 g, 17.5 mmol, 1.30 eq) was dissolved in 54 mL of acetic acid. Subsequently, (3-amino-2-fluorophenyl)methanol **20** (1.65 g, 13.4 mmol, 1.00 eq) was added and the mixture was stirred for 48 h at rt. The residual dark oil was purified by column chromatography using 5 to 20% EtOAc in cH. (*E*)-(3-((2,6-difluorophenyl)diazenyl)-2-fluorophenyl)methanol **6** was obtained as pale-red solid (548 mg, 2.06 mmol, 61%).

^{1}H NMR (400 MHz, DMSO-d$_6$): δ = 7.73 (td, *J* = 7.2, 1.7 Hz, 1H), 7.63 – 7.53 (m, 2H), 7.35 (td, *J* = 8.7, 2.2 Hz, 3H), 5.48 (t, *J* = 5.7 Hz, 1H), 4.69 (d, *J* = 5.6 Hz, 2H). ppm. **^{13}C NMR (101 MHz, DMSO-d$_6$)**: δ = 156.1 (d, J = 4.4 Hz), 153.5 (d, J = 4.4 Hz), 152.5, 144.4, 131.7 (t, J = 10.4 Hz), 130.4 (d, J = 20.8 Hz), 130.4, 129.3, 121.7, 119.5, 113.2 – 113.1 (m), 113.0 – 112.9 (m), 62.3 ppm. **^{19}F NMR (376 MHz, DMSO-d$_6$):** δ = −126.86 ppm. **TLC:** R_F= 0.25 (developed in 10% EtOAc in cH) **HRMS (EI+):** *m/z* calcd. for $C_{13}H_{10}F_2N_2O$ [M+H] = 248.0761 Da, found 248.0761 Da (Δ= 0.73 ppm). **IR (ATR):** ṽ = 3199 (w), 2875 (w), 1936 (vw), 1612 (m), 1586 (m), 1462 (m), 1440 (m), 1310 (w), 1281 (w), 1236 (m), 1197 (w), 1125 (w), 1081 (vw), 1022 (m), 918 (w), 880 (w), 787 (m), 723 (m), 683 (m), 581 (m), 508 (w), 493 (m), 463 (w), 443 (w) cm^{-1}.

***(E)*-3-((2,6-difluorophenyl)diazenyl)-2-fluorobenzaldehyde (7)**

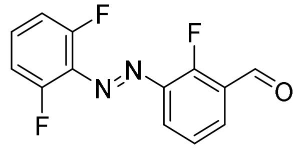

(*E*)-(3-((2,6-difluorophenyl)diazenyl)-2-fluorophenyl)methanol **6** (500 mg, 1.88 mmol, 1.00 eq) and PDC (848 mg, 2.25 mmol, 1.20 eq) were dried in a schlenk flask. 9.4 mL of dry CH_2Cl_2 were added subsequently under an argon atmosphere.[4] The mixture was stirred for 2 h at rt. The mixture was filtered through silica and the solvent was removed under reduced pressure to yield 275 mg (*E*)-3-((2,6-difluorophenyl)diazenyl)-2-fluorobenzaldehyde **7** (1.04 mmol, 55%) as a red solid.

TLC: R_f= 0.43 (developed in toluene). **^{1}H NMR (300 MHz, DMSO-d$_6$):** δ = 10.35 (s, 1H), 8.09 (ddd, *J* = 7.9, 6.4, 1.8 Hz, 1H), 7.95 (td, *J* = 7.7, 1.8 Hz, 1H), 7.65 (tt, *J* = 8.1, 6.1 Hz, 1H), 7.55 (t, *J* = 7.8 Hz, 1H), 7.42 – 7.34 (m, 2H) ppm. **^{13}C NMR (101 MHz, DMSO-d$_6$)**: δ = 187.3 (d, *J* = 6.1 Hz), 161.7, 159.0, 156.3 (d, *J* = 4.1 Hz), 153.8 (d, *J* = 4.0 Hz), 153.0, 151.6 (d, *J* = 5.4 Hz), 150.4, 149.1 (d, *J* = 5.6 Hz), 140.8 (d, *J* = 6.3 Hz), 133.4 – 132.4 (m), 131.2 (t, *J* = 9.7 Hz), 130.2 (t, *J* = 9.8 Hz), 126.3 (t, *J* = 1.5 Hz), 125.6 (d, *J* = 7.0 Hz), 125.4 (t, *J* = 4.7 Hz), 124.6 (d, *J* = 7.4 Hz), 122.8, 113.4 (d, *J* = 3.6 Hz), 113.2 (d, *J* = 3.3 Hz), 112.8 (d, *J* = 4.2 Hz), 112.6 (d, *J* = 4.0 Hz) ppm. The occurrence of multiplets can be traced back to the C-F coupling and additional peaks can be assigned to the *Z*-isomer (32%). **^{19}F NMR (376 MHz, DMSO-d$_6$):** δ = −121.20, −132.82 ppm.

^{1}H NMR (400 MHz, CD_3CN) δ 10.40 (s, 1H), 8.04 (ddd, *J* = 8.0, 6.4, 1.8 Hz, 1H), 7.94 (ddd, *J* = 8.0, 7.3, 1.8 Hz, 1H), 7.53 (tt, *J* = 8.5, 6.0 Hz, 1H), 7.46 (t, *J* = 7.9 Hz, 1H), 7.20 (dd, *J* = 9.5, 8.5 Hz, 2H). **^{13}C NMR (101 MHz, CD_3CN)** δ 187.83 (d, *J* = 7.0 Hz), 163.57 , 160.89 , 158.04 (d, *J* = 4.2 Hz), 155.46 (d, *J* = 4.2 Hz), 142.43 (d, *J* = 6.5 Hz), 133.62 (t, *J* = 10.8 Hz), 133.32 (d, *J* = 2.5 Hz), 131.68 , 126.84 (d, *J* = 7.2 Hz), 126.02 (d, *J* = 4.7 Hz), 123.76 , 113.89 (d, *J* = 24.1 Hz). **^{19}F NMR (376 MHz, CD_3CN)** δ -126.67, -139.27. **HRMS (EI+):** *m/z* calcd. for $C_{13}H_7F_3N_2O$ [M+H] = 264.0510 Da, found 264.0508 Da (Δ= 0.77 ppm). **IR (ATR, ṽ) =** 2898 (vw), 1696 (w), 1611 (w), 1579 (w), 1467 (w), 1409 (w), 1297 (w), 1252 (w), 1217 (w), 1193 (w), 1164 (w), 1030 (w), 945 (w), 825 (vw), 787 (m), 736 (w), 716 (w), 646 (w), 604 (vw), 528 (w), 508 (w), 476 (w) cm^{-1}.

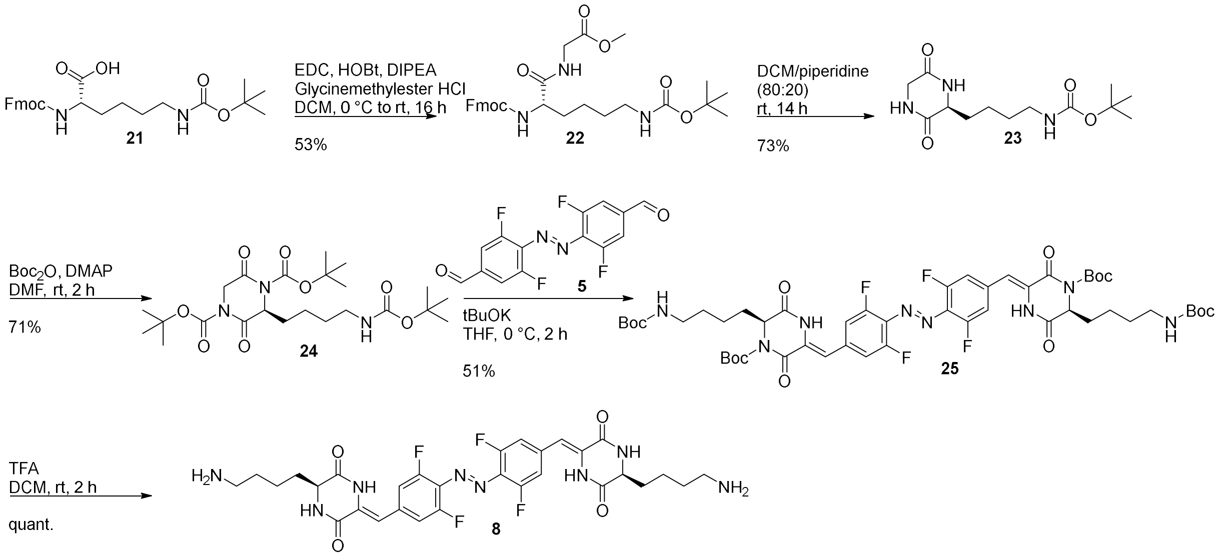

*Scheme S 4: Synthesis of Bis-(cyclo(Lys)-(2,6-difluoro-4-vinyl-azobenzene) (**8**).*

Fmoc-Lys(Boc)-Gly-OMe (22)

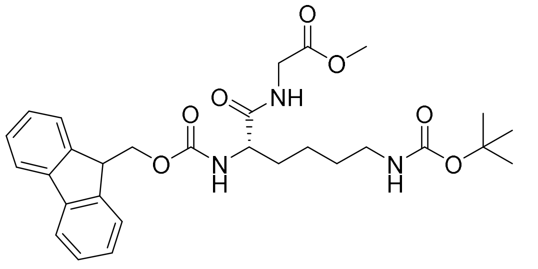

(2~{S})-2-(9~{H}-fluoren-9-ylmethoxycarbonylamino)-6-[(2-methylpropan-2-yl)oxycarbonylamino]hexanoic acid (15.0 g, 32.0 mmol, 1.00 eq), 1-hydroxybenzotriazole;hydrate (5.93 g, 38.7 mmol, 1.21 eq) and 3-(ethyliminomethylideneamino)propyl-dimethylazanium;chloride (7.43 g, 38.7 mmol, 1.21 eq) were dissolved in in CH_2Cl_2 (604 mL) and cooled to 0 °C. Methyl 2-aminoacetate;hydrochloride (4.02 g, 32.0 mmol, 1.00 eq) and *N*-ethyl-*N*-propan-2-ylpropan-2-amine (13.4 g, 18.5 mL, 104 mmol, 3.24 eq) were added and the reaction was stirred at 0 °C for 1 h. The reaction mixture was warmed up to rt and stirred overnight. Half amount of the solvent was evaporated, the residue was washed with water and dried over Na_2SO_4. The crude was purified by flash column chromatography (99:1 to 95:5 CH_2Cl_2/MeOH) to obtain the product as white foam (9.23 g, 17.1 mmol, 53%).[5]

TLC: R_F = 0.30 developed in 95:5 CH_2Cl_2/MeOH. **^{1}H NMR (400 MHz, CDCl$_3$):** δ = 7.76 (d, *J* = 7.5 Hz, 2H), 7.59 (d, *J* = 7.5 Hz, 2H), 7.40 (t, *J* = 7.4 Hz, 2H), 7.31 (td, *J* = 7.5, 1.2 Hz, 2H), 6.64 (s, 1H), 5.53 (s, 1H), 4.66 (s, 1H), 4.41 (d, *J* = 7.5 Hz, 2H), 4.21 (t, *J* = 6.8 Hz, 2H), 4.08 – 3.99 (m,2H), 3.74 (s, 3H), 3.14 – 3.06 (m, 2H), 1.92 – 1.85 (m, 1H), 1.73 (s, 1H), 1.73 – 1.66 (m ,1H), 1.49 (s, 3H), 1.43 (s, 9H). **^{13}C NMR (101 MHz, CDCl$_3$)**: δ = 172.1, 170.2, 156.4, 143.9, 141.4, 127.9, 127.2, 125.2,

120.1, 79.3, 67.2, 54.8, 52.5, 47.3, 41.3, 40.0, 32.1, 29.7, 28.6, 22.5. ppm. **HRMS (FAB):** *m/z* calcd. for $C_{29}H_{37}N_3O_7$ [M+H] = 540.2710 Da, found 540.2709 Da (Δ = -0.17 ppm). **IR (ATR):** ṽ = 3299 (m), 3065 (vw), 3041 (vw), 2973 (w), 2938 (w), 2861 (w), 1740 (w), 1681 (vs), 1650 (vs), 1527 (vs), 1477 (w), 1446 (s), 1391 (m), 1366 (s), 1343 (w), 1298 (m), 1268 (vs), 1247 (vs), 1232 (vs), 1210 (vs), 1167 (vs), 1102 (s), 1088 (s), 1033 (s), 1018 (s), 1009 (m), 984 (m), 936 (w), 902 (w), 894 (w), 866 (w), 854 (w), 778 (w), 756 (s), 735 (vs), 656 (vs), 645 (vs), 620 (s), 596 (s), 586 (s), 562 (m), 547 (s), 524 (m), 514 (m), 503 (m), 490 (m), 467 (w), 462 (w), 445 (w), 426 (m), 402 (w), 382 (m) cm^{-1}.

cyclo(Lys(Boc)-Gly) (23)

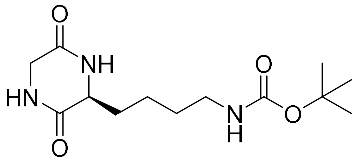

The linear dipeptide Fmoc-L-lys(Boc)-gly-OMe (2 g, 0.370 mmol) was dissolved in DCM/piperidine (80:20; 18.5 mL) and stirred at rt for 14 h in total. Formation of the product can be observed by gelation. The gel was filtered and the filtrate was further stirred overnight. The product was filtrated, washed with CH_2Cl_2 followed by H_2O and dried. To eliminate trapped impurities, the solid was grinded with a mortar and washed again with CH_2Cl_2 followed by H_2O and dried. The product was obtained as white solid (650 mg, 2.28 mmol, 61%)[5]

^{1}H NMR (400 MHz, DMSO-*d*$_6$): *δ* = 8.15 (s, 1H), 7.98 (s, 1H), 6.77 (t, *J* = 5.7 Hz, 1H), 3.84 – 3.59 (m, 3H), 2.89 (q, *J* = 6.4 Hz, 2H), 1.64 (qd, *J* = 9.1, 8.6, 4.7 Hz, 2H), 1.46 (s, 1H), 1.37 (s, 9H), 1.31 – 1.18 (m, 3H). **^{13}C NMR (101 MHz, DMSO-*d*$_6$)**: *δ* = 168.0, 166.1, 155.6, 77.3, 54.1, 44.3, 43.8, 32.5, 29.2, 28.3, 22.4, 21.8, 21.4. ppm. **HRMS (EI):** *m/z* calcd. for $C_{13}H_{23}N_3O_4$ [M+] = 286.1767 Da, found 286.1768 Da (Δ = 0.35 ppm). **IR (ATR, ṽ)** = 3366 (w), 3197 (w), 3166 (w), 3078 (w), 3053 (w), 3013 (vw), 2968 (w), 2955 (w), 2932 (w), 2868 (w), 1677 (vs), 1560 (w), 1521 (vs), 1466 (s), 1449 (m), 1388 (w), 1366 (m), 1334 (s), 1290 (m), 1248 (s), 1230 (w), 1169 (vs), 1142 (m), 1111 (w), 1082 (m), 1054 (w), 1041 (w), 1004 (m), 952 (w), 926 (w), 897 (w), 868 (w), 810 (s), 762 (m), 734 (w), 673 (w), 650 (w), 640 (w), 578 (m), 564 (m), 501 (w), 467 (w), 452 (s), 445 (s), 419 (w), 405 (w), 387 (m) cm^{-1}.

Di-Boc-cyclo(Lys(Boc)-Gly) (24)

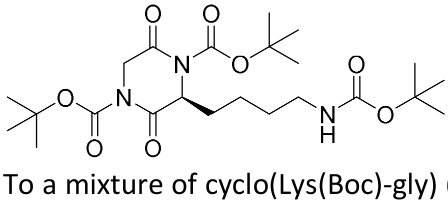

To a mixture of cyclo(Lys(Boc)-gly) (1.00 g, 3.50 mmol, 1.00 eq) and tert-butyl (2-methylpropan-2-yl)oxycarbonyl carbonate (1.61 g, 7.36 mmol, 2.10 eq) in DMF (4.1 mL) was added *N*,*N*-dimethylpyridin-4-amine (903 mg, 7.39 mmol, 2.11 eq) and the reaction mixture was stirred at rt for 2 h. Then, the reaction mixture was diluted with EtOAc, washed with aqueous $KHSO_4$, and

dried over Na_2SO_4. After removal of the solvent, the crude product was purified by column chromatography (3:7 EtOAc/cH). The product was obtained as colorless solid (1.24 g, 2.50 mmol, 71%).

TLC: R_f = 0.40 developed in 3:7 EtOAc/cH. **^{1}H NMR (400 MHz, $CDCl_3$):** δ = 4.88 – 4.66 (m, 2H), 4.54 (s, 1H), 4.14 (d, *J* = 18.4 Hz, 1H), 3.11 (q, *J* = 6.4 Hz, 2H), 1.95 – 1.77 (m, 2H), 1.54 (s, 9H), 1.53 (s, 9H), 1.43 (s, 9H). **^{13}C NMR (101 MHz, $CDCl_3$)**: δ = 166.4, 164.6, 156.1, 150.1, 149.9, 85.2, 85.1, 60.1, 48.9, 40.1, 32.4, 29.6, 28.6, 28.1, 28.0, 23.1. ppm. **HRMS (EI):** *m/z* calcd. for $C_{23}H_{40}N_3O_8$ [M+] = 486.2810 Da, found 486.2808 Da (Δ = -0.47 ppm). **IR (ATR)** ṽ = 3394 (vw), 2979 (w), 2934 (w), 2868 (vw), 1779 (m), 1720 (vs), 1513 (w), 1476 (w), 1456 (w), 1391 (w), 1367 (s), 1282 (vs), 1247 (vs), 1140 (vs), 1004 (w), 973 (w), 936 (w), 928 (w), 870 (w), 846 (m), 775 (m), 739 (w), 633 (w), 605 (w), 589 (w), 565 (w), 523 (w), 511 (w), 487 (w), 477 (w), 462 (w), 439 (w), 412 (w), 404 (w), 397 (w), 382 (w), 375 (w) cm^{-1}.

Bis-(Boc-cyclo(Lys(Boc))-(2,6-difluoro-4-vinyl-azobenzene) (25)

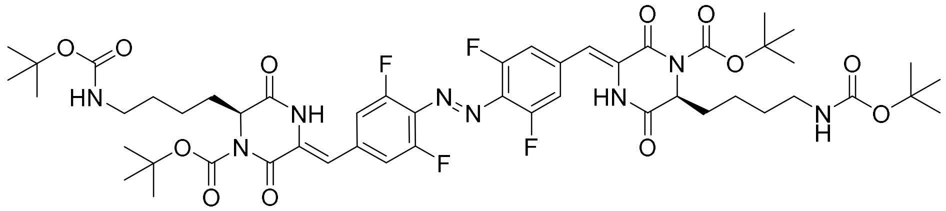

A mixture of (*E*)-4,4'-(diazene-1,2-diyl)bis(3,5-difluorobenzaldehyde) (120 mg, 387 µmol, 1.00 eq) and Di-Boc-cyclo(lys(Boc)-gly) (413 mg, 851 µmol, 2.20 eq) in THF (3.5 mL) was cooled to 0 °C in an ice water bath. Potassium;2-methylpropan-2-olate (96.4 mg, 859 µmol, 2.22 eq) was added and the solution was stirred for 2 h at rt. Then the reaction mixture was partitioned between EtOAc and NH_4Cl. The crude product was purified by column chromatography (3:7 to 4:6 EtOAc/cH). The product was obtained as dark red solid 206 mg, 0.196 mmol, 51%).

TLC: R_f = 0.23 developed in 4:6 EtOAc/cH. **^{1}H NMR (400 MHz, DMSO-d_6):** δ = 10.76 (s, 2H), 7.78 – 7.39 (m, 4H), 6.95 (s, 2H), 6.76 (t, *J* = 5.7 Hz, 2H), 4.62 – 4.38 (m, 2H), 2.89 (q, *J* = 6.3 Hz, 4H), 1.81 (dp, *J* = 20.4, 6.5, 6.1 Hz, 4H), 1.50 (s, 18H), 1.45 – 1.37 (m, 8H), 1.34 (s, 18H). **^{13}C NMR (101 MHz, DMSO-d_6)**: δ = 166.6 , 159.3 , 156.0 (d, *J* = 5.2 Hz), 155.6 , 153.4 (d, *J* = 5.3 Hz), 150.3 , 138.1 (t, *J* = 11.1 Hz), 130.1 (t, *J* = 10.0 Hz), 129.3 , 115.6 , 114.2 (d, *J* = 22.1 Hz), 83.7 , 77.4 , 58.7 , 33.6 , 28.9 , 28.3 , 27.6 , 22.3 . ppm. **^{19}F NMR (376 MHz, DMSO-d_6):** δ = -121.36 ppm. **HRMS (FAB):** *m/z* calcd. for $C_{50}H_{65}N_8O_{12}F_4$ [M+H] = 1045.4653 Da, found 1045.4655 Da (Δ = 0.26 ppm). **IR (ATR):** ṽ = 3369 (vw), 2976 (w), 2932 (w), 2866 (w), 1772 (w), 1691 (vs), 1633 (m), 1616 (m), 1567 (w), 1510 (w), 1477 (w), 1451 (m), 1366 (vs), 1283 (s), 1232 (vs), 1143 (vs), 1047 (s), 1011 (m), 890 (w), 847 (m), 806 (w), 773 (m), 745 (m), 694 (w), 662 (w), 642 (m), 632 (m), 606 (m), 595 (m), 575 (w), 552 (w), 541 (w), 514 (w), 493 (w), 472 (m), 460 (m), 450 (m), 443 (m), 433 (m), 425 (m), 415 (w), 392 (w), 380 (w) cm^{-1}.

Bis-(cyclo(Lys)-(2,6-difluoro-4-vinyl-azobenzene) (8)

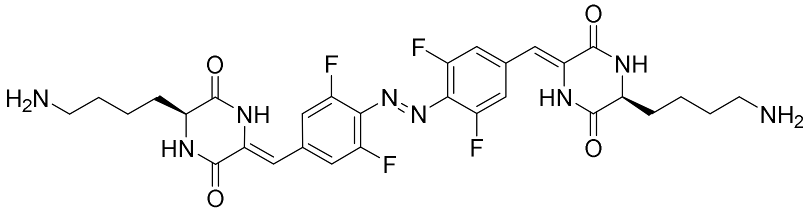

Bis-(Boc-cyclo(Lys(Boc))-(2,6-difluoro-4-vinyl-azobenzene) (295 mg, 266 µmol, 1.00 eq) was dissolved in CH_2Cl_2 (3.70 mL) and 2,2,2-trifluoroacetic acid (3.70 mL) was added. The mixture was stirred for 2 h at rt. Toluene was added (3x) and the solvent was evaporated. No further purification was necessary and the TFA-salt of the product Bis-(cyclo(Lys)-(2,6-difluoro-4-vinyl-azobenzene) was obtained as orange solid (202 mg, 0.266 mmol, 100%)

HPLC: R_t = 8.9 min; 5-95% MeCN in H_2O with 0.1% TFA.**^{1}H NMR (400 MHz, DMSO-d$_6$):** δ = 10.48 (s, 2H), 8.66 (d, *J* = 2.5 Hz, 2H), 7.80 (s, 6H), 7.46 (d, *J* = 11.4 Hz, 4H), 6.71 (s, 2H), 4.13 (td, *J* = 5.4, 2.2 Hz, 2H), 3.46 (s, 8H), 2.79 (q, *J* = 6.6 Hz, 4H), 1.78 (tt, *J* = 14.6, 7.6 Hz, 4H), 1.56 (p, *J* = 7.6 Hz, 4H), 1.49 – 1.30 (m, 4H). **^{13}C NMR (101 MHz, DMSO-d$_6$)**: δ = 167.2 , 159.3 , 156.0 (d, *J* = 5.5 Hz), 153.5 (d, *J* = 5.6 Hz), 138.7 (t, *J* = 11.0 Hz), 130.1 , 129.6 (t, *J* = 9.9 Hz), 114.5 – 112.3 (m), 110.6 , 54.6 , 38.6 , 33.1 , 26.7 , 20.8 . ppm. **^{19}F NMR (376 MHz, DMSO-d$_6$):** δ = -121.62 ppm. **HRMS (ESI):** *m/z* calcd. for $C_{30}H_{32}N_8O_4F_4$ [M+H] = 645.2555 Da, found 645.2544 Da (Δ = -1.70 ppm). **IR (ATR)** $\tilde{\nu}$ = 3187 (w), 3170 (w), 3152 (w), 3064 (w), 3058 (w), 3048 (w), 3033 (w), 2956 (w), 2928 (w), 2874 (w), 2817 (w), 2749 (w), 1679 (vs), 1619 (vs), 1562 (m), 1543 (m), 1534 (m), 1475 (w), 1458 (m), 1425 (vs), 1387 (s), 1351 (s), 1327 (m), 1299 (w), 1200 (vs), 1179 (vs), 1130 (vs), 1050 (vs), 1003 (w), 982 (w), 948 (w), 905 (w), 885 (w), 834 (vs), 798 (vs), 755 (s), 721 (vs), 680 (m), 645 (m), 635 (m), 589 (m), 554 (w), 534 (m), 520 (m), 499 (s), 479 (m), 459 (s), 438 (s), 416 (m), 407 (m), 385 (m) cm^{-1}.

3. Photophysical properties of the compounds 2-8

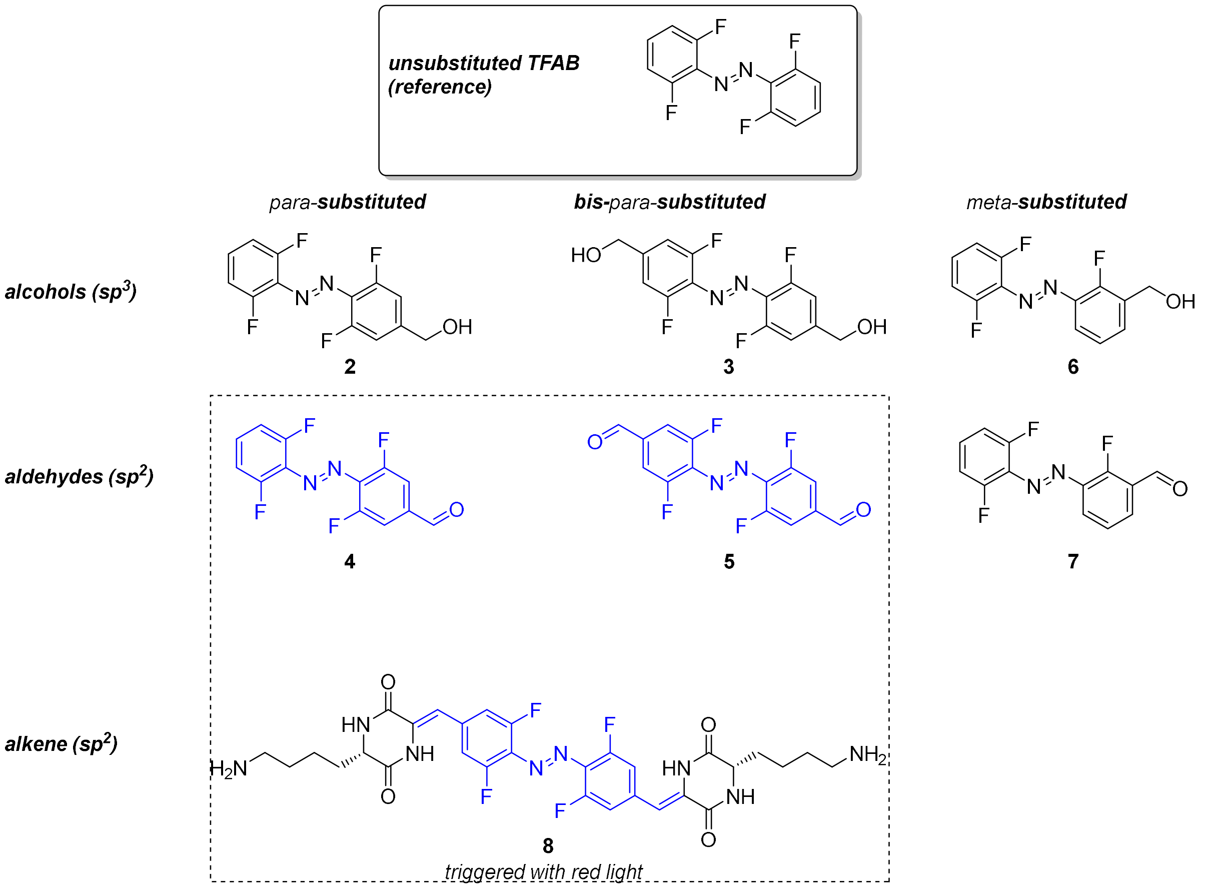

*Figure S 1: Structures of the fluorinated azobenzene derivatives **2-8**.*

Photostationary states determined by 1H NMR measurements

Photostationary states were determined by ^{1}H NMR measurements for samples (10 mg/mL; in some cases – e.g. **5** – when the solubility in d^3-MeCN was lower, the saturated supernatant was taken for the NMR measurements) equilibrated for 8 hours under the indicated light wavelength (λ_{max} of the respective LED light diode; in case of the 660 nm LED additionally equipped with a 630 nm cut-off filter SCHOTT RG-630 to eliminate the <630 nm component of the emitted light). ^{1}H NMR measurements for sample **8** were prepared in DMSO and irradiated for 3 h (660 nm) or 1 h (470 nm). Unsubstituted **TFAB** was synthesized according to the literature.[2]

*Table S 2: Absorption maxima of the compounds **2-5** (for each photoisomer separately).*

λ_{max} (nm)	**2**	**3**	**4**	**5**	**8**
E-isomer	312, 452	318, 457	311, 466	314, 475	376
Z-isomer	286, 418	292, 417	275, 421	277, 424	304, 433

Photostability - Switching cycles of compound 8

The photostability of compound 8 was determined by cycling a 1 mM solution in DMSO between the PSS at wavelengths 470 nm and 660 nm (Figure S 2). In the experiment the solution was irradiated for 20 min with light at the respective wavelength, followed by an analytical HPLC measurement. The chromatograms of the initial dark state and the last cycle at 470 nm are shown in Figure S 3 to proof that no degradation occurred.

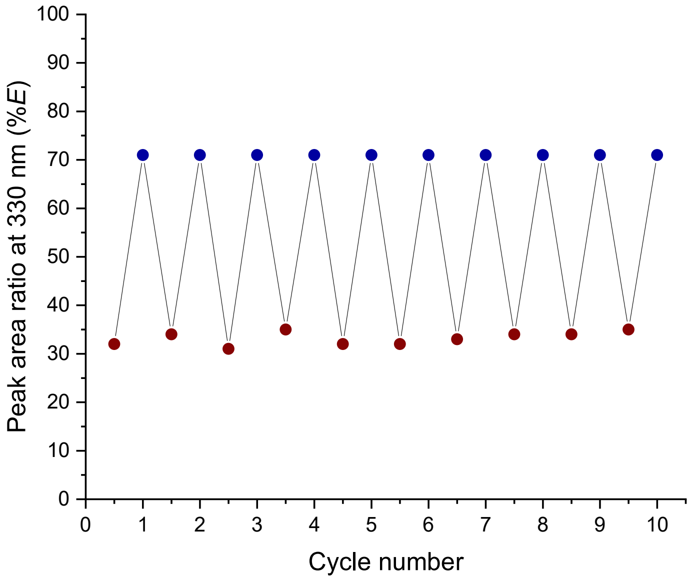

*Figure S 2: Switching cycles of compound **8**. Fluctuations after red light irradiation appear in consequence of the low thermal stability and heat generation of the 660 nm LED.*

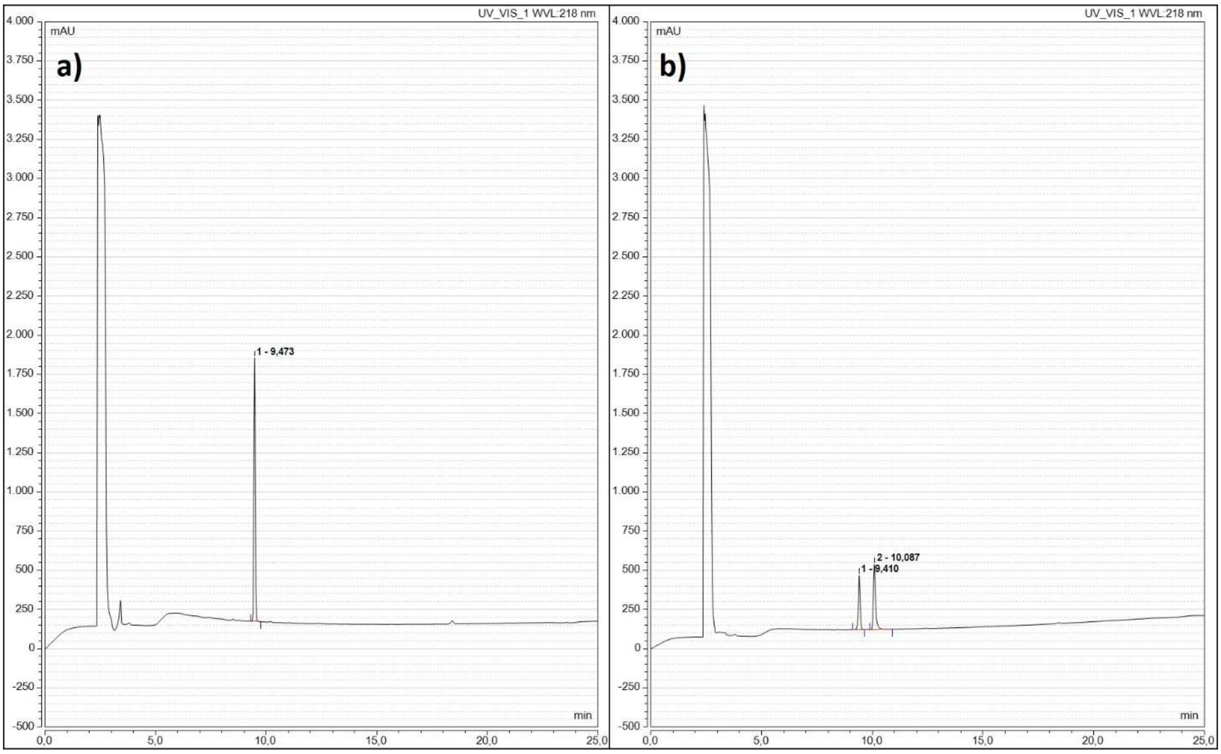

*Figure S 3: Chromatogram of the compound **8**. a) initial dark state before irradiation. b) last cycle after irradiation at 660 nm.*

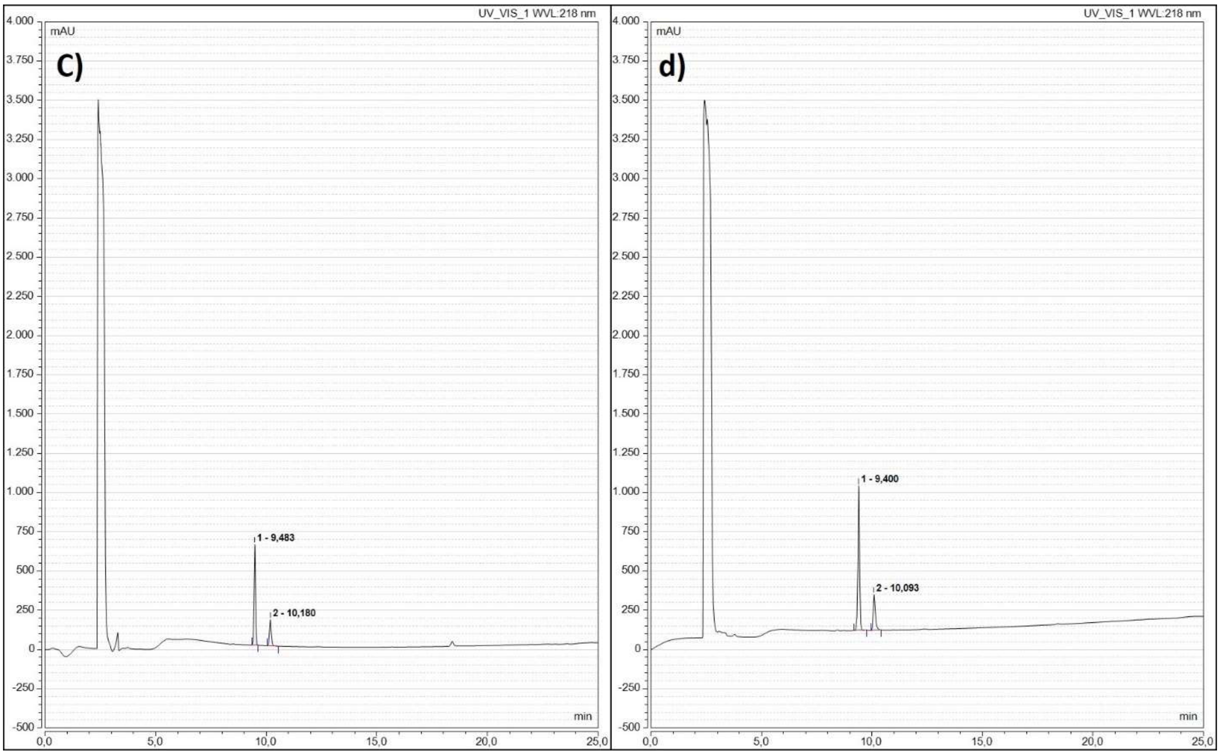

*Figure S 4: Chromatogram of the compound **8**. c) irradiation at 470 nm (cycle 1). d) irradiation at 470 nm (cycle 10).*

Thermal stability

The thermal stability was determined for compound **4**, **5** and **8.** Samples were prepared in MeCN (**4**, **5**) or DMSO (**8**) and irradiated at 623 nm to yield a high PSS. The solution was kept in MeCN at 60 °C (**4**, **5**) or in DMSO at 20 °C (**8**) and the isomer ratio was determined by HPLC in intervals. The obtained data was processed by calculating the ln(X_0/X_t), where X is the percentage of the respective *Z*-isomer and linear fitting (Equation (1)) of the obtained values. The calculated slope corresponds to the degradation rate constant *k* which is used to calculate the half-life $t_{1/2}$.

(1) $$x_t = x_0 \cdot e^{-k \cdot t} \leftrightarrow ln\left(\frac{x_0}{x_t}\right) = k \cdot t$$

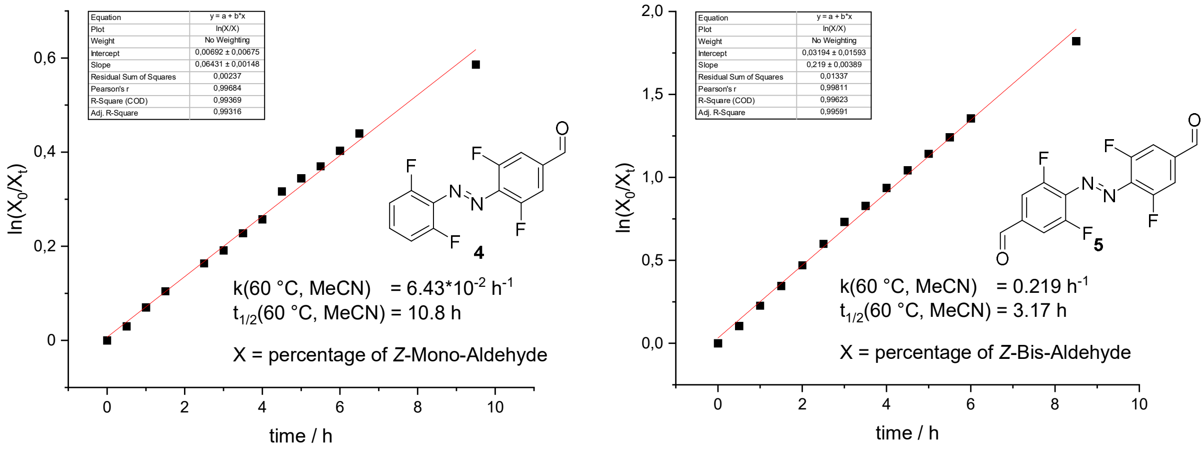

*Figure S 5: Linear fit of the decay of the Z-isomer of compound **4** and **5** at 60 °C in MeCN for first-order kinetics.*

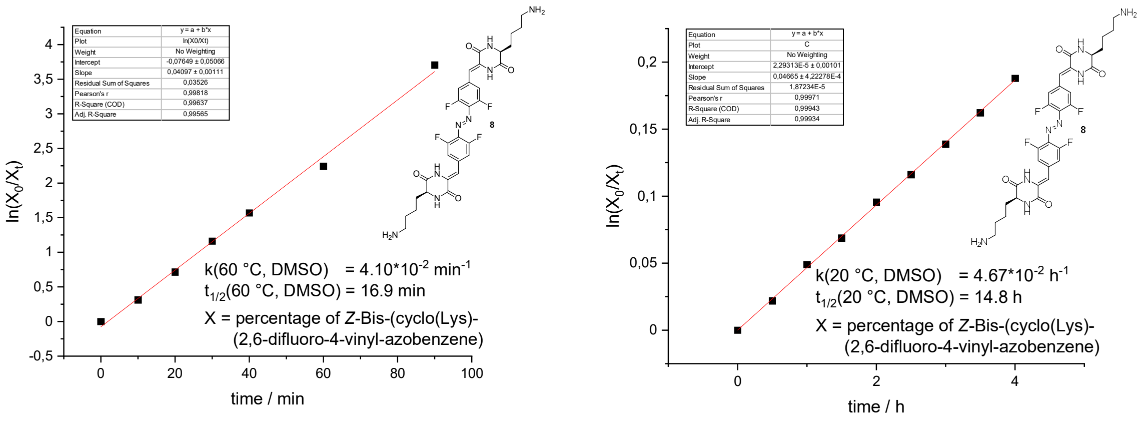

*Figure S 6: Linear fit of the decay of the Z-isomer of compound **8** at 60 °C and 20 °C in DMSO for first-order kinetics*

4. Computations

All calculations were performed with the GAUSSIAN09 program package,[11] employing the PBE0-D3/def2-TZVP level of theory.[12-14] This level performed well in studies on basic properties for similar systems.[21-22] We optimized geometries of *Z*- and *E*-isomers and performed vibrational frequency calculations to check the minimum nature of these species.

Cartesian Coordinates of the optimized structures

The presented data were obtained and visualized with Avogadro 1.2.0.

***E*-TFAB**

N	-0.40303	0.47486	0.07373
F	-2.19399	2.14180	1.11476
F	-1.50313	-1.92989	-1.12662
C	-4.52136	-0.32958	-0.03858
C	-4.02976	0.82755	0.54456
C	-2.66676	1.03248	0.55899
C	-1.75235	0.11016	0.03635
C	-2.29975	-1.04079	-0.54873
C	-3.66101	-1.26273	-0.59553
N	0.40306	-0.47496	0.07389
F	2.19418	-2.14182	1.11480
F	1.50295	1.92984	-1.12655
C	4.52133	0.32969	-0.03869
C	4.02984	-0.82747	0.54449
C	2.66685	-1.03248	0.55900
C	1.75236	-0.11023	0.03639
C	2.29966	1.04076	-0.54873
C	3.66090	1.26279	-0.59559
H	-5.58992	-0.50533	-0.06376
H	-4.67982	1.57253	0.98423
H	-4.02433	-2.16393	-1.07250
H	5.58988	0.50550	-0.06393
H	4.67997	-1.57241	0.98412
H	4.02414	2.16401	-1.07258

Zero-point correction =	0.158694 (Hartree/Particle)
Sum of electronic and zero-point Energies =	-968.881266
Sum of electronic and thermal Energies =	-968.867009
Sum of electronic and thermal Enthalpies =	-968.866065
Sum of electronic and thermal Free Energies =	-968.924727

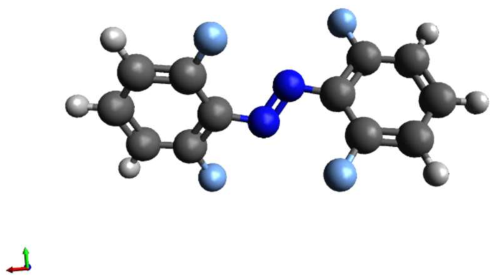

***Z*-TFAB**

N	-0.61057	0.08454	-1.80342
F	-2.74104	-1.48848	-1.19685
F	-0.05577	2.13152	0.04833
C	-3.02429	0.78952	1.56990
C	-3.31005	-0.26214	0.71163
C	-2.47803	-0.48710	-0.36394
C	-1.34005	0.28005	-0.60120
C	-1.09887	1.33270	0.27817
C	-1.92103	1.59942	1.35351
N	0.61060	-0.08435	-1.80342
F	0.05567	-2.13141	0.04819
F	2.74115	1.48852	-1.19672
C	3.02426	-0.78970	1.56987
C	1.92096	-1.59952	1.35342
C	1.09882	-1.33269	0.27809
C	1.34007	-0.27999	-0.60121
C	2.47808	0.48710	-0.36388
C	3.31008	0.26201	0.71168
H	-3.67346	0.98309	2.41482
H	-4.16568	-0.90816	0.85937
H	-1.68616	2.43633	1.99838
H	3.67341	-0.98336	2.41479
H	1.68603	-2.43646	1.99823
H	4.16574	0.90798	0.85947

Zero-point correction =	0.158776 (Hartree/Particle)
Sum of electronic and zero-point Energies =	-968.871519
Sum of electronic and thermal Energies =	-968.857481
Sum of electronic and thermal Enthalpies =	-968.856537
Sum of electronic and thermal Free Energies =	-968.913562

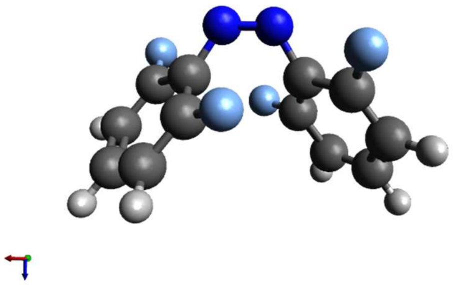

***E*-2 (**F4-alcohol)

N	-1.13644	-0.48726	-0.03237
F	-2.93264	-2.18836	-1.01047
F	-2.22924	1.98375	1.03540
C	-5.24977	0.35818	-0.00856
C	-4.76183	-0.83086	-0.52712
C	-3.40060	-1.04772	-0.51572
C	-2.48258	-0.10938	-0.02936
C	-3.02669	1.07354	0.49153
C	-4.38642	1.30982	0.51090
N	-0.32387	0.45770	-0.07571
F	1.47524	2.20852	-0.92959
F	0.79129	-2.03104	0.98555
C	3.81258	-0.31066	0.08630
C	3.30841	0.87739	-0.41744
C	1.94245	1.06405	-0.44666
C	1.02306	0.10131	-0.01395
C	1.58030	-1.08430	0.49575
C	2.93940	-1.28909	0.55294
C	5.28890	-0.56929	0.13912
O	5.99539	0.56109	-0.30626
H	-6.31684	0.54437	-0.00465
H	-5.41342	-1.59160	-0.93659
H	-4.74604	2.23708	0.93825
H	3.96589	1.65419	-0.78163
H	3.30228	-2.22084	0.97188
H	5.56055	-0.82941	1.17264
H	5.50846	-1.44850	-0.48452
H	6.93295	0.35918	-0.30316

Zero-point correction = 0.191118 (Hartree/Particle)
Sum of electronic and zero-point Energies = -1083.302660

Sum of electronic and thermal Energies =	-1083.285377
Sum of electronic and thermal Enthalpies =	-1083.284432
Sum of electronic and thermal Free Energies =	-1083.350858

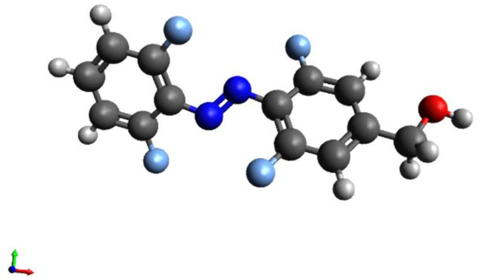

***Z*-2 (**F4-alcohol)

N	-1.67276	1.44171	-0.97286
F	-3.25425	-0.59762	-1.81372
F	-0.90100	1.33477	1.73579
C	-3.03876	-1.55537	1.64440
C	-3.40569	-1.57188	0.30687
C	-2.90937	-0.59598	-0.53024
C	-2.02543	0.38675	-0.09153
C	-1.69403	0.37253	1.26101
C	-2.18685	-0.57740	2.13199
N	-0.49845	1.76283	-1.17109
F	-0.14284	-0.99150	-1.70624
F	1.47236	2.91329	0.29750
C	2.92847	-0.37212	0.05914
C	1.93465	-1.03440	-0.64340
C	0.78887	-0.35139	-0.99752
C	0.58488	0.98859	-0.68397
C	1.62315	1.62812	-0.00806
C	2.76689	0.97265	0.38147
C	4.19336	-1.07259	0.46043
O	4.09406	-2.44710	0.18055
H	-3.42611	-2.31157	2.31592
H	-4.06894	-2.32531	-0.09777
H	-1.89854	-0.53298	3.17427
H	2.04397	-2.07462	-0.91744
H	3.52046	1.52133	0.93450
H	5.03112	-0.61536	-0.08634
H	4.36922	-0.89506	1.53118
H	4.94532	-2.85697	0.34542

Zero-point correction =	0.191383 (Hartree/Particle)
Sum of electronic and zero-point Energies =	-1083.292684
Sum of electronic and thermal Energies =	-1083.275695
Sum of electronic and thermal Enthalpies =	-1083.274750
Sum of electronic and thermal Free Energies =	-1083.338994

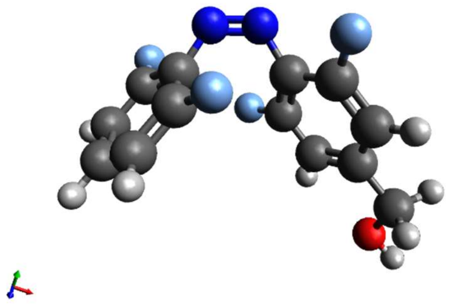

***E*-3 (**F4-bisalcohol)

N	-0.40661	-0.47333	-0.03045
F	-2.20689	-2.23810	-0.86091
F	-1.52107	2.04849	0.94583
C	-4.54190	0.32225	0.05529
C	-4.03822	-0.88209	-0.40887
C	-2.67309	-1.07706	-0.41583
C	-1.75274	-0.10833	-0.00004
C	-2.30939	1.09346	0.46984
C	-3.66817	1.30754	0.50535
N	0.40660	0.47329	-0.03045
F	2.20685	2.23808	-0.86091
F	1.52112	-2.04853	0.94584
C	4.54191	-0.32223	0.05528
C	4.03820	0.88210	-0.40888
C	2.67306	1.07705	-0.41584
C	1.75274	0.10830	-0.00004
C	2.30942	-1.09349	0.46984
C	3.66820	-1.30754	0.50534
C	6.01712	-0.59158	0.08031
O	6.72650	0.55401	-0.32078
C	-6.01711	0.59162	0.08034
O	-6.72652	-0.55393	-0.32081
H	-4.69550	-1.66537	-0.75934
H	-4.02997	2.25230	0.89520
H	4.69547	1.66540	-0.75935
H	4.03001	-2.25229	0.89518

H	6.29892	-0.90035	1.09753
H	6.22491	-1.44217	-0.58565
H	7.66148	0.34241	-0.34880
H	-6.29891	0.90033	1.09760
H	-6.22489	1.44226	-0.58556
H	-7.66148	-0.34229	-0.34888

Zero-point correction =	0.223545 (Hartree/Particle)
Sum of electronic and zero-point Energies =	-1197.723889
Sum of electronic and thermal Energies =	-1197.703562
Sum of electronic and thermal Enthalpies =	-1197.702618
Sum of electronic and thermal Free Energies =	-1197.776885

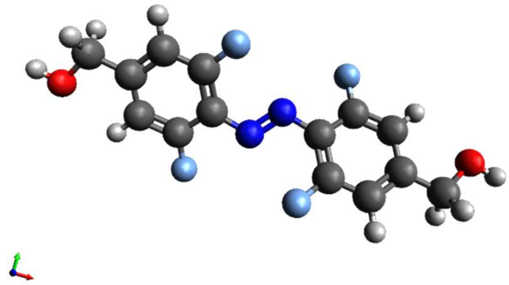

***Z*-3 (**F4-bisalcohol)

N	-0.55635	-2.37581	0.47117
F	-2.48458	-1.39235	2.10998
F	-0.27000	-1.01707	-1.99215
C	-3.04642	0.84408	-0.64875
C	-3.19325	0.22788	0.58525
C	-2.34087	-0.79955	0.92895
C	-1.30367	-1.22736	0.10616
C	-1.19893	-0.59456	-1.13142
C	-2.04739	0.41792	-1.51733
N	0.67780	-2.38095	0.46181
F	0.41414	-0.05963	2.05233
F	2.59400	-2.24634	-1.46043
C	3.18929	0.98378	0.01700
C	2.19689	1.00146	0.98389
C	1.34090	-0.07562	1.09289
C	1.43202	-1.19500	0.27171
C	2.46538	-1.18928	-0.66367
C	3.32060	-0.12352	-0.81631
C	4.15010	2.12737	-0.12808

O	3.71653	3.22417	0.63908
C	-3.94227	1.97506	-1.06198
O	-4.98836	2.12552	-0.13428
H	-3.96603	0.53851	1.27456
H	-1.91874	0.85755	-2.49975
H	2.08441	1.84464	1.65135
H	4.08033	-0.16957	-1.58796
H	5.14496	1.78705	0.19490
H	4.22715	2.39110	-1.19255
H	4.41245	3.88390	0.65220
H	-3.33625	2.89080	-1.12809
H	-4.32927	1.76801	-2.06999
H	-5.49077	2.91022	-0.36141

Zero-point correction =	0.223848 (Hartree/Particle)
Sum of electronic and zero-point Energies =	-1197.713428
Sum of electronic and thermal Energies =	-1197.693429
Sum of electronic and thermal Enthalpies =	-1197.692485
Sum of electronic and thermal Free Energies =	-1197.764435

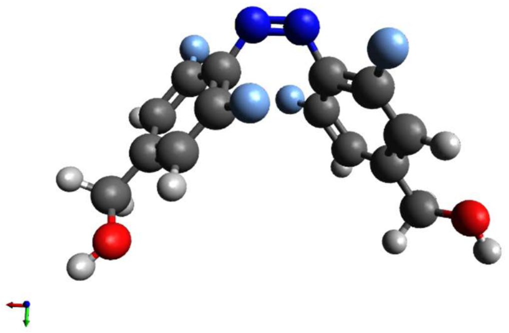

***E*-4 (**F4-aldehyde)

N	-0.99731	-0.39255	-0.15655
F	-2.66625	-2.16392	-1.20242
F	-2.26186	1.86974	1.17725
C	-5.15861	0.04616	0.11680
C	-4.58839	-1.04066	-0.52658
C	-3.21410	-1.12378	-0.58945
C	-2.36643	-0.14459	-0.05305
C	-2.99380	0.93304	0.59301
C	-4.36641	1.03150	0.68563
O	5.94955	-1.09042	0.38836
N	-0.25508	0.60111	-0.03646

F	1.41368	2.38492	-1.11120
F	0.98023	-1.74967	1.06987
C	5.36484	-0.14317	-0.06644
C	3.89541	0.01232	-0.05718
C	3.32957	1.15591	-0.60784
C	1.95982	1.29451	-0.58752
C	1.11308	0.31475	-0.06066
C	1.72915	-0.82080	0.49266
C	3.09492	-0.97634	0.50528
H	-6.23708	0.12569	0.17998
H	-5.18555	-1.82303	-0.97604
H	-4.79246	1.87925	1.20656
H	5.91489	0.69968	-0.53580
H	3.93989	1.93579	-1.04832
H	3.54403	-1.85656	0.94831

Zero-point correction =	0.167878 (Hartree/Particle)
Sum of electronic and zero-point Energies =	-1082.120131
Sum of electronic and thermal Energies =	-1082.103801
Sum of electronic and thermal Enthalpies =	-1082.102857
Sum of electronic and thermal Free Energies =	-1082.166360

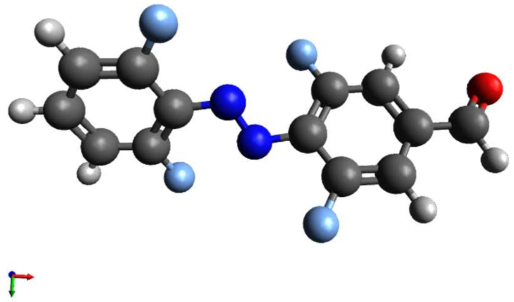

***Z*-4 (**F4-aldehyde)

N	-1.55473	1.47024	-0.94634
F	-3.39142	-0.37187	-1.69772
F	-0.66175	1.27488	1.72344
C	-3.09140	-1.37498	1.74087
C	-3.52997	-1.34330	0.42511
C	-2.98065	-0.41826	-0.43587
C	-1.97325	0.46074	-0.04240
C	-1.57302	0.40274	1.29081
C	-2.11649	-0.49609	2.18502

O	5.01376	-1.21370	0.82515
N	-0.36367	1.67054	-1.19010
F	-0.27869	-1.06492	-1.81151
F	1.70926	2.61253	0.28002
C	4.05159	-1.60240	0.21764
C	2.88387	-0.76258	-0.11848
C	1.82334	-1.32351	-0.81882
C	0.73010	-0.53972	-1.11768
C	0.65928	0.79957	-0.74316
C	1.76027	1.33351	-0.06771
C	2.85631	0.57648	0.26287
H	-3.52054	-2.09084	2.43094
H	-4.29056	-2.01923	0.05659
H	-1.77234	-0.48872	3.21111
H	3.98249	-2.65237	-0.13752
H	1.83707	-2.36097	-1.13303
H	3.68629	1.00747	0.80857

Zero-point correction =	0.168066 (Hartree/Particle)
Sum of electronic and zero-point Energies =	-1082.110620
Sum of electronic and thermal Energies =	-1082.094537
Sum of electronic and thermal Enthalpies =	-1082.093593
Sum of electronic and thermal Free Energies =	-1082.155382

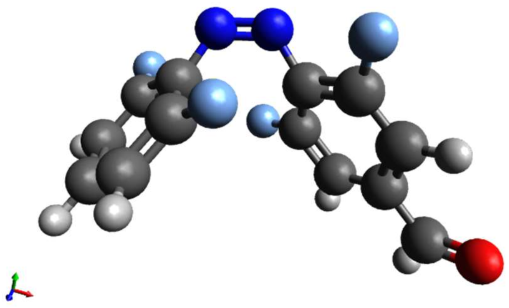

***E*-5 (**F4-bisaldehyde)

O	6.60295	-0.87770	0.57046
N	0.35521	0.51171	-0.16127
F	1.97939	2.29690	-1.27738
F	1.65294	-1.72114	1.13181
C	5.99762	0.01604	0.04197
C	4.52160	0.10587	-0.00534
C	3.92716	1.18849	-0.64144

C	2.55266	1.26580	-0.67313
C	1.73152	0.28025	-0.11408
C	2.37741	-0.79332	0.52570
C	3.74730	-0.88441	0.58989
O	-6.60295	0.87774	0.57046
N	-0.35522	-0.51174	-0.16125
F	-1.97940	-2.29691	-1.27737
F	-1.65292	1.72111	1.13183
C	-5.99762	-0.01601	0.04197
C	-4.52160	-0.10585	-0.00534
C	-3.92717	-1.18848	-0.64143
C	-2.55267	-1.26581	-0.67312
C	-1.73153	-0.28026	-0.11407
C	-2.37740	0.79331	0.52571
C	-3.74729	0.88441	0.58989
H	6.52801	0.85383	-0.45735
H	4.51798	1.96839	-1.10756
H	4.21972	-1.71563	1.09848
H	-6.52802	-0.85379	-0.45736
H	-4.51800	-1.96838	-1.10756
H	-4.21970	1.71564	1.09849

Zero-point correction =	0.176994 (Hartree/Particle)
Sum of electronic and zero-point Energies =	-1195.358134
Sum of electronic and thermal Energies =	-1195.339714
Sum of electronic and thermal Enthalpies =	-1195.338770
Sum of electronic and thermal Free Energies =	-1195.407224

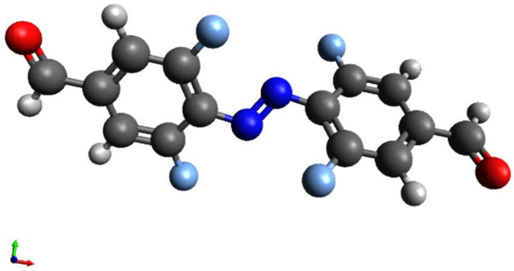

***Z*-5 (**F4-bisaldehyde)

O	-4.02043	3.18303	-0.63003
N	-0.65979	-2.30540	-0.42812
F	-2.53704	-2.12398	1.52020

F	-0.40158	-0.05883	-2.08941
C	-4.06104	2.21044	0.07518
C	-3.14639	1.05540	-0.05022
C	-3.27934	-0.01824	0.82317
C	-2.42550	-1.09086	0.69586
C	-1.41196	-1.11748	-0.25914
C	-1.32229	-0.02620	-1.12830
C	-2.16899	1.05168	-1.03993
O	4.85831	2.42063	0.30892
N	0.57191	-2.30409	-0.43575
F	0.31860	-0.96179	2.02051
F	2.44232	-1.32525	-2.12831
C	3.98621	1.98994	1.01489
C	3.07200	0.89335	0.62986
C	2.09654	0.47547	1.52608
C	1.23886	-0.53717	1.15536
C	1.32522	-1.15658	-0.08894
C	2.34237	-0.73005	-0.94818
C	3.19974	0.28959	-0.61829
H	-4.81154	2.12043	0.88829
H	-4.03748	-0.02786	1.59786
H	-2.08098	1.88468	-1.72631
H	3.81016	2.40410	2.02987
H	1.99584	0.92752	2.50624
H	3.96284	0.62115	-1.31128

Zero-point correction =	0.177111 (Hartree/Particle)
Sum of electronic and zero-point Energies =	-1195.348938
Sum of electronic and thermal Energies =	-1195.330732
Sum of electronic and thermal Enthalpies =	-1195.329787
Sum of electronic and thermal Free Energies =	-1195.396804

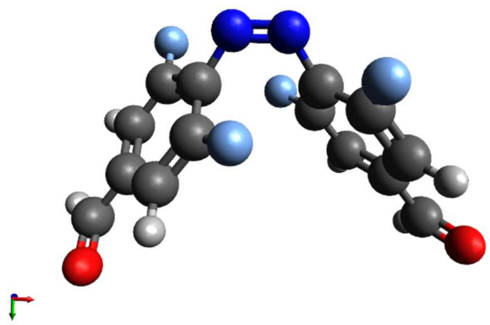

***E*-6 (**F3-alcohol)

N	-0.78814	-0.18173	-0.19948
F	-2.31938	-2.13108	-1.17303
F	-2.21887	1.94743	1.15956
C	-4.96607	-0.16415	0.23680
C	-4.31572	-1.19548	-0.42216
C	-2.94353	-1.14136	-0.54362
C	-2.17606	-0.07728	-0.05647
C	-2.87989	0.93699	0.60790
C	-4.25101	0.90033	0.76224
N	-0.18031	0.90430	-0.24741
F	1.50832	-1.40896	0.37463
C	3.98690	0.99171	-0.59777
C	3.20495	2.10240	-0.87859
C	1.83477	2.01881	-0.73537
C	1.21677	0.83055	-0.34141
C	2.03575	-0.26871	-0.05188
C	3.41659	-0.20192	-0.17376
C	4.25666	-1.39040	0.17813
O	4.43798	-1.41149	1.58304
H	-6.04331	-0.19212	0.34675
H	-4.84905	-2.03926	-0.83996
H	-4.73506	1.70704	1.29770
H	5.06604	1.04468	-0.69524
H	3.66493	3.02933	-1.19788
H	1.18914	2.86694	-0.93031
H	3.76008	-2.30444	-0.16668
H	5.22100	-1.31014	-0.34062
H	4.75035	-2.28368	1.83543

Zero-point correction =	0.199739 (Hartree/Particle)
Sum of electronic and zero-point Energies =	-984.103535
Sum of electronic and thermal Energies =	-984.087247
Sum of electronic and thermal Enthalpies =	-984.086303
Sum of electronic and thermal Free Energies =	-984.150047

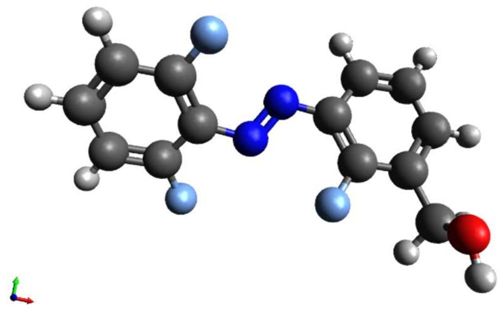

***Z*-6 (**F3-alcohol)

N	-1.29064	-0.81768	-1.72435
F	-2.35722	1.68523	-1.56640
F	-1.24901	-2.25998	0.68412
C	-2.91960	0.70466	1.84710
C	-2.92752	1.47315	0.69219
C	-2.35378	0.96075	-0.45186
C	-1.73594	-0.28617	-0.48318
C	-1.76712	-1.03036	0.69274
C	-2.34603	-0.55662	1.85234
N	-0.13581	-1.22048	-1.88007
F	0.58774	1.29475	-0.84325
C	2.97761	-0.75354	0.89226
C	2.64192	-2.01102	0.41014
C	1.60995	-2.14797	-0.49809
C	0.86986	-1.03802	-0.89247
C	1.24296	0.21276	-0.41400
C	2.29126	0.37962	0.47820
C	2.67003	1.75093	0.94391
O	3.48521	2.35101	-0.04609
H	-3.37305	1.09102	2.75151
H	-3.37153	2.45972	0.66140
H	-2.34296	-1.18373	2.73451
H	3.79862	-0.63862	1.59147
H	3.19581	-2.88387	0.73328
H	1.33779	-3.11531	-0.90291
H	1.76242	2.34154	1.11578
H	3.20668	1.66572	1.89791
H	3.53458	3.29357	0.13070

Zero-point correction = 0.199771 (Hartree/Particle)
Sum of electronic and zero-point Energies = -984.093382

Sum of electronic and thermal Energies =	-984.077278
Sum of electronic and thermal Enthalpies =	-984.076334
Sum of electronic and thermal Free Energies =	-984.138587

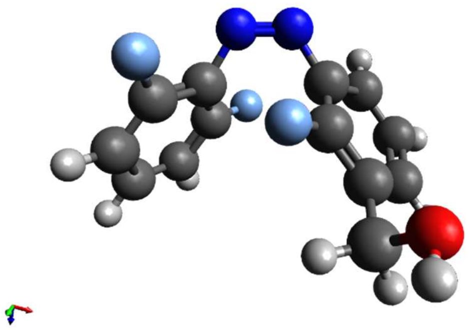

***E*-7 (**F3-aldehyde)

N	0.77302	-0.19810	0.12729
F	2.24008	-2.26648	0.90973
F	2.28131	2.07262	-0.89780
C	4.96300	-0.17683	-0.12290
C	4.27548	-1.27318	0.37263
C	2.89978	-1.21599	0.43783
C	2.16386	-0.08877	0.05043
C	2.90641	0.99157	-0.44996
C	4.28240	0.95490	-0.54412
N	0.15378	0.87942	0.20962
F	-1.47016	-1.38399	-0.69080
C	-4.03705	0.89316	0.40310
C	-3.28264	1.97568	0.80976
C	-1.90204	1.91625	0.71370
C	-1.24541	0.77949	0.24450
C	-2.03433	-0.30049	-0.17573
C	-3.42174	-0.25176	-0.10337
C	-4.24919	-1.39250	-0.55285
O	-5.45339	-1.39160	-0.50290
H	6.04395	-0.20542	-0.18653
H	4.78251	-2.16921	0.70563
H	4.79824	1.81490	-0.95158
H	-5.11986	0.89874	0.45296
H	-3.76260	2.86782	1.19213
H	-1.27889	2.75297	1.00775
H	-3.69614	-2.26485	-0.94376

Zero-point correction =	0.176277 (Hartree/Particle)
Sum of electronic and zero-point Energies =	-982.922560
Sum of electronic and thermal Energies =	-982.907063
Sum of electronic and thermal Enthalpies =	-982.906119
Sum of electronic and thermal Free Energies =	-982.967934

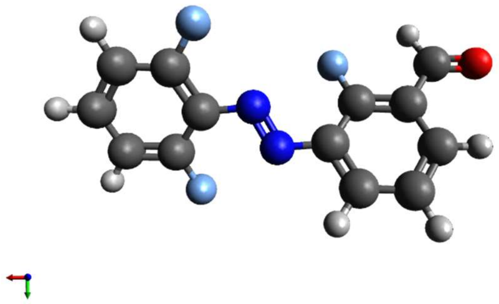

***Z*-7 (**F3-aldehyde)

N	-1.36847	-1.27144	-1.39370
F	-2.59443	1.12345	-1.79775
F	-0.97983	-1.98862	1.29453
C	-2.73429	1.11085	1.79540
C	-2.91613	1.52664	0.48447
C	-2.42798	0.73976	-0.53636
C	-1.72700	-0.43961	-0.29848
C	-1.58304	-0.82764	1.03073
C	-2.07387	-0.07430	2.07713
N	-0.20883	-1.65353	-1.56392
F	0.44576	1.07357	-1.34277
C	3.13316	-0.32687	0.61986
C	2.83126	-1.67279	0.53798
C	1.71877	-2.08897	-0.17694
C	0.87028	-1.16588	-0.77608
C	1.20594	0.18202	-0.70417
C	2.32877	0.61913	-0.01308
C	2.67036	2.05583	0.06006
O	3.62127	2.47641	0.66869
H	-3.11827	1.71576	2.60739
H	-3.42978	2.44739	0.24016
H	-1.93551	-0.42912	3.09021
H	4.00161	0.03215	1.15959
H	3.46620	-2.40632	1.01913
H	1.47304	-3.14037	-0.26954

H 1.99269 2.73937 -0.48235

Zero-point correction =	0.176385 (Hartree/Particle)
Sum of electronic and zero-point Energies =	-982.912119
Sum of electronic and thermal Energies =	-982.896855
Sum of electronic and thermal Enthalpies =	-982.895911
Sum of electronic and thermal Free Energies =	-982.955907

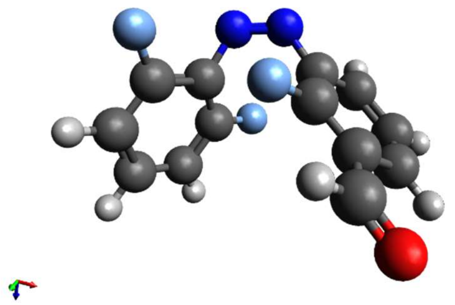

Molecular Orbitals

The orbital energies were obtained from Gaussian09, and the corresponding 3D representation of the electron probability distributions were visualized by Avogadro 1.2.0.

Table S3: *HOMO-1 (H-1), HOMO (H) and LUMO (L) Kohn-Sham orbital energies (in eV) of the* E*-isomers and* Z*-isomers of the compounds* ***2-7*** *(compared with TFAB) calculated on the PBE0-D3/def2-TZVP level of theory. In all cases, the HOMO is the n-orbital. The HOMO-LUMO gaps (ΔHL) were calculated independently for* Z*- and* E*-isomers.*

compound		$\varepsilon_{H-1}(Z)$	$\varepsilon_{H}(Z)$	$\varepsilon_{L}(Z)$	$\Delta_{HL}(Z)$	$\varepsilon_{H-1}(E)$	$\varepsilon_{H}(E)$	$\varepsilon_{L}(E)$	$\Delta_{HL}(E)$
TFAB		-7,507	-6.780	-2.333	4.447	-7,426	-6.602	-2.525	4.077
F4-alcohol	**2**	-7,373	-6.603	-2.208	4.395	-7,145	-6.449	-2.402	4.047
F4-bisalcohol	**3**	-7,260	-6.458	-2.105	4.353	-6,941	-6.299	-2.284	4.015
F4-aldehyde	**4**	-7,771	-7.080	-2.886	4.194	-7,730	-6.898	-3.060	3.838
F4-bisaldehyde	**5**	-8,082	-7.359	-3.277	4.082	-7,994	-7.163	-3.493	3.670
F3-alcohol	**6**	-7,380	-6.626	-2.165	4.461	-7,136	-6.493	-2.378	4.115
F3-aldehyde	**7**	-7,669	-6.937	-2.571	4.366	-7,436	-6.799	-2.722	4.077

In the **Tables S 4-S 17** below, we have depicted molecular orbitals of **TFAB** and compounds **2-7** (for both *E*- and *Z*-isomer). For all compounds, we have plotted LUMO+1, LUMO, HOMO and HOMO-1 orbitals. For individual compounds, we have also plotted the molecular orbitals, which contributed to the lowest excitation state of the given molecule (Isovalue: 0.03).

*Table S4: Molecular orbitals for the E-**TFAB**, obtained on PBE0-D3/def2-TZVP level of theory.*

LUMO+1	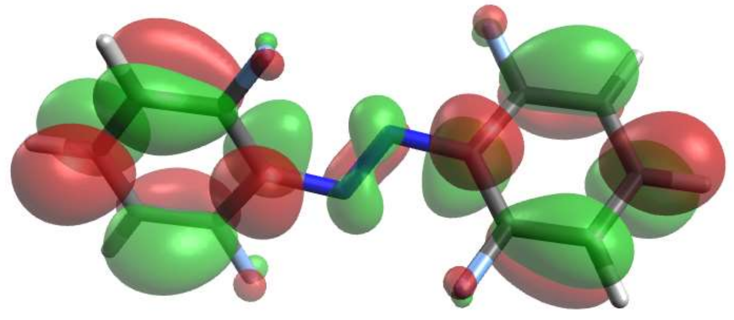
LUMO	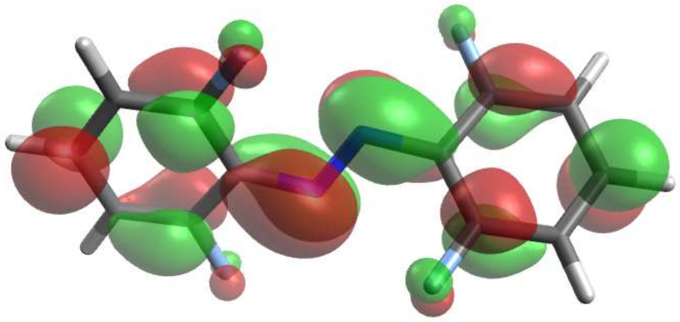
HOMO	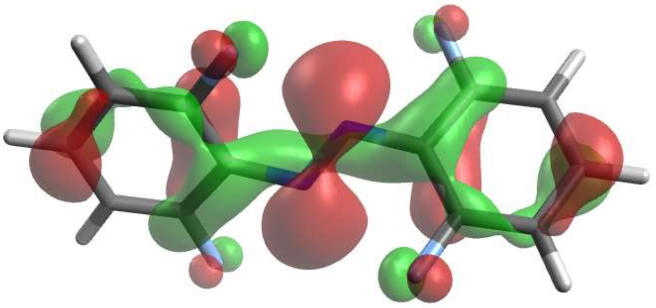

HOMO-1	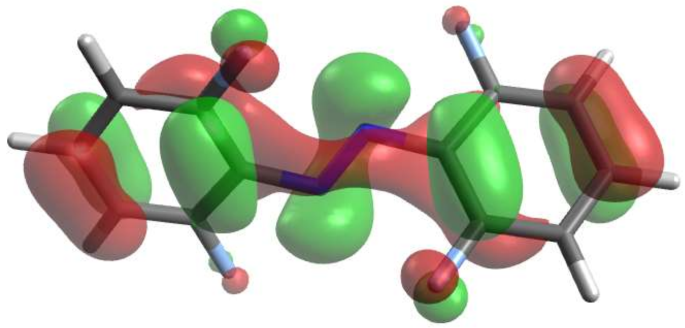
HOMO-3	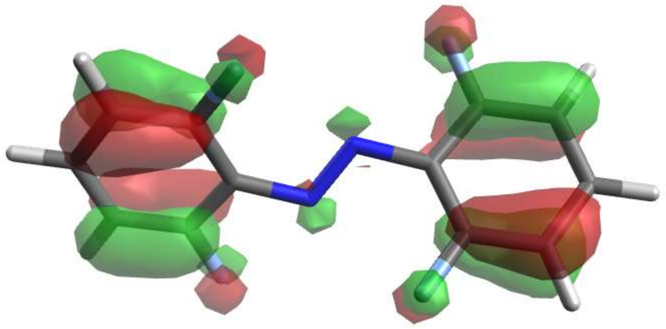

*Table S5: Molecular orbitals for the Z-**TFAB**, obtained on PBE0-D3/def2-TZVP level of theory.*

LUMO+1	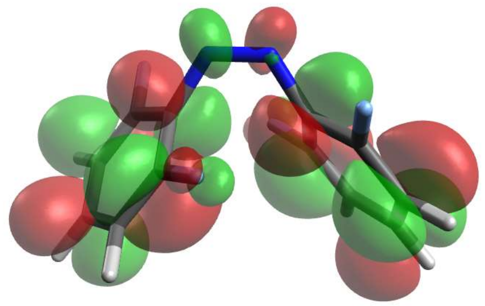
LUMO	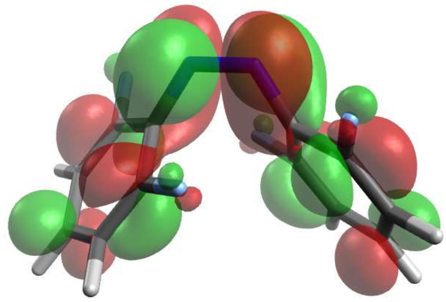

HOMO	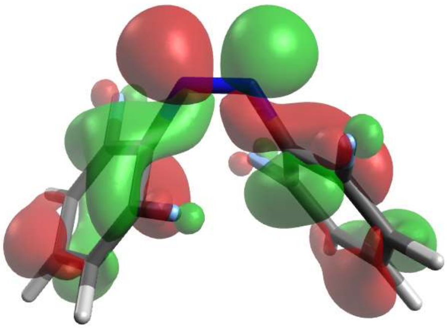
HOMO-1	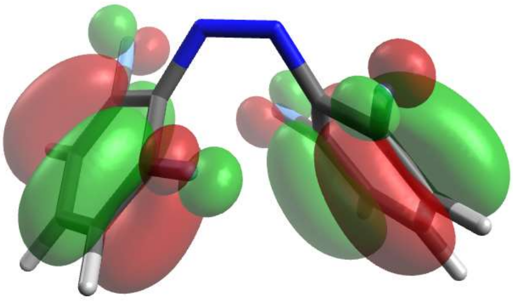
HOMO-4	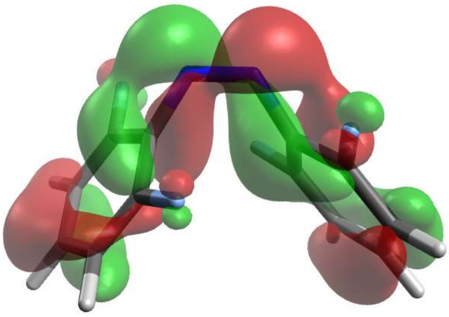

*Table S6: Molecular orbitals for the E-**2**, obtained on PBE0-D3/def2-TZVP level of theory.*

LUMO+1	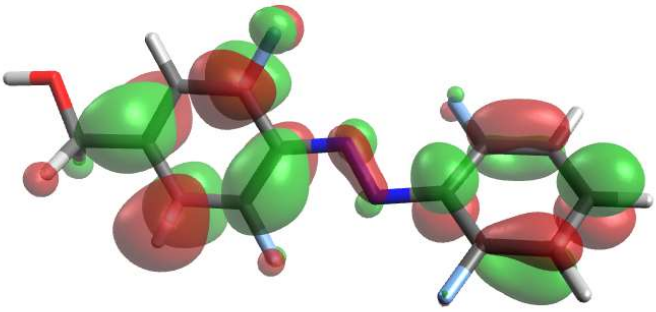

LUMO	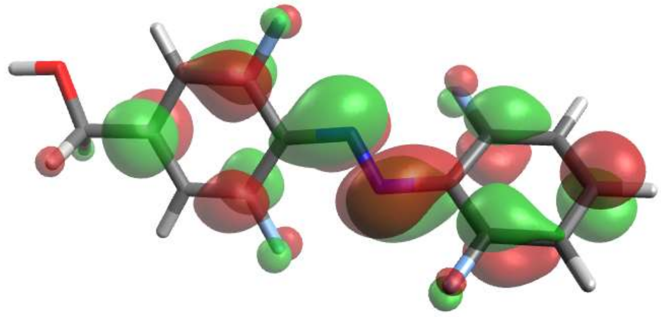
HOMO	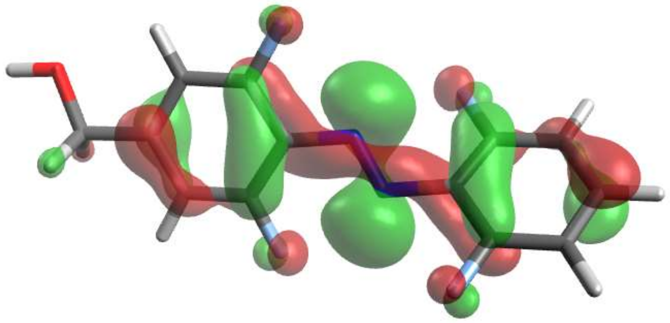
HOMO-1	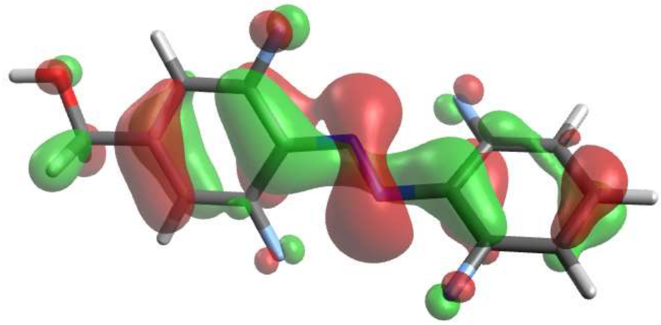

*Table S7: Molecular orbitals for the Z-**2**, obtained on PBE0-D3/def2-TZVP level of theory.*

LUMO+1	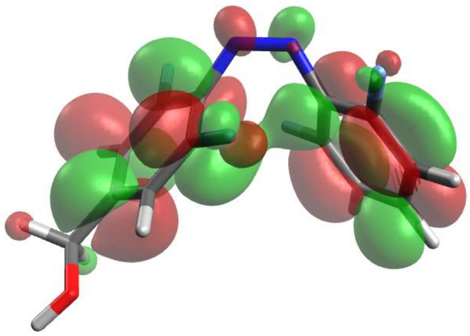

LUMO	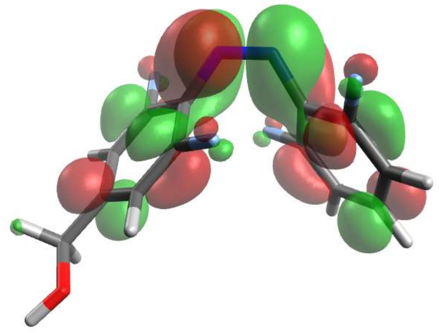
HOMO	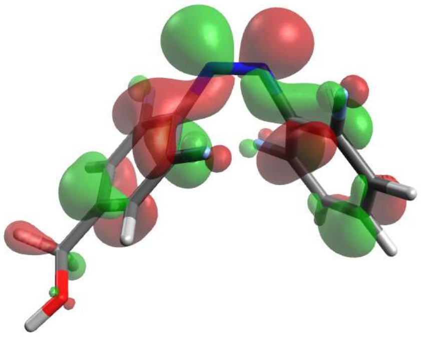
HOMO-1	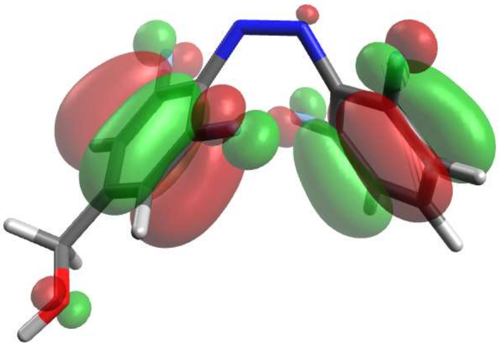
HOMO-3	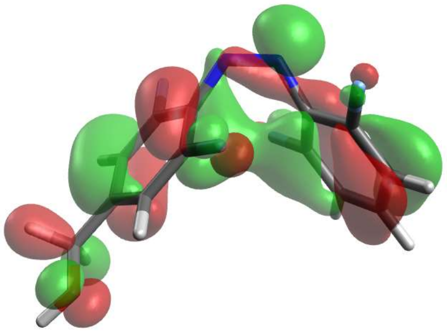

HOMO-4	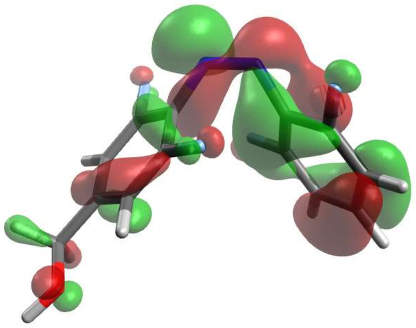

*Table S8: Molecular orbitals for the E-**3**, obtained on PBE0-D3/def2-TZVP level of theory.*

LUMO+1	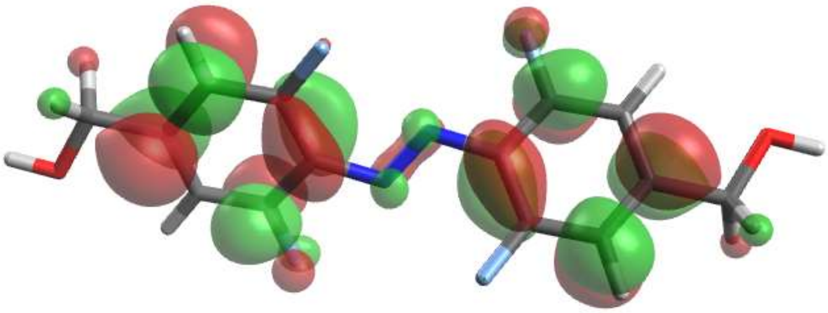
LUMO	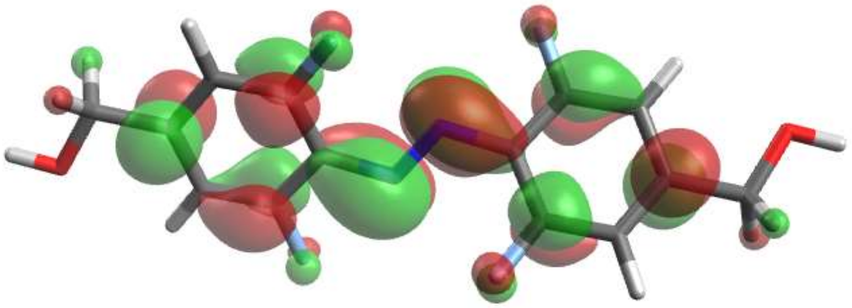
HOMO	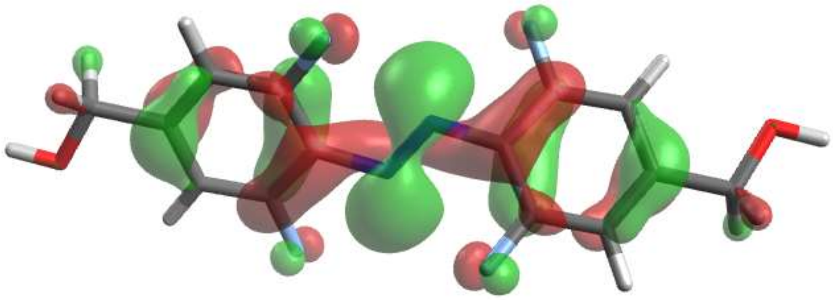

HOMO-1	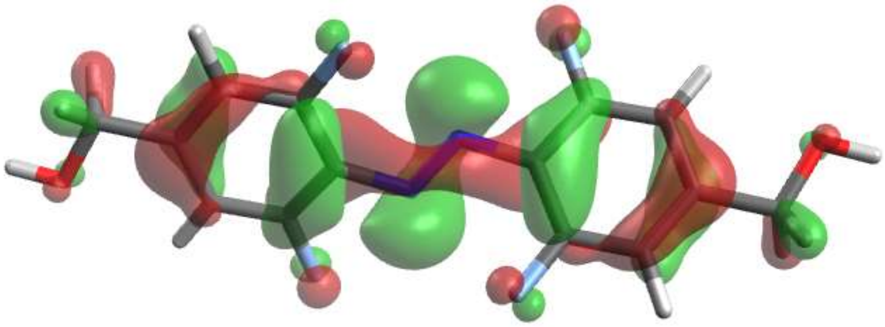

Table S9: Molecular orbitals for the Z-**3**, obtained on PBE0-D3/def2-TZVP level of theory.

LUMO+1	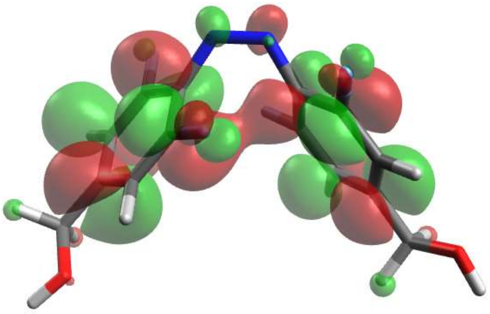
LUMO	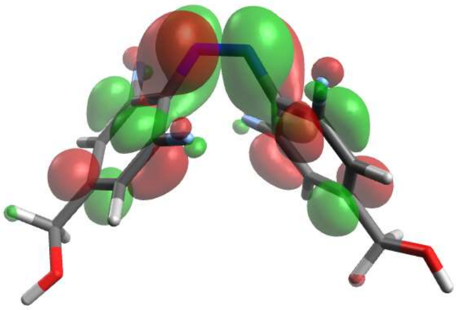
HOMO	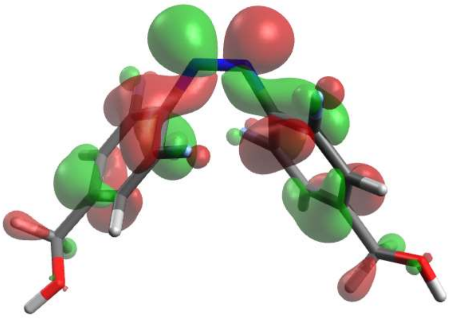

HOMO-1	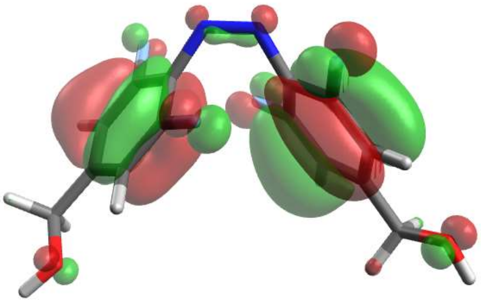
HOMO-4	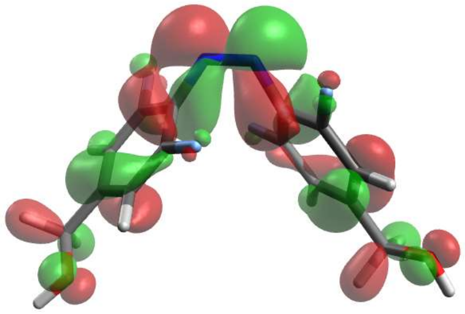

*Table S10: Molecular orbitals for the E-**4**, obtained on PBE0-D3/def2-TZVP level of theory.*

LUMO+1	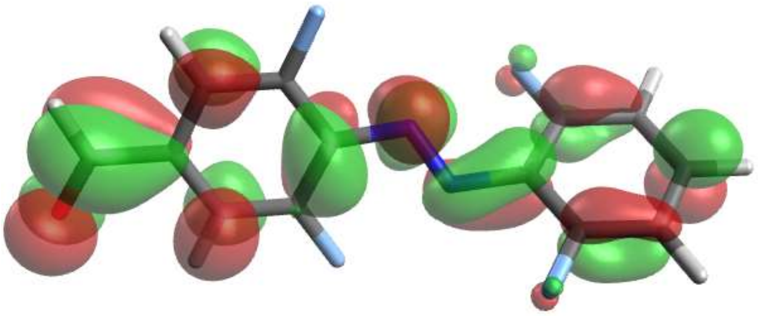
LUMO	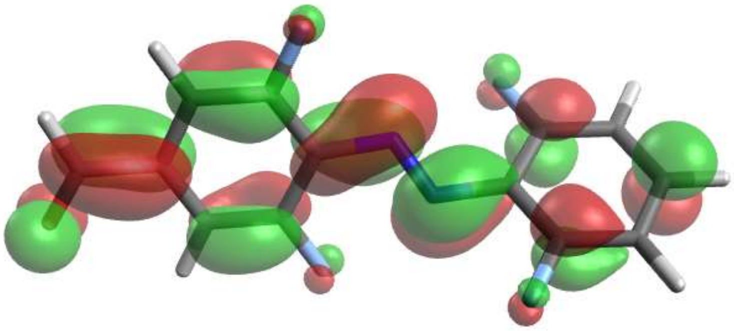

HOMO	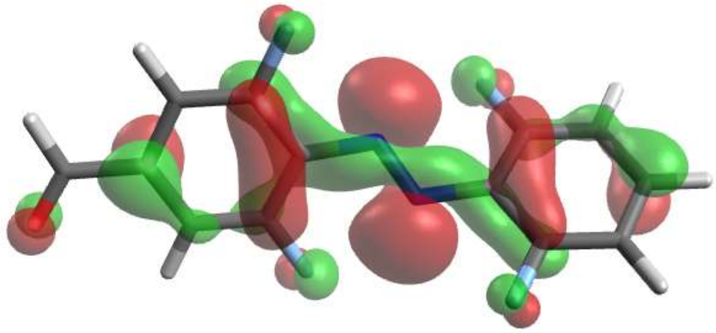
HOMO-1	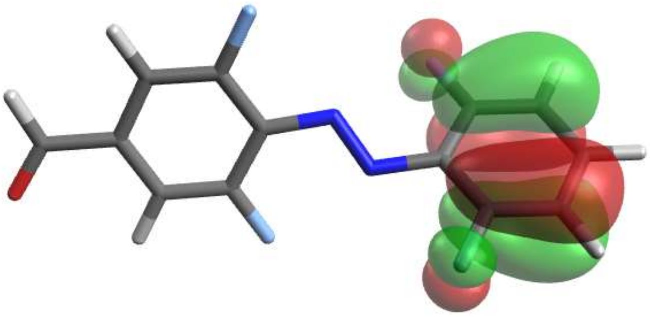
HOMO-2	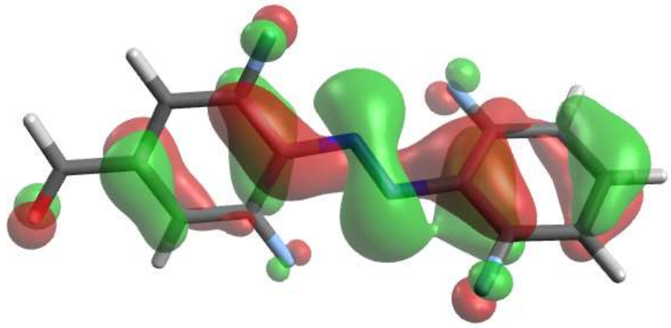

*Table S11: Molecular orbitals for the Z-**4**, obtained on PBE0-D3/def2-TZVP level of theory.*

LUMO+1	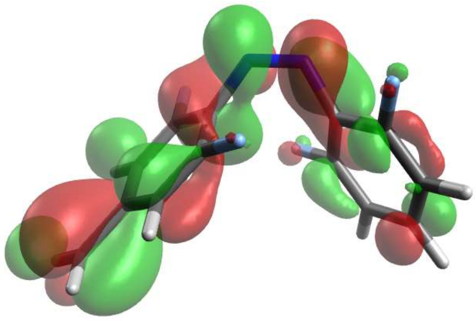

LUMO	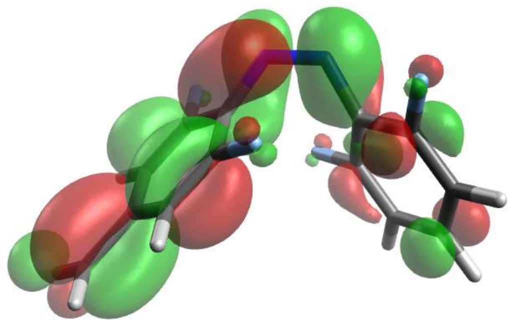
HOMO	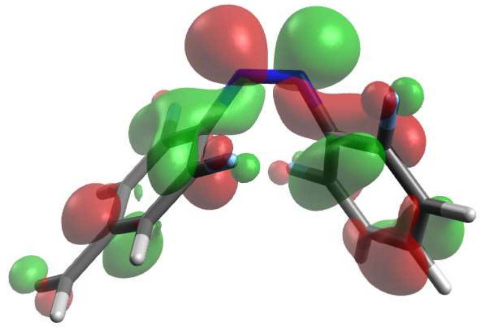
HOMO-1	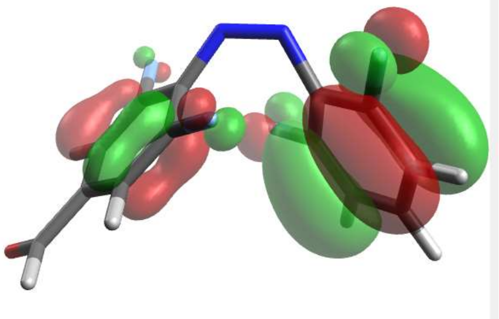
HOMO-5	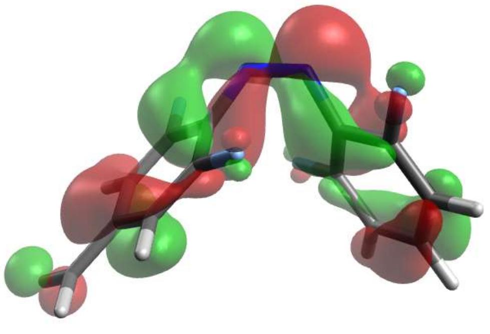

*Table S12: Molecular orbitals for the E-**5**, obtained on PBE0-D3/def2-TZVP level of theory.*

LUMO+1	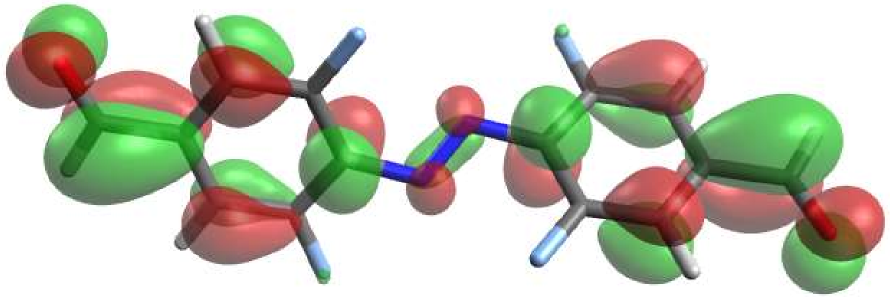
LUMO	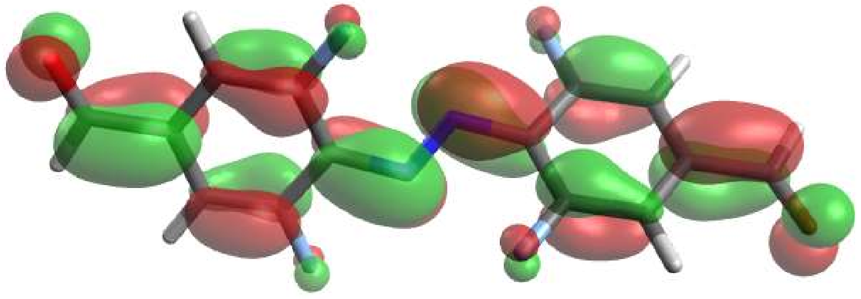
HOMO	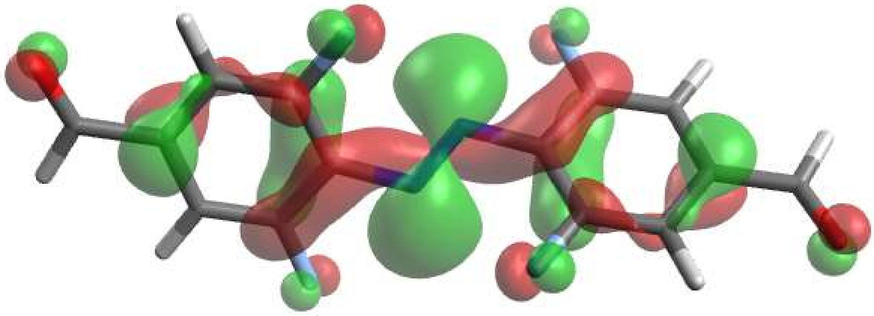
HOMO-1	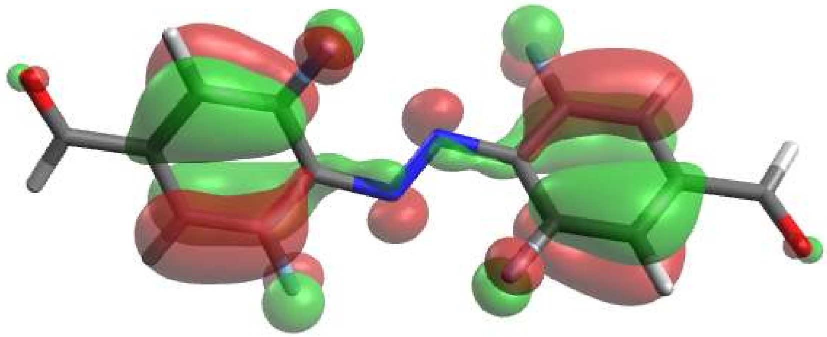

LUMO+2	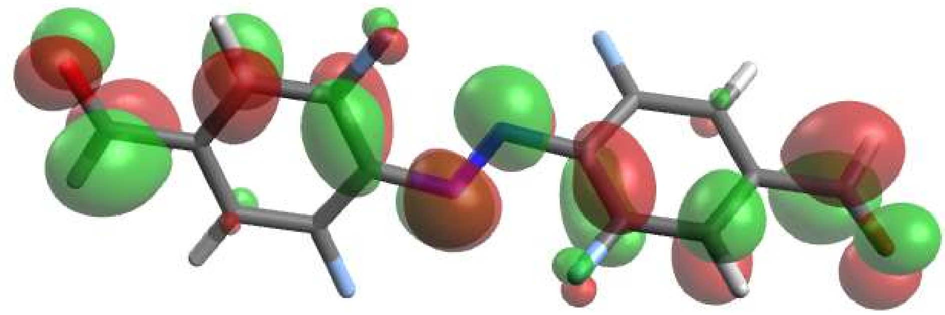
HOMO-3	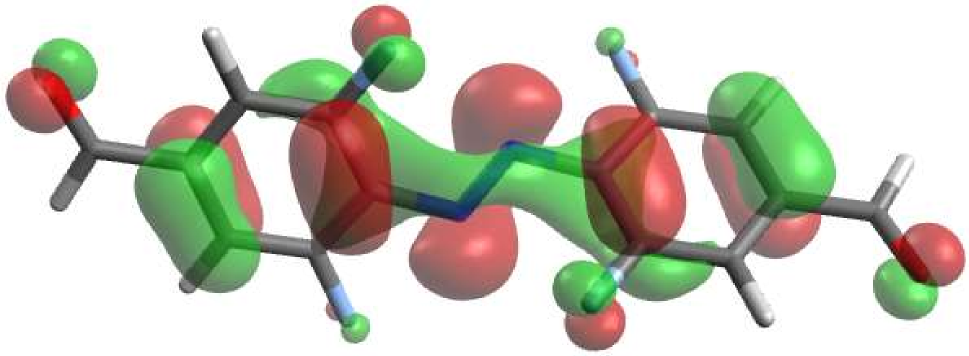

*Table S13: Molecular orbitals for the Z-**5**, obtained on PBE0-D3/def2-TZVP level of theory.*

LUMO+1	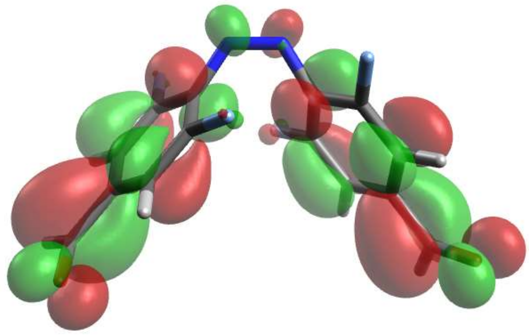
LUMO	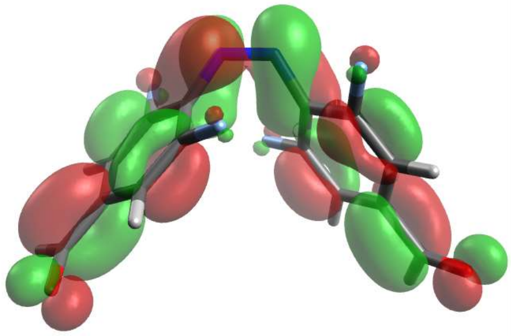

HOMO	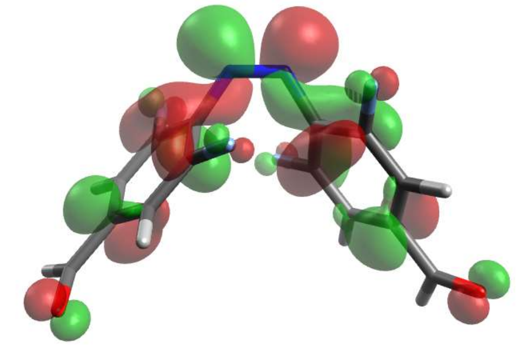
HOMO-1	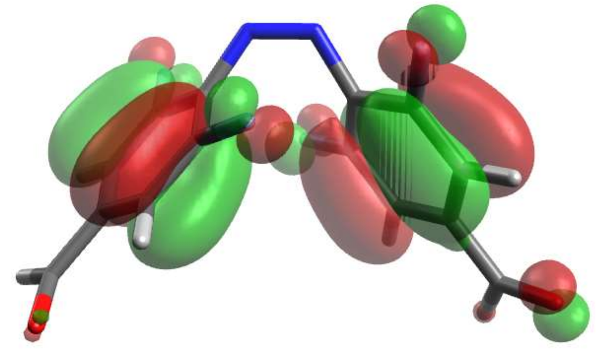
LUMO+2	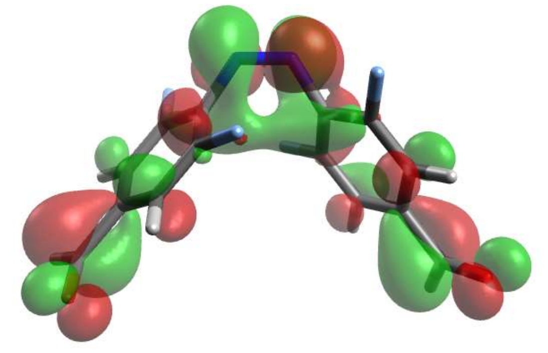
HOMO-6	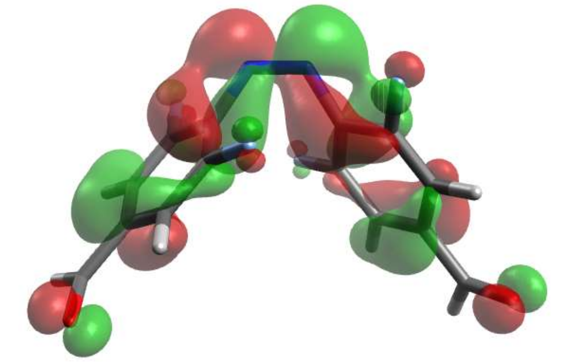

*Table S14: Molecular orbitals for the E-**6**, obtained on PBE0-D3/def2-TZVP level of theory.*

LUMO+1	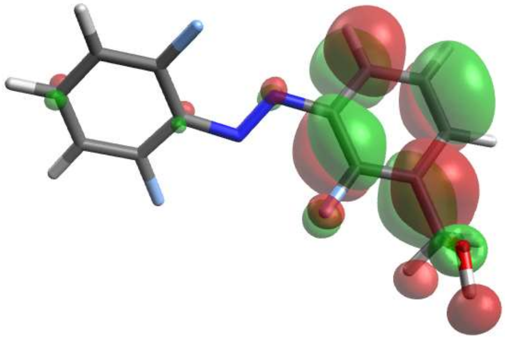
LUMO	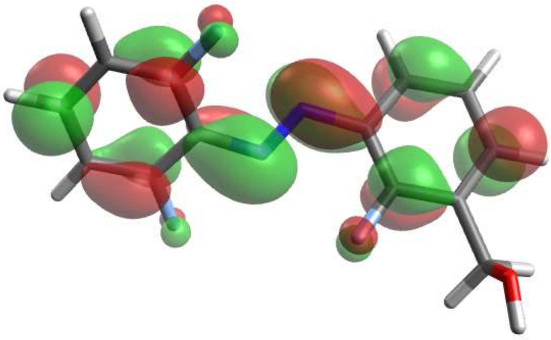
HOMO	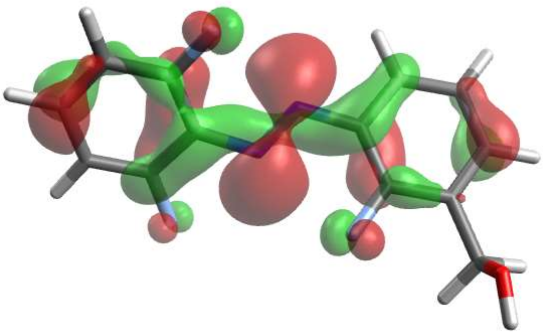
HOMO-1	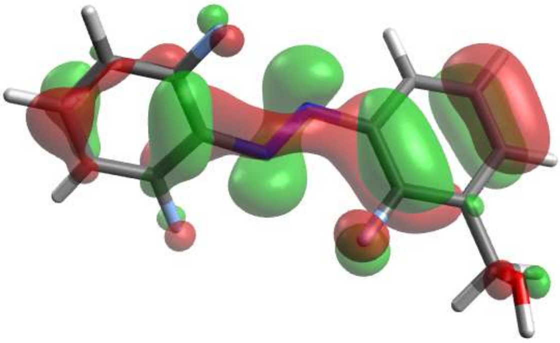

HOMO-3	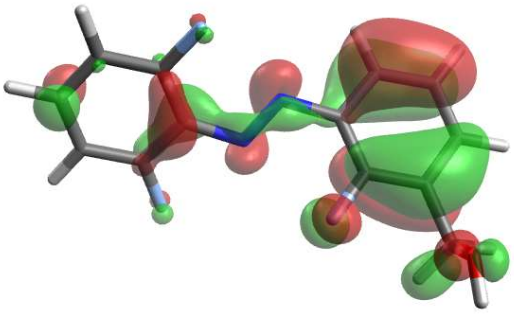

*Table S15: Molecular orbitals for the Z-**6**, obtained on PBE0-D3/def2-TZVP level of theory.*

LUMO+1	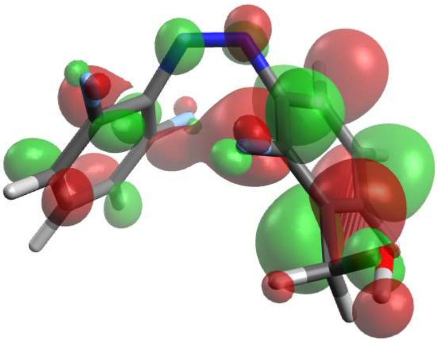
LUMO	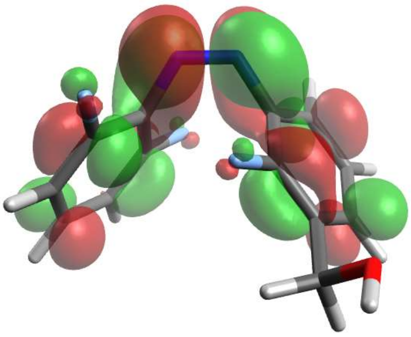
HOMO	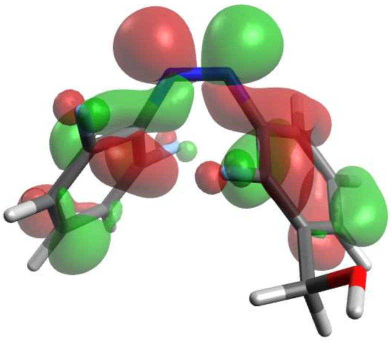

HOMO-1	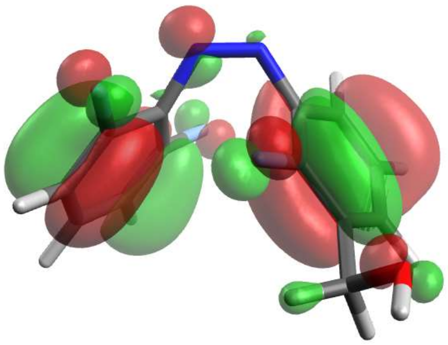
HOMO-4	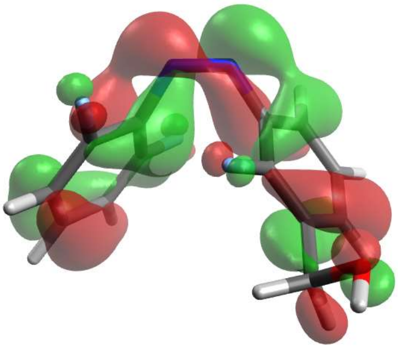
HOMO-5	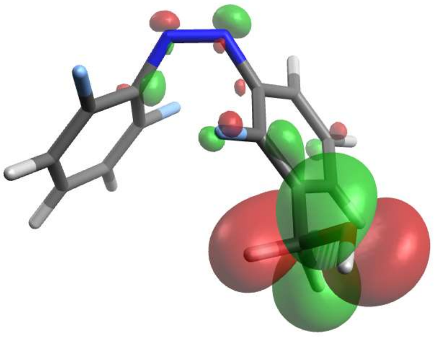

*Table S16: Molecular orbitals for the E-**7**, obtained on PBE0-D3/def2-TZVP level of theory.*

LUMO+1	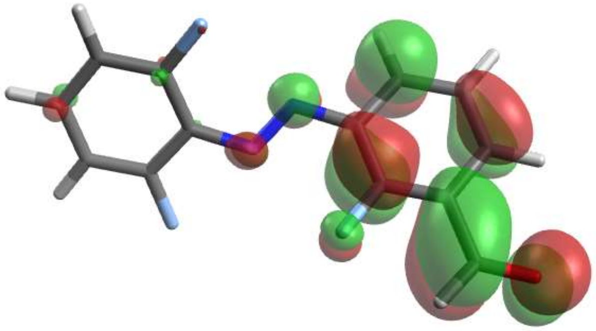

LUMO	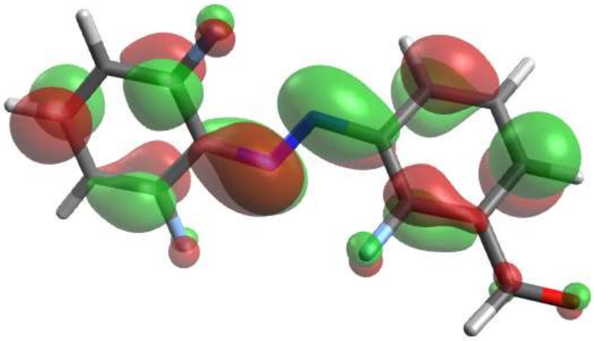
HOMO	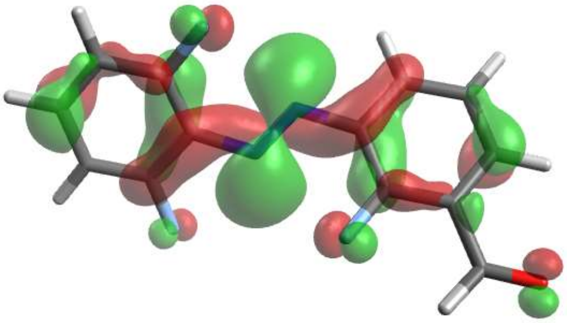
HOMO-1	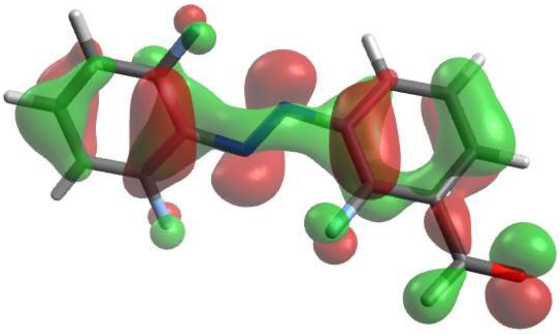

*Table S17: Molecular orbitals for the Z-**7**, obtained on PBE0-D3/def2-TZVP level of theory.*

LUMO+1	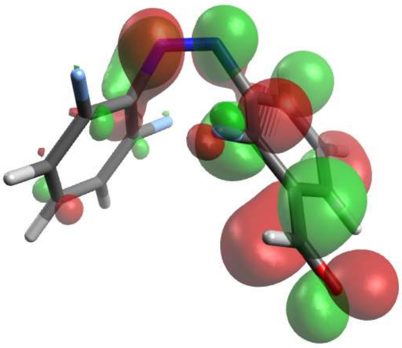

LUMO	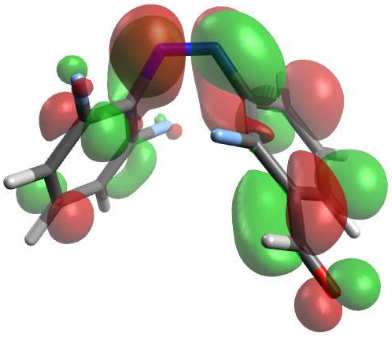
HOMO	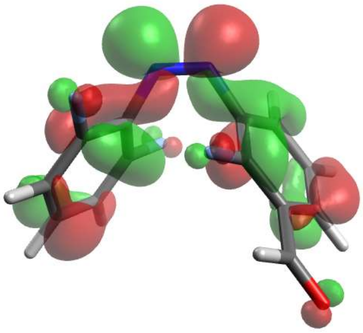
HOMO-1	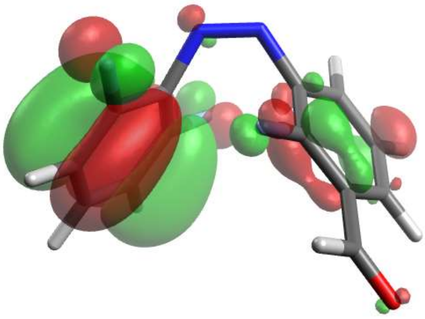
HOMO-5	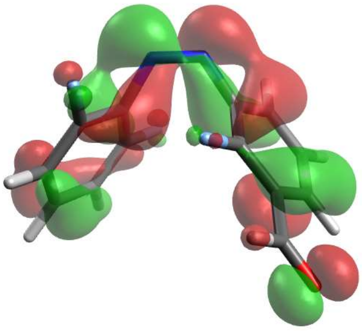

*Table S18: Calculated values of the N-N bond lengths and dihedral angles for **TFAB** and the molecules **2-7**.*

compound		N-N bond length [Å]	C–C–N–N dihedral angles Φ1	C–C–N–N dihedral angles Φ2	C–N–N–C dihedral angle Ψ
**TFAB**	*E*	1,246	29,4	29,5	176,6
	Z	1,233	59,7	59,7	8,9
**2**	*E*	1,247	29,8	21,7	177,0
	Z	1,233	59,4	58,0	9,0
**3**	*E*	1,248	23,4	23,4	177,2
	Z	1,234	58,4	58,0	9,1
**4**	*E*	1,246	23,4	36,5	176,2
	Z	1,232	58,1	60,8	9,2
**5**	*E*	1,246	31,8	31,8	175,7
	Z	1,232	59,6	59,8	9,2
**6**	*E*	1,245	30,9	17,7	177,8
	Z	1,233	62,1	58,0	7,7
**7**	*E*	1,246	26,7	19,9	178,1
	Z	1,233	61,6	58,7	7,6

Excitation Energies

Excitation energies were then calculated on TD-PBE0/def2-TZVP//PBE0-D3/def2-TZVP[23-26] level of theory, using previously obtained structures. Solvent interactions were simulated using a polarizable continuum model (PCM),[15] with parameters taken for MeCN. The five lowest transitions computed in the TD calculations (25 states in total) are reported. For each excited state the energy (in eV), the associated wavelength (in nm) and the oscillator strength of the transition is given. The numbers of the orbitals involved in the transition (with general formula occupied → unoccupied) and the associated (largest) coefficient in the CI expansion is printed.

E-TFAB

Excited State 1: 2.5530 eV 485.64 nm f=0.0628

61 -> 65 -0.30666

64 -> 65 0.62936

Excited State 2: 3.9384 eV 314.81 nm f=0.0092

62 -> 65 0.69595

Excited State 3: 3.9391 eV 314.75 nm f=0.0299

63 -> 65 0.69357

Excited State 4: 3.9715 eV 312.19 nm f=0.8651

61 -> 65 0.62930

64 -> 65 0.31278

Excited State 5: 4.8293 eV 256.73 nm f=0.0005

60 -> 65 0.66089

64 -> 66 -0.22123

***Z*-TFAB**

Excited State 1: 2.8466 eV 435.55 nm f=0.0429

60 -> 65 0.21774

64 -> 65 0.66605

Excited State 2: 3.9852 eV 311.11 nm f=0.0135

63 -> 65 0.69897

Excited State 3: 4.0877 eV 303.31 nm f=0.0032

61 -> 65 0.17805

62 -> 65 0.67488

Excited State 4: 4.2596 eV 291.07 nm f=0.0312

61 -> 65 0.65922

62 -> 65 -0.18617

64 -> 66 0.10647

Excited State 5: 4.3818 eV 282.95 nm f=0.1876

60 -> 65 0.66125

64 -> 65 -0.22114

***E*-2 (**F4-alcohol)

Excited State 1: 2.5510 eV 486.03 nm f=0.0656

71 -> 73 -0.35073

72 -> 73 0.59891

Excited State 2: 3.8237 eV 324.25 nm f=0.9371

71 -> 73 0.60071

72 -> 73 0.36186

Excited State 3: 3.9537 eV 313.59 nm f=0.0752

69 -> 73 0.27691

70 -> 73 0.63322

Excited State 4: 3.9660 eV 312.62 nm f=0.0209

69 -> 73 0.63430

70 -> 73 -0.28180

Excited State 5: 4.7316 eV 262.03 nm f=0.0035

68 -> 73 0.67153

72 -> 74 0.10251

72 -> 76 0.13245

***Z*-2 (**F4-alcohol)

Excited State 1: 2.8283 eV 438.37 nm f=0.0539

68 -> 73 0.20283

69 -> 73 0.12267

72 -> 73 0.65520

Excited State 2: 3.9762 eV 311.82 nm f=0.0177

71 -> 73 0.68406

Excited State 3: 4.0810 eV 303.81 nm f=0.0363

69 -> 73 0.37599

70 -> 73 0.56267

Excited State 4: 4.1634 eV 297.79 nm f=0.0635

67 -> 73 -0.11413

68 -> 73 0.10557

69 -> 73 0.52088

70 -> 73 -0.40980

72 -> 73 -0.12825

Excited State 5: 4.3395 eV 285.71 nm f=0.1513

68 -> 73 0.64066

69 -> 73 -0.15128

72 -> 73 -0.17963

***E*-3 (**F4-bisalcohol)

Excited State 1: 2.5528 eV 485.67 nm f=0.0739

79 -> 81 0.40582

80 -> 81 0.57064

Excited State 2: 3.7280 eV 332.57 nm f=1.0980

79 -> 81 0.57204

80 -> 81 -0.41107

Excited State 3: 3.9667 eV 312.56 nm f=0.0052

78 -> 81 0.69168

Excited State 4: 3.9786 eV 311.63 nm f=0.0455

77 -> 81 0.69373

80 -> 83 -0.10294

Excited State 5: 4.6330 eV 267.61 nm f=0.0008

76 -> 81 0.68092

80 -> 84 0.12113

***Z*-3** (F4-bisalcohol)

Excited State 1: 2.8131 eV 440.73 nm f=0.0637

76 -> 81 -0.24003

80 -> 81 0.65293

Excited State 2: 3.9709 eV 312.23 nm f=0.0193

79 -> 81 0.69069

Excited State 3: 4.0461 eV 306.43 nm f=0.0256

78 -> 81 0.67339

Excited State 4: 4.1614 eV 297.94 nm f=0.1374

74 -> 81 0.15475

76 -> 81 -0.38538

77 -> 81 0.51906

80 -> 81 -0.19591

Excited State 5: 4.2297 eV 293.12 nm f=0.1087

76 -> 81 0.48145

77 -> 81 0.43572

78 -> 81 -0.14810

80 -> 81 0.15401

***E*-4** (F4-aldehyde)

Excited State 1: 2.4420 eV 507.72 nm f=0.0873

69 -> 72 -0.27464

71 -> 72 0.62499

71 -> 73 -0.13828

Excited State 2: 3.5744 eV 346.87 nm f=0.0003

67 -> 72 0.61263

67 -> 73 0.33188

Excited State 3: 3.6939 eV 335.65 nm f=0.0196

70 -> 72   0.69427

Excited State 4: 3.7911 eV 327.04 nm f=1.0080

69 -> 72   0.62015

71 -> 72   0.30723

Excited State 5: 3.8180 eV 324.73 nm f=0.0318

68 -> 72   0.69011

***Z*-4** (F4-aldehyde)

Excited State 1: 2.7747 eV 446.84 nm f=0.0647

66 -> 72   -0.18758

71 -> 72   0.64168

71 -> 73   -0.19762

Excited State 2: 3.6720 eV 337.65 nm f=0.0001

67 -> 72   0.11236

68 -> 72   0.56858

68 -> 73   0.37152

68 -> 77   -0.10858

Excited State 3: 3.8020 eV 326.10 nm f=0.0126

70 -> 72   0.69347

Excited State 4: 3.9960 eV 310.27 nm f=0.0177

67 -> 72   -0.20001

69 -> 72   0.62216

71 -> 72   -0.11686

71 -> 73   -0.14490

Excited State 5: 4.0822 eV 303.72 nm f=0.0668

66 -> 72   0.12843

67 -> 72 0.48697

67 -> 73 -0.11428

69 -> 72 0.30187

71 -> 72 0.15925

71 -> 73 0.29711

***E*-5 (**F4-bisaldehyde)

Excited State 1: 2.3329 eV 531.46 nm f=0.1143

75 -> 79 -0.25826

78 -> 79 0.63593

78 -> 81 0.13720

Excited State 2: 3.4766 eV 356.62 nm f=0.0003

73 -> 80 -0.31931

74 -> 79 0.59807

74 -> 81 -0.16334

Excited State 3: 3.4770 eV 356.59 nm f=0.0001

73 -> 79 0.59757

73 -> 81 -0.16319

74 -> 80 -0.31982

Excited State 4: 3.6224 eV 342.27 nm f=0.0139

77 -> 79 0.69259

Excited State 5: 3.6228 eV 342.23 nm f=0.0672

76 -> 79 0.68732

***Z*-5 (**F4-bisaldehyde)

Excited State 1: 2.7221 eV 455.47 nm f=0.0780

72 -> 79 -0.18707

78 -> 79 0.64881

78 -> 81 -0.19273

Excited State 2: 3.6145 eV 343.02 nm f=0.0001

74 -> 79 0.55045

74 -> 80 -0.36183

74 -> 81 0.21902

Excited State 3: 3.6189 eV 342.60 nm f=0.0001

75 -> 79 0.54558

75 -> 80 0.36908

75 -> 81 0.21269

Excited State 4: 3.7686 eV 328.99 nm f=0.0254

77 -> 79 0.69249

Excited State 5: 3.8836 eV 319.25 nm f=0.0006

76 -> 79 0.68987

***E*-6 (**F3-alcohol)

Excited State 1: 2.5565 eV 484.98 nm f=0.0446

65 -> 69 0.12313

67 -> 69 0.29247

68 -> 69 0.62353

Excited State 2: 3.8786 eV 319.66 nm f=0.6390

67 -> 69 0.63467

68 -> 69 -0.29005

Excited State 3: 3.9939 eV 310.43 nm f=0.0188

66 -> 69 0.69455

Excited State 4: 4.0997 eV 302.42 nm f=0.3139

65 -> 69 0.67732

68 -> 69 -0.14315

Excited State 5: 4.8711 eV 254.53 nm f=0.0020 <

63 -> 69 -0.19523

64 -> 69 0.65403

68 -> 72 0.11987

***Z*-6 (**F3-alcohol)

Excited State 1: 2.8508 eV 434.90 nm f=0.0374

63 -> 69 -0.10850

64 -> 69 -0.17410

68 -> 69 0.66428

Excited State 2: 4.0227 eV 308.21 nm f=0.0216

67 -> 69 0.68008

Excited State 3: 4.2005 eV 295.16 nm f=0.0193

63 -> 69 0.11409

64 -> 69 0.13504

65 -> 69 0.29324

66 -> 69 0.59521

Excited State 4: 4.2576 eV 291.20 nm f=0.0347

65 -> 69 0.60817

66 -> 69 -0.30295

Excited State 5: 4.4800 eV 276.75 nm f=0.1499

63 -> 69 0.26313

64 -> 69 0.58001

66 -> 69 -0.17729

68 -> 69 0.17758

***E*-7** (F3-aldehyde)

Excited State 1: 2.5339 eV 489.31 nm f=0.0414

66 -> 68 -0.28147

67 -> 68 0.62339

67 -> 69 0.10543

Excited State 2: 3.6990 eV 335.19 nm f=0.0054

63 -> 68 -0.14367

63 -> 69 0.40803

64 -> 68 0.11213

64 -> 69 -0.15717

66 -> 69 -0.23440

67 -> 69 0.44334

Excited State 3: 3.8588 eV 321.30 nm f=0.5500

63 -> 69 0.23654

64 -> 68 -0.11895

66 -> 68 0.53247

67 -> 68 0.26974

67 -> 69 -0.21836

Excited State 4: 3.9103 eV 317.07 nm f=0.0196

65 -> 68 0.68938

Excited State 5: 3.9982 eV 310.10 nm f=0.3553

63 -> 68 0.19246

63 -> 69 -0.29581

66 -> 68 0.34243

66 -> 69 0.12597

67 -> 68 0.11097

67 -> 69 0.45131

***Z*-7** (F3-aldehyde)

Excited State 1: 2.8445 eV 435.88 nm f=0.0373 <S**2>=0.000

62 -> 68 0.17657

67 -> 68 0.62709

67 -> 69 0.23462

Excited State 2: 3.7383 eV 331.66 nm f=0.0016

63 -> 68 -0.19816

63 -> 69 0.24539

64 -> 69 -0.10259

65 -> 68 -0.27899

65 -> 69 0.42861

67 -> 68 0.10325

67 -> 69 -0.28257

Excited State 3: 3.9364 eV 314.97 nm f=0.0319

63 -> 69 0.15415

65 -> 68 -0.24975

65 -> 69 0.13969

66 -> 68 -0.32030

67 -> 68 -0.23278

67 -> 69 0.46659

Excited State 4: 4.0147 eV 308.83 nm f=0.0179

65 -> 68 -0.14224

66 -> 68 0.60231

66 -> 69 0.13358

67 -> 68 -0.10414

67 -> 69 0.25987

Excited State 5: 4.2286 eV 293.20 nm f=0.0357

62 -> 68	0.14900
63 -> 68	0.38199
64 -> 68	0.40307
64 -> 69	0.14375
65 -> 68	-0.26155
65 -> 69	-0.14340
67 -> 69	-0.15535

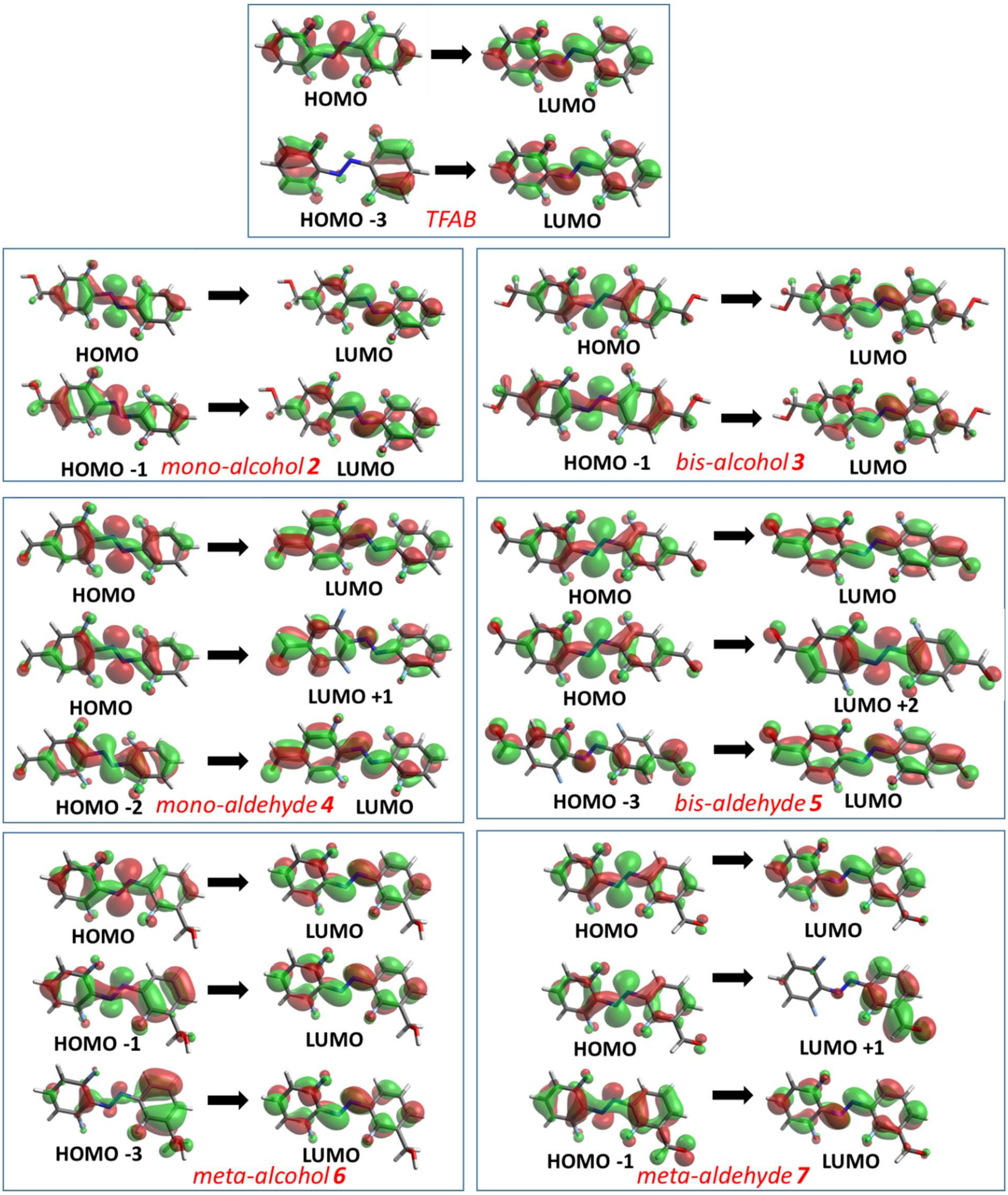

*Figure S 7: Visualized transitions of molecular orbitals, of which – in the TD-DFT calculations – the excited state 1 is composed for the corresponding E-isomer of molecules **2-7**.*

*Table S19: Excitation features of the compounds **2-7**; TD-B3LYP/6-311G*/PCM (ACN) level of theory.*

Compound	isomer	transition	λ [nm]	osc. strength	$\Delta\lambda_{n-\pi^*}$[nm]
**2**	*Z*	n→π*	438	0.0539	48
	Z	π→π*	312	0.0177	
	E	n→π*	486	0.0656	
	E	π→π*	324	0.9371	
**3**	*Z*	n→π*	441	0.0637	45
	Z	π→π*	312	0.0193	
	E	n→π*	486	0.0739	
	E	π→π*	333	1.0980	
**4**	*Z*	n→π*	447	0.0647	61
	Z	π→π*	338	0.0001	
	E	n→π*	508	0.0873	
	E	π→π*	347	0.0003	
**5**	*Z*	n→π*	456	0.0780	75
	Z	π→π*	343	0.0001	
	E	n→π*	531	0.1143	
	E	π→π*	357	0.0003	
**6**	*Z*	n→π*	435	0.0374	50
	Z	π→π*	308	0.0216	
	E	n→π*	485	0.0446	
	E	π→π*	320	0.6390	
**7**	*Z*	n→π*	436	0.0373	53
	Z	π→π*	332	0.0016	
	E	n→π*	489	0.0414	
	E	π→π*	335	0.0054	

Simulated UV/Vis Spectra

The UV/Vis spectra were simulated based on TD calculations (25 states in total) with Gaussview assuming a gaussian band shape (characterized by a standard deviation s = 0.2 eV) and the extracted data was plotted using OriginPro 2020 9.7.188 with the peaks, furnished by the calculation.

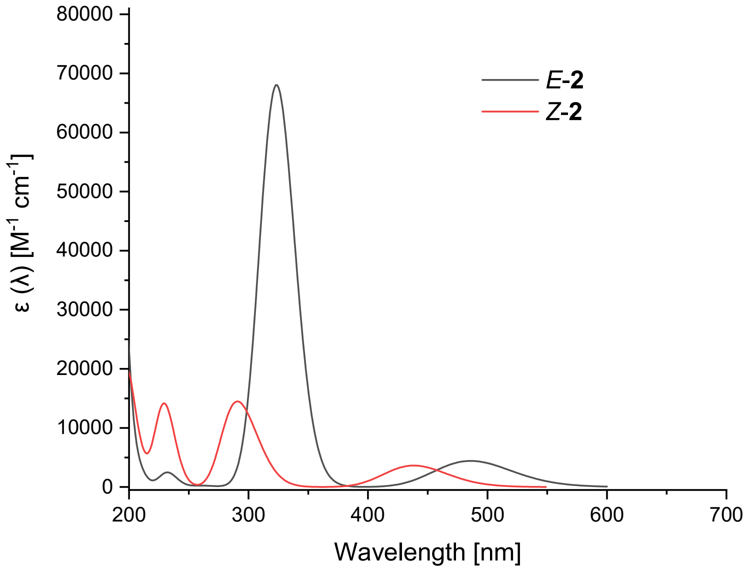

80000
70000
60000
50000
40000
30000
20000
10000
0
ε (λ) [M-1 cm-1]
200
300
400
500
600
700
Wavelength [nm]
E-2
Z-2

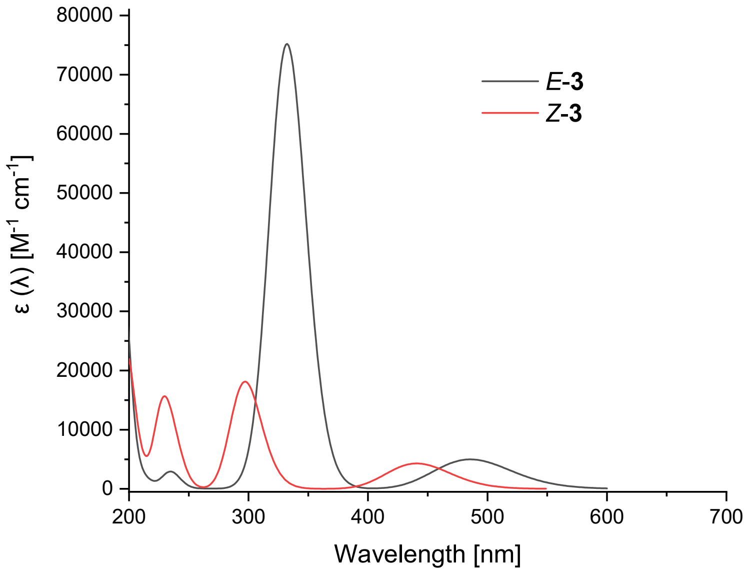

80000
70000
60000
50000
40000
30000
20000
10000
0
ε (λ) [M-1 cm-1]
200
300
400
500
600
700
Wavelength [nm]
E-3
Z-3

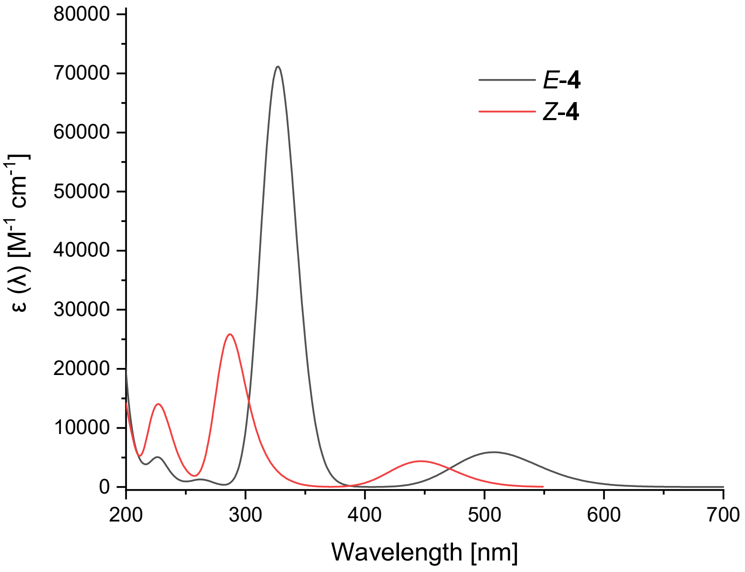

80000
70000
60000
50000
40000
30000
20000
10000
0
ε (λ) [M-1 cm-1]
200
300
400
500
600
700
Wavelength [nm]
E-4
Z-4

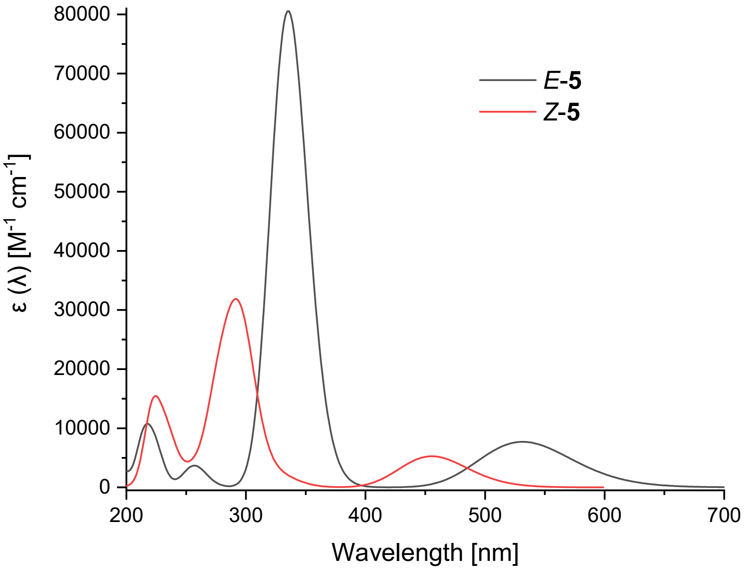

80000
70000
60000
50000
40000
30000
20000
10000
0
ε (λ) [M-1 cm-1]
200
300
400
500
600
700
Wavelength [nm]
E-5
Z-5

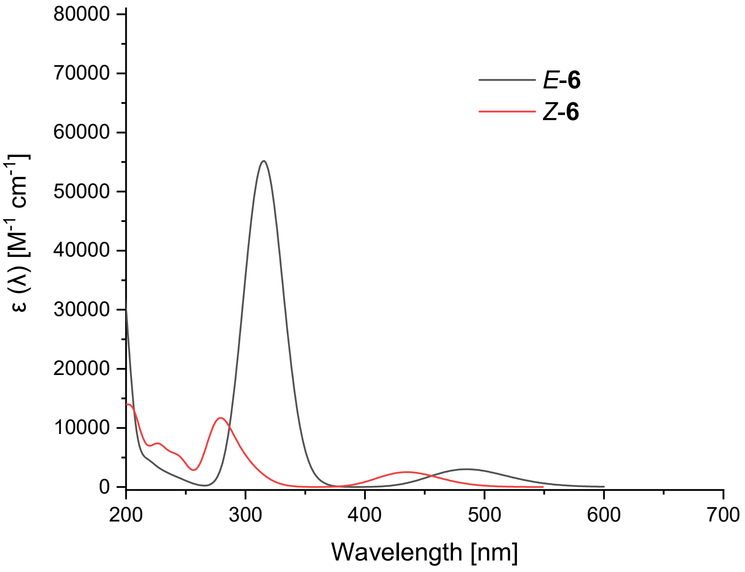

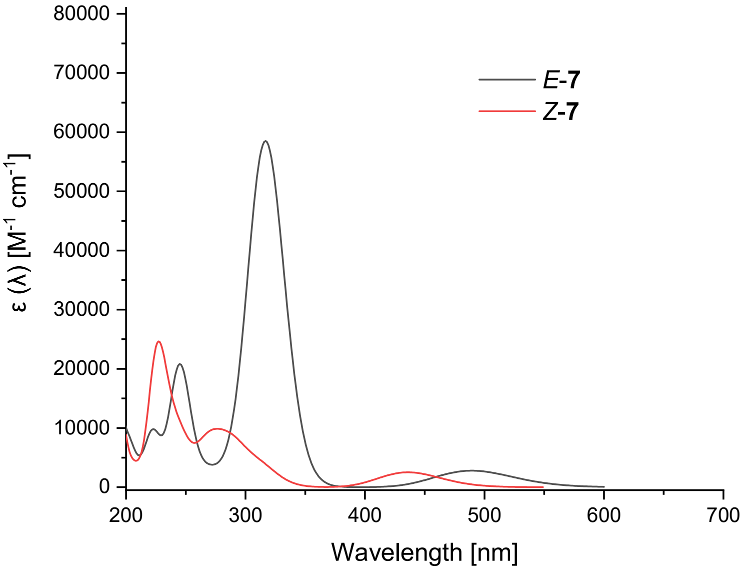

Figure S8. Simulated UV-Vis spectra of the *E*- (black curves) and *Z*-isomers (red curves) of compounds **2-7**.

Measured spectra

Calculated spectra

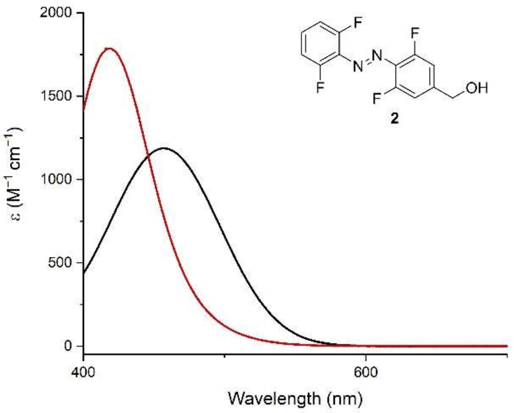

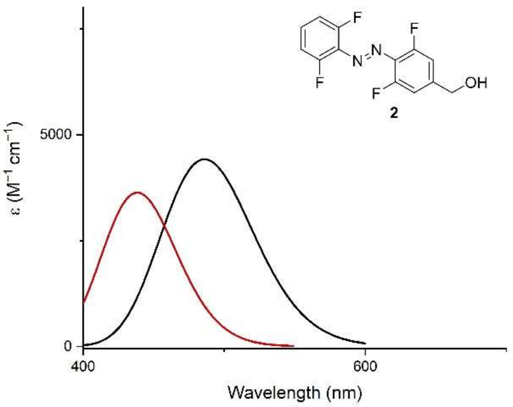

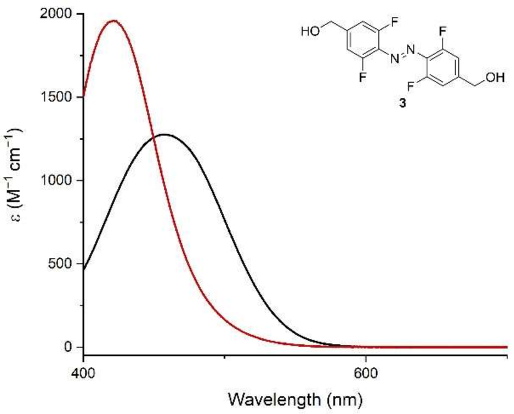

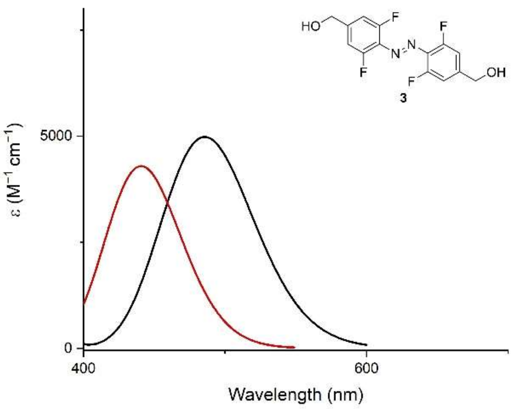

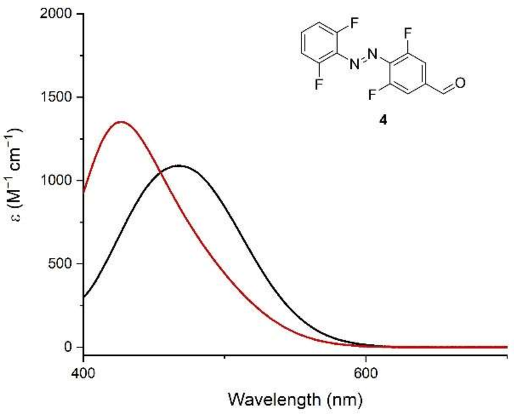

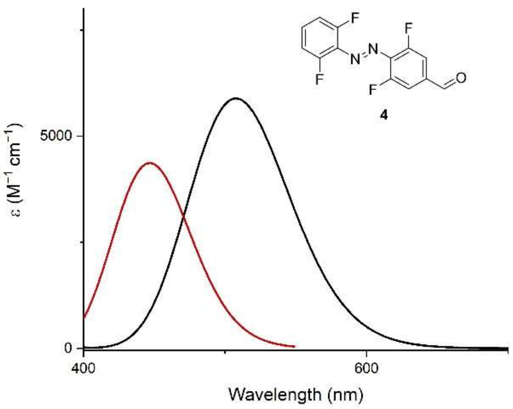

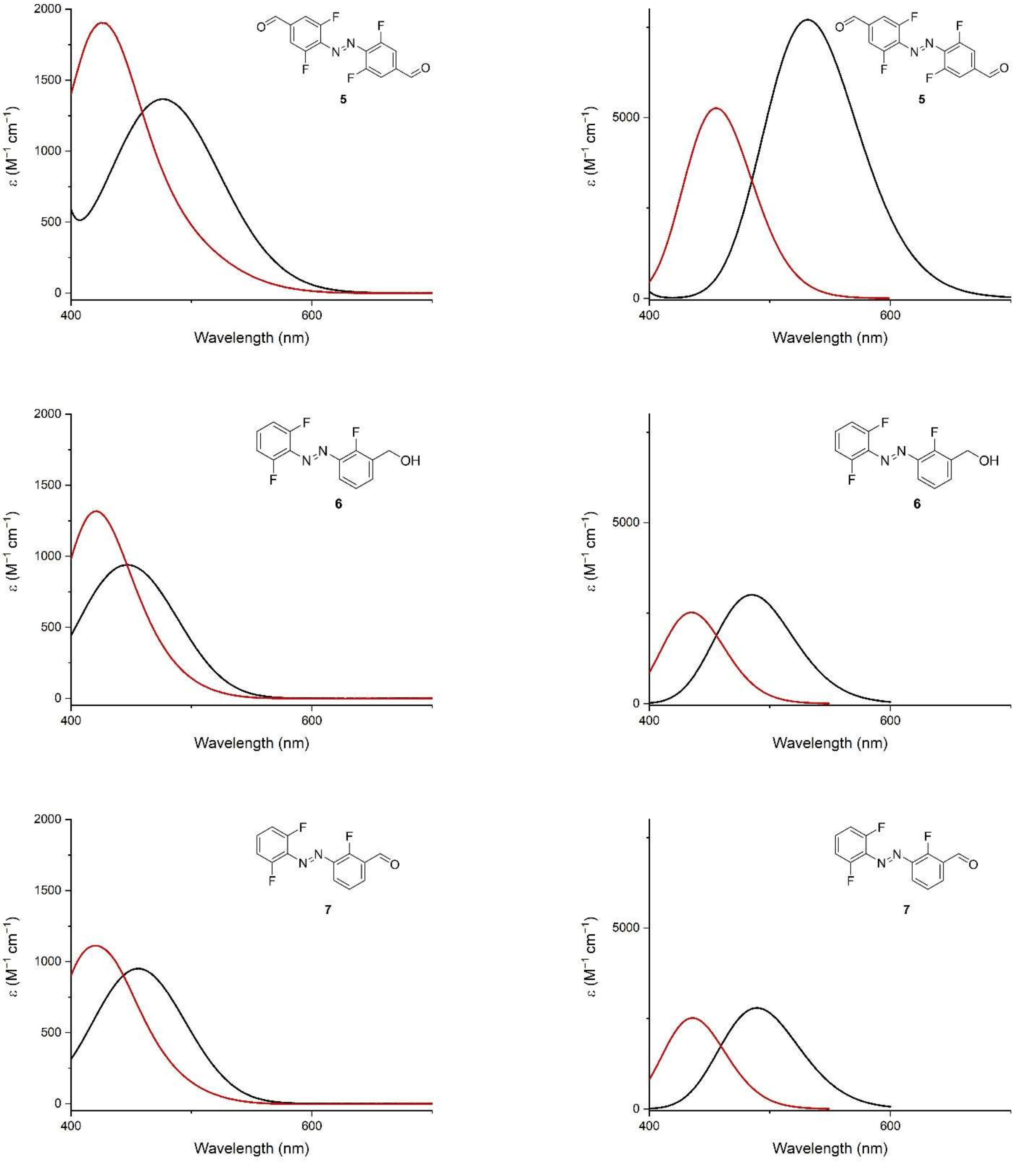

Figure S9. Comparison of the measured (*left*) and calculated (*right*) UV-Vis spectra of compounds **2-7** in the visible light range (>400 nm). Black curves – *E*-isomers, red curves – *Z*-isomers.

5. UV/Vis isomerization experiments

Stock solutions were prepared for each substance. To determine the final concentration where a maximum of absorbance about 1.0 au was reached, each compound was measured at a concentration of 50 µM and the thereupon calculated final concentrations are 38 µM (**5**) and 33 µM (**8**). The cuvettes with the samples were irradiated with light of the indicated wavelength directly before the measurement.

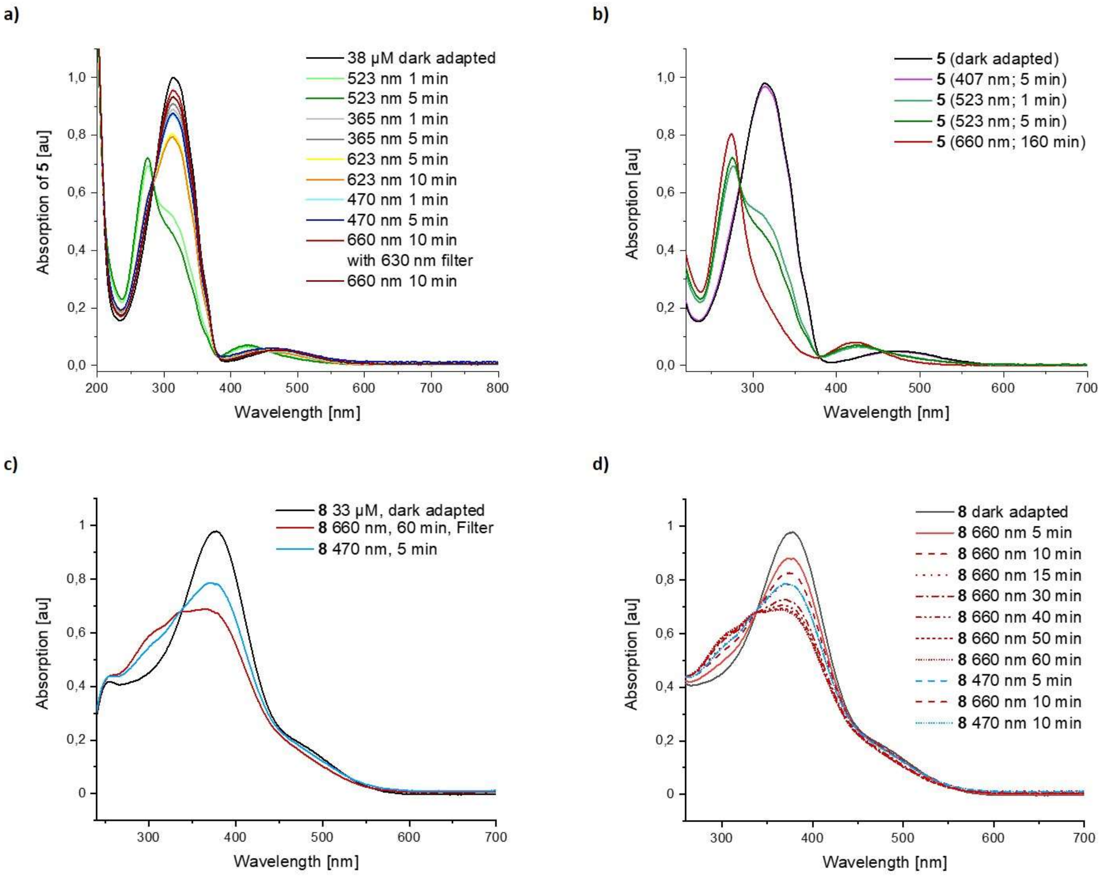

*Figure S 10: a) The absorption curves display a distinct isosbestic point of compound **5**. b) Photostationary state of dialdehyde **5** after irradiation at 407 nm, 523 nm and 660 nm. c,d) absorption curves of **8** before and after irradiation.*

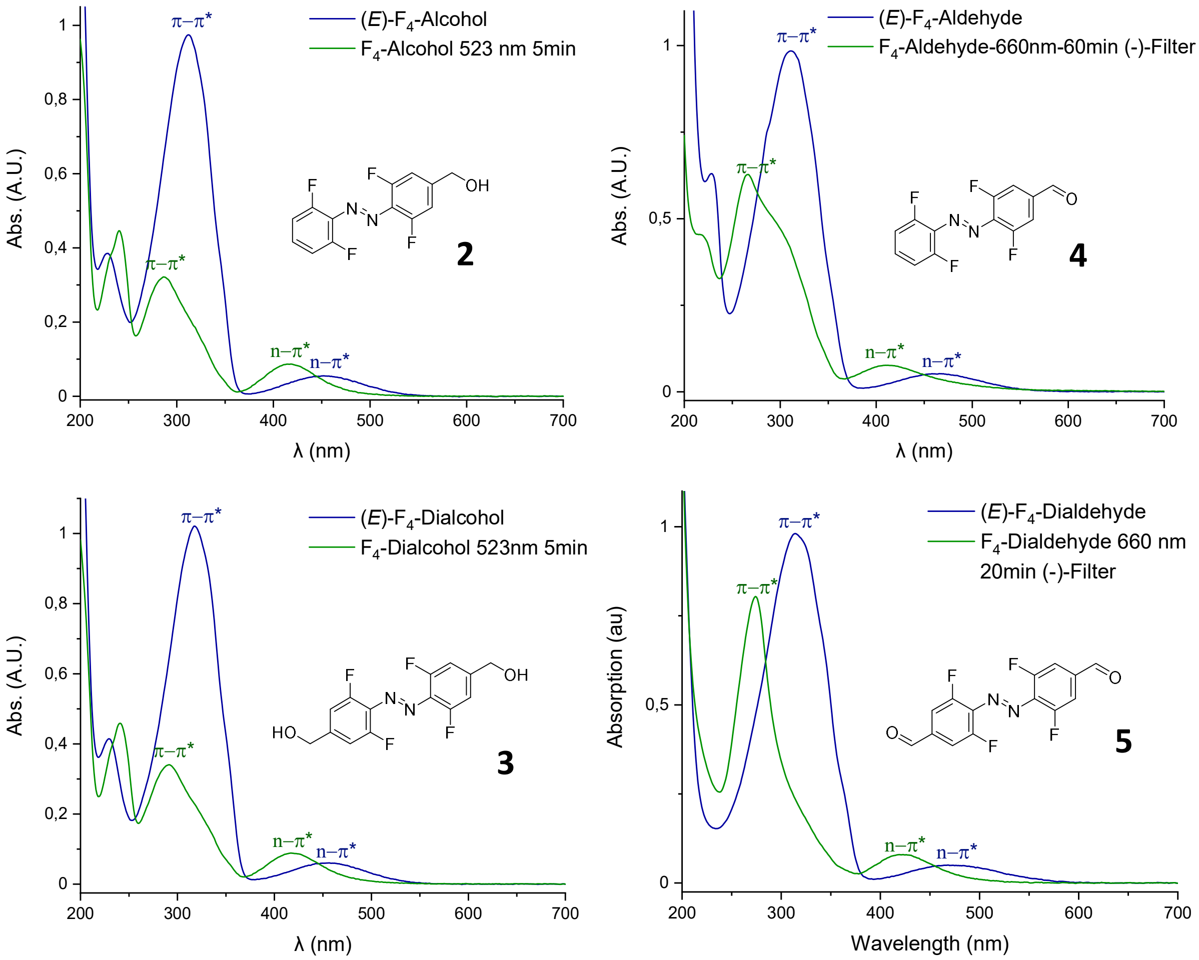

*Figure S 11: UV-Vis spectra measured for the compounds **2**-**5** – comparison of the E-isomer ("dark state") and the irradiated mixture (with given wavelength and time) containing the majority of Z-isomer*

6. Calculation of the attenuation coefficients

A 1.5 mM solution of compound **2 – 8** was prepared by dissolving the dry substance in d_6-DMSO. The solution was transferred to a 1.5 mL quartz cuvette and the absorption (400 nm-700 nm) was measured. Subsequently the cuvette was irradiated at 523 nm (15 min) and the absorption measured again. An aliquot (0.55 mL) of the irradiated solution was directly measured by NMR and the *E*/*Z* ratio was determined.

The molar extinction coefficient **ε** (M^{-1} cm^{-1}) was calculated based on the Lambert-Beer Law: **A** is the absorbance, **c** is the concentration (M) and **l** the optical path-length (cm).

$$A = \varepsilon * \mathrm{c} * \mathrm{l}$$

The absorbance spectrum of the *Z*-isomer was calculated by the following formula:

$$A_{Z\ isomer} = \frac{A_{PSS,523nm} - \left(A_{E\ isomer} * \left(\% \frac{E\ isomer}{100}\right)\right)}{\left(\% \frac{Z\ isomer}{100}\right)}$$

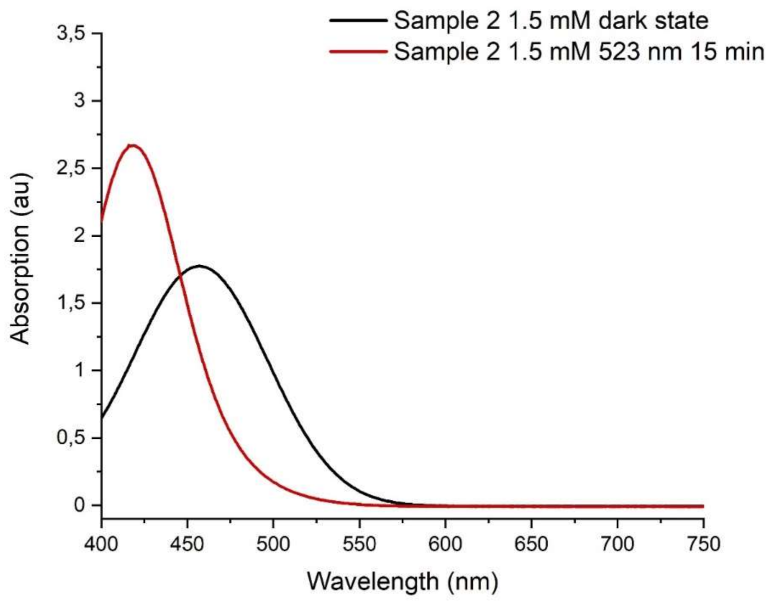

Figure S 12: Absorbance spectrum of ***2****. Raw data before calculation., y-axis: absorption (au), x-axis wavelength (nm).*

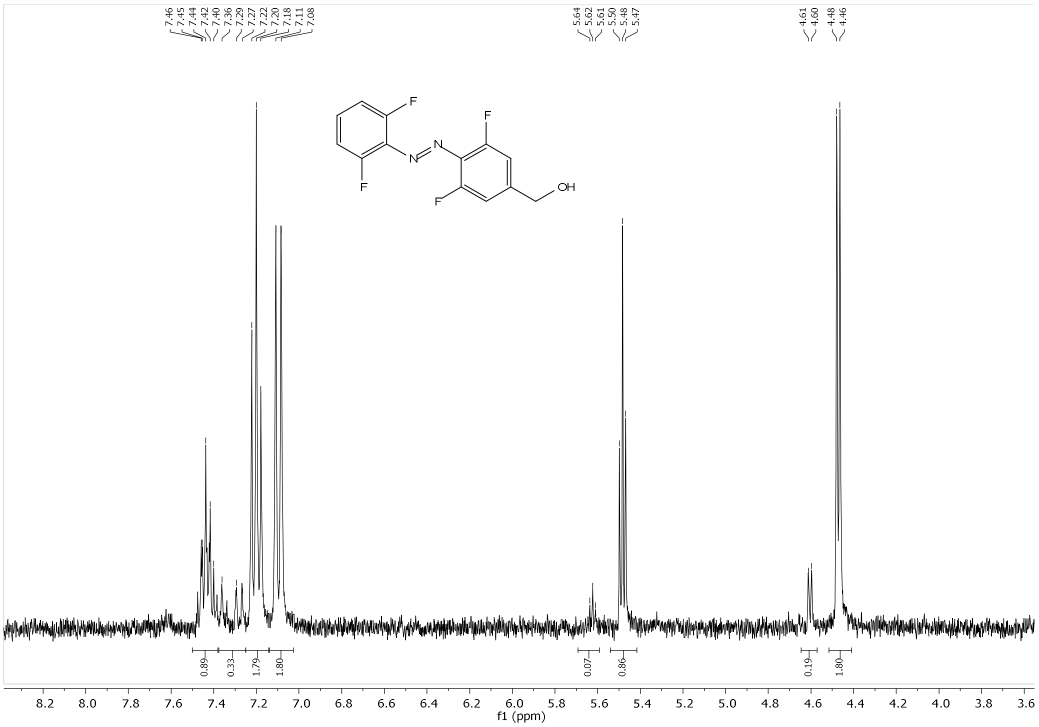

Figure S 13: 1H-NMR-spectrum (400 MHz, 16 scans, DMSO-d6) of ***2*** *(1.5 mM) after irradiation at 523 nm.*

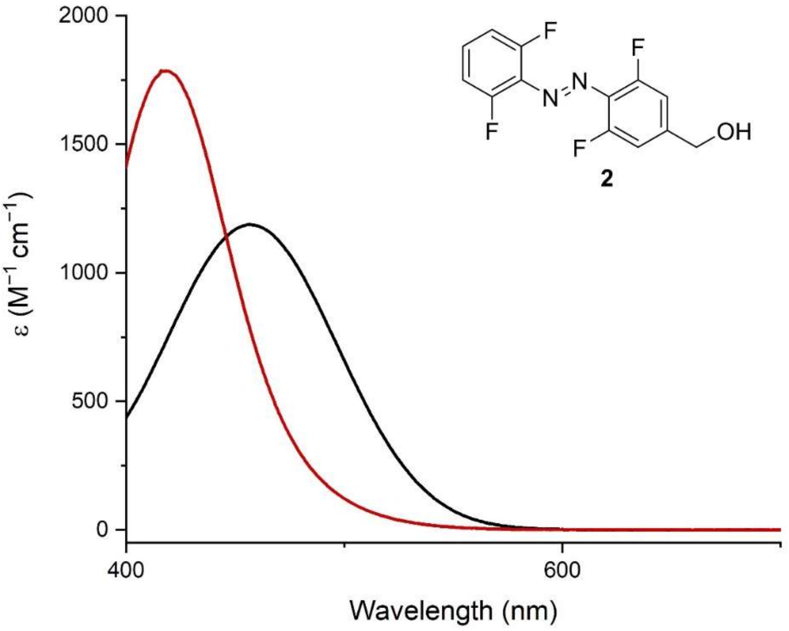

*Figure S 14: Molar extinction coefficient of **2-E** (black) and **2-Z** (red).*

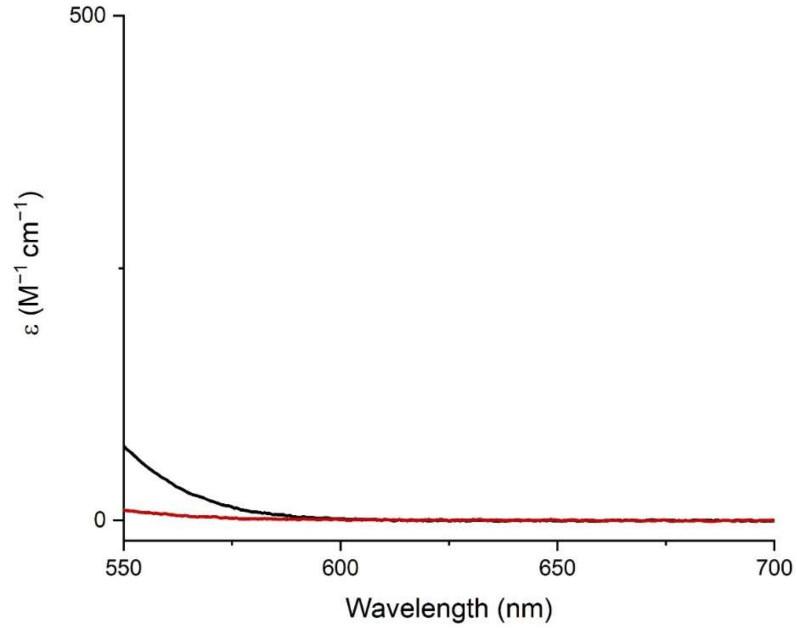

*Figure S 15: Molar extinction coefficient of **2-E** (black) and **2-Z** (red), section between 550 nm – 700 nm.*

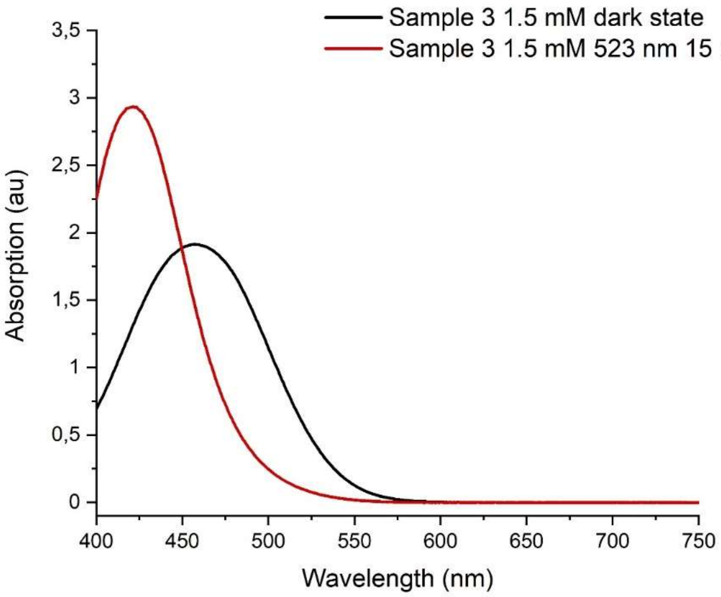

*Figure S 16: Absorbance spectrum of **3**. Raw data before calculation., y-axis: absorption (au), x-axis wavelength (nm).*

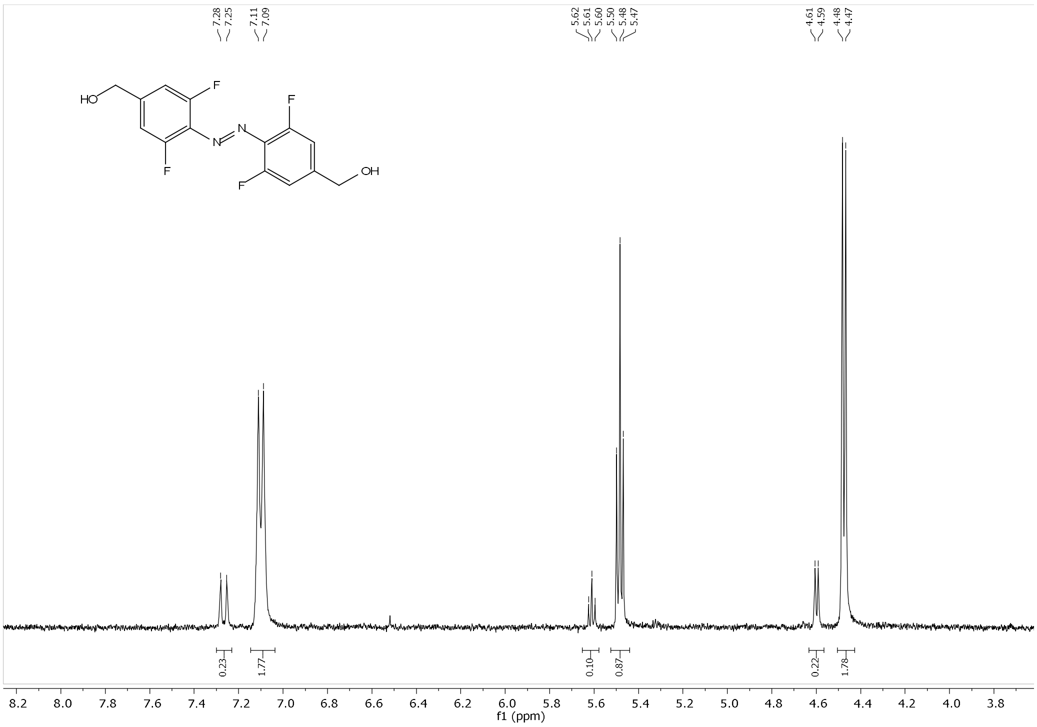

*Figure S 17: 1H-NMR-spectrum (400 MHz, 32 scans, DMSO-d6) of **3** (1.5 mM) after irradiation at 523 nm.*

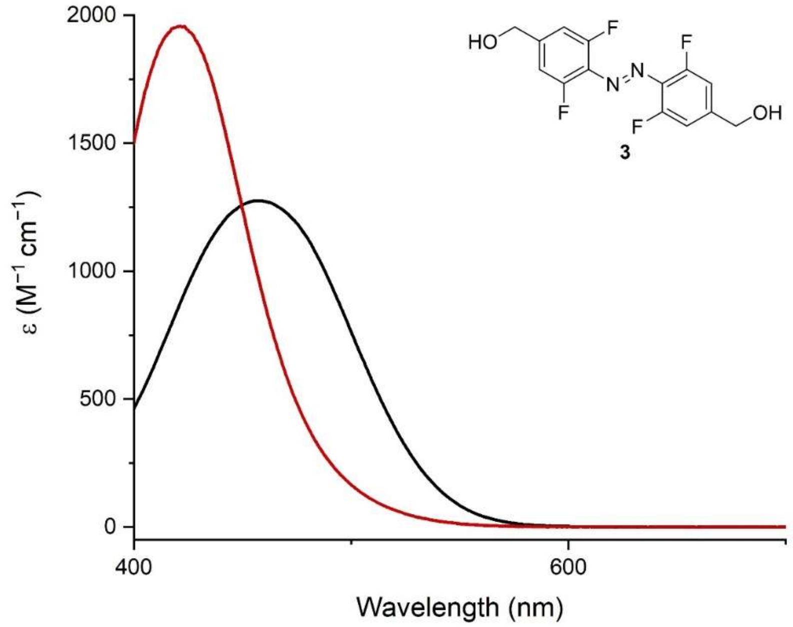

Figure S 18: Molar extinction coefficient of ***3-E*** *(black) and* ***3-Z*** *(red).*

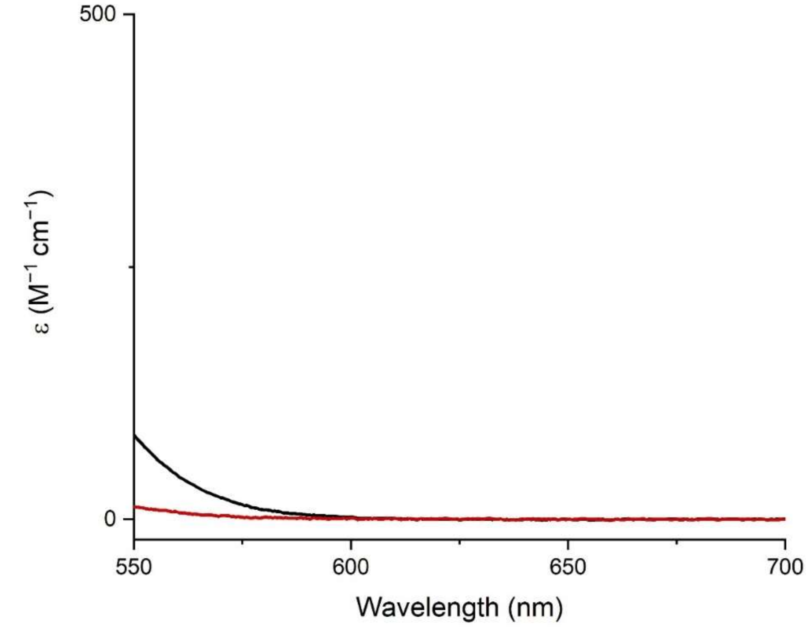

Figure S 19: Molar extinction coefficient of ***3-E*** *(black) and* ***3-Z*** *(red), section between 550 nm – 700 nm.*

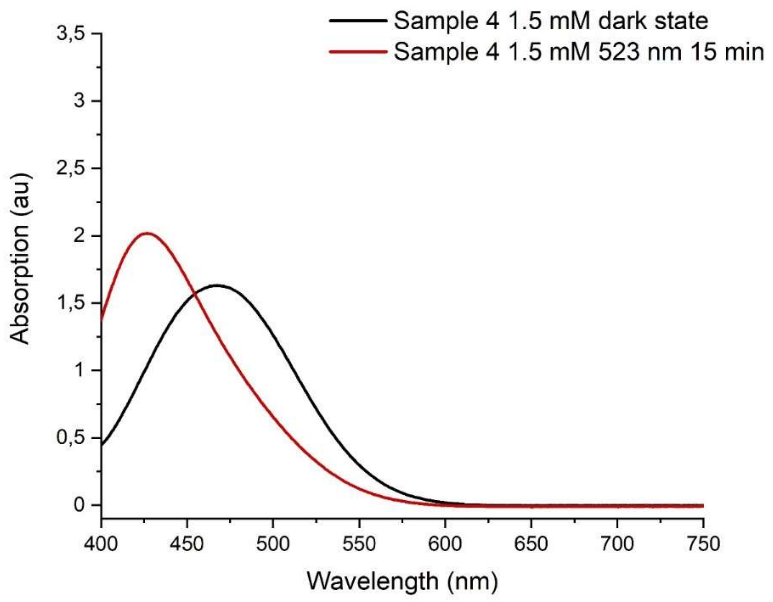

*Figure S 20: Absorbance spectrum of **4**. Raw data before calculation., y-axis: absorption (au), x-axis wavelength (nm).*

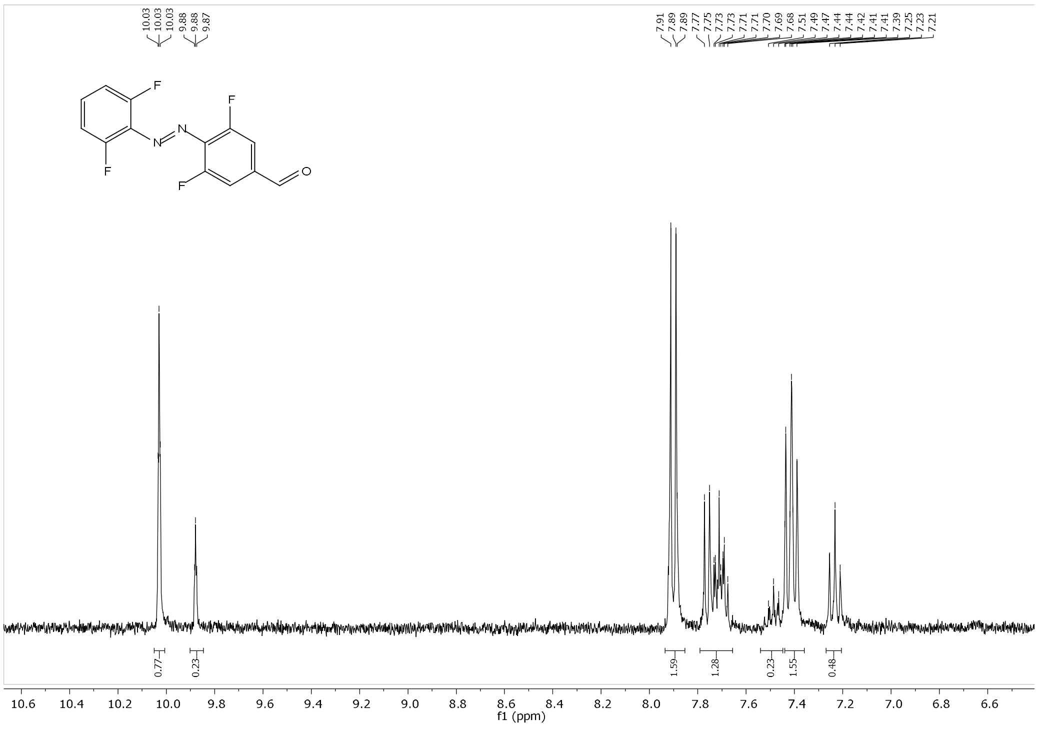

*Figure S 21: 1H-NMR-spectrum (400 MHz, 32 scans, DMSO-d6) of **4** (1.5 mM) after irradiation at 523 nm.*

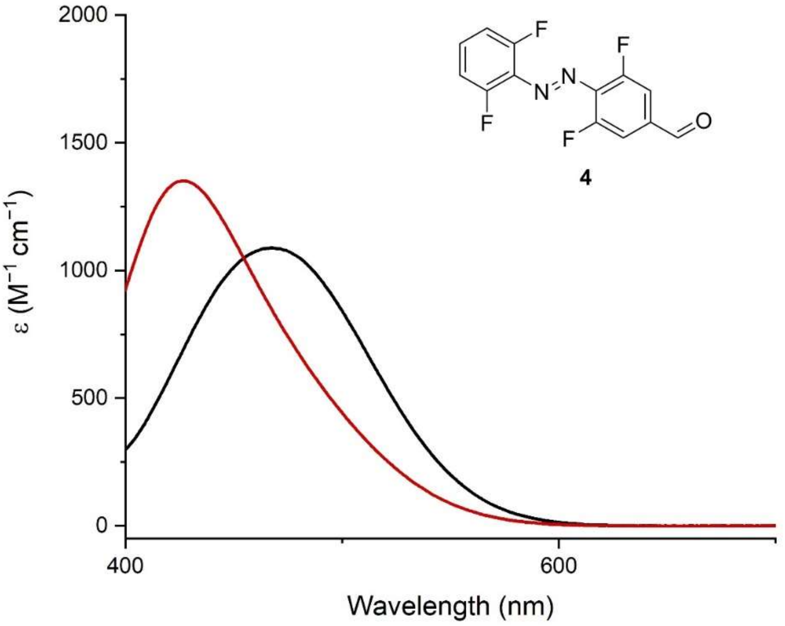

*Figure S 22: Molar extinction coefficient of **4-E** (black) and **4-Z** (red).*

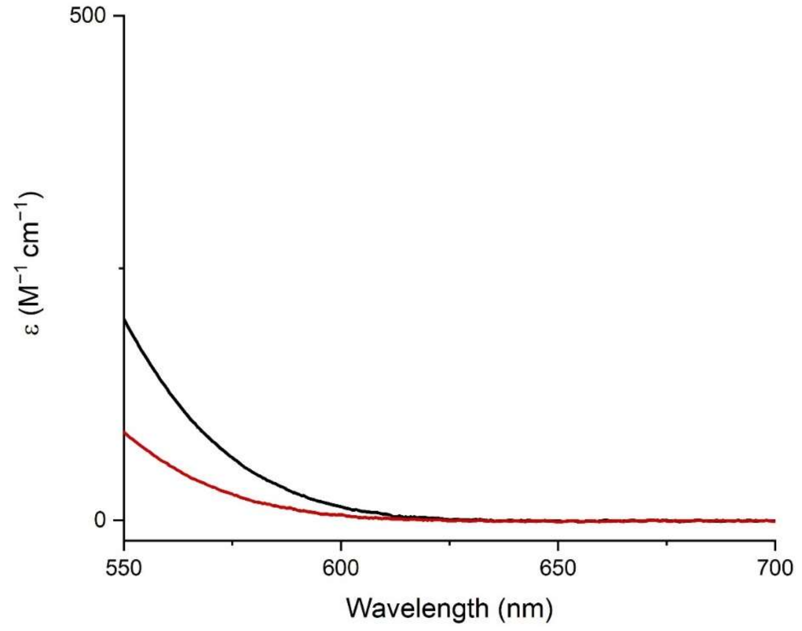

*Figure S 23: Molar extinction coefficient of **4-E** (black) and **4-Z** (red), section between 550 nm – 700 nm.*

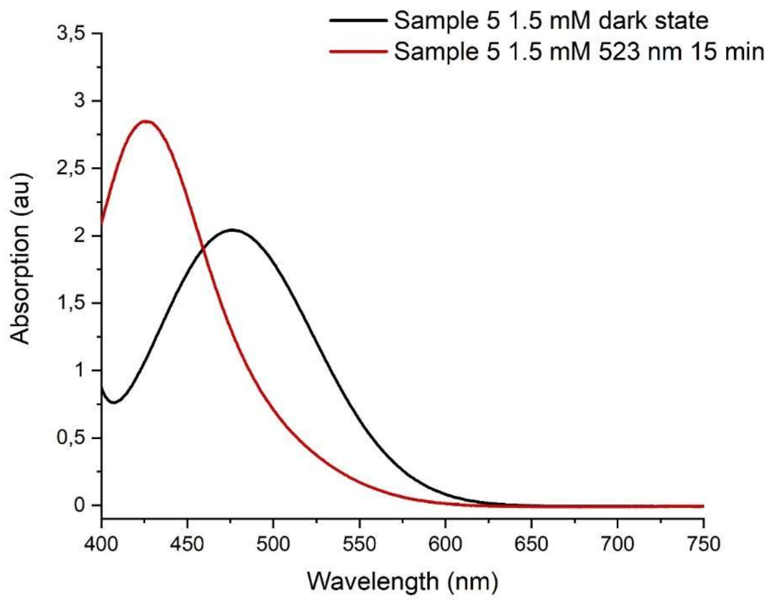

*Figure S 24: Absorbance spectrum of **5**. Raw data before calculation., y-axis: absorption (au), x-axis wavelength (nm).*

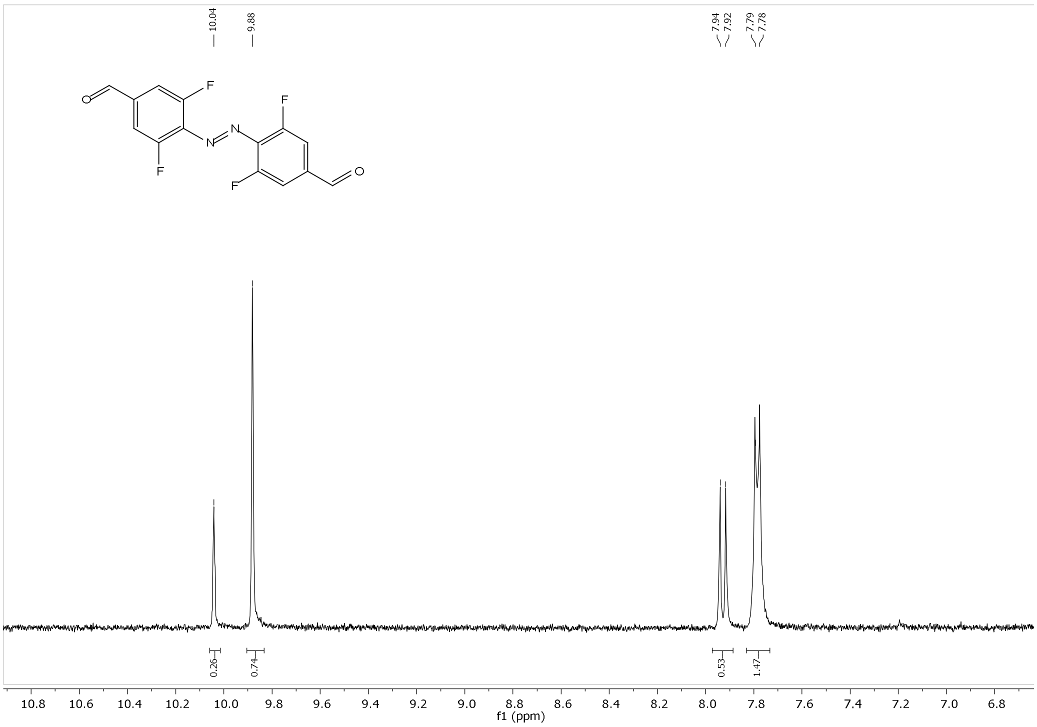

*Figure S 25: 1H-NMR-spectrum (400 MHz, 32 scans, DMSO-d6) of **5** (1.5 mM) after irradiation at 523 nm.*

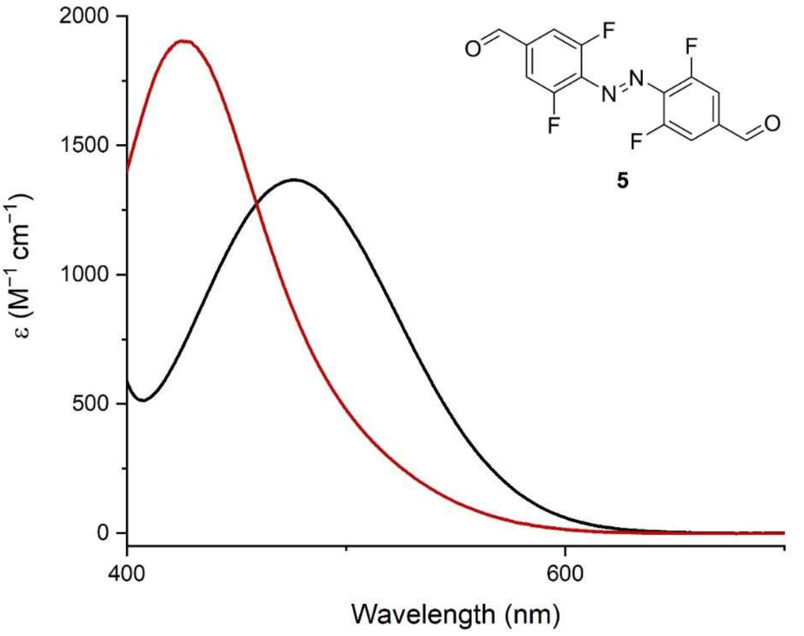

*Figure S 26: Molar extinction coefficient of **5-E** (black) and **5-Z** (red).*

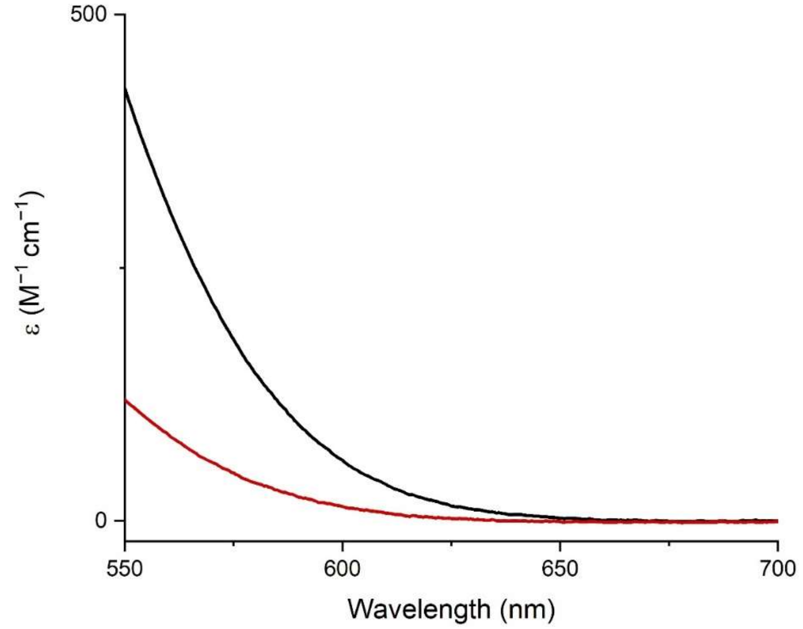

*Figure S 27: Molar extinction coefficient of **5-E** (black) and **5-Z** (red), section between 550 nm – 700 nm.*

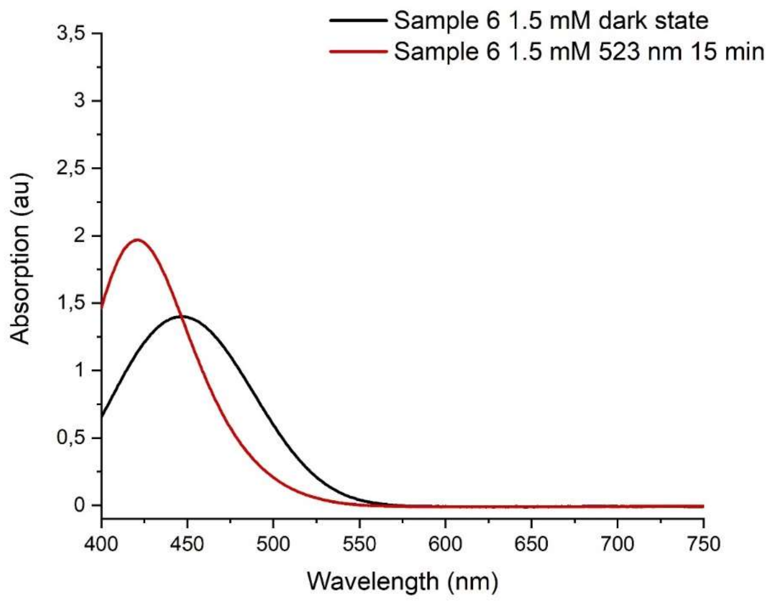

*Figure S 28: Absorbance spectrum of **6**. Raw data before calculation., y-axis: absorption (au), x-axis wavelength (nm).*

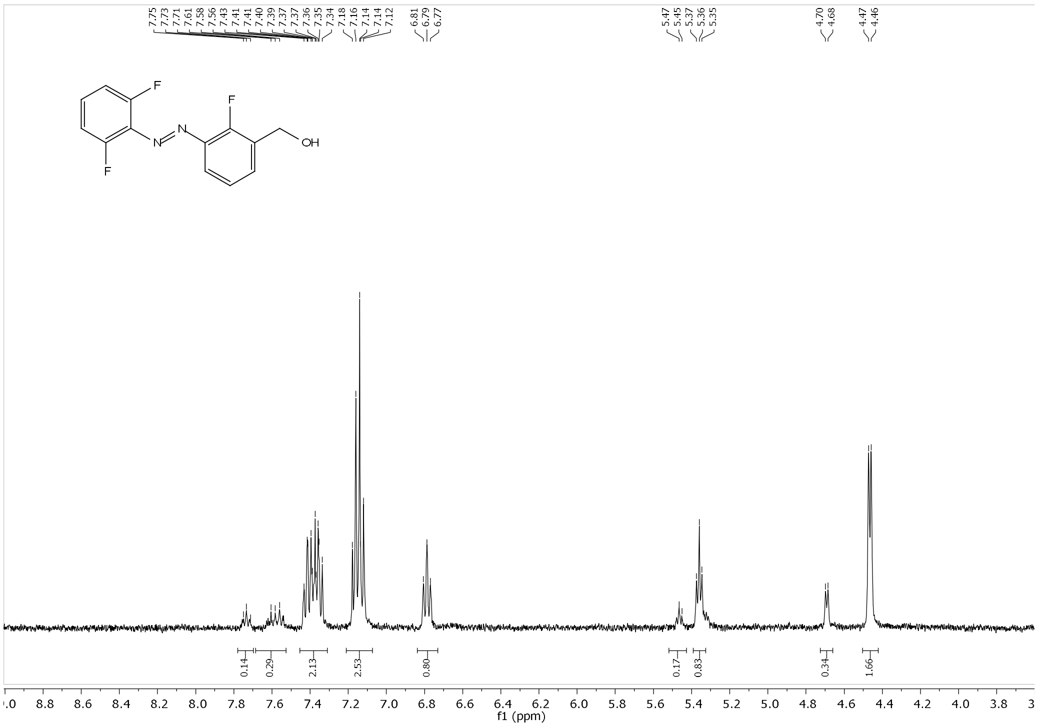

*Figure S 29: 1H-NMR-spectrum (400 MHz, 32 scans, DMSO-d6) of **6** (1.5 mM) after irradiation at 523 nm.*

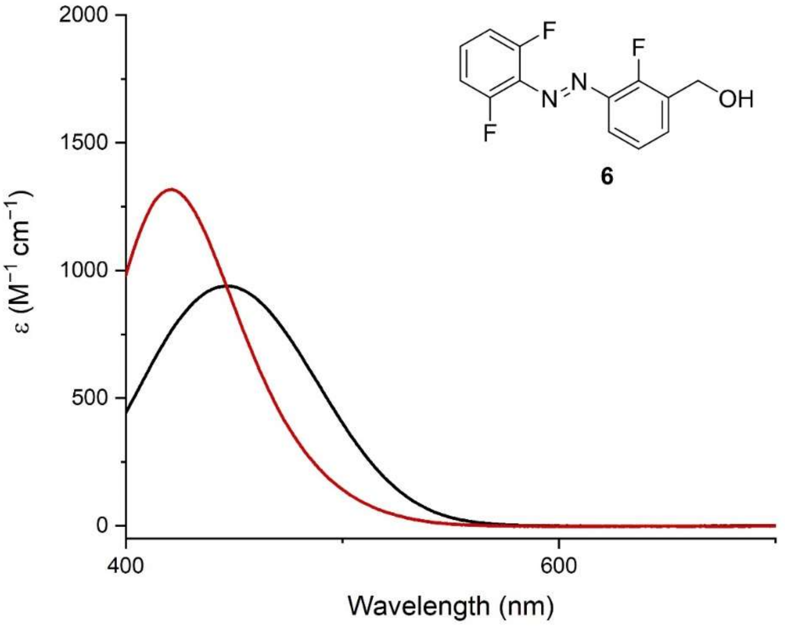

Figure S 30: Molar extinction coefficient of ***6-E*** *(black) and* ***6-Z*** *(red).*

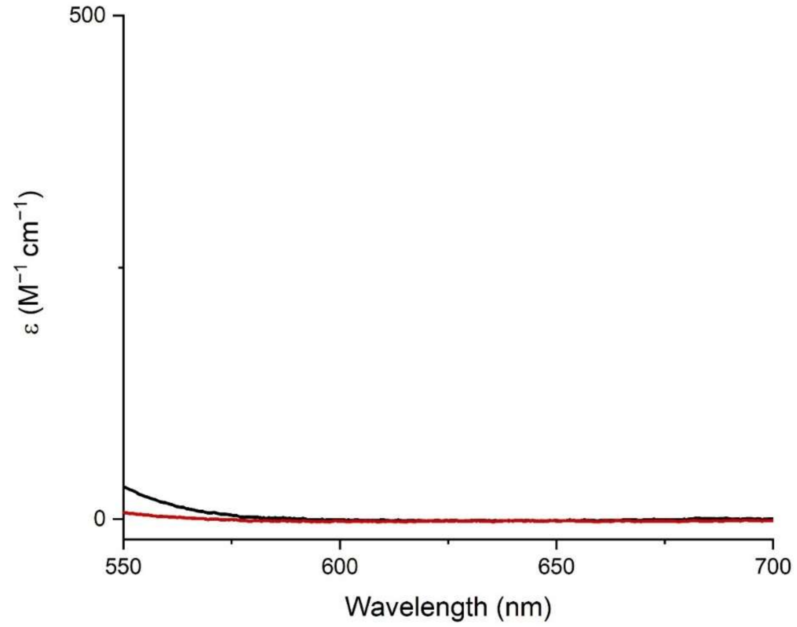

Figure S 31: Molar extinction coefficient of ***6-E*** *(black) and* ***6-Z*** *(red), section between 550 nm – 700 nm.*

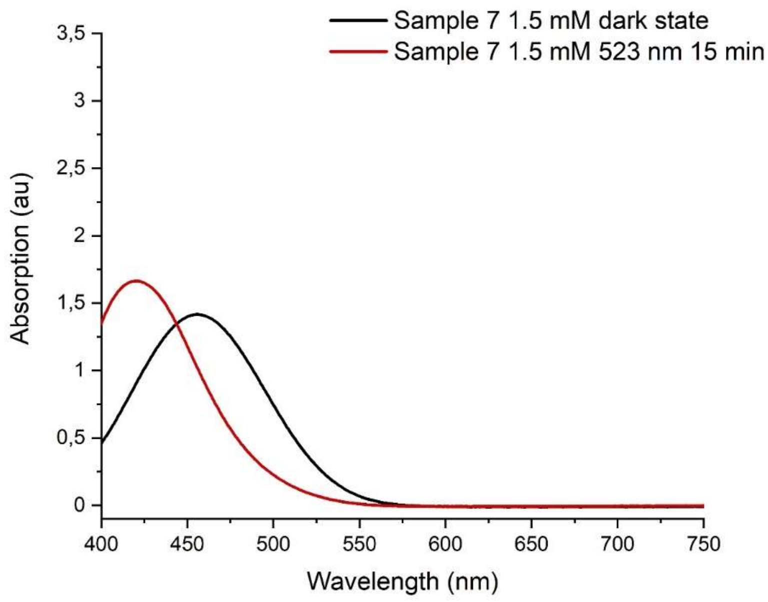

*Figure S 32: Absorbance spectrum of **7**. Raw data before calculation., y-axis: absorption (au), x-axis wavelength (nm).*

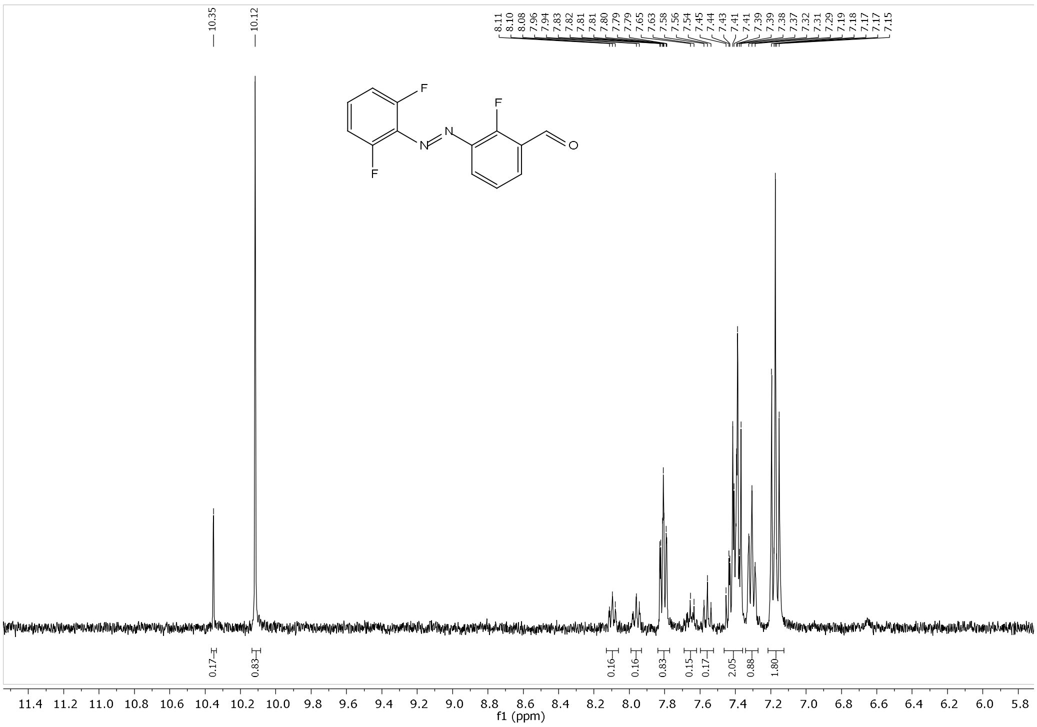

*Figure S 33: 1H-NMR-spectrum (400 MHz, 32 scans, DMSO-d6) of **7** (1.5 mM) after irradiation at 523 nm.*

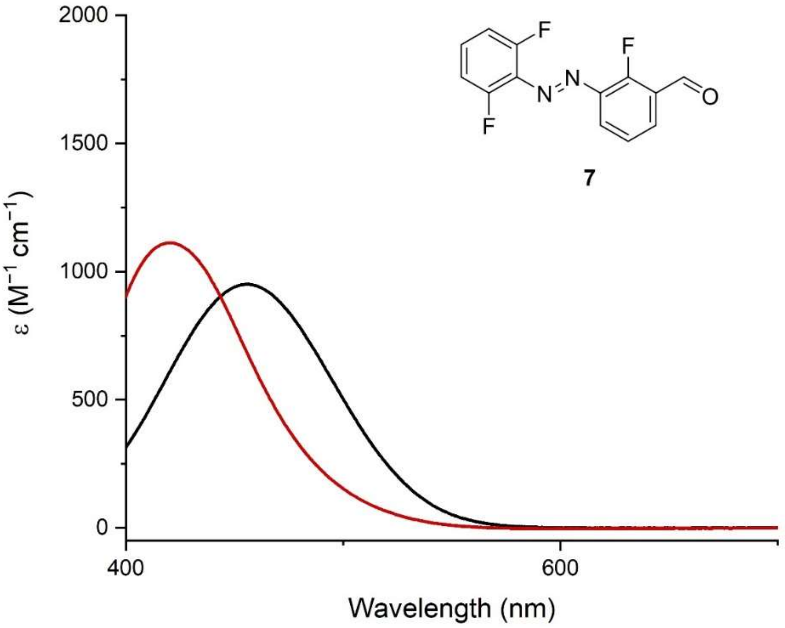

Figure S 34: Molar extinction coefficient of ***7-E*** *(black) and* ***7-Z*** *(red).*

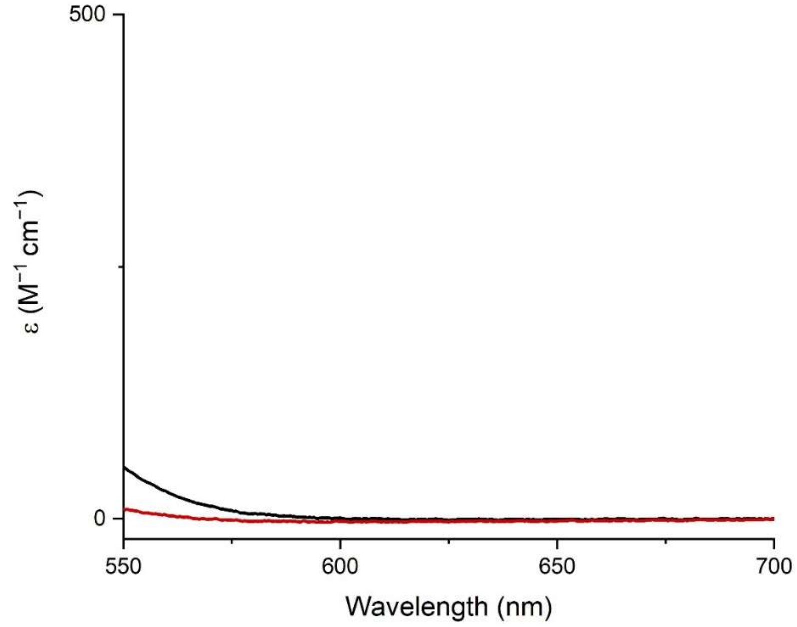

Figure S 35: Molar extinction coefficient of ***7-E*** *(black) and* ***7-Z*** *(red), section between 550 nm – 700 nm.*

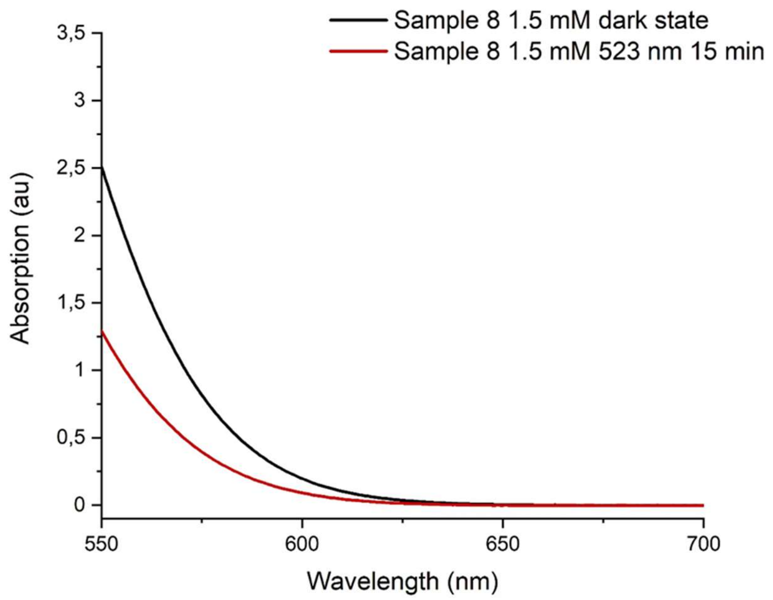

*Figure S 36: Absorbance spectrum of **8**. Raw data before calculation., y-axis: absorption (au), x-axis wavelength (nm).*

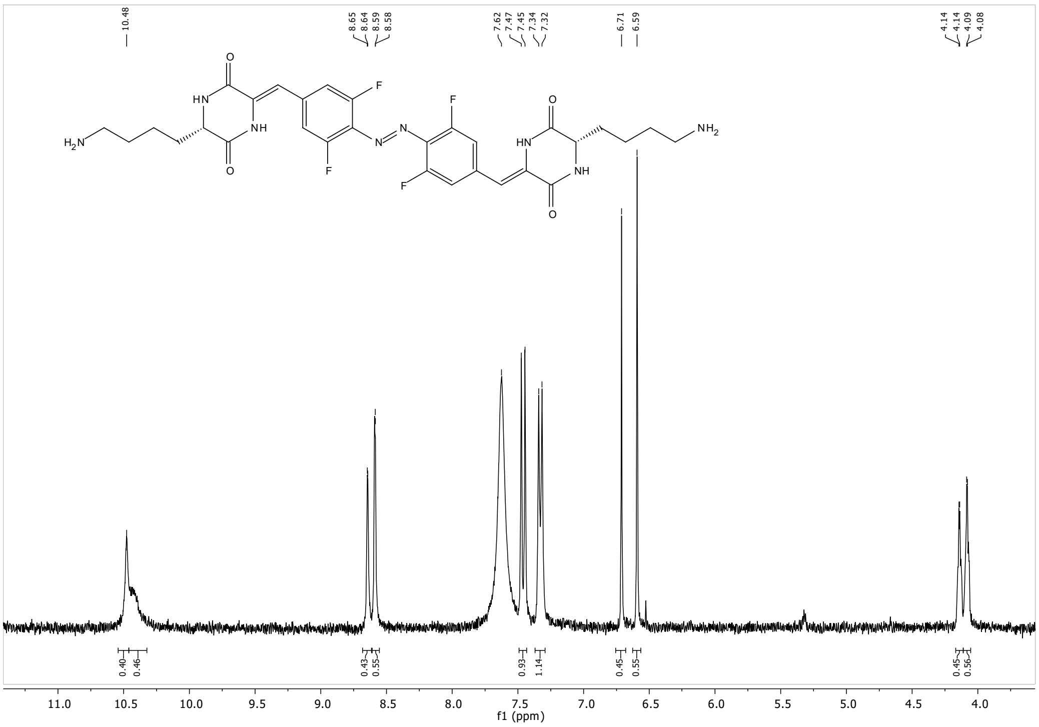

*Figure S 37: 1H-NMR-spectrum (400 MHz, 32 scans, DMSO-d6) of **8** (1.5 mM) after irradiation at 523 nm.*

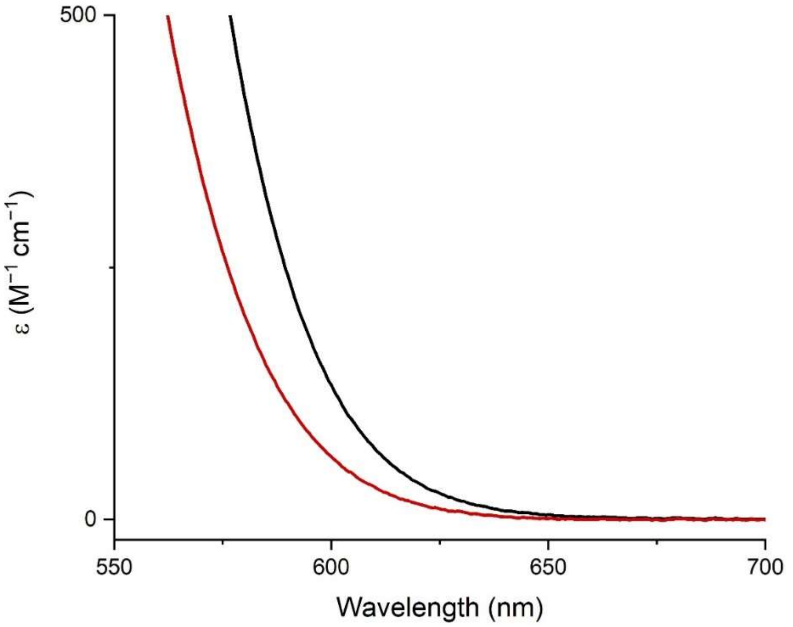

*Figure S 38: Molar extinction coefficient of **8-E** (black) and **8-Z** (red).*

Due to the high absorbance of compound **8** between 400 nm- 500 nm, a dilution to a final concentration of 100 µM was prepared to measure the range between 400 nm-700 nm.

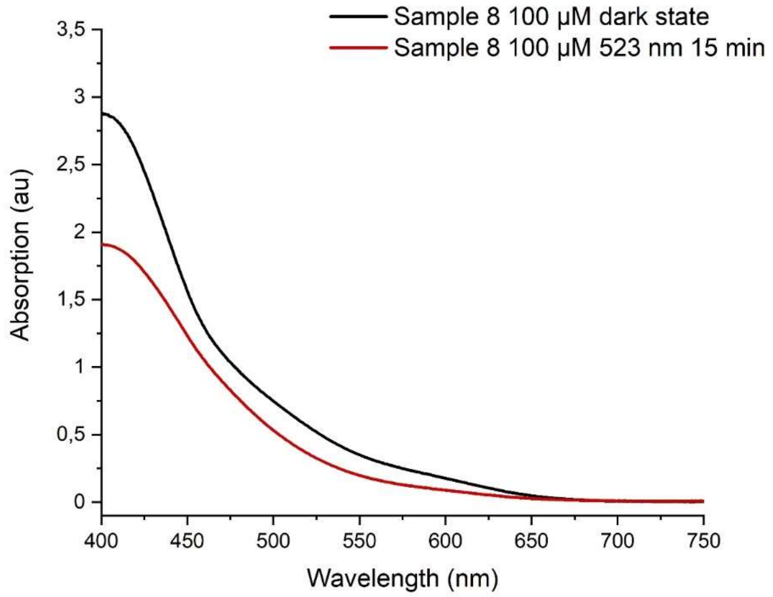

*Figure S 39: Absorbance spectrum of **8**. Raw data before calculation., y-axis: absorption (au), x-axis wavelength (nm).*

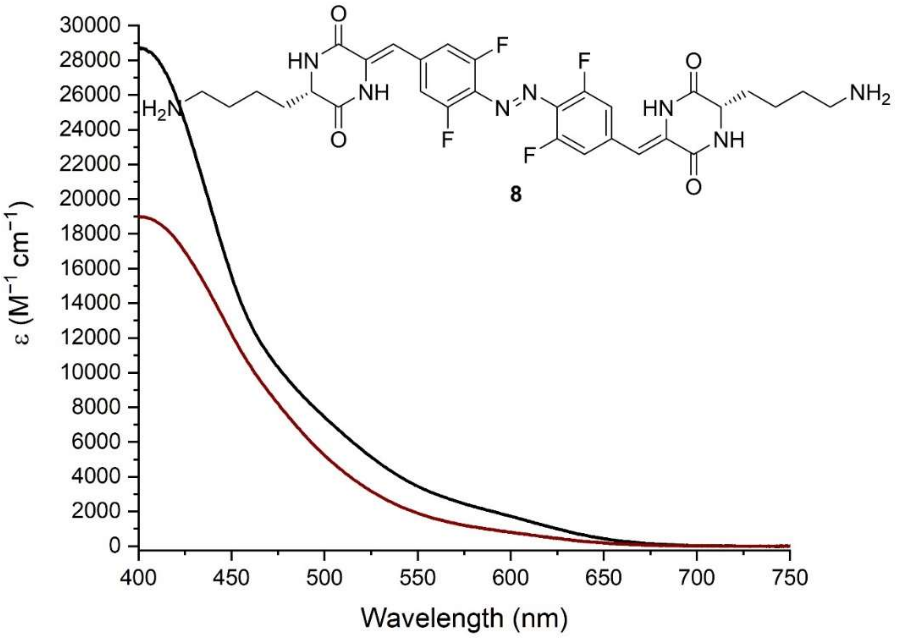

Figure S40. Molar extinction coefficient of **8-E** (black) and **8-Z**

7. Biological stability

For prospective applications, stability of the compound **8** under physiological conditions had to be investigated. Reduction of azobenzene derivatives with thiol groups to arylhydrazines in biological systems is considered the most serious limitation for their in vivo application as photoswitches. Therefore, we evaluated the stability of **8** in the reducing environment. A solution with final concentrations of 0.1 mM of **8**, 10 mM reduced glutathione and 5 mM TCEP was prepared in 0.1 M pH 7.4 DPBS (no magnesium, no calcium) with 5% DMSO. Stock solutions were prepared as follows: 2 mM compound **8** in DMSO and 0.25 M reduced glutathione in DPBS, the pH was adjusted to 7.4 with NaOH. The mixture was kept at 25 °C and HPLC was measured in 30 min intervals over a period of 12 h. For analysis the peak area ratio of **8** and reduced glutathione was calculated, assuming that the area of the reduced glutathione is constant (100-fold higher conc.).

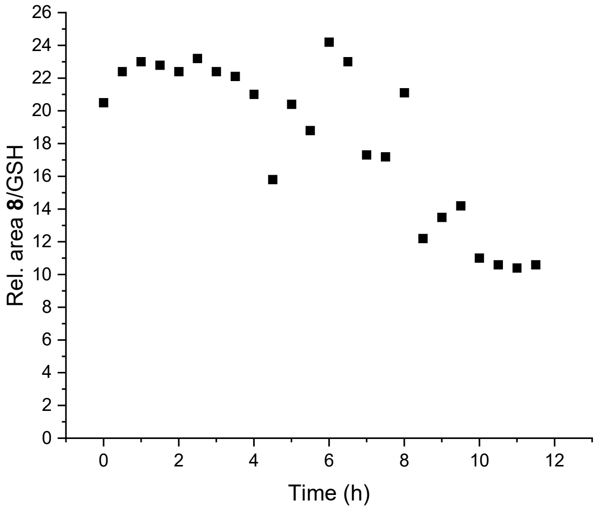

*Figure S 41: Biological stability of **8**. Within 10 hours, roughly half of the compound **8** has been degraded.*

8. Viscosity experiments

To a 1.5 mL-vial (crimp top, 12×32 mm) was added the photochromic material **8** as powder and 500 µL of the aqueous solution. This suspension was treated by ultrasonic waves followed by heating to 80 °C in a vial block. After equilibration at 80 °C for 5 min, the sample was heated to the boiling point by a heat gun. The hot solution became completely clear and upon cooling the viscosity increased.

*Table S 20: *melting point was determined in a single measurement.*

Composition of the solution (x mg + 500 µL H_2O)	Approx. Conc.	T_m °C	gelation time (room temp.)
10	2 wt%	55*	> 5 min

*Table S 21: *melting point was determined in a single measurement*

Composition of the solution (x mg + 500 µL 200 mM NaCl)	Approx. Conc.	T_m °C	gelation time (room temp.)
10	2 wt%	>99*	> 5 min
7.5	1.5 wt%	89 ± 1	> 5 min
5	1 wt%	93 ± 5	> 5 min

*Table S 22: *melting point was determined in a single measurement*

Composition of the solution (x mg + 500 µL Ringer's solution)	Approx. Conc.	T_m °C	gelation time (room temp.)
10	2 wt%	83*	> 5 min
7.5	1.5 wt%	98*	> 5 min
5	1 wt%	84 ± 14	> 5 min
4	0.8 wt%	-	> 20 min
2.5	0.5 wt%	-	Not stable

9. Microscopy images

For the Transmission Electron Microscopy images, a sample of gel-like material containing 1 wt% of **8** was prepared with Ringer`s solution with additional 1% lead citrate solution (25 mg lead citrate in 0.1 M aq. NaOH) as described in the section 5. “Viscosity experiments”. After cooling, the sample was equilibrated overnight at room temperature. Some samples were irradiated to confirm photomodulation of the material. Sample A was dark adapted, B was irradiated at 660 nm for 50 min and C was irradiated at 660 nm for 50 min then regenerated by boiling as in the initial formation of the material. The resulting viscous solutions were diluted 1:10 with diH_2O and added as small droplet to carbon-coated copper grids (400 mesh). The dilution of sample B was irradiated at 660 nm for additional 50 min to obtain sample D. The supernatant was removed carefully with a lint-free sheet and the grid was dried under atmospheric pressure. Examination was carried out on a Philips CM200 FEG transmission electron microscope, operated at 200 kV accelerating voltage.

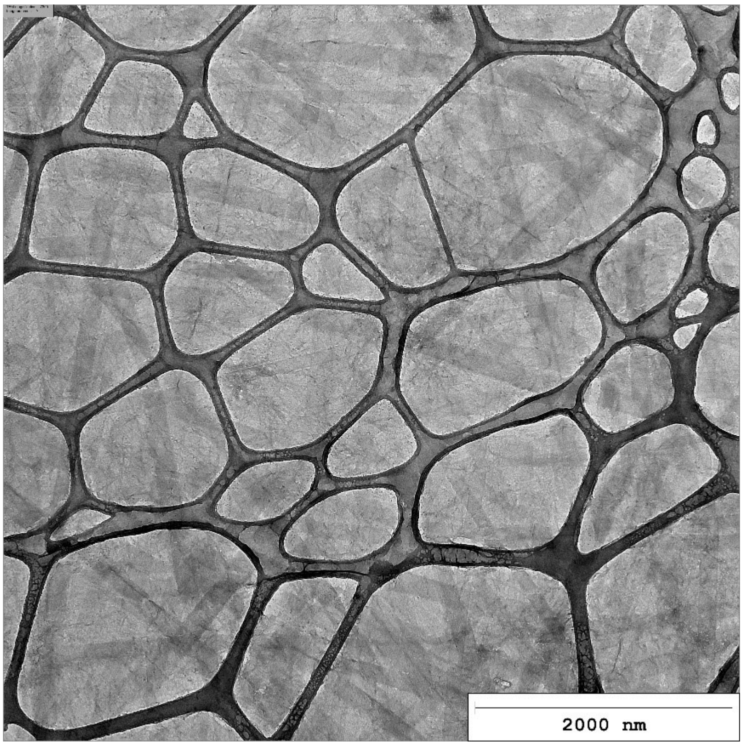

Figure S 42: TEM sample A.

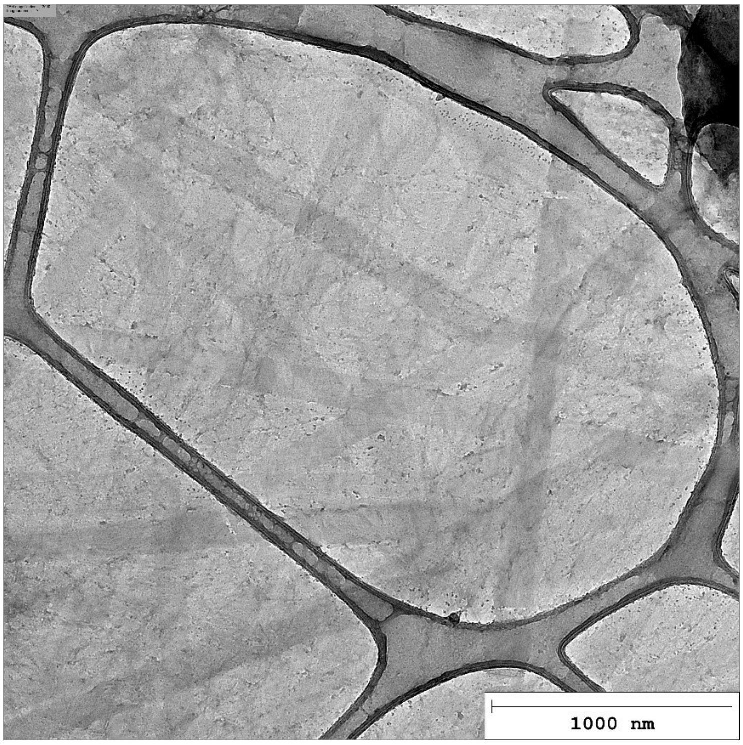

Figure S 43: TEM sample A.

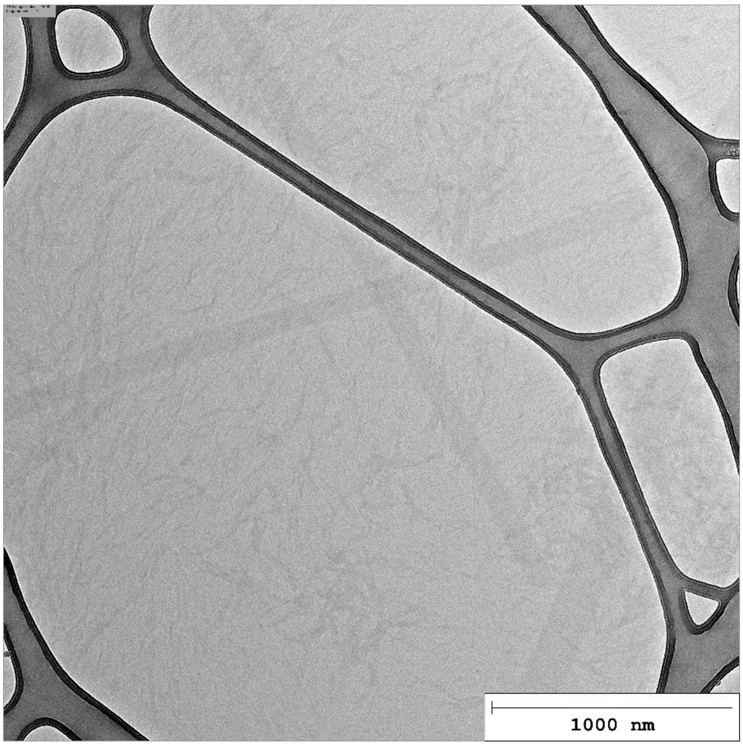

Figure S 44: TEM sample B.

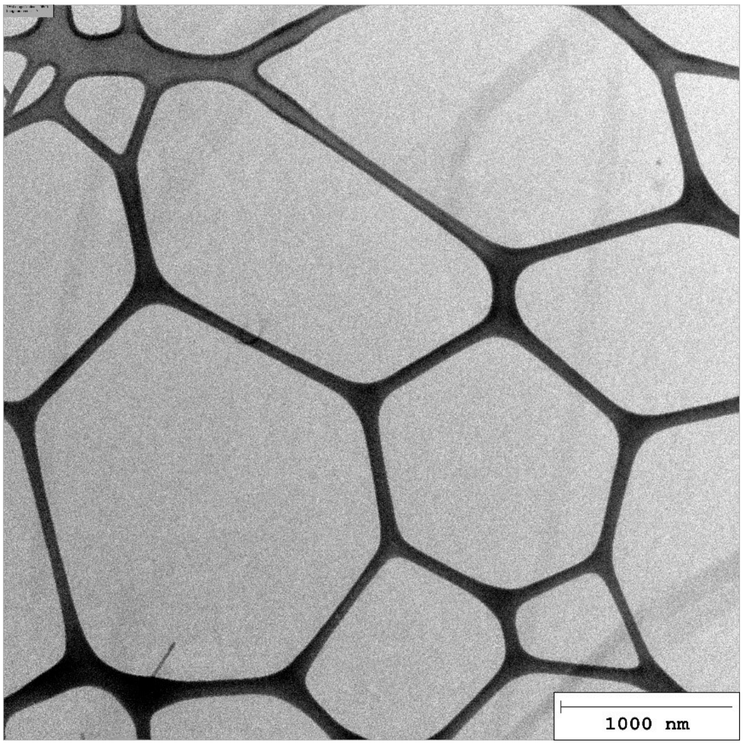

Figure S 45 TEM: sample B.

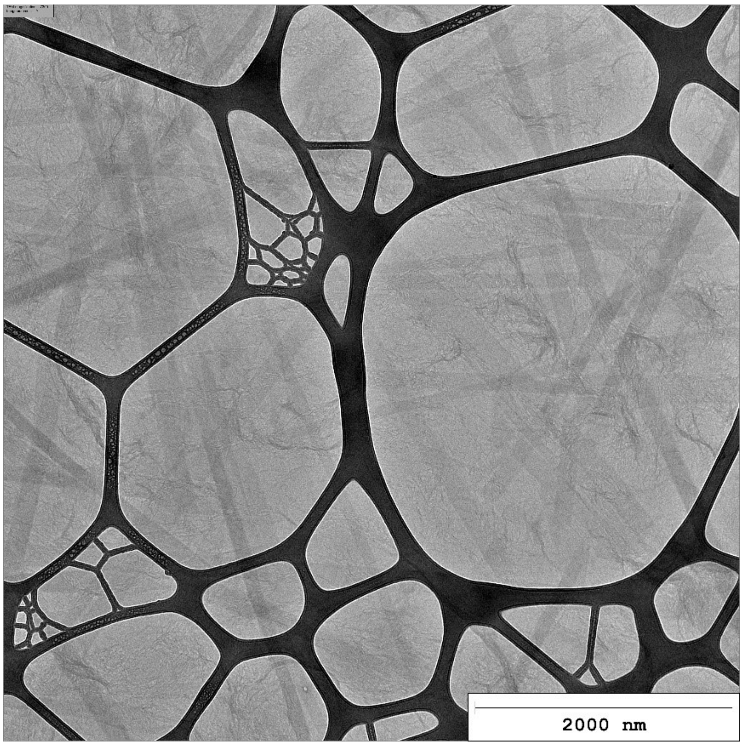

Figure S 46: TEM sample C.

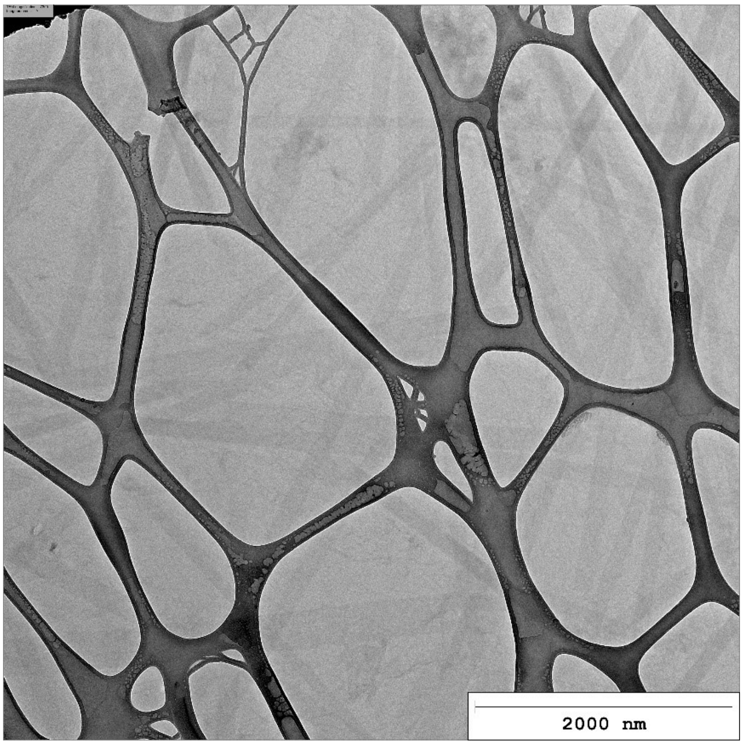

Figure S 47: TEM sample C.

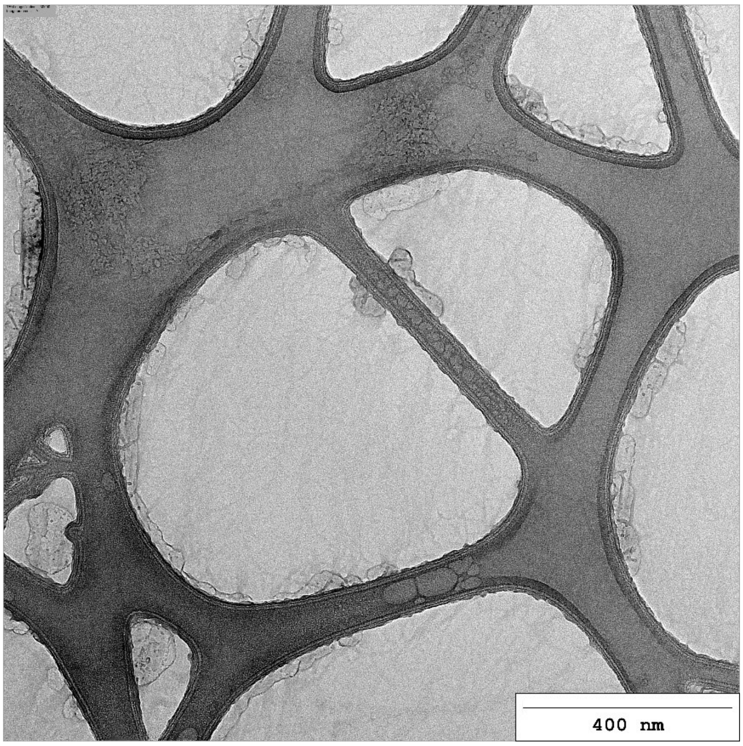

Figure S 48: TEM sample D.

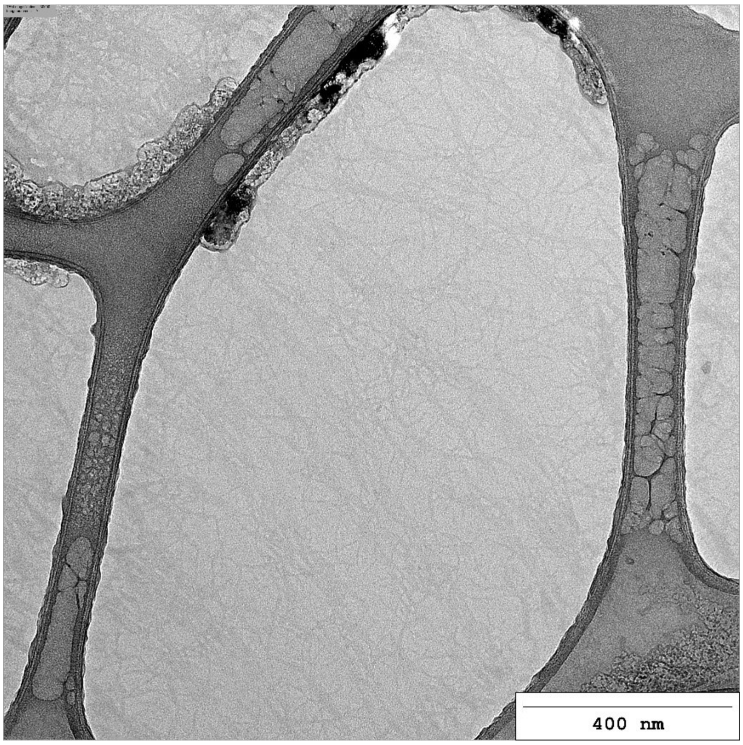

Figure S 49: TEM sample D.

For the Scanning Electron Microscopy images, a 1 wt% viscous solution sample of **8** was prepared with Ringer`s solution (sample **E**) and a 2 wt% viscous solution sample of **8** was prepared with diH_2O (sample **F**). The resulting material was freeze dried by lyophilization and then coated with a thin layer of platinum.

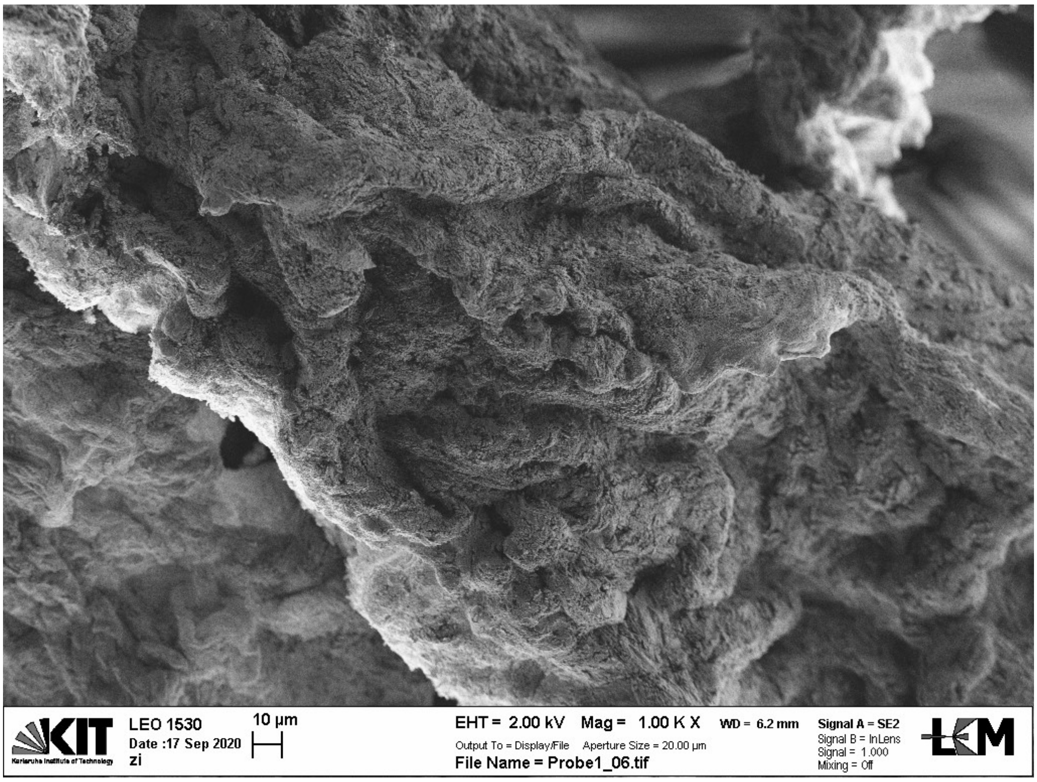

Figure S 50: REM sample E.

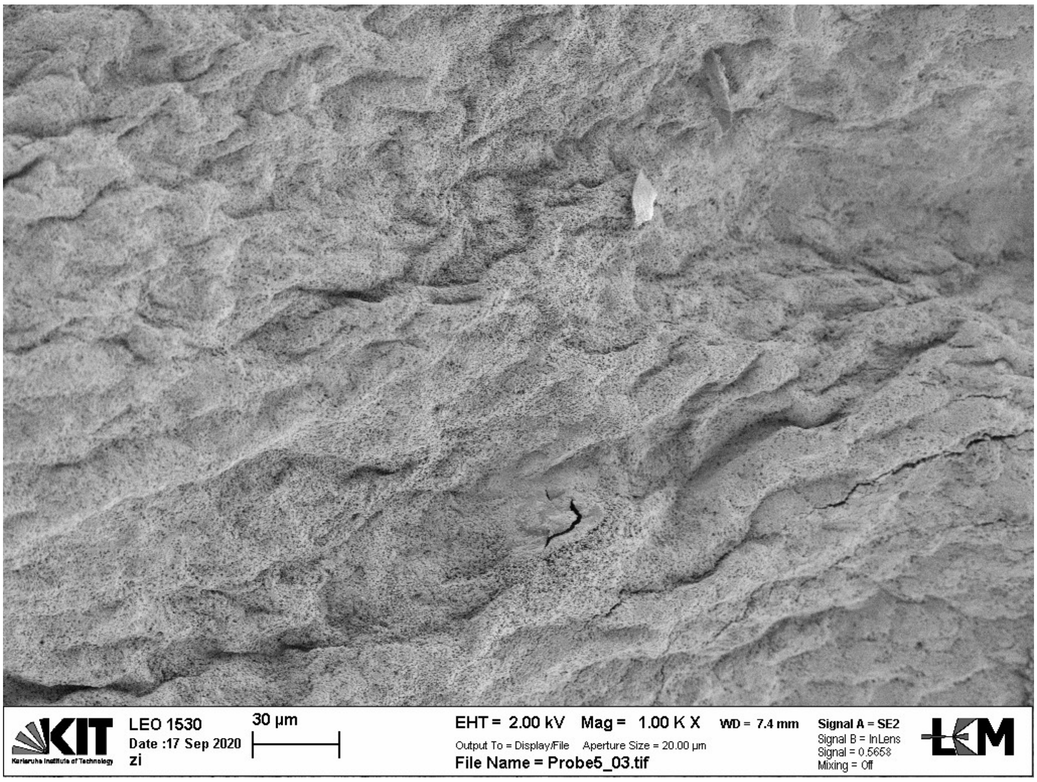

Figure S 51: REM sample E.

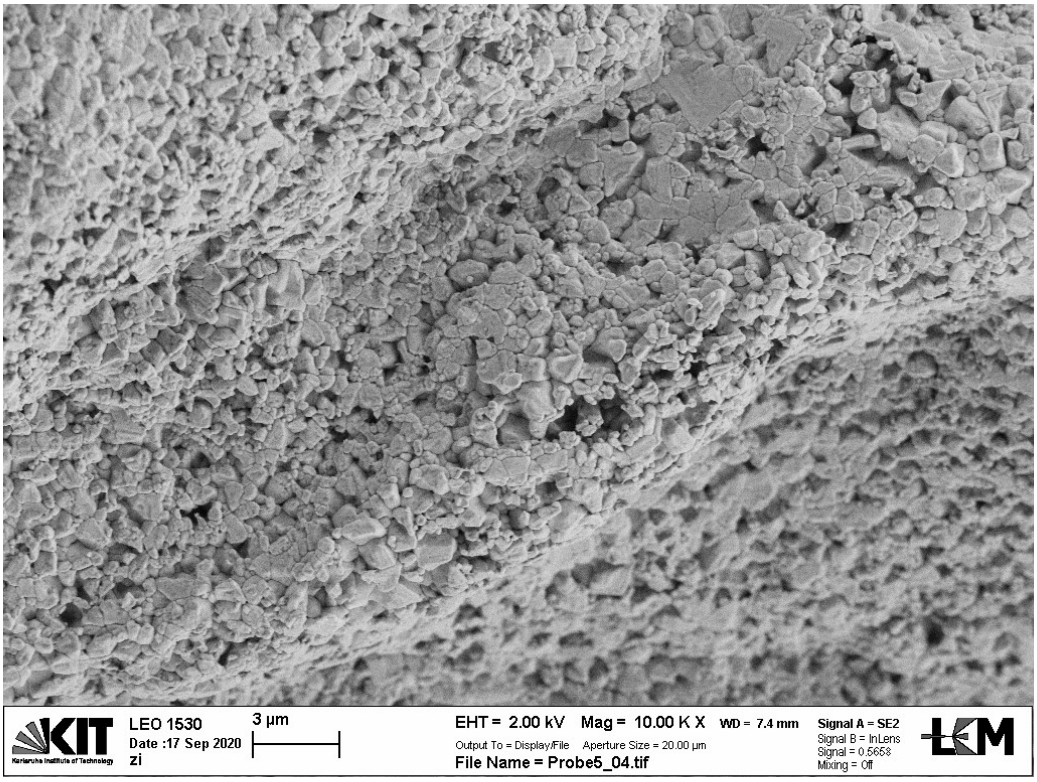

Figure S 52: REM sample E.

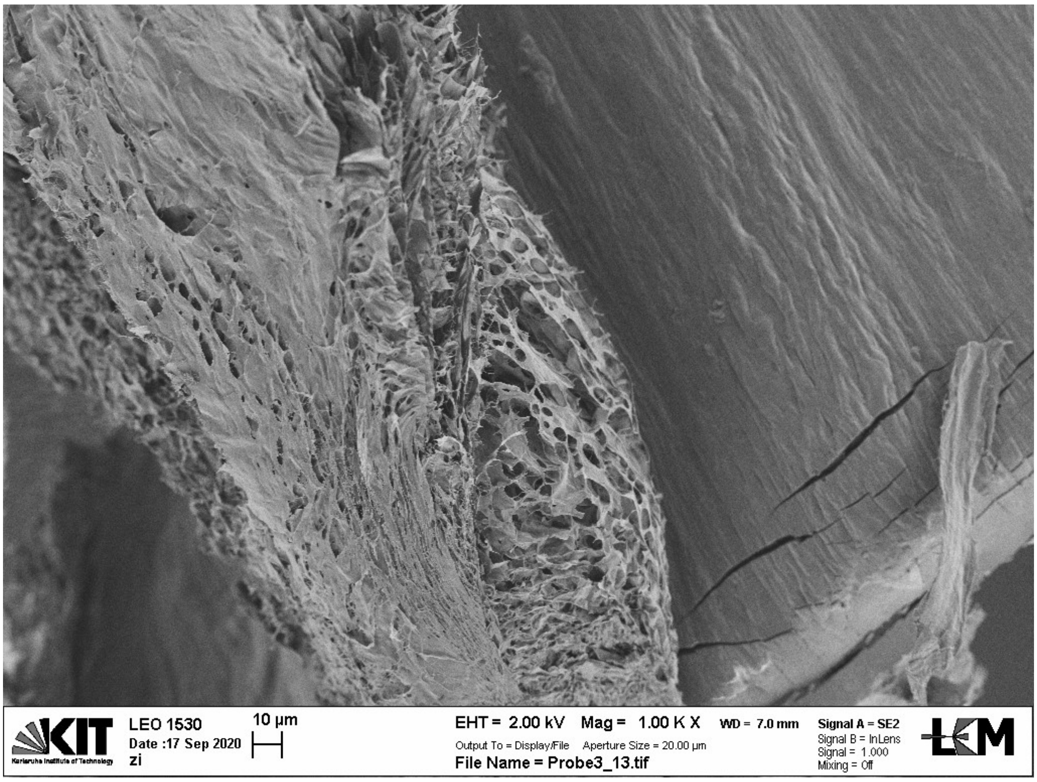

Figure S 53: REM sample F.

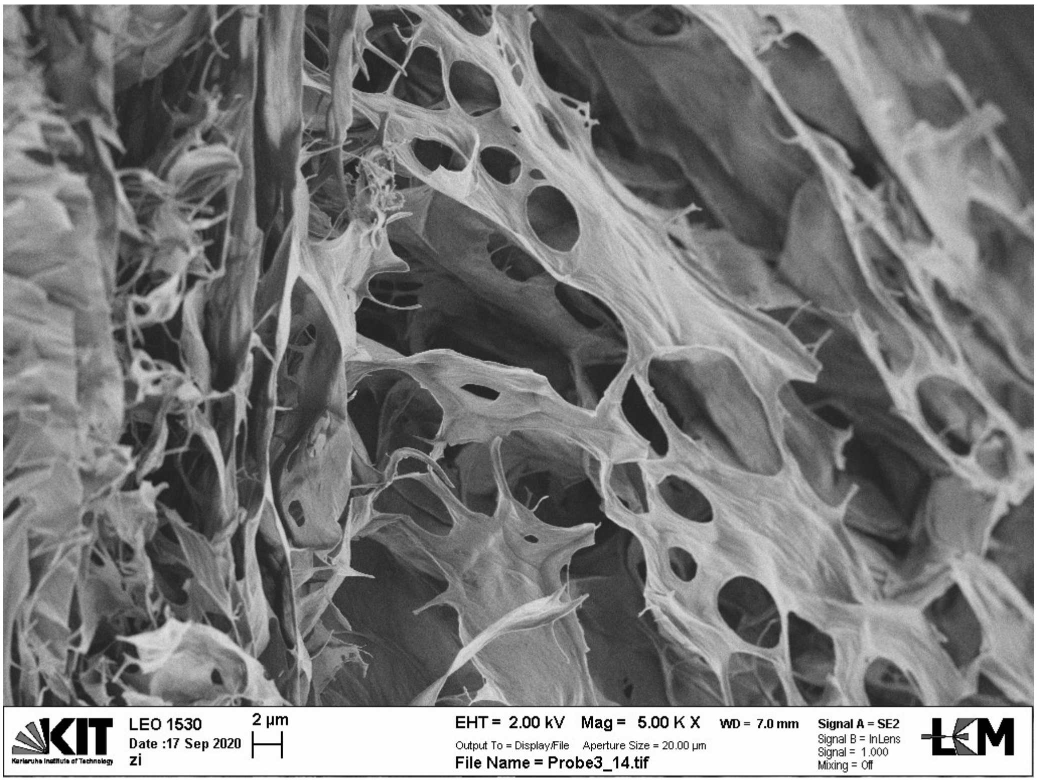

Figure S 54: REM sample F.

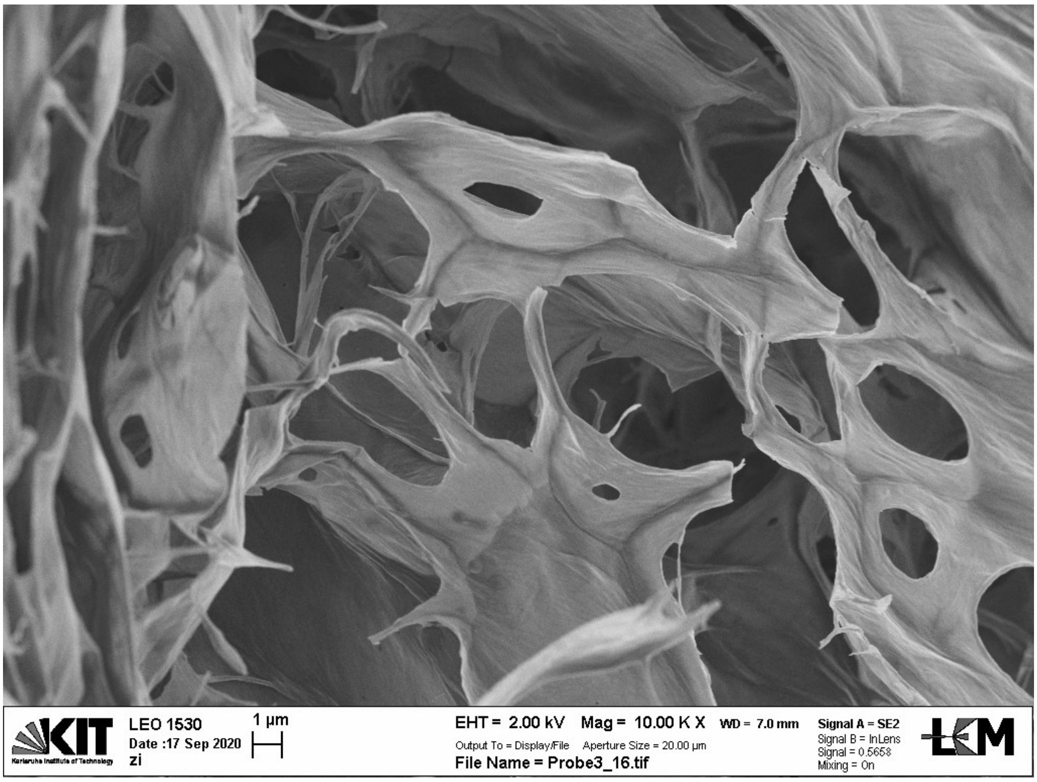

Figure S 55: REM sample F.

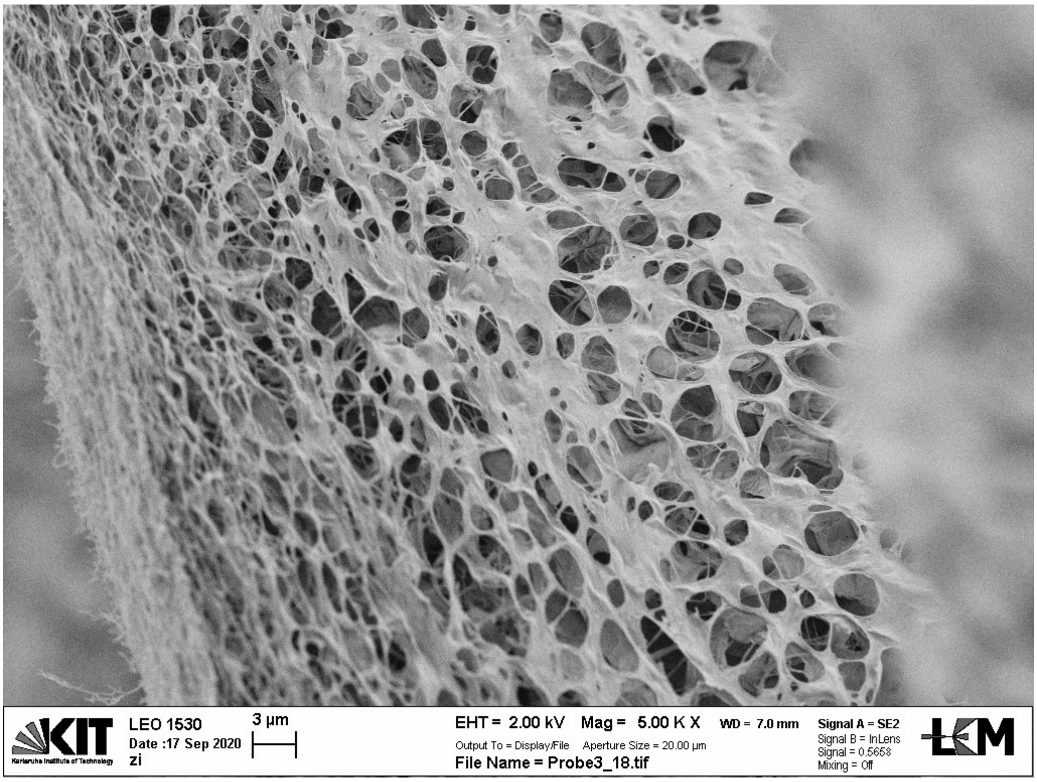

Figure S 56: REM sample F.

10. Rheology and NMR relaxometry of 8

Rheology

Rheological properties were investigated via oscillatory shear experiments on the strain-controlled rotational rheometer Ares G2 (TA Instruments, Eschborn, Germany) with a parallel-plate geometry (diameter: 30 mm). The sample of material 8 with a concentration of 20 g/L (2 wt%) and a volume of 3 mL was prepared according to the procedure described in part 5 of the supporting information in a 10 mL-vial and poured on to the lower plate. The gap was set to 1 mm and the temperature controlled to 25 ± 0.1 °C by a Peltier element (Advanced Peltier System, TA Instruments). An oscillatory strain-sweep with a constant frequency of 1 Hz and a varying strain of γ0 = 0.01-1000 % was used to determine the linear viscoelastic regime (LVE) of the samples. A strain of 0.1 % was fixed for the subsequent frequency sweeps where the frequency was varied from 0.03 to 100 Hz.

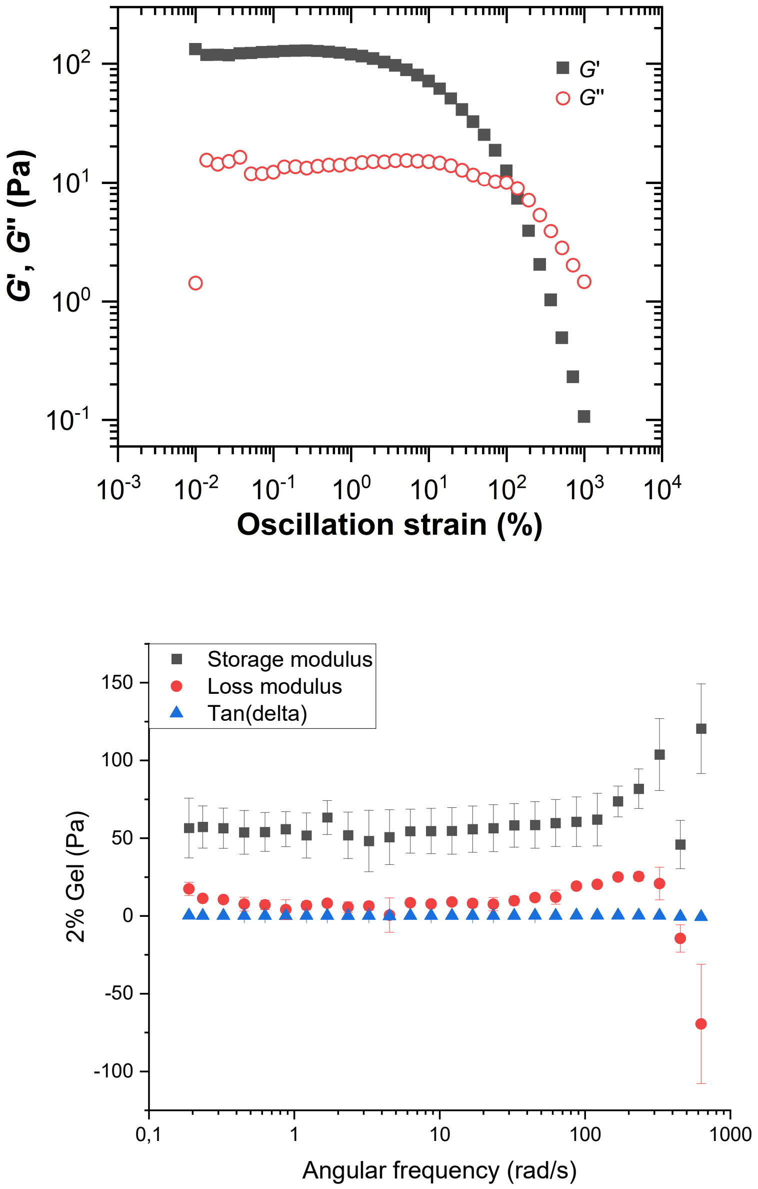

*Figure S 57: Rheological measurements of the viscous material composed of 2 wt% compound **8** in water. Strain sweep experiment (top); frequency sweep experiment (bottom).*

^{1}H NMR T_2 relaxation

The ^{1}H-NMR T_2 relaxation measurements were performed on a bench-top 20 MHz minispec (Bruker, ND series). A flat NMR tube (diameter: 10 mm) was filled with 50 mg of 8 and 500 µL D_2O (treated with ultrasonic for 1 h, then heated to boiling) to a maximum height of 10 mm, ensuring the highest homogeneity of the B1 magnetic field. The sample temperature was controlled to 39.5 °C by a BVT3000 unit (Bruker).

To acquire a full relaxation curve over several orders of magnitude (10^{-3} – 10^{3} ms), a combination of a MSE (magic sandwich echo) and two CPMG (Carr-Purcell-Meiboom-Gill) pulse sequences have been used.[27,28] To avoid spin locking effects a XY16 phase cycle was used for the CPMG.[29] The data points were acquired over 128 scans with a recycle delay of 800 ms. The MSE recorded the first 100 μs, followed by two CPMGs with a pulse separation of 0.04 ms and 1 ms. The full relaxation curve is shown in **Figure S58**. The CPMG refocuses the magnetization of the more mobile components, e.g. solvent as HDO, and is evaluated with a biexponential fitting function (CPMG fit) to determine the contribution of mobile components. The distinct decay of the MSE clearly indicates a highly crystalline component in the sample.

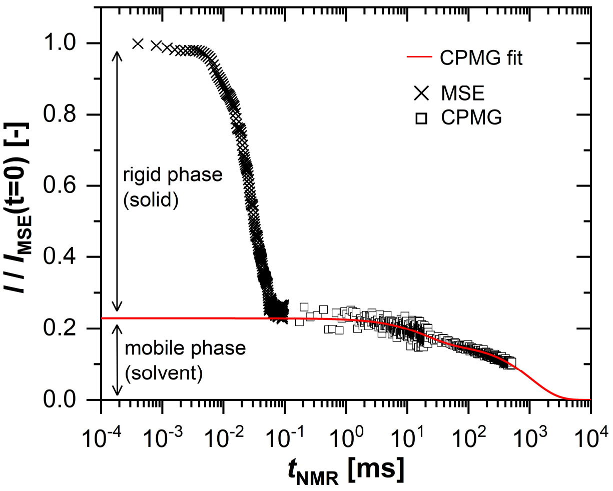

*Figure S 58: NMR relaxometry of the viscous material composed of the compound **8** in D_2O.*

11. NMR spectra of the synthesized compounds

(*E*)-1,2-bis(2,6-difluorophenyl)diazene (TFAB)

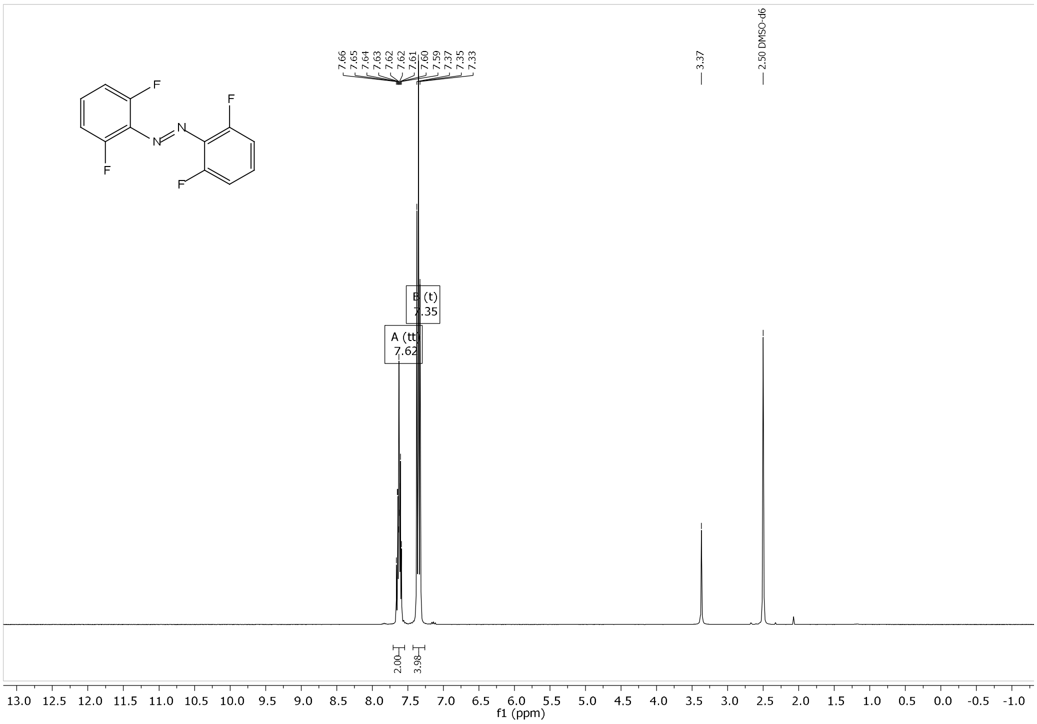

Figure S 59: 1H-NMR-spectrum (400 MHz, DMSO-d6) of TFAB.

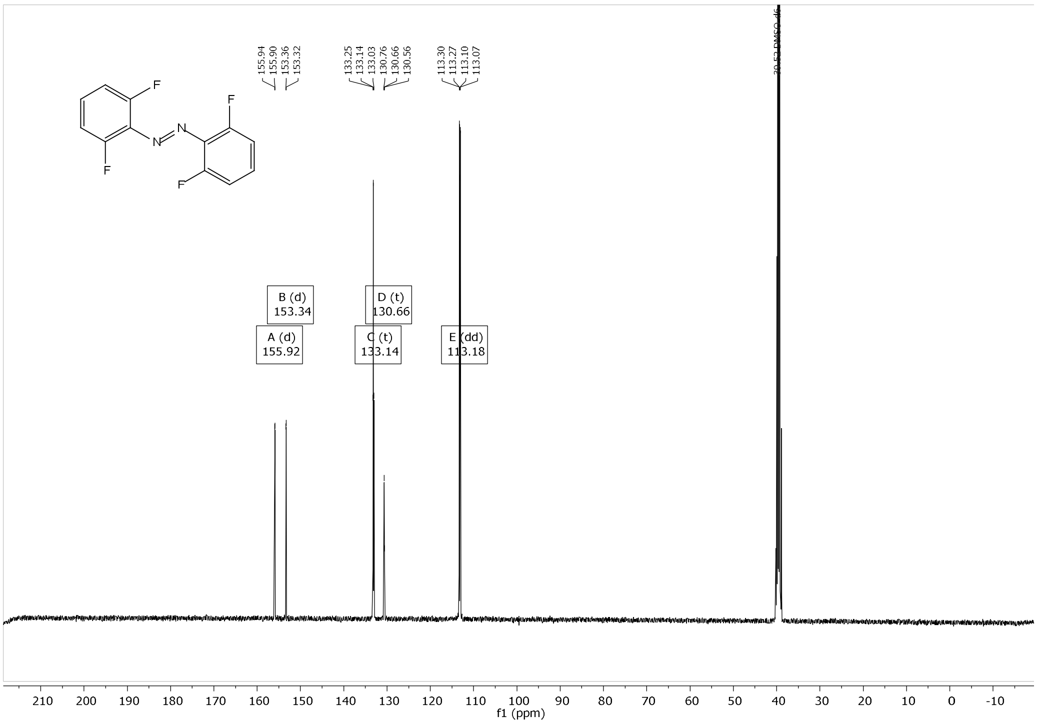

Figure S 60: 13C-NMR-spectrum (101 MHz, DMSO-d6) of TFAB.

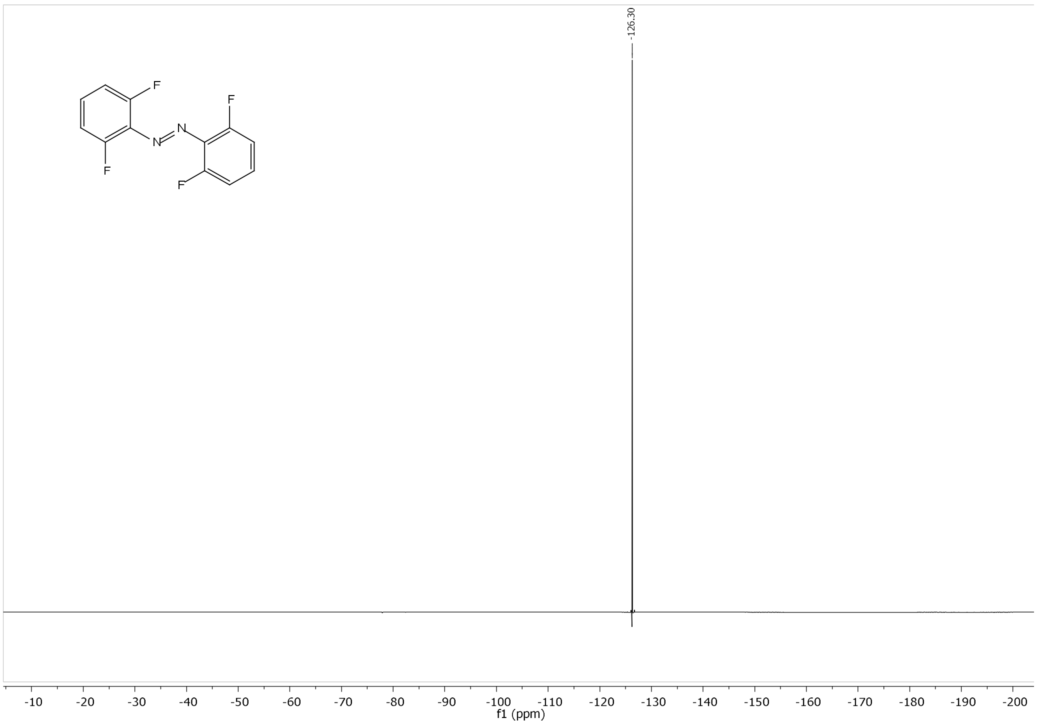

Figure S 61: 19F-NMR-spectrum (376 MHz, DMSO) of TFAB.

*(E)-(*4-((2,6-difluorophenyl)diazenyl)-3,5-difluorophenyl)methanol (2)

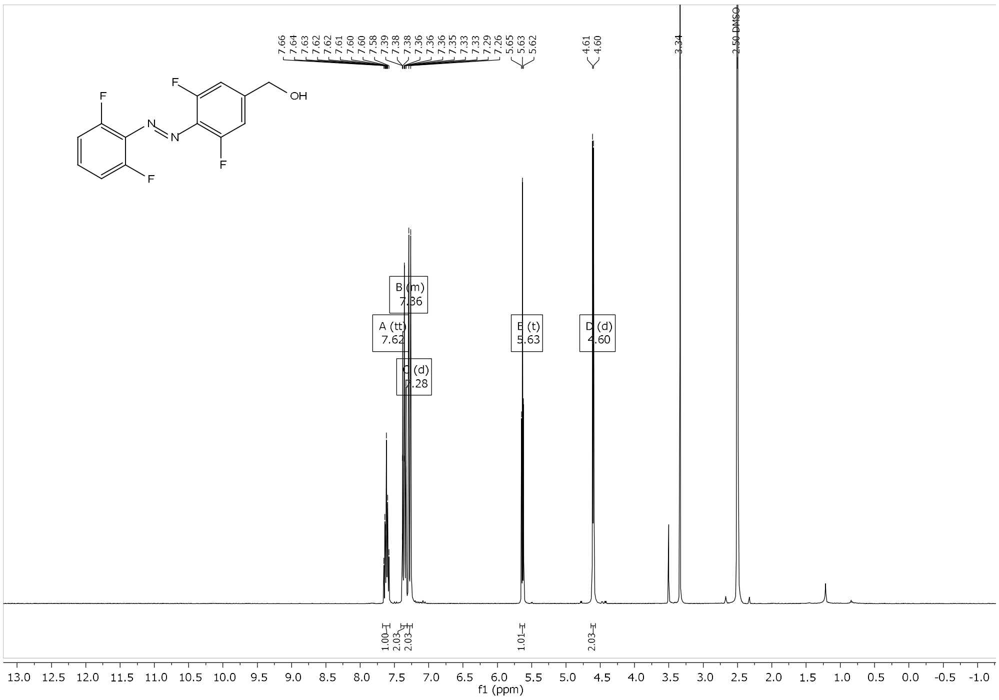

Figure S 62: 1H-NMR-spectrum (300 MHz, DMSO-d6) of the compound 2.

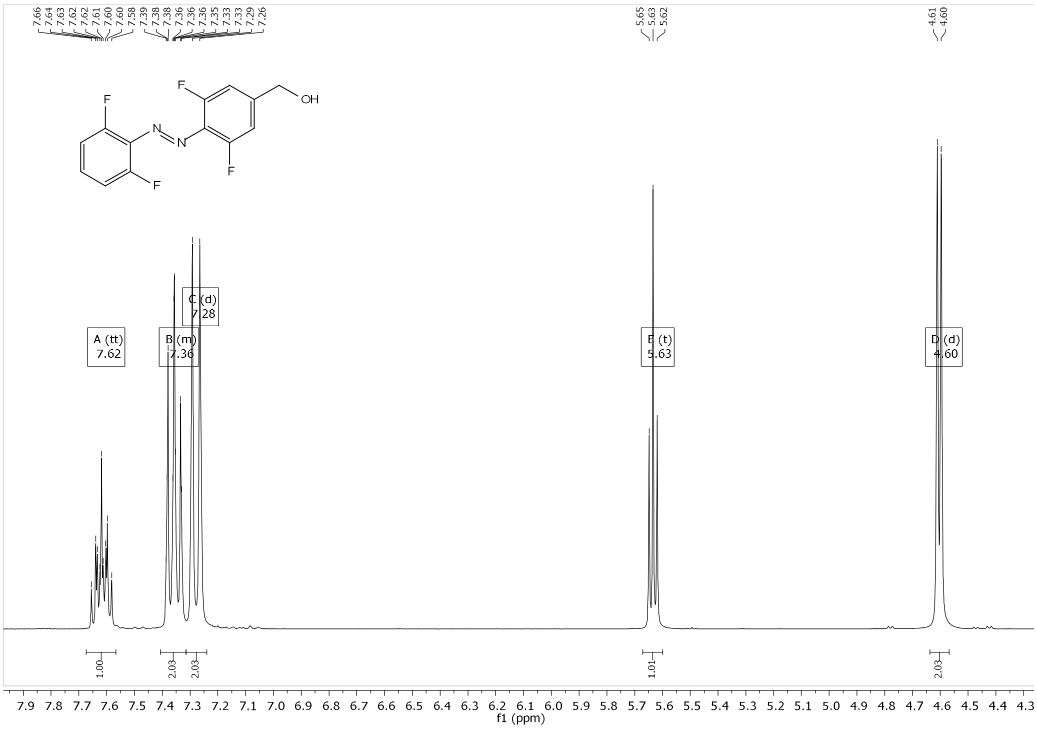

*Figure S 63: ^{1}H-NMR-spectrum (300 MHz, DMSO-d_6) of the compound **2**.*

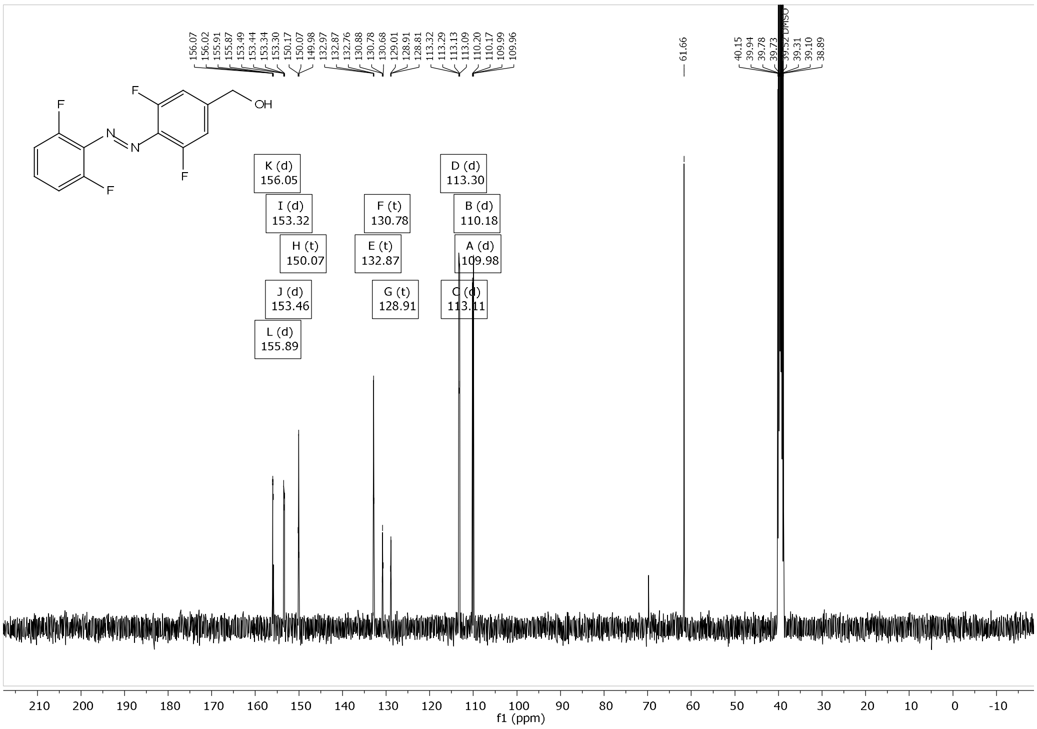

Figure S 64: 13C-NMR-spectrum (101 MHz, DMSO-d6) of the compound 2.

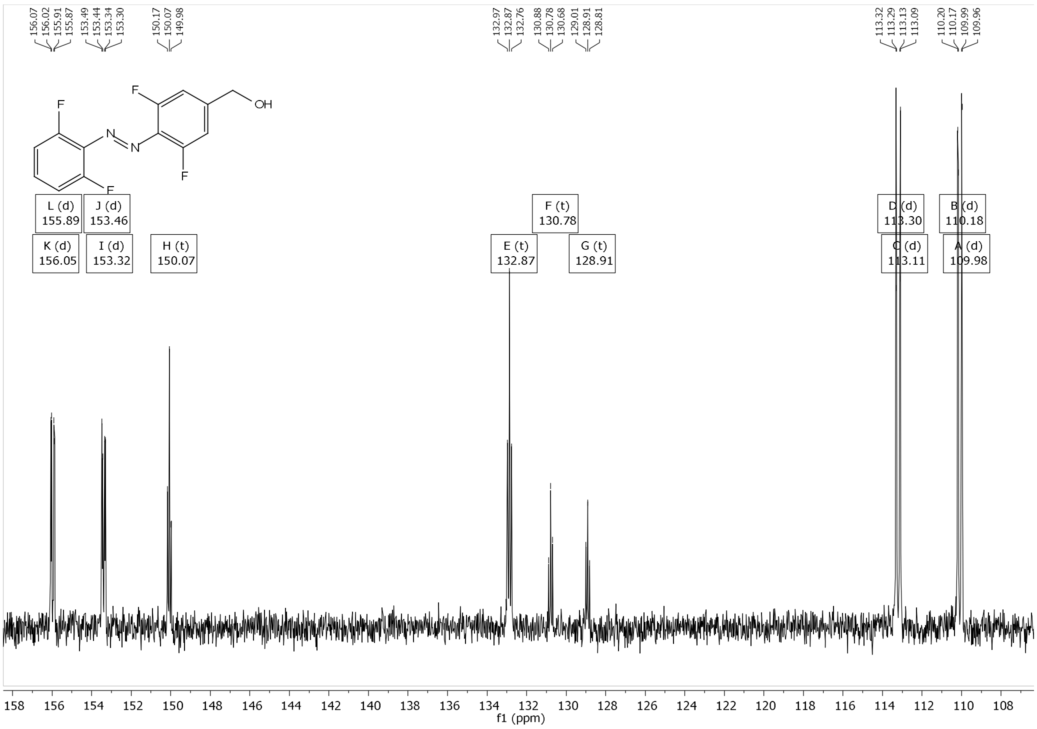

*Figure S 65: ^{13}C-NMR-spectrum (101 MHz, DMSO-d_6) of the compound **2**.*

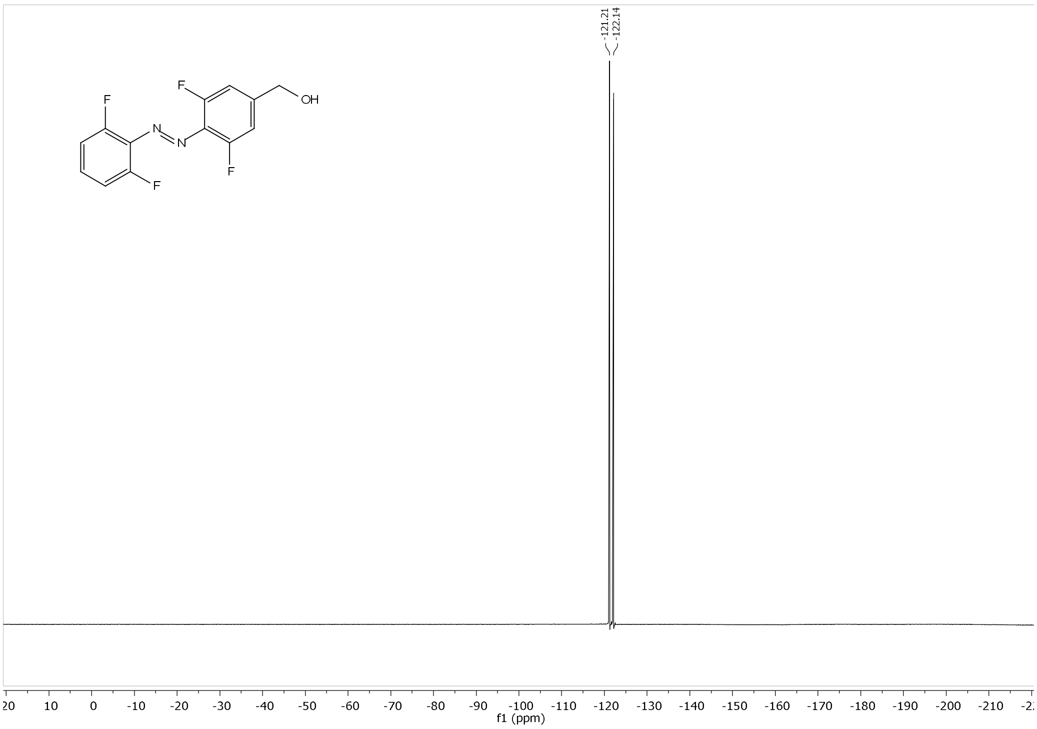

*Figure S 66: ^{19}F-NMR-spectrum (376 MHz, DMSO-d$_6$) of the compound **2**.*

(*E*)-(diazene-1,2-diylbis(3,5-difluoro-4,1-phenylene))dimethanol (3)

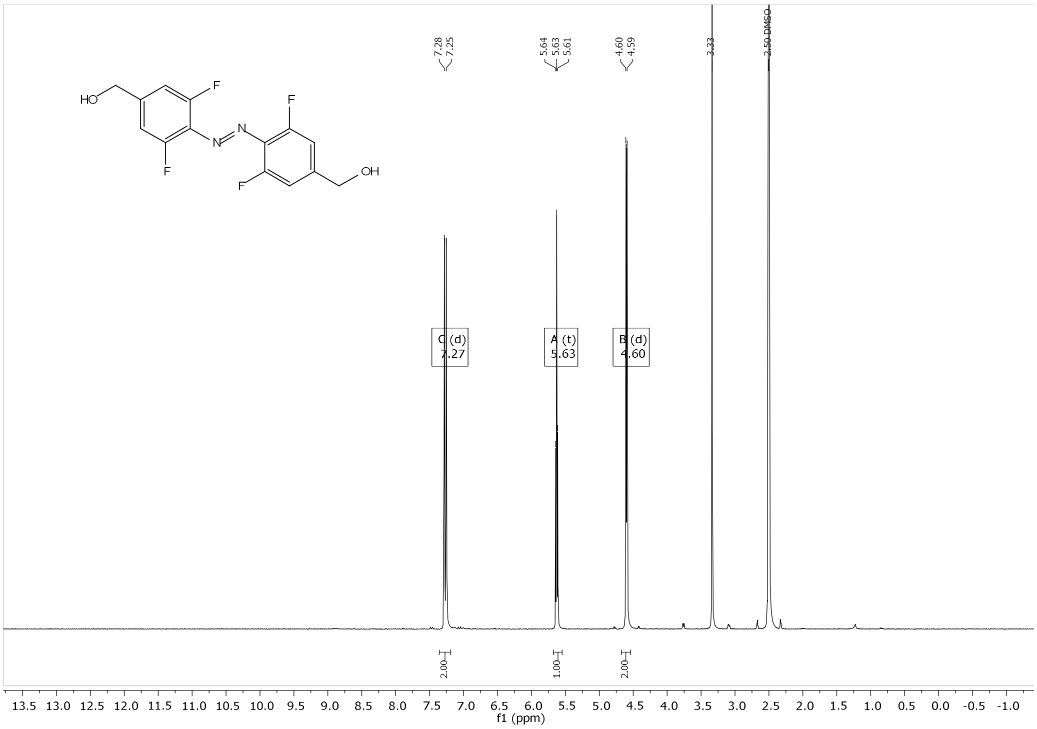

Figure S 67: 1H-NMR-spectrum (400 MHz, DMSO-d6) of the compound 3.

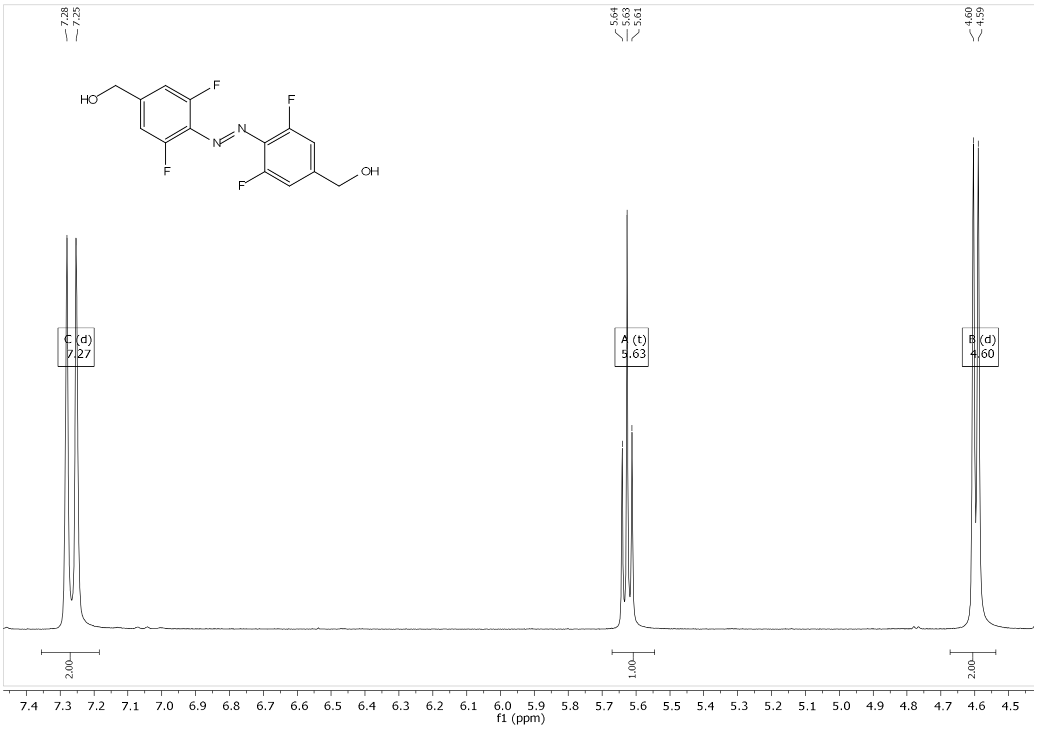

Figure S 68: 1H-NMR-spectrum (400 MHz, DMSO-d6) of the compound 3.

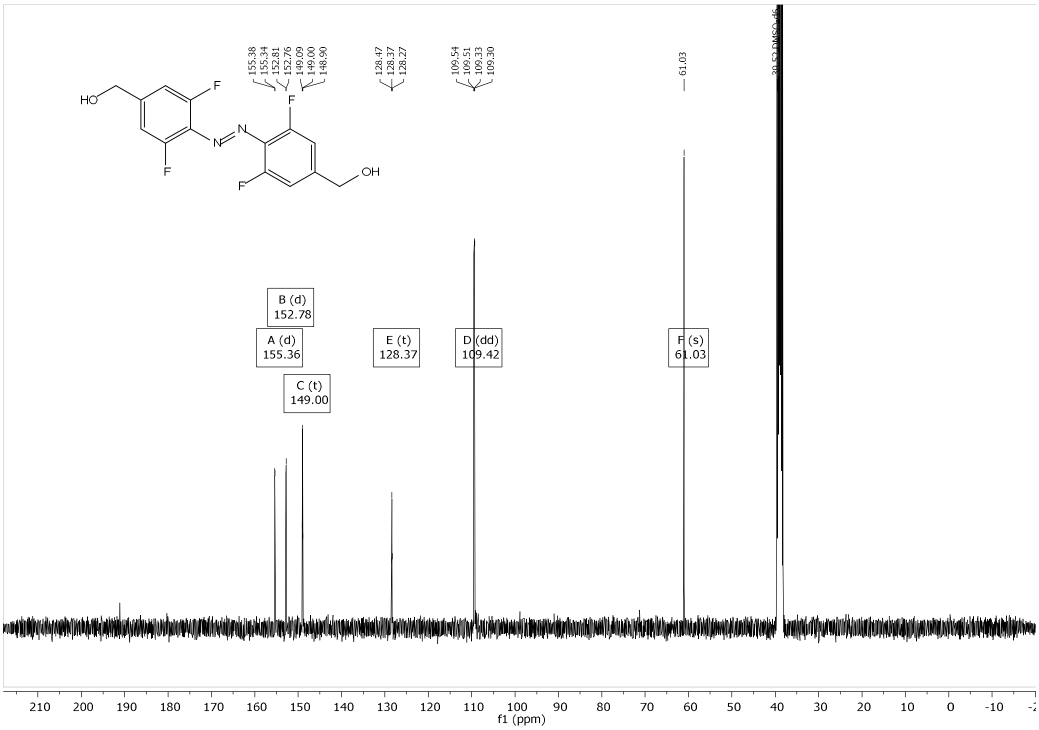

Figure S 69: 13C-NMR-spectrum (101 MHz, DMSO-d6) of the compound 3.

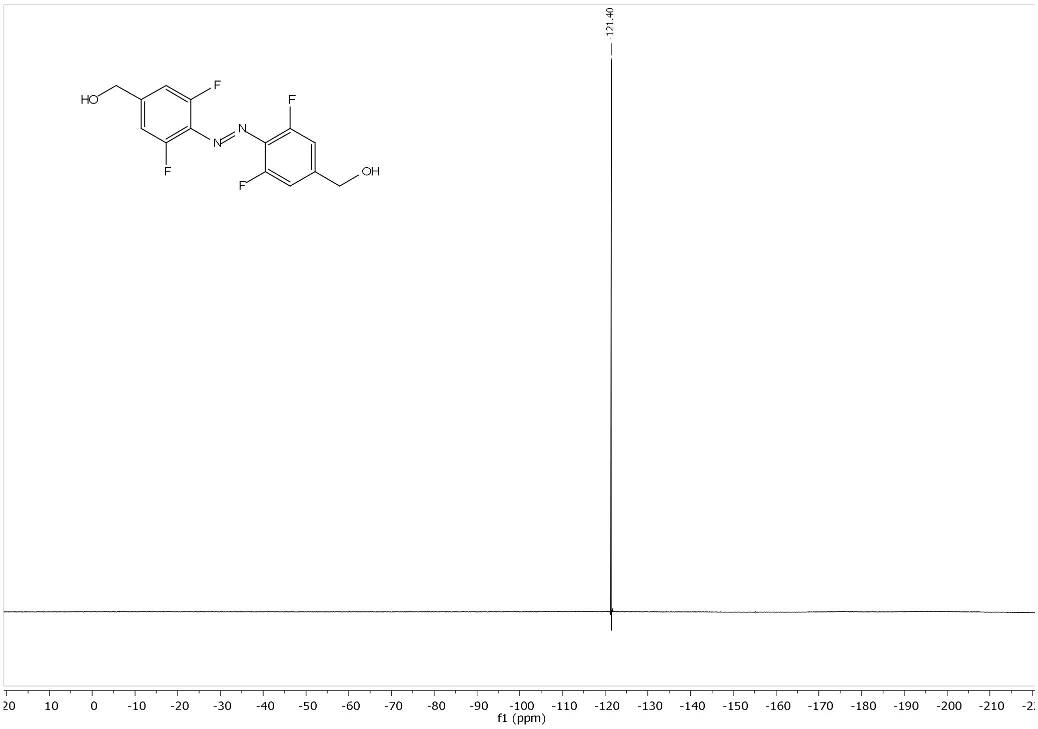

Figure S 70: ^{19}F-NMR-spectrum (376 MHz, DMSO-d_6) of the compound **3**.

(*E*)-4-(2,6-difluorophenyl)diazenyl-3,5-difluorobenzaldehyde (4)

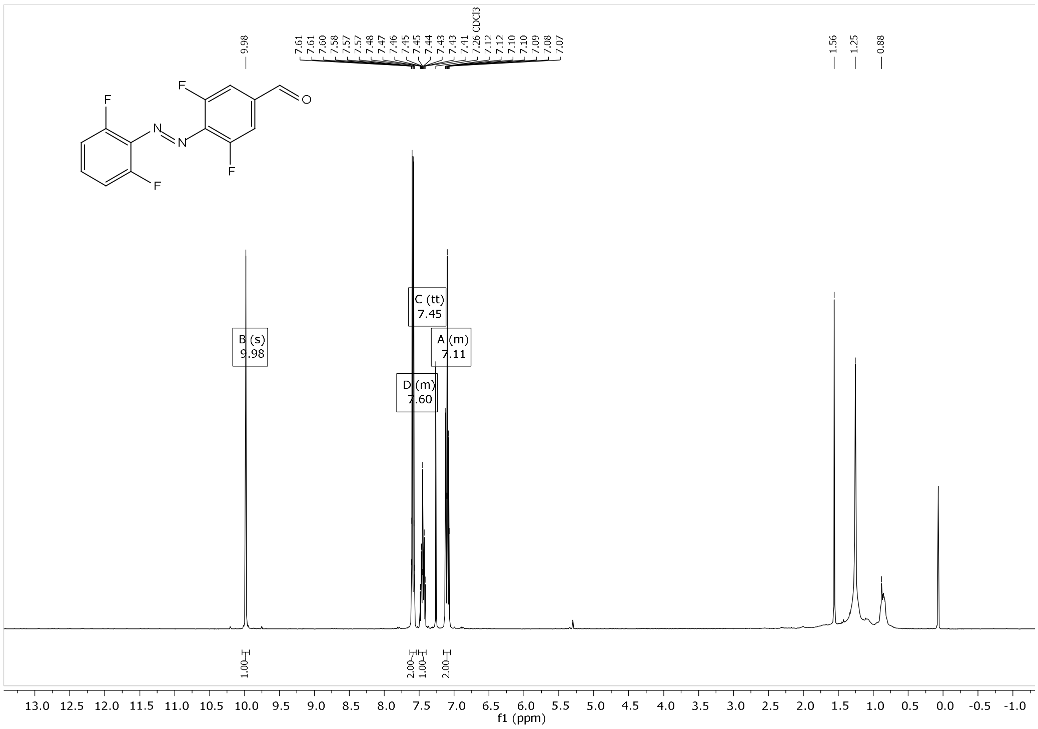

Figure S 71: 1H-NMR-spectrum (400 MHz, CDCl3) of the compound 4.

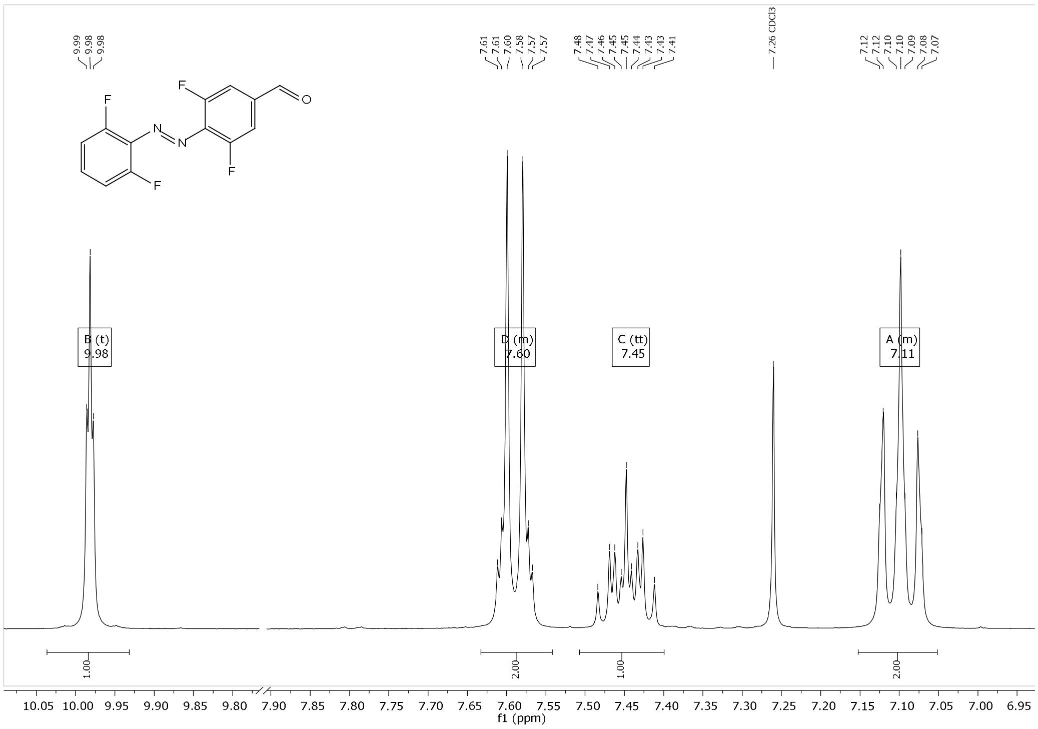

Figure S 72: 1H-NMR-spectrum (400 MHz, CDCl3) of the compound 4.

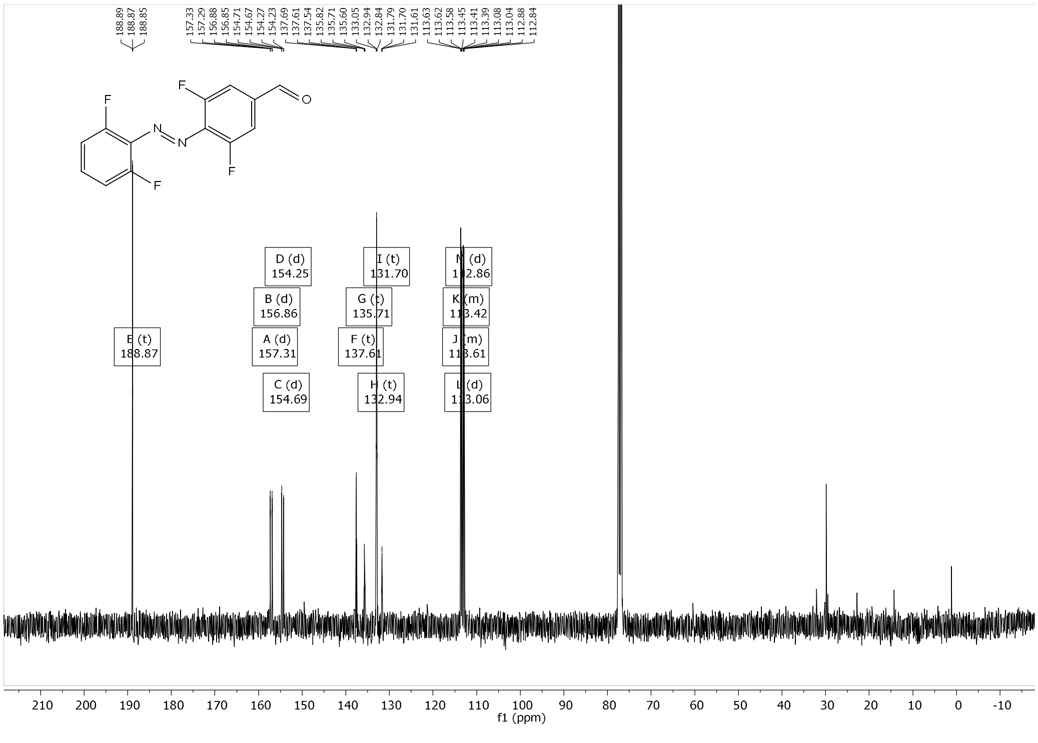

Figure S 73: 13C-NMR-spectrum (101 MHz, CDCl3) of the compound 4.

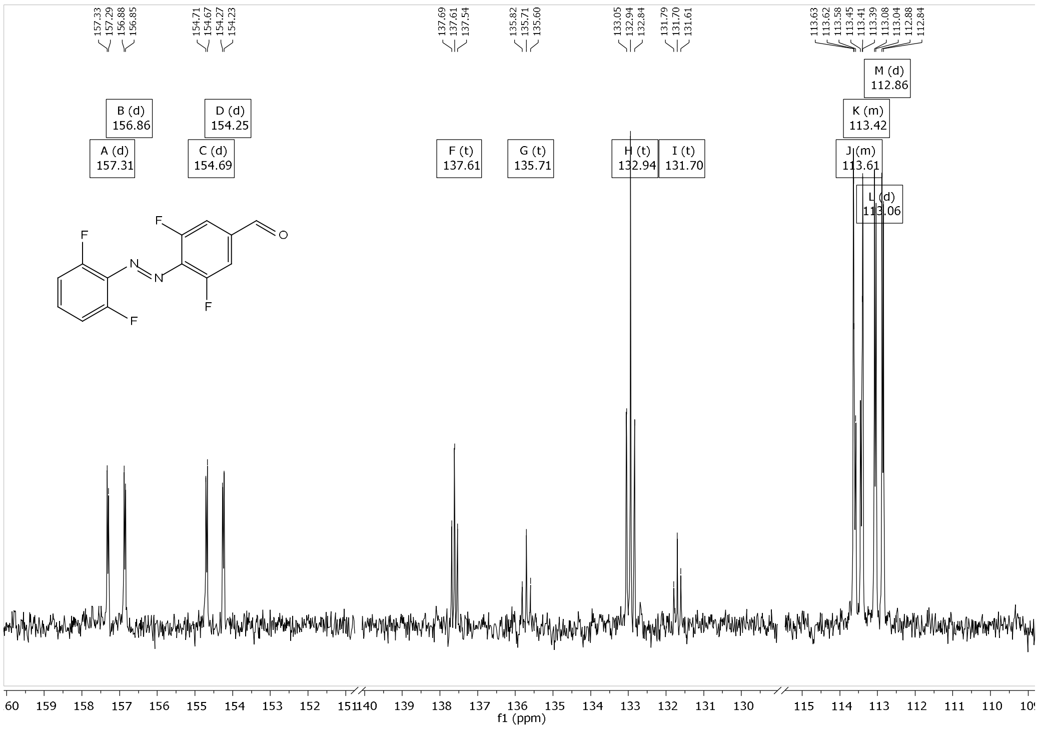

Figure S 74: 13C-NMR-spectrum (101 MHz, CDCl3) of the compound 4.

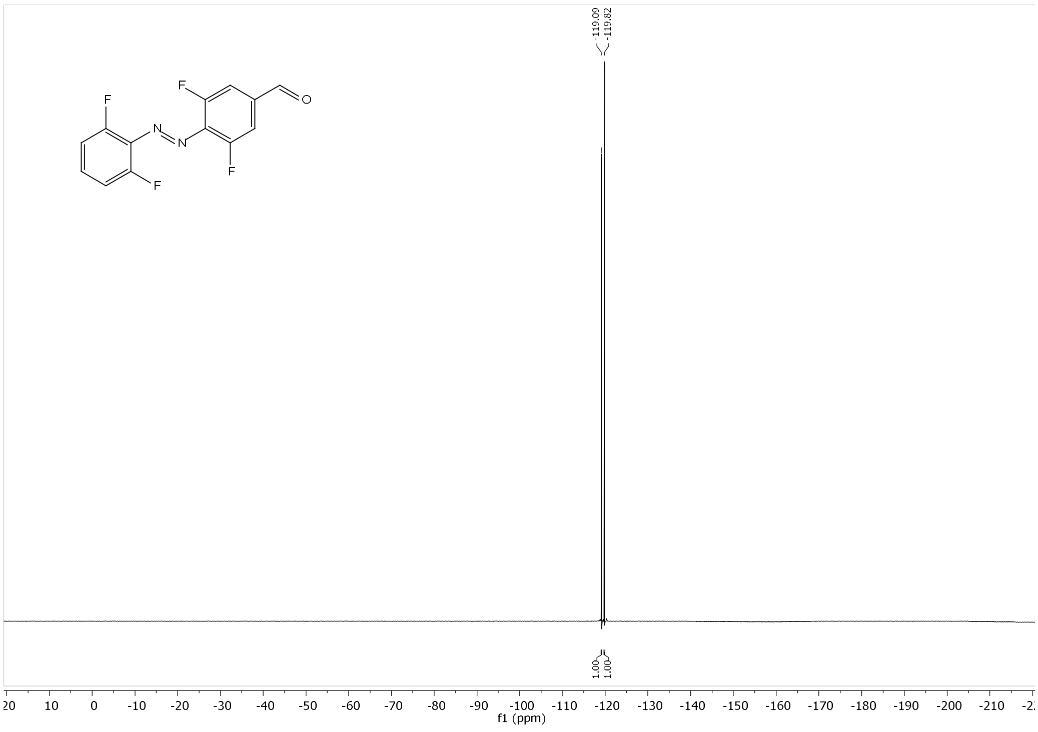

Figure S 75: 19F-NMR-spectrum (376 MHz, CDCl3) of the compound 4.

(*E*)-4,4'-(diazene-1,2-diyl)bis(3,5-difluorobenzaldehyde) (5)

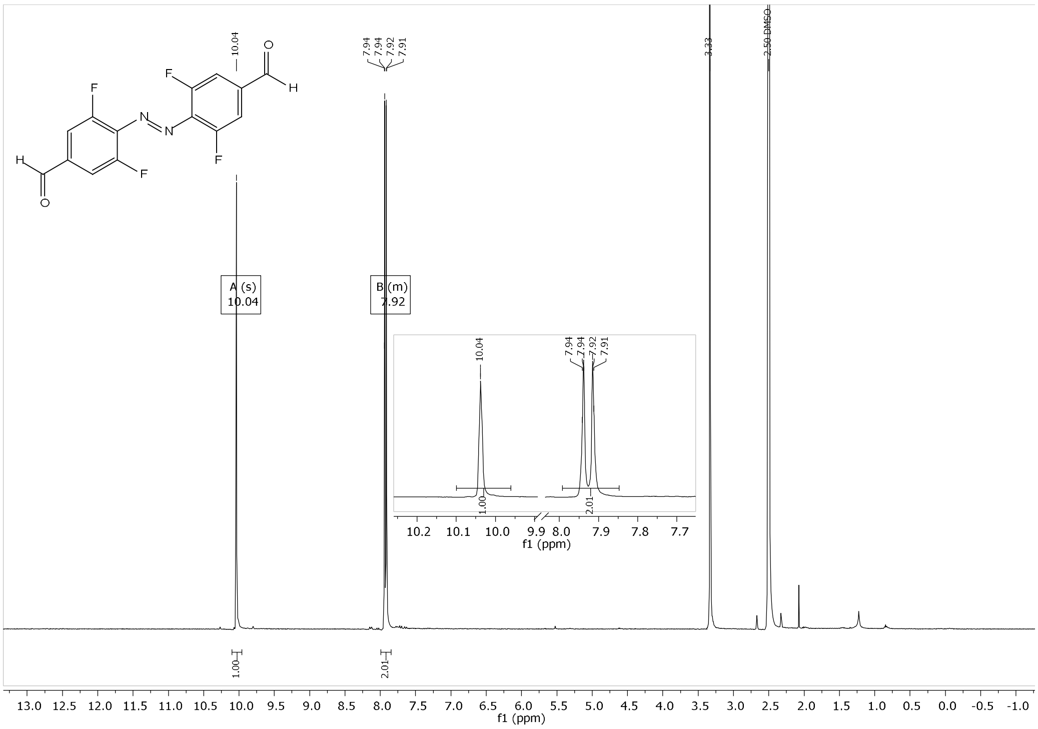

Figure S 76: 1H-NMR-spectrum (400 MHz, DMSO-d6) of the compound 5.

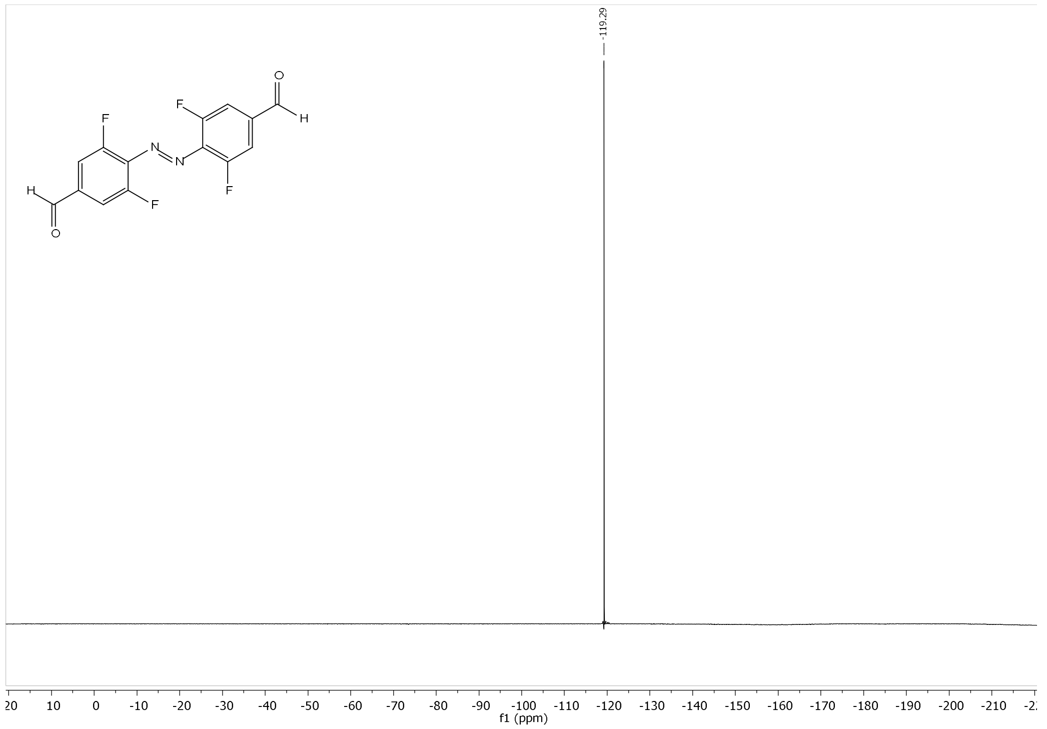

*Figure S 77: ^{19}F-NMR-spectrum (376 MHz, DMSO-d_6) of the compound **5**.*

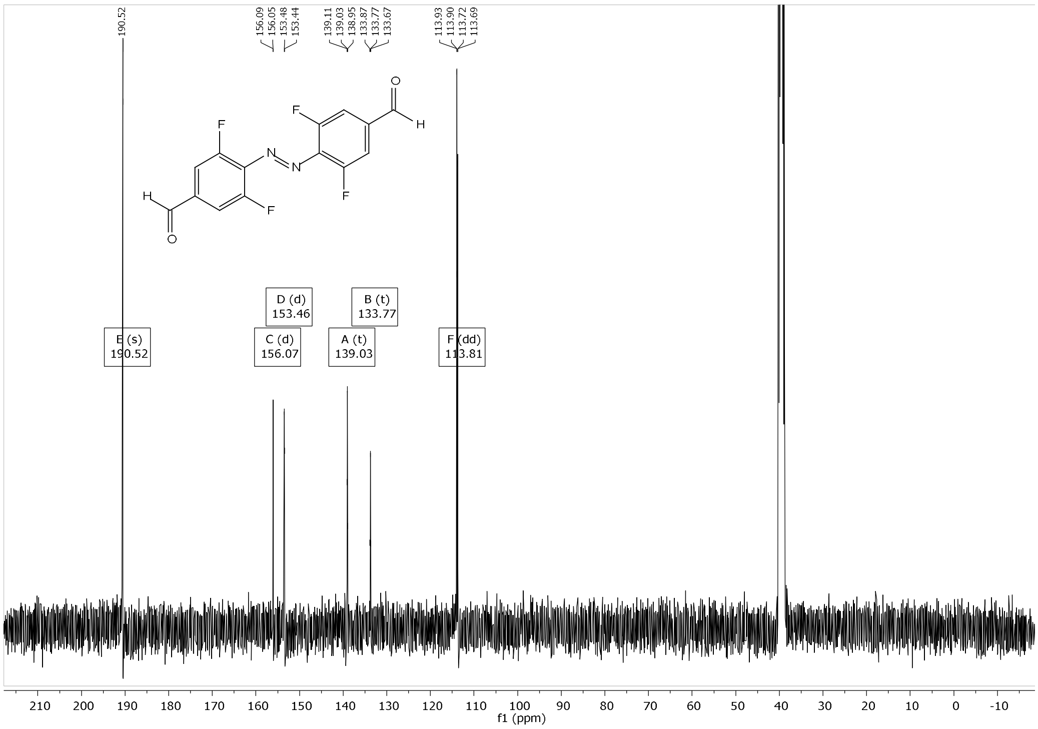

Figure S 78: 13C-NMR-spectrum (101 MHz, DMSO-d6) of the compound 5.

(E)-(3-((2,6-difluorophenyl)diazenyl)-2-fluorophenyl)methanol (6)

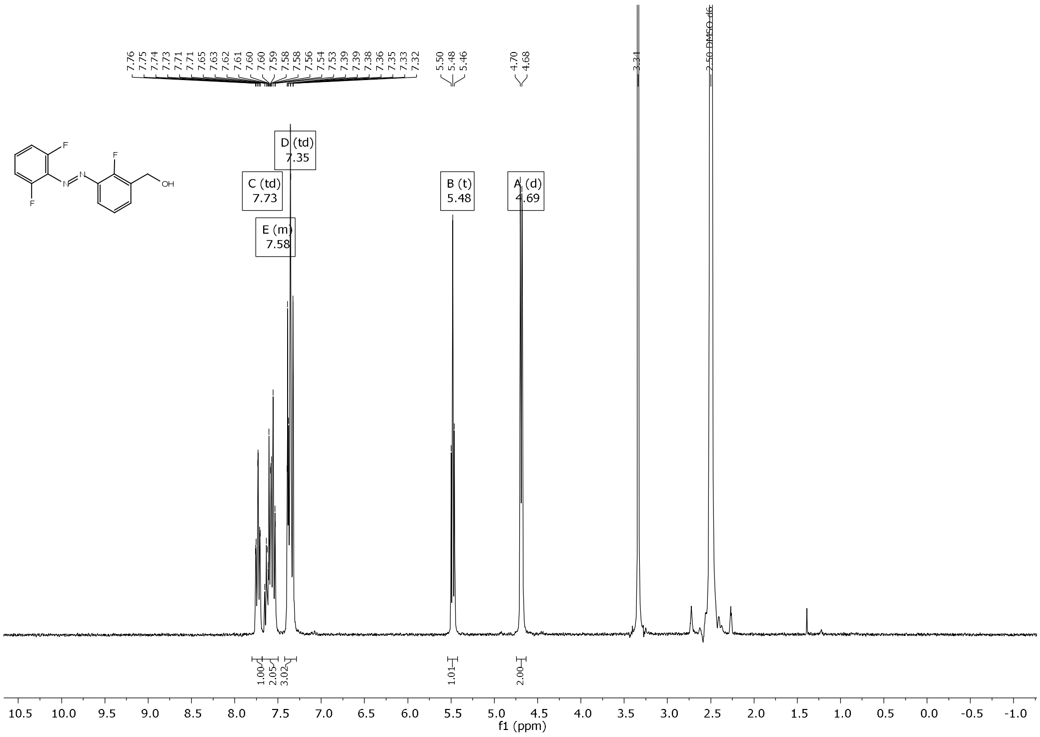

Figure S 79: 1H-NMR-spectrum (300 MHz, DMSO-d6) of the compound 6.

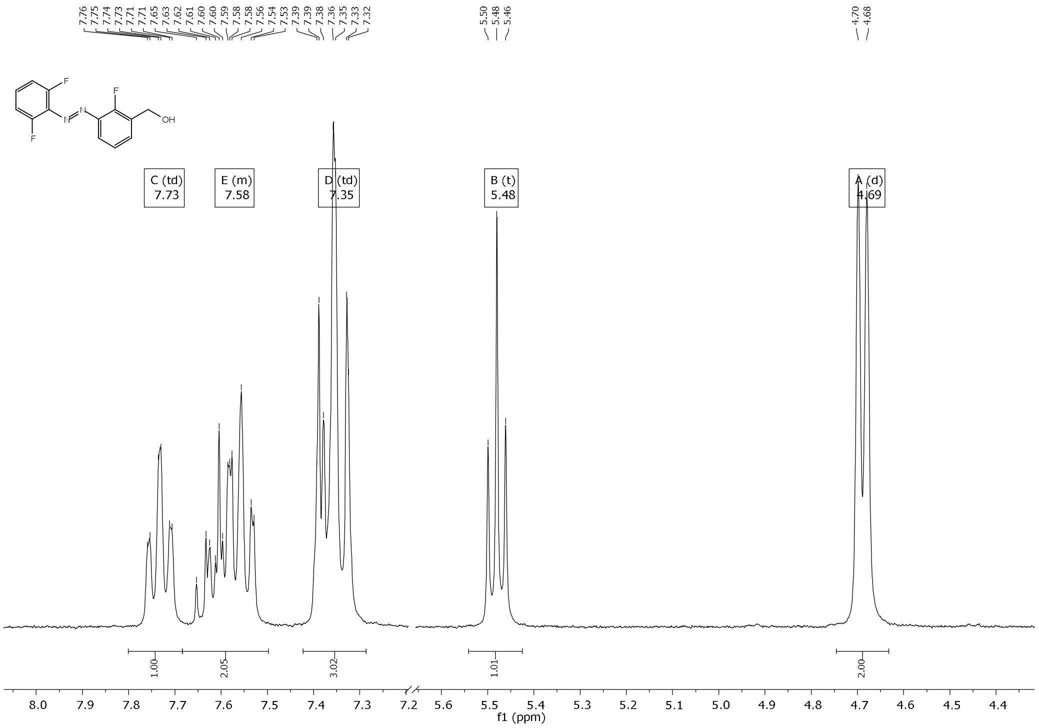

Figure S 80: 1H-NMR-spectrum (300 MHz, DMSO-d6) of the compound 6.

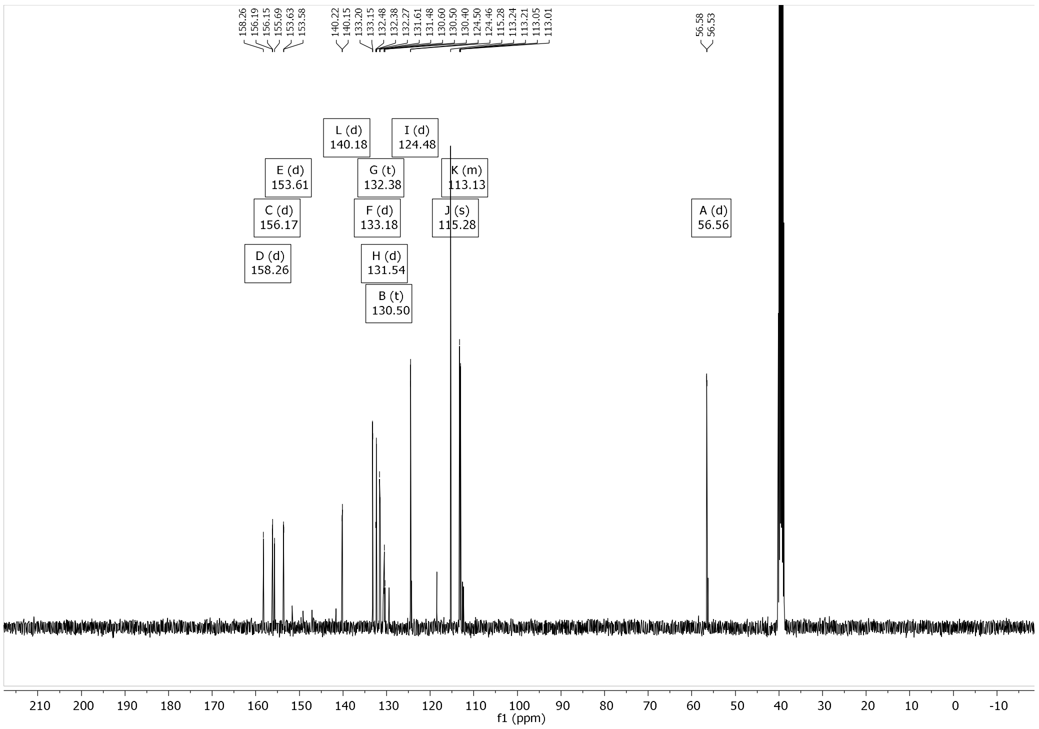

Figure S 81: 13C-NMR-spectrum (101 MHz, DMSO-d6) of the compound 6.

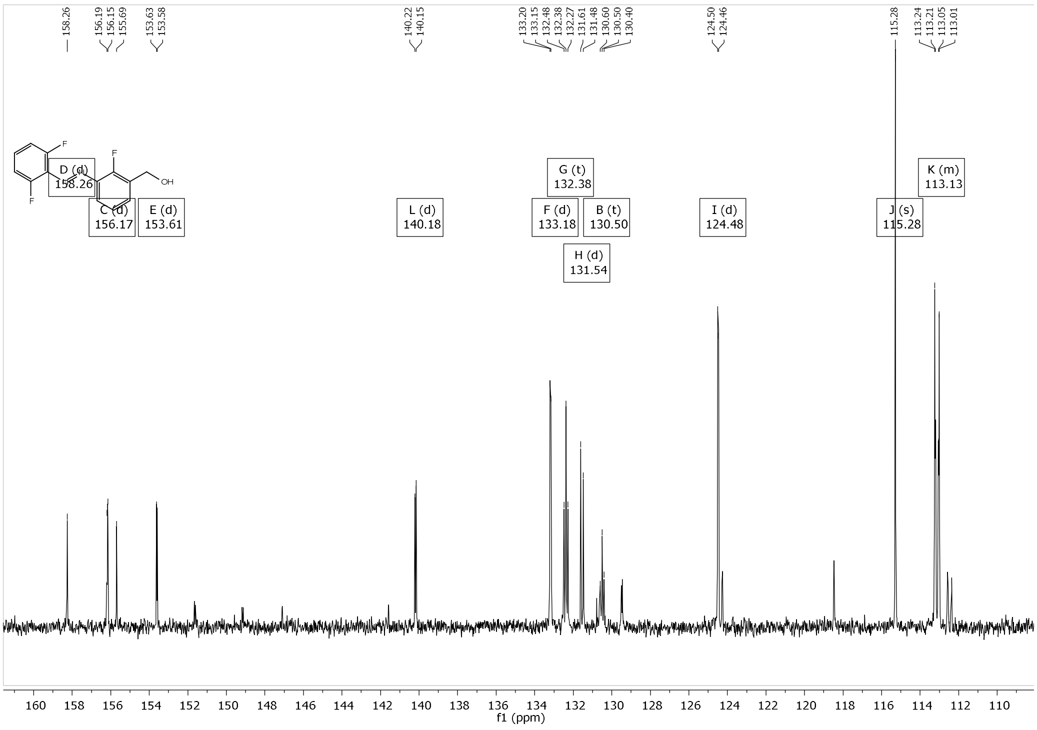

Figure S 82: 13C-NMR-spectrum (101 MHz, DMSO-d6) of the compound 6.

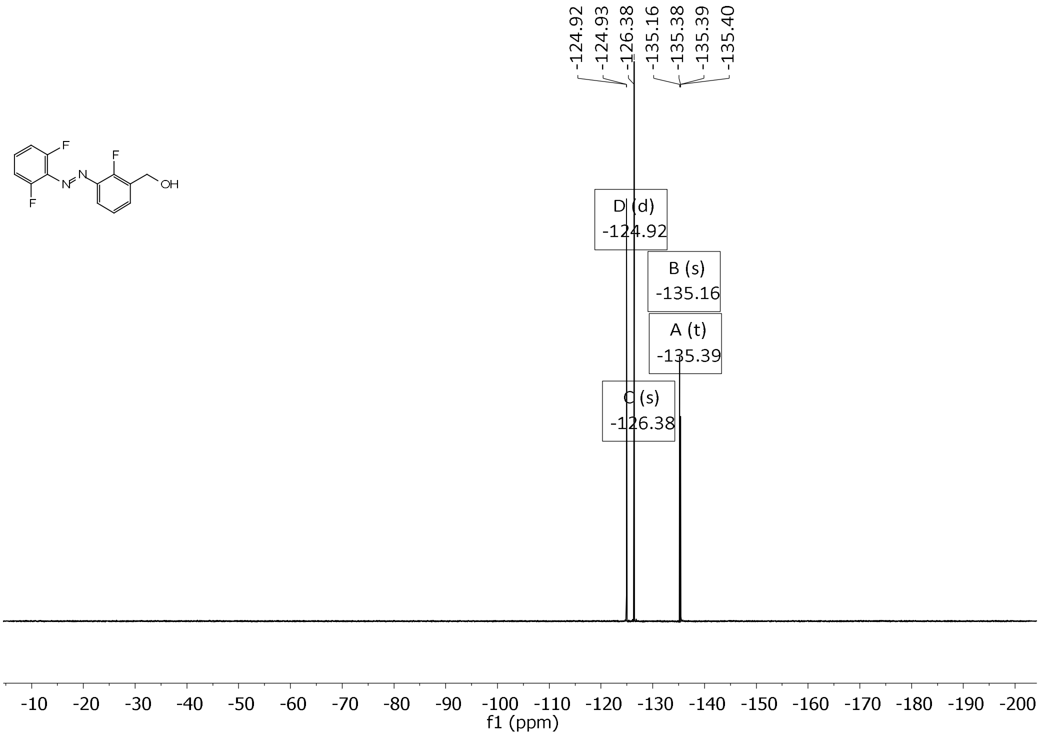

Figure S 83: 19F-NMR-spectrum (376 MHz, DMSO-d6) of the compound 6.

(E)-3-((2,6-difluorophenyl)diazenyl)-2-fluorobenzaldehyde (7)

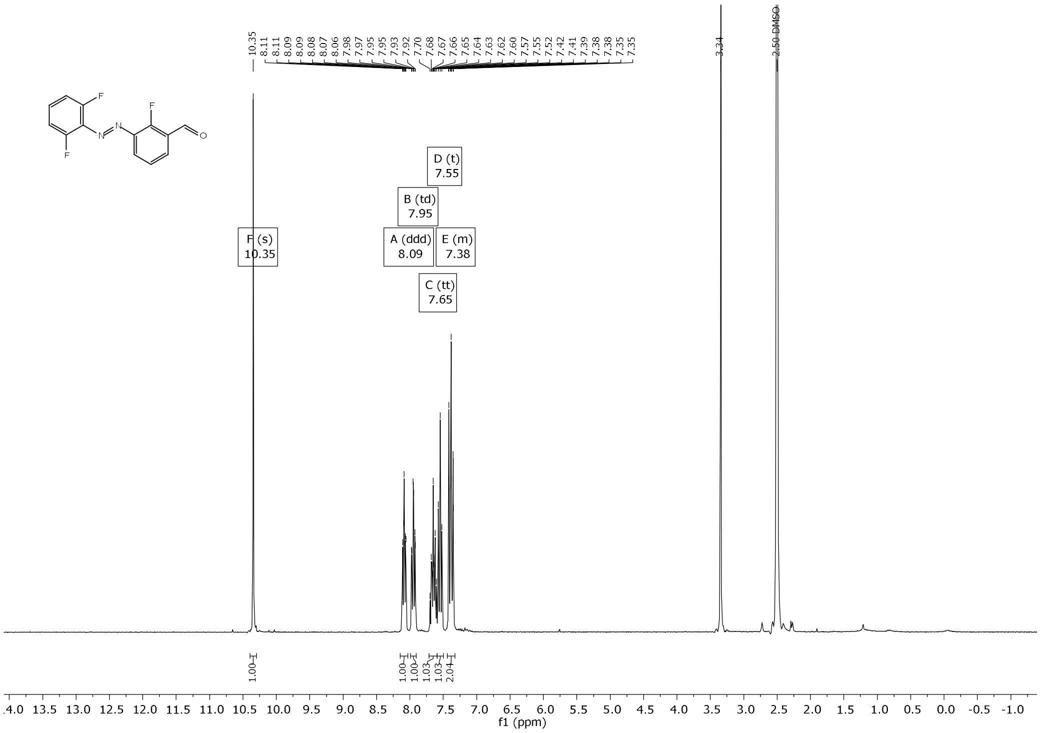

Figure S 84: 1H-NMR-spectrum (300 MHz, DMSO-d6) of the compound 7.

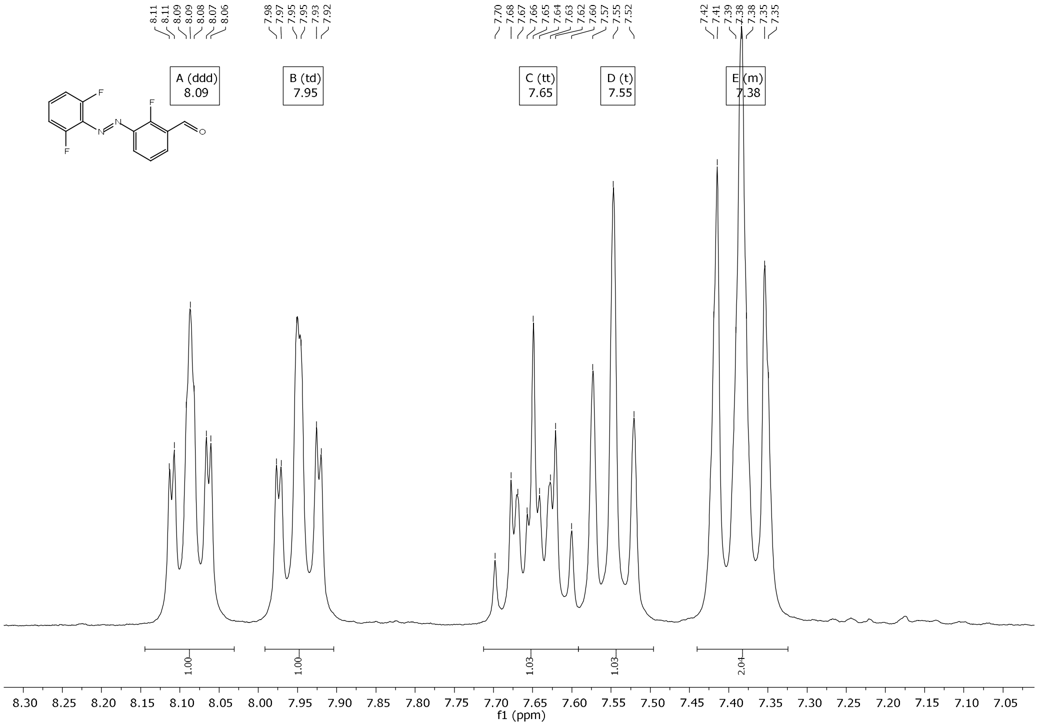

Figure S 85: 1H-NMR-spectrum (300 MHz, DMSO-d6) of the compound 7.

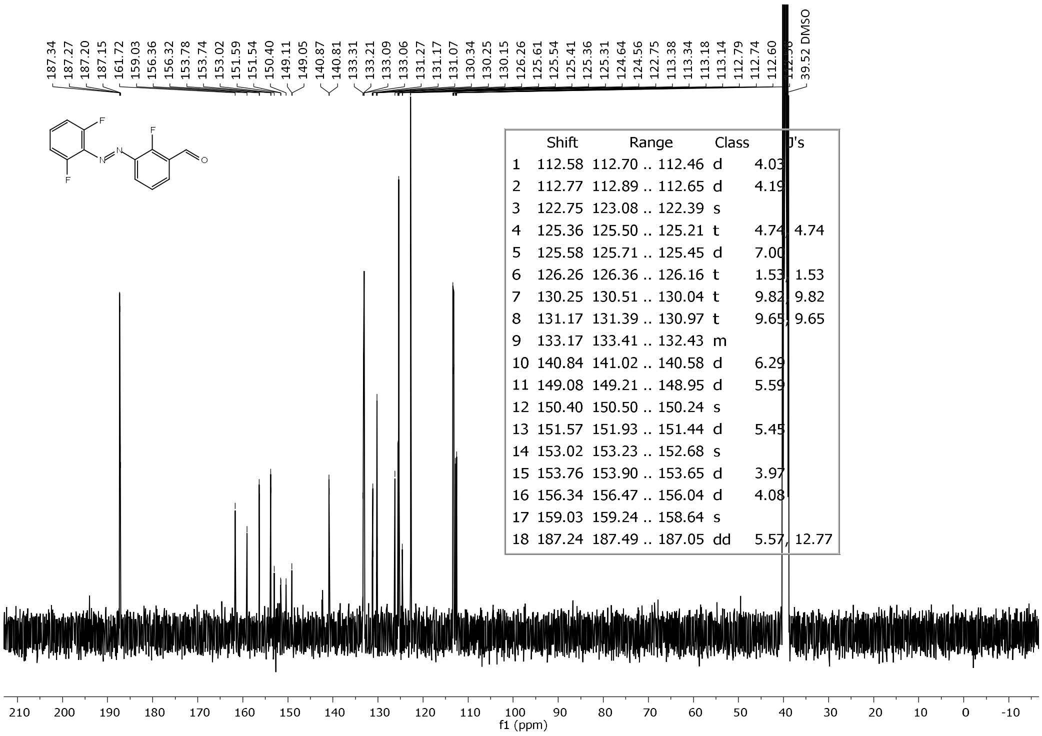

	Shift	Range	Class	J's
1	112.58	112.70 .. 112.46	d	4.03
2	112.77	112.89 .. 112.65	d	4.19
3	122.75	123.08 .. 122.39	s	
4	125.36	125.50 .. 125.21	t	4.74, 4.74
5	125.58	125.71 .. 125.45	d	7.00
6	126.26	126.36 .. 126.16	t	1.53, 1.53
7	130.25	130.51 .. 130.04	t	9.82, 9.82
8	131.17	131.39 .. 130.97	t	9.65, 9.65
9	133.17	133.41 .. 132.43	m	
10	140.84	141.02 .. 140.58	d	6.29
11	149.08	149.21 .. 148.95	d	5.59
12	150.40	150.50 .. 150.24	s	
13	151.57	151.93 .. 151.44	d	5.45
14	153.02	153.23 .. 152.68	s	
15	153.76	153.90 .. 153.65	d	3.97
16	156.34	156.47 .. 156.04	d	4.08
17	159.03	159.24 .. 158.64	s	
18	187.24	187.49 .. 187.05	dd	5.57, 12.77

Figure S 86: 13C-NMR-spectrum (101 MHz, DMSO-d6) of the compound 7.

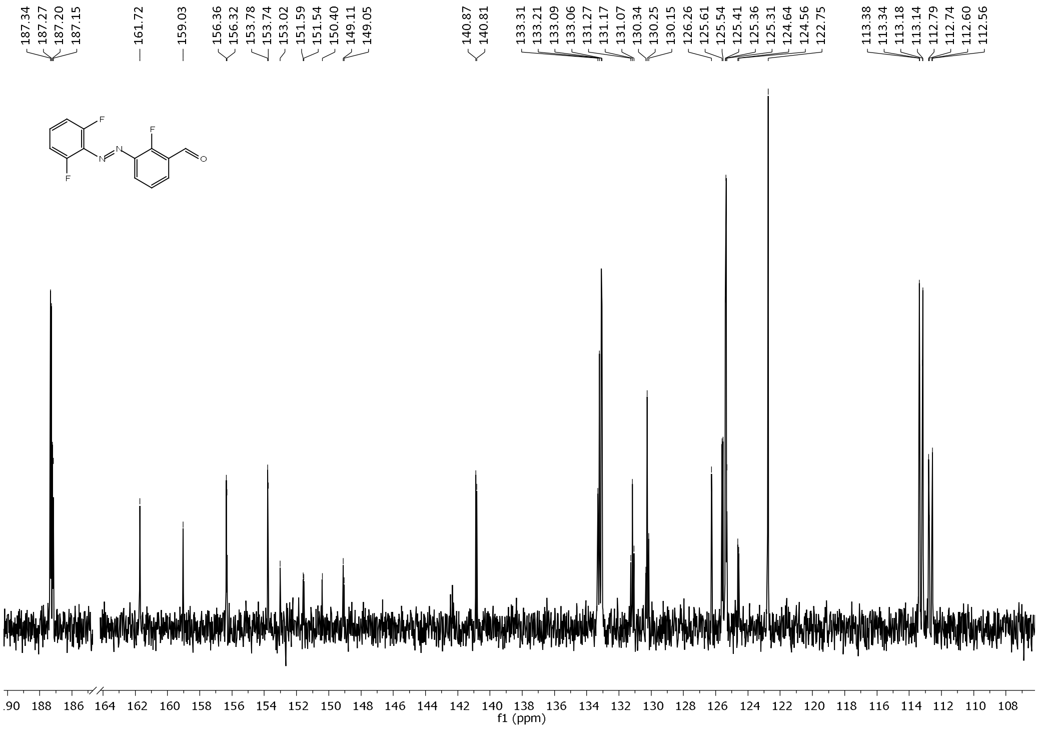

Figure S 87: 13C-NMR-spectrum (101 MHz, DMSO-d6) of the compound 7.

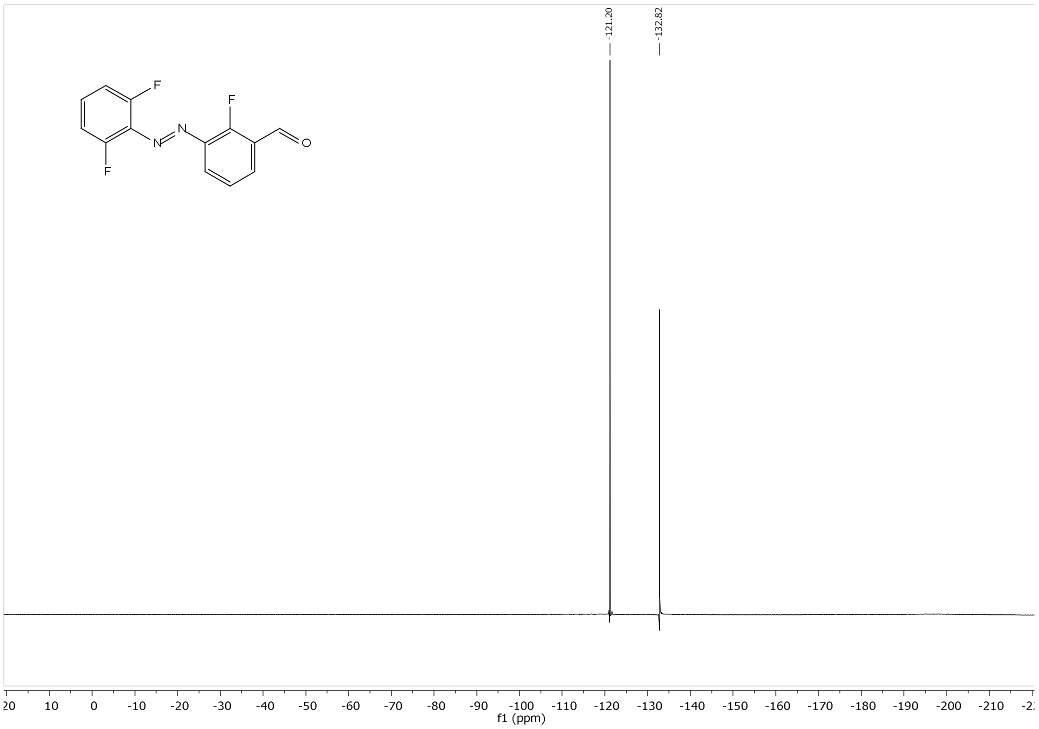

Figure S 88: 19F-NMR-spectrum (376 MHz, DMSO-d6) of the compound 7.

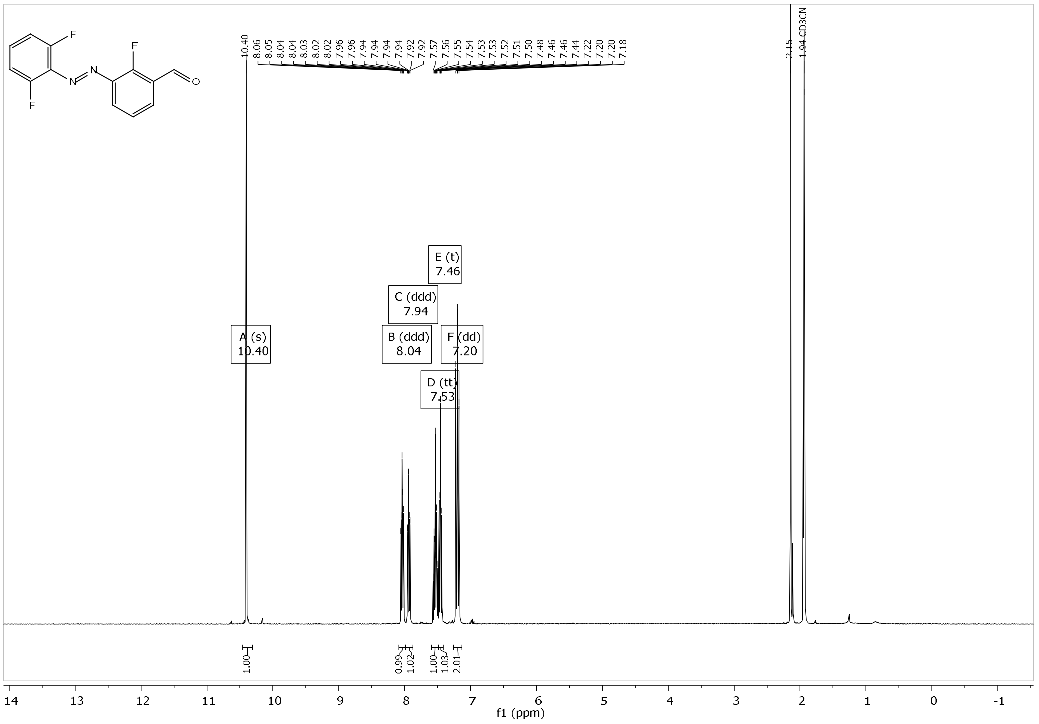

Figure S 89: 1H-NMR-spectrum (400 MHz, CD3CN) of the compound 7.

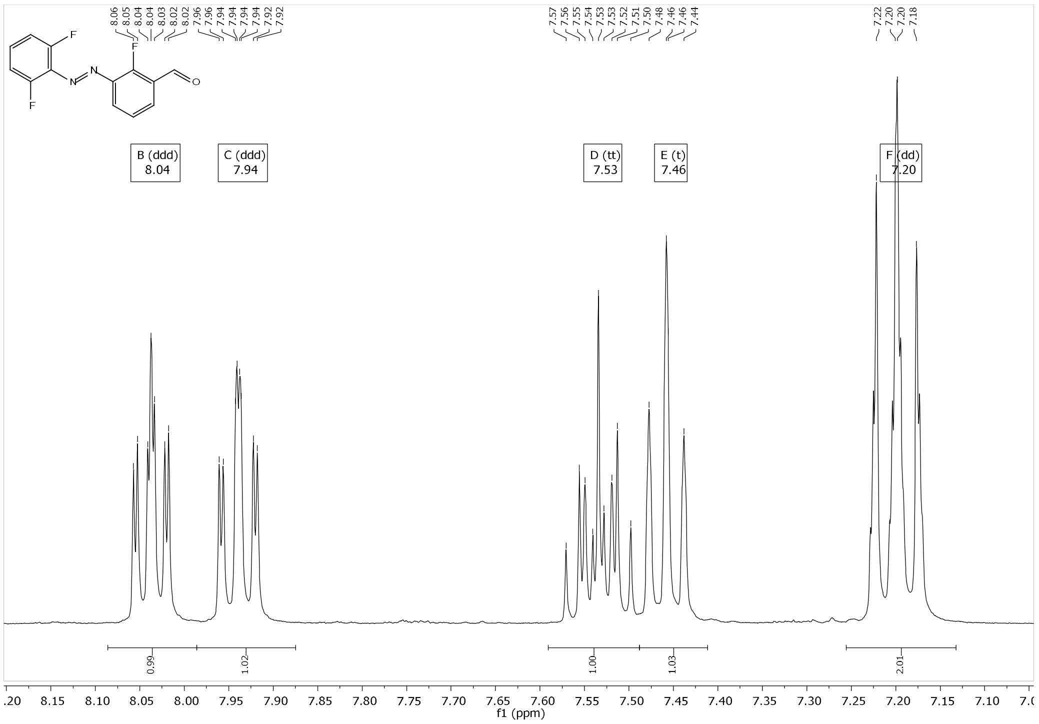

Figure S 90: 1H-NMR-spectrum (400 MHz, CD3CN) of the compound 7.

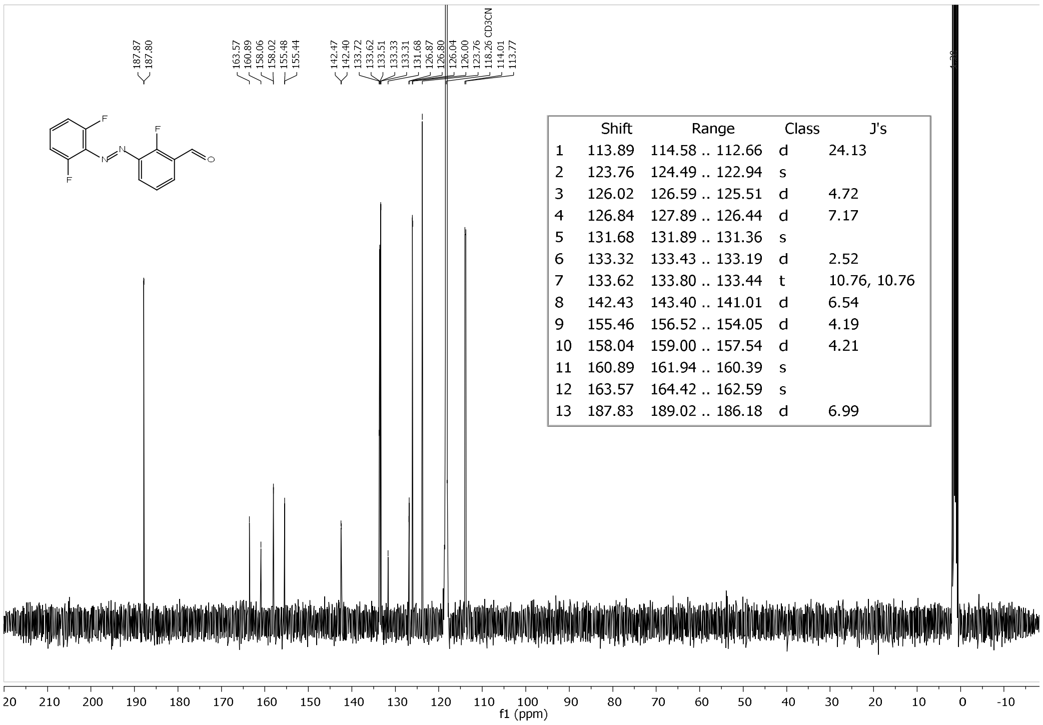

	Shift	Range	Class	J's
1	113.89	114.58 .. 112.66	d	24.13
2	123.76	124.49 .. 122.94	s	
3	126.02	126.59 .. 125.51	d	4.72
4	126.84	127.89 .. 126.44	d	7.17
5	131.68	131.89 .. 131.36	s	
6	133.32	133.43 .. 133.19	d	2.52
7	133.62	133.80 .. 133.44	t	10.76, 10.76
8	142.43	143.40 .. 141.01	d	6.54
9	155.46	156.52 .. 154.05	d	4.19
10	158.04	159.00 .. 157.54	d	4.21
11	160.89	161.94 .. 160.39	s	
12	163.57	164.42 .. 162.59	s	
13	187.83	189.02 .. 186.18	d	6.99

Figure S 91: 13C-NMR-spectrum (101 MHz, CD3CN) of the compound 7.

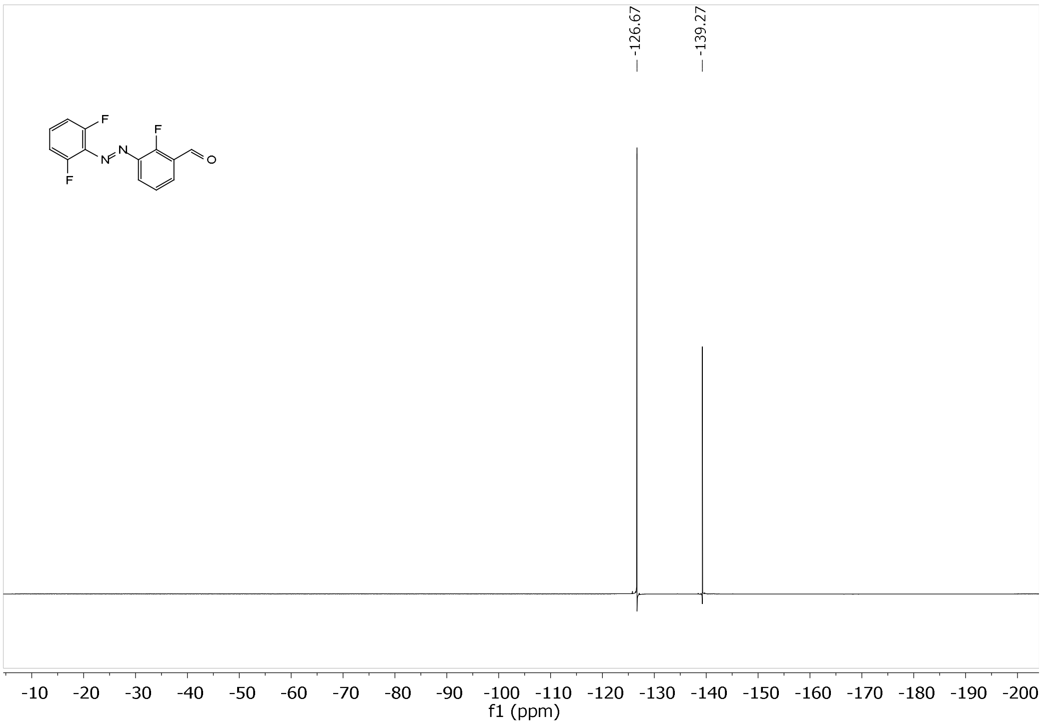

Figure S 92: 19F-NMR-spectrum (376 MHz, CD3CN) of the compound 7.

4-amino-3,5-difluorobenzonitrile (13)

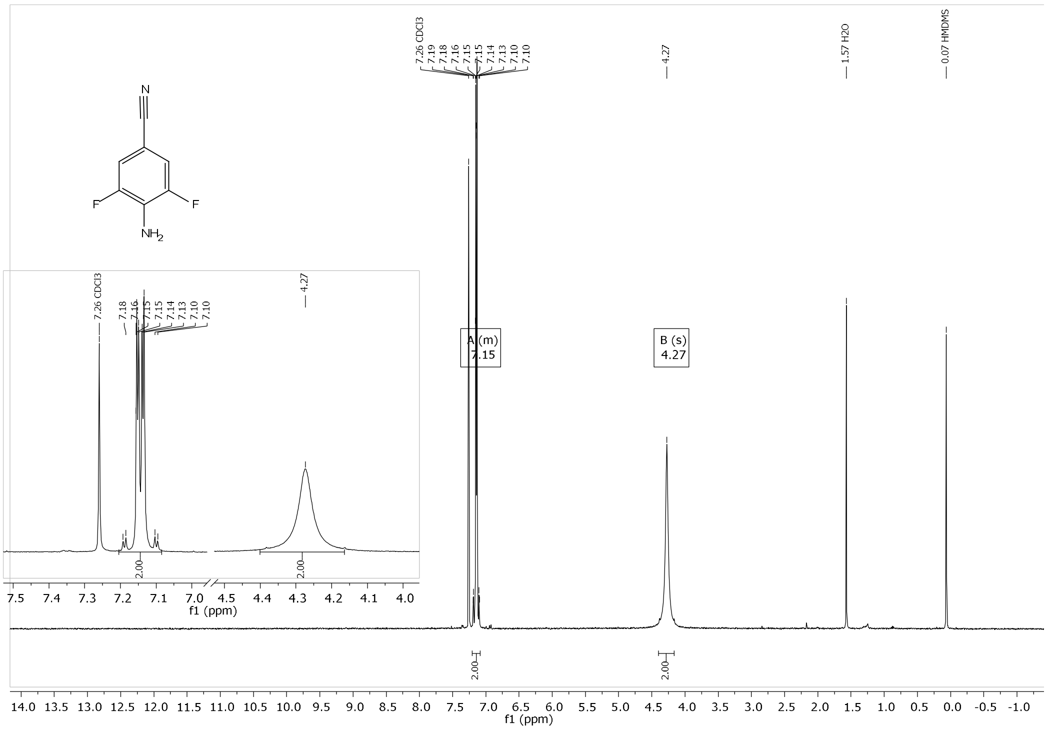

*Figure S 93: ^{1}H NMR spectrum (400 MHz, $CDCl_3$) of the compound **13**.*

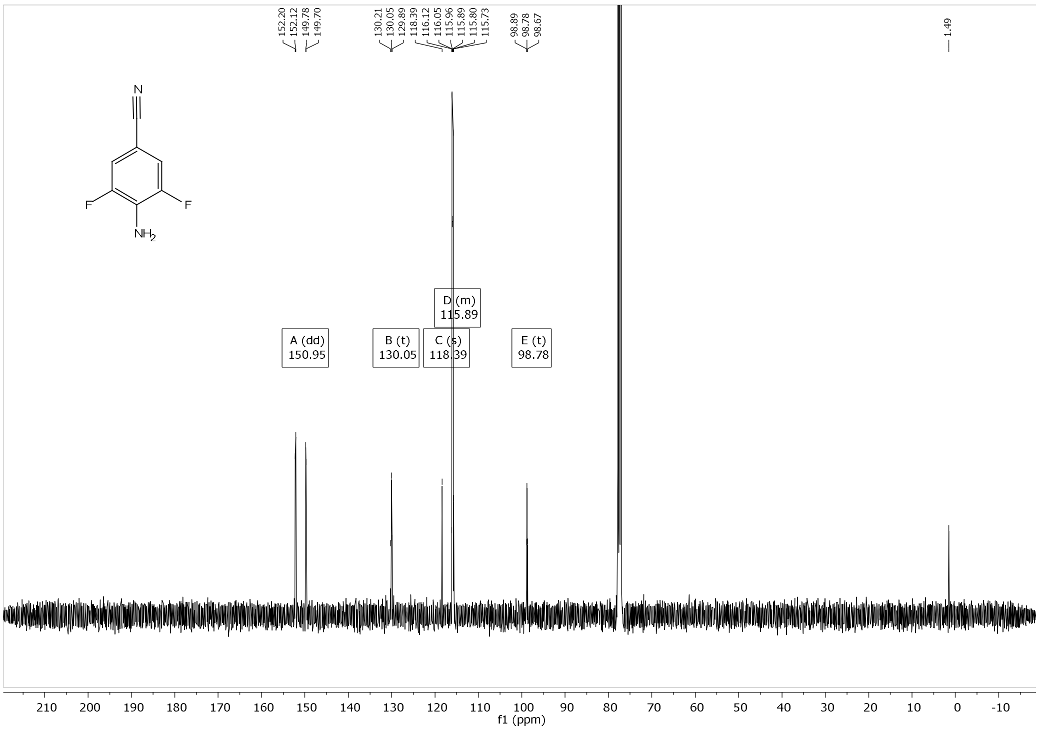

*Figure S 94: ^{13}C NMR spectrum (101 MHz, $CDCl_3$) of the compound **13.***

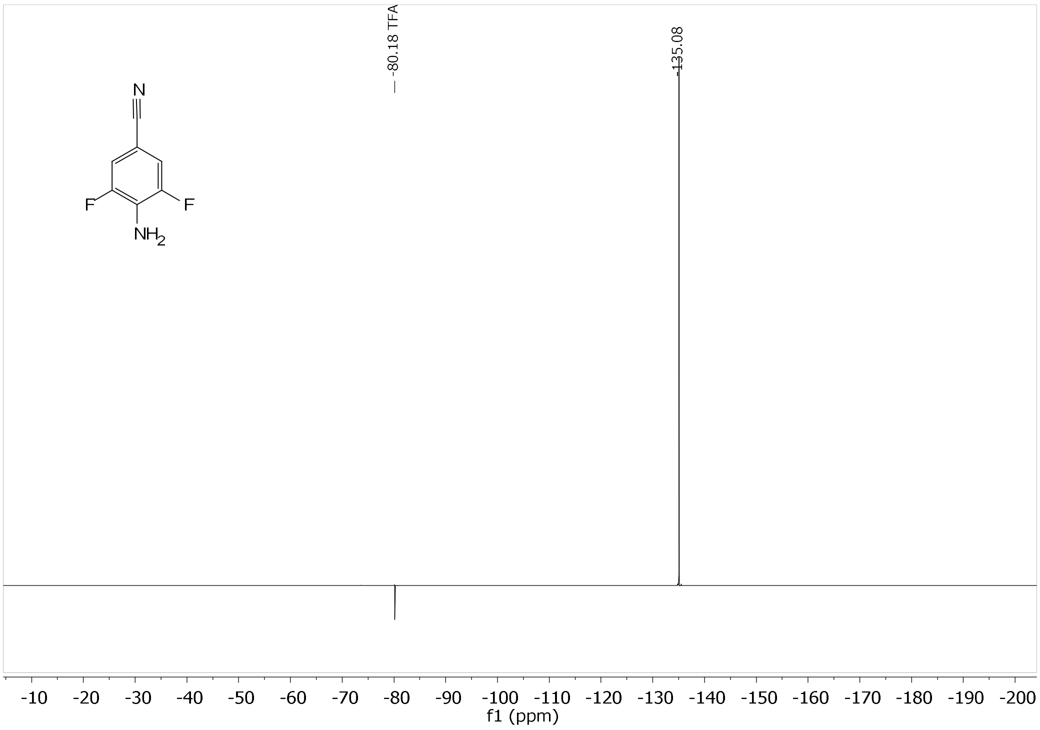

*Figure S 95: ^{19}F NMR spectrum (376 MHz, $CDCl_3$) of the compound **13**.*

4-amino-3,5-difluorobenzoic acid (14)

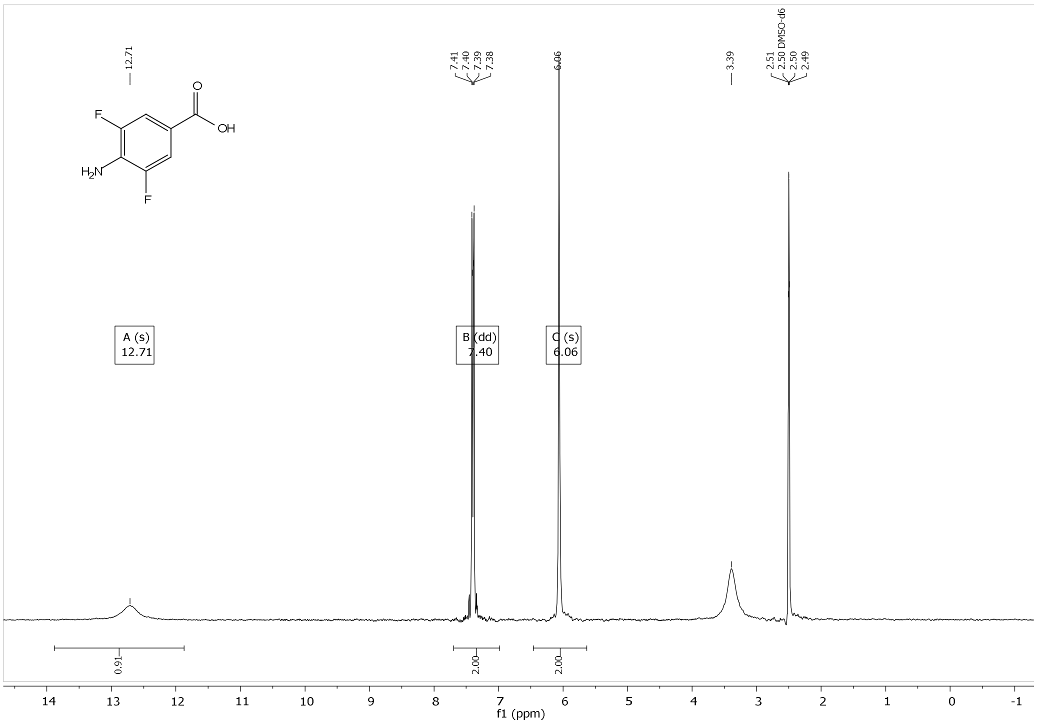

*Figure S 96: ^{1}H NMR spectrum (400 MHz, DMSO-d_6) of the compound **14**.*

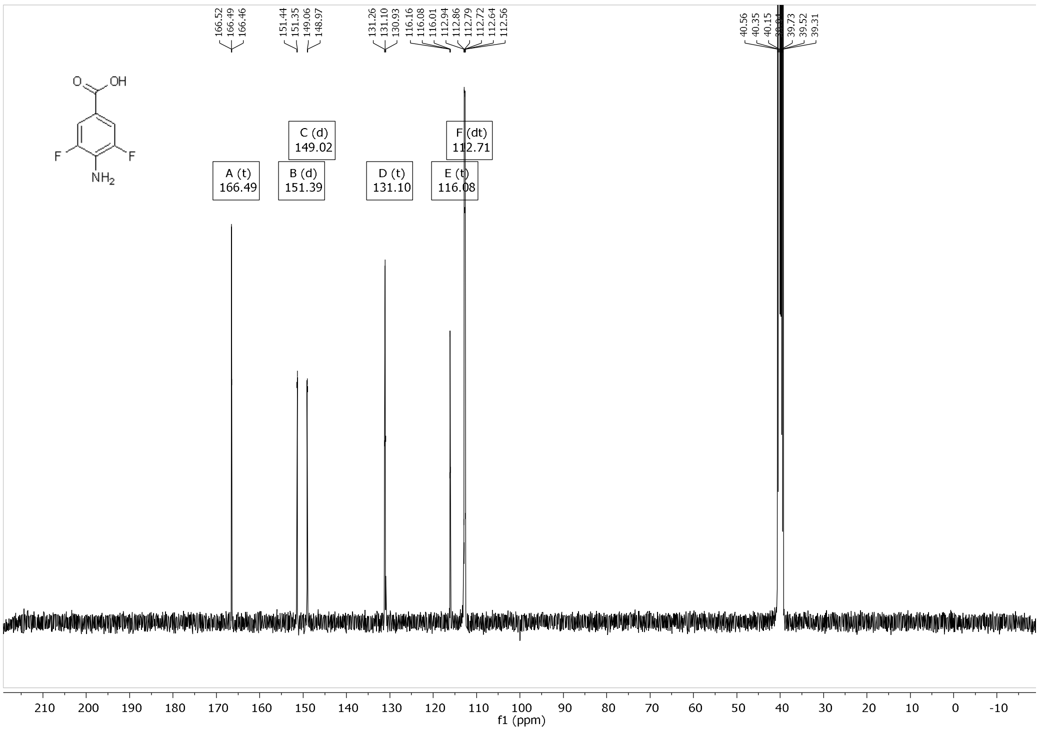

*Figure S 97: ^{13}C NMR spectrum (101 MHz, DMSO) of the compound **14.***

(4-amino-3,5-difluorophenyl)methanol (15)

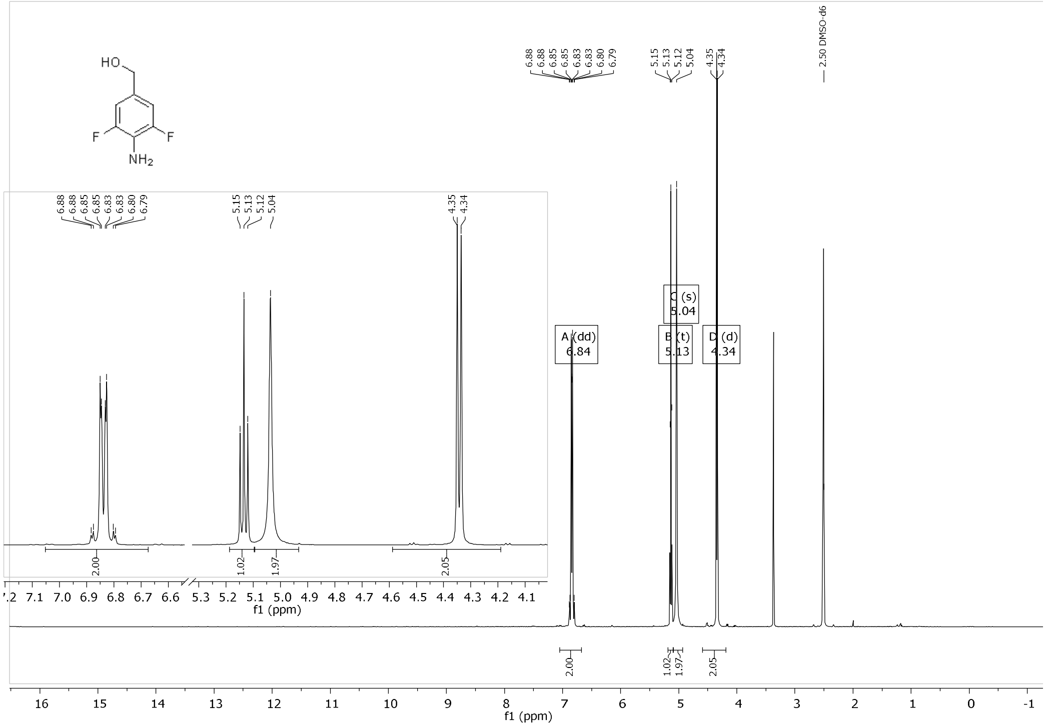

*Figure S 98: ^{1}H-NMR-spectrum (400 MHz, DMSO-d_6) of the compound **15**.*

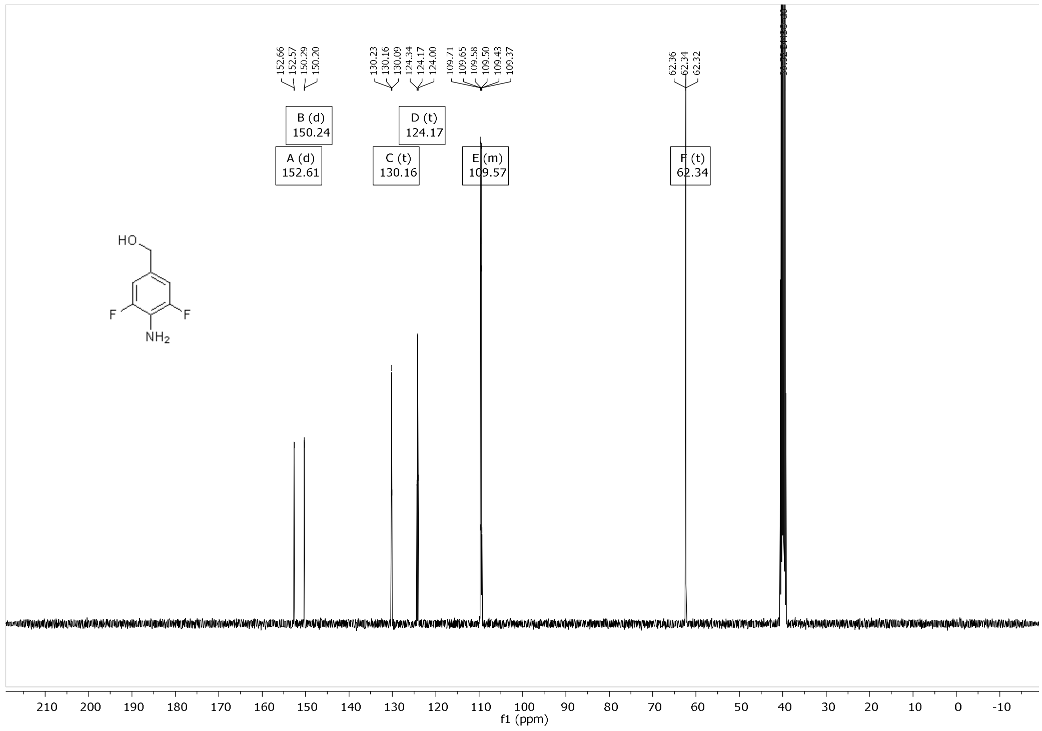

*Figure S 99: ^{13}C-NMR-spectrum (101 MHz, DMSO-d_6) of the compound **15.***

4-Amino-3,5-difluorobenzyl acetate (16)

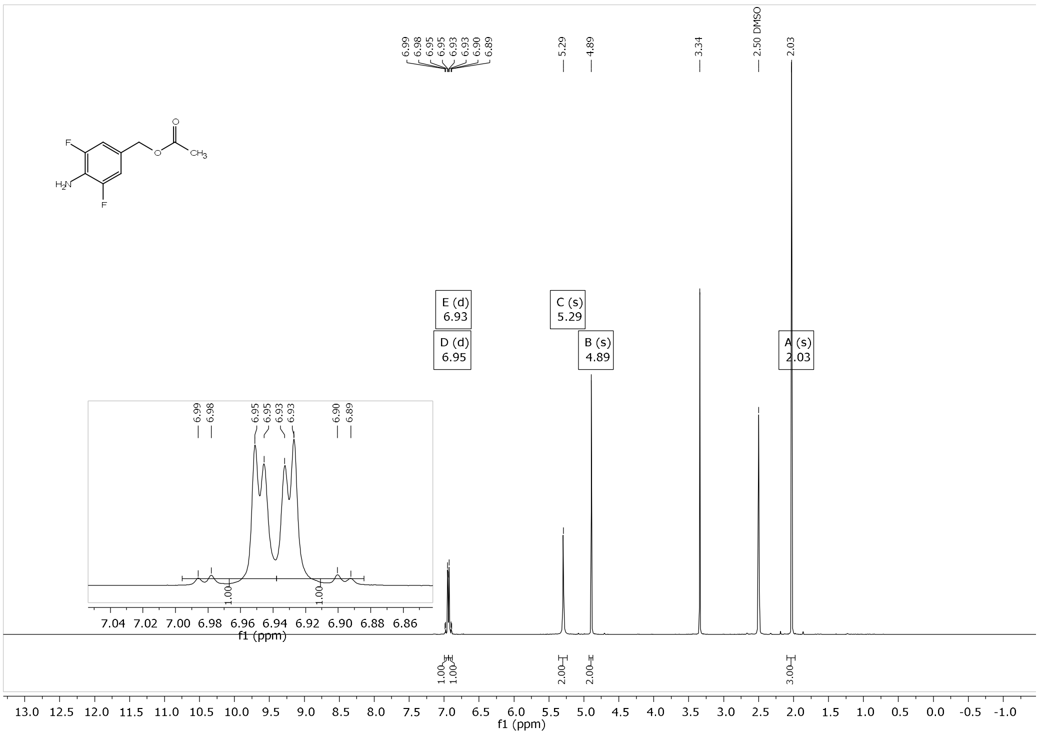

*Figure S 100: ^{1}H-NMR-spectrum (400 MHz, DMSO-d_6) of the compound **16**.*

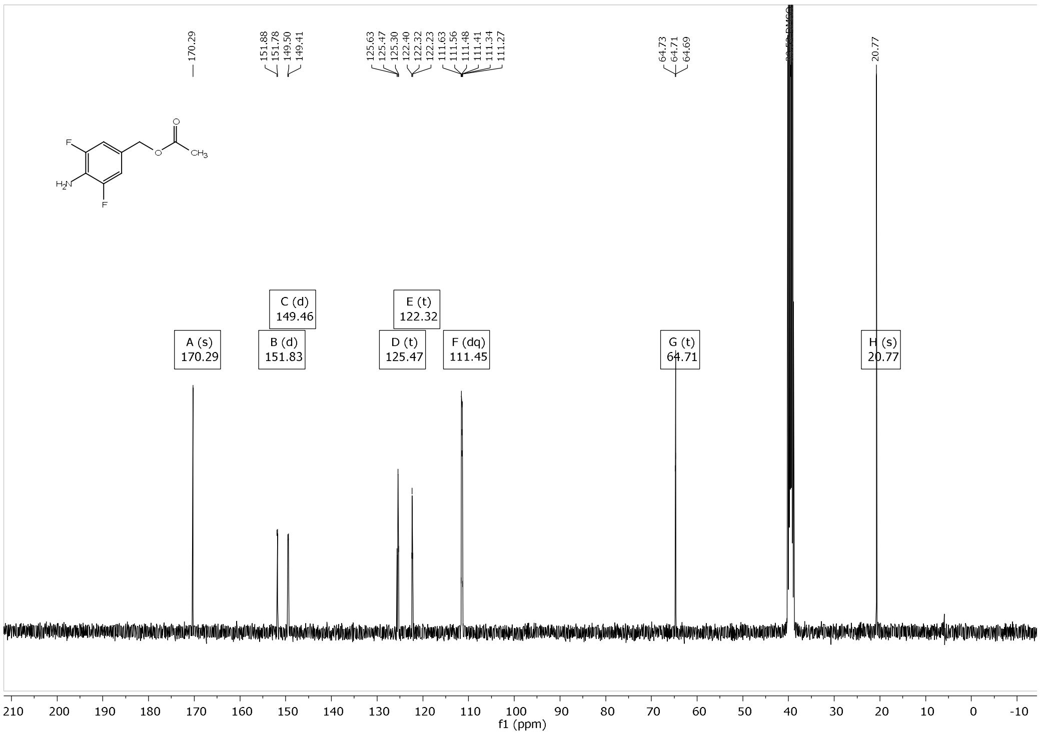

*Figure S 101: ^{13}C-NMR-spectrum (101 MHz, DMSO-d6) of the compound **16**.*

(*E*)-4-((2,6-Difluorophenyl)diazenyl)-3,5-difluorobenzyl acetate (18)

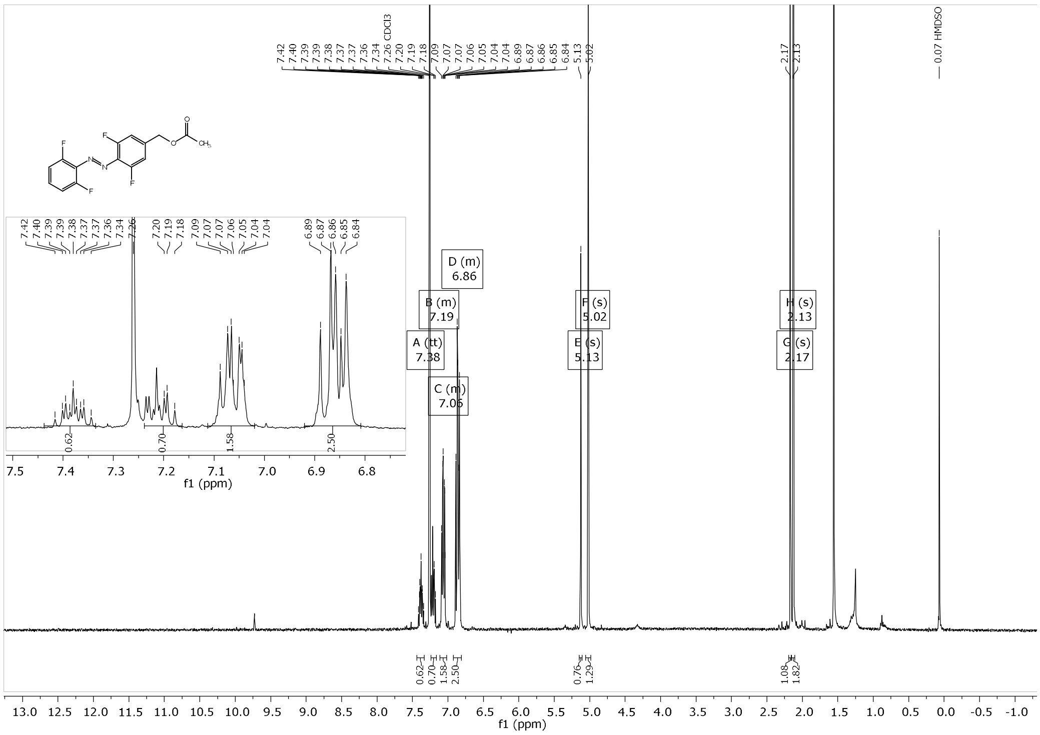

*Figure S 102: ^{1}H-NMR-spectrum (400 MHz, $CDCl_3$) of the compound **18** (mixed isomers).*

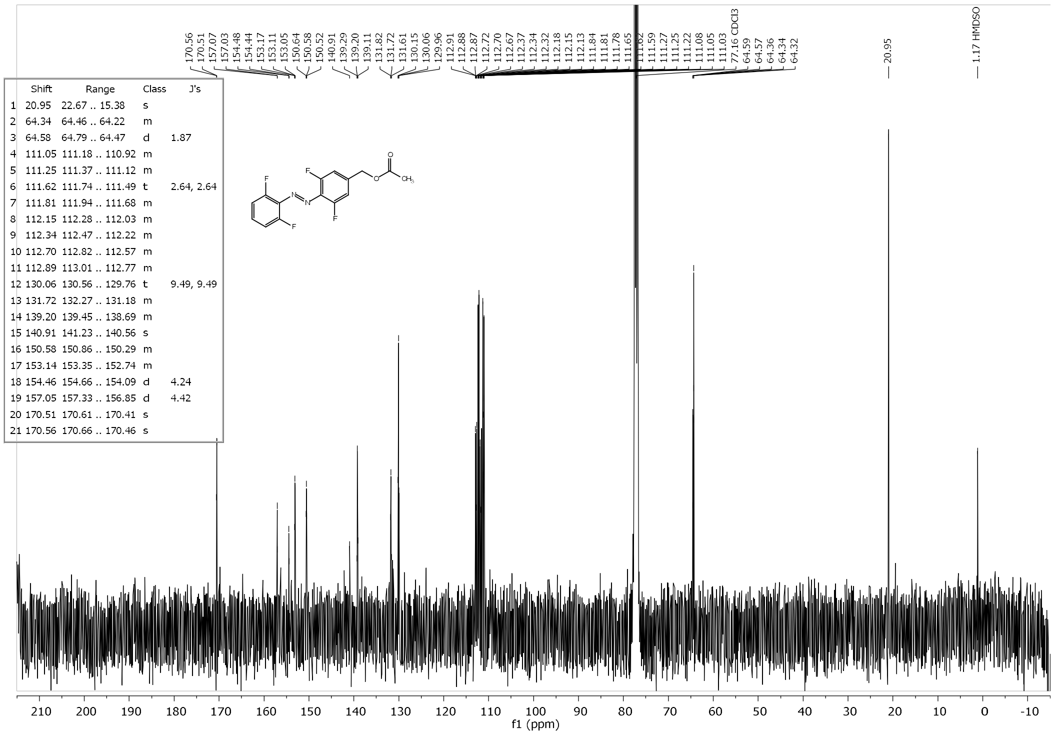

*Figure S 103: ^{13}C-NMR-spectrum (101 MHz, $CDCl_3$) of the compound **18** (mixed isomers)*

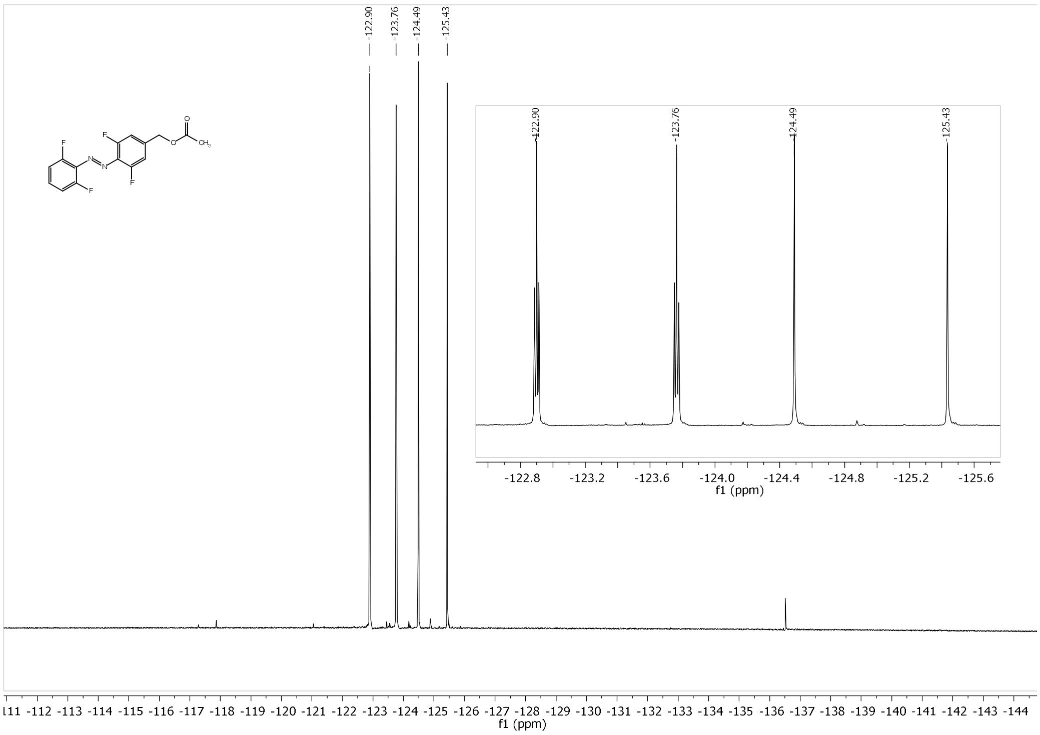

*Figure S 104: ^{19}F-NMR-spectrum (376 MHz, $CDCl_3$) of the compound **18** (mixed isomers).*

(E)-(diazene-1,2-diylbis(3,5-difluoro-4,1-phenylene))bis(methylene) diacetate (19)

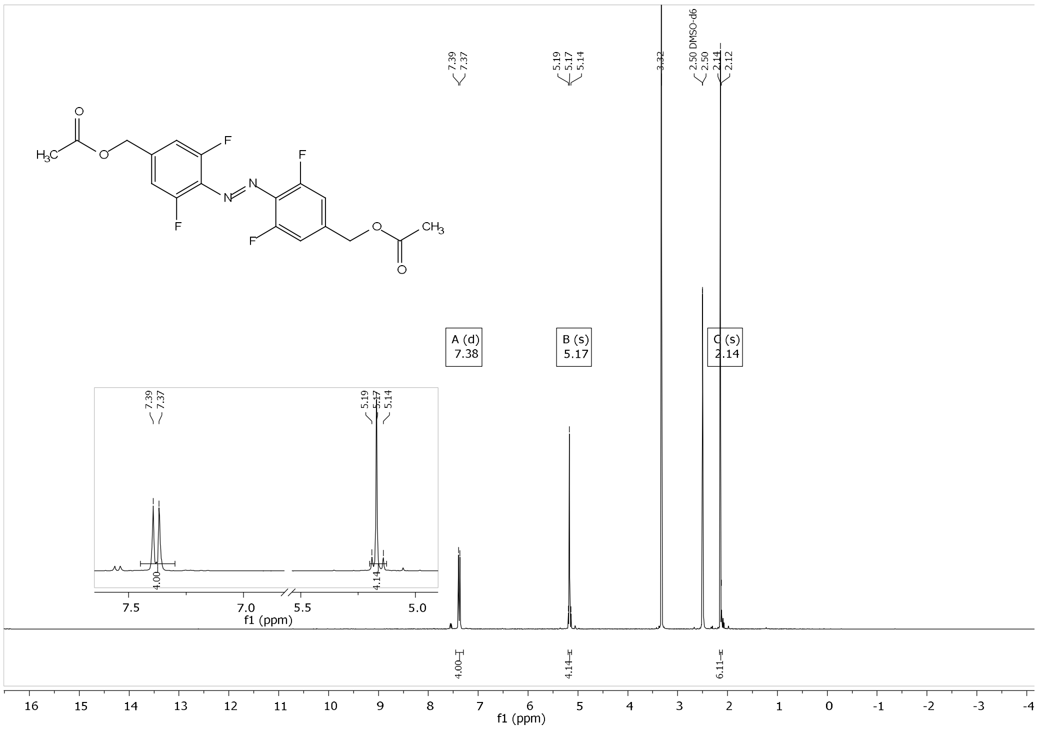

*Figure S 105: ^{1}H-NMR-spectrum (400 MHz, DMSO-d_6) of the compound **19**.*

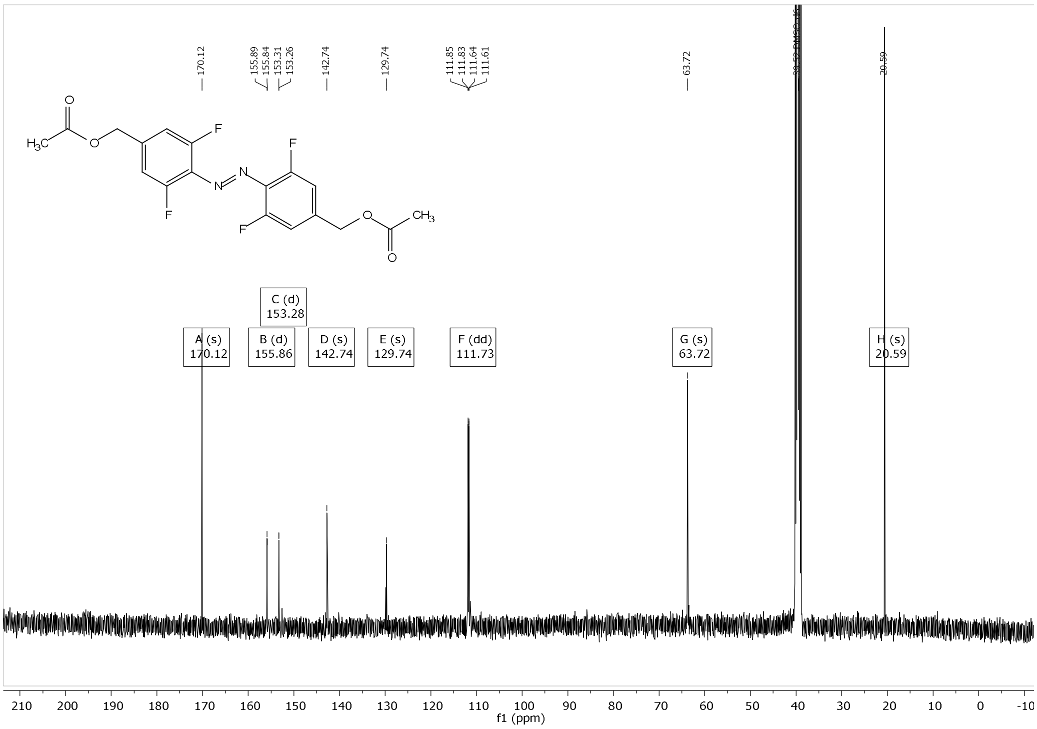

*Figure S 106: ^{13}C-NMR-spectrum (101 MHz, DMSO-d_6) of the compound **19**.*

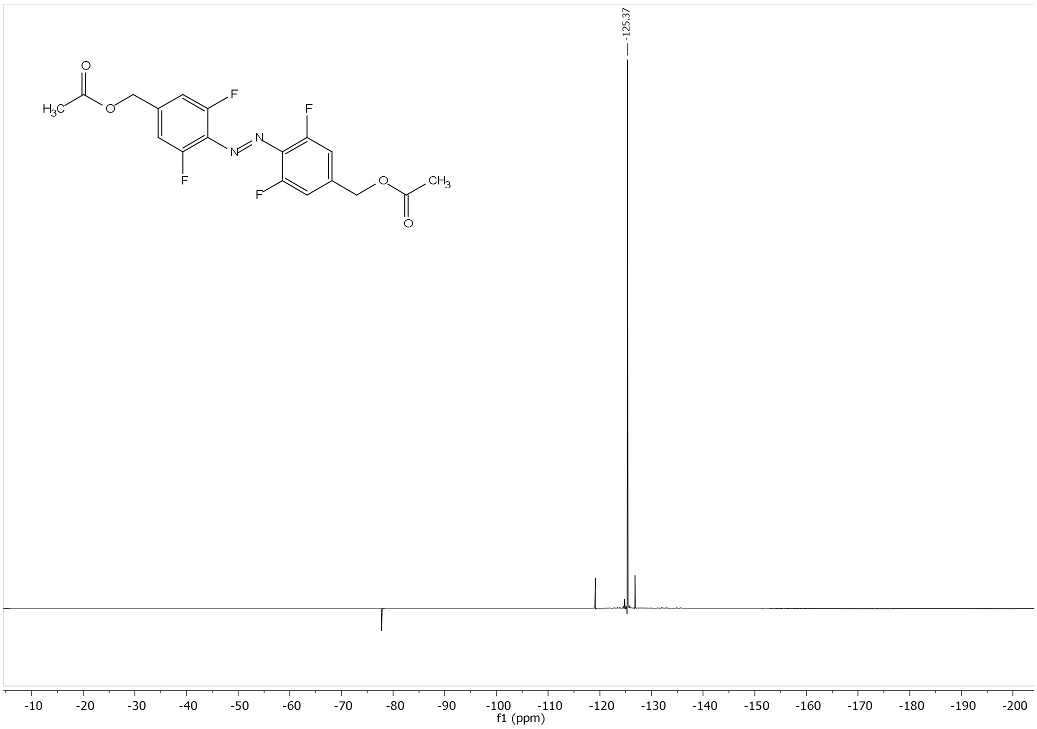

*Figure S 107: ^{19}F NMR spectrum (376 MHz, DMSO) of the compound **19.***

Fmoc-Lys(Boc)-Gly-OMe (22)

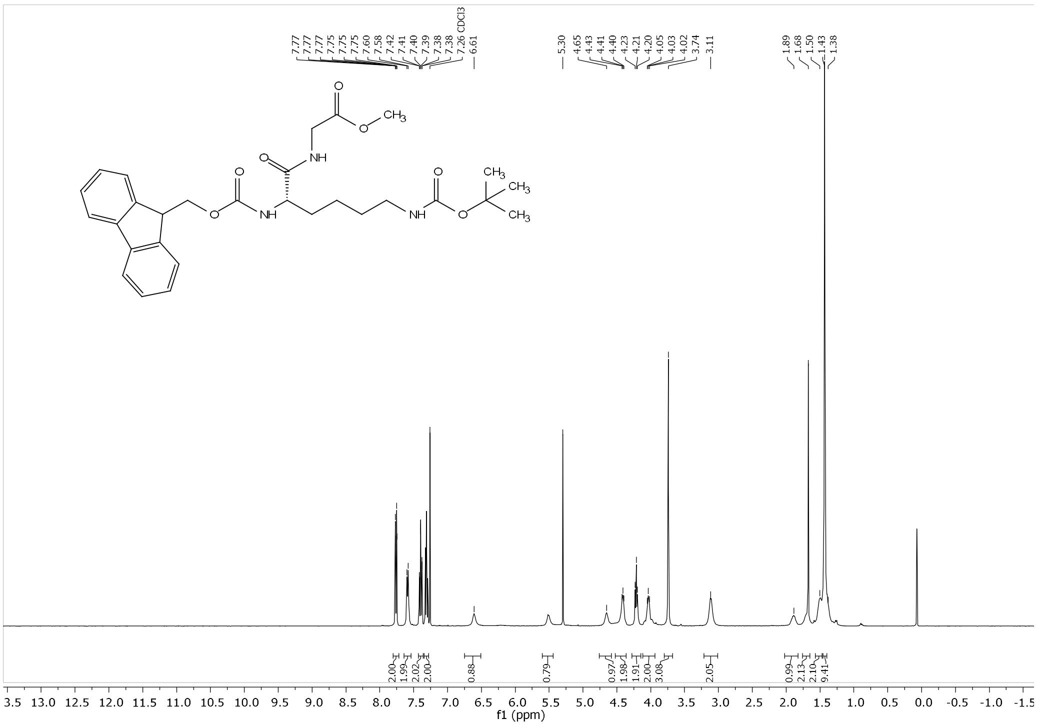

Figure S 108: ^{1}H-NMR-spectrum (400 MHz, $CDCl_3$) of the compound **22**.

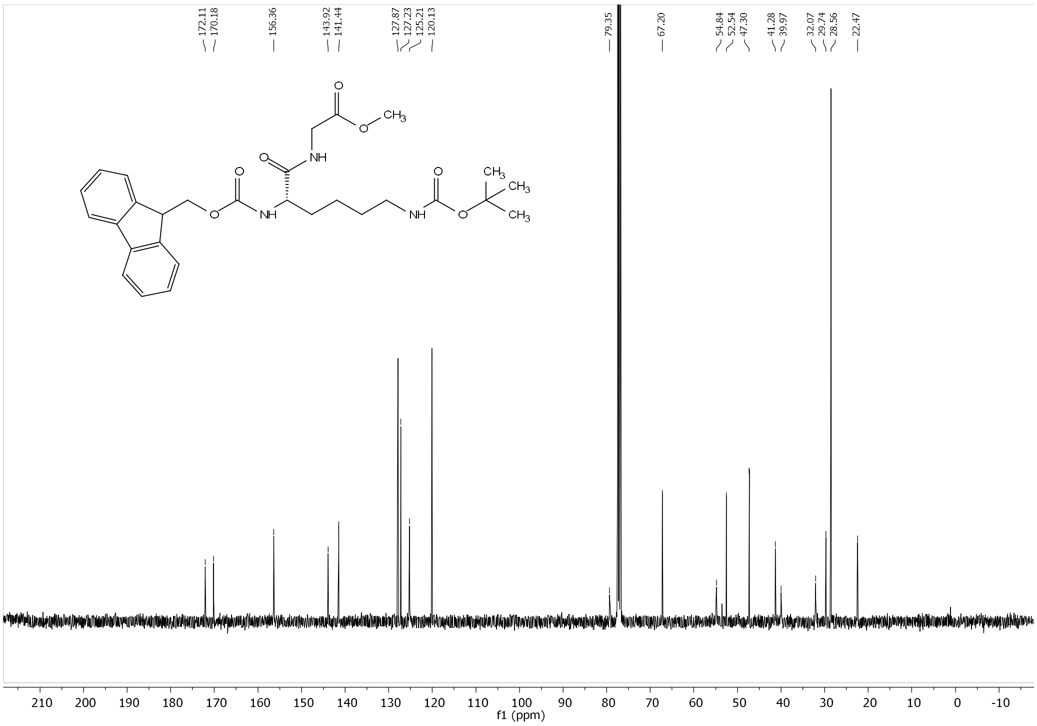

Figure S 109: ^{13}C-NMR-spectrum (101 MHz, $CDCl_3$) of the compound **22**.

cyclo(Lys(Boc)-Gly) (23)

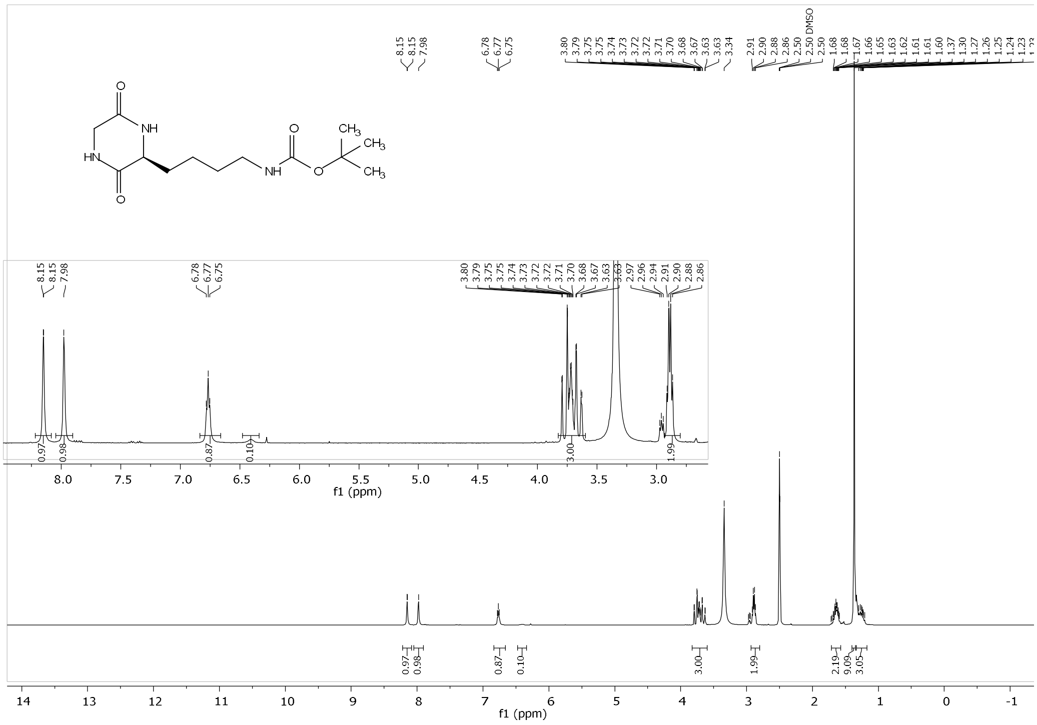

*Figure S 110: ^{1}H-NMR-spectrum (400 MHz, DMSO-d_6) of the compound **23**.*

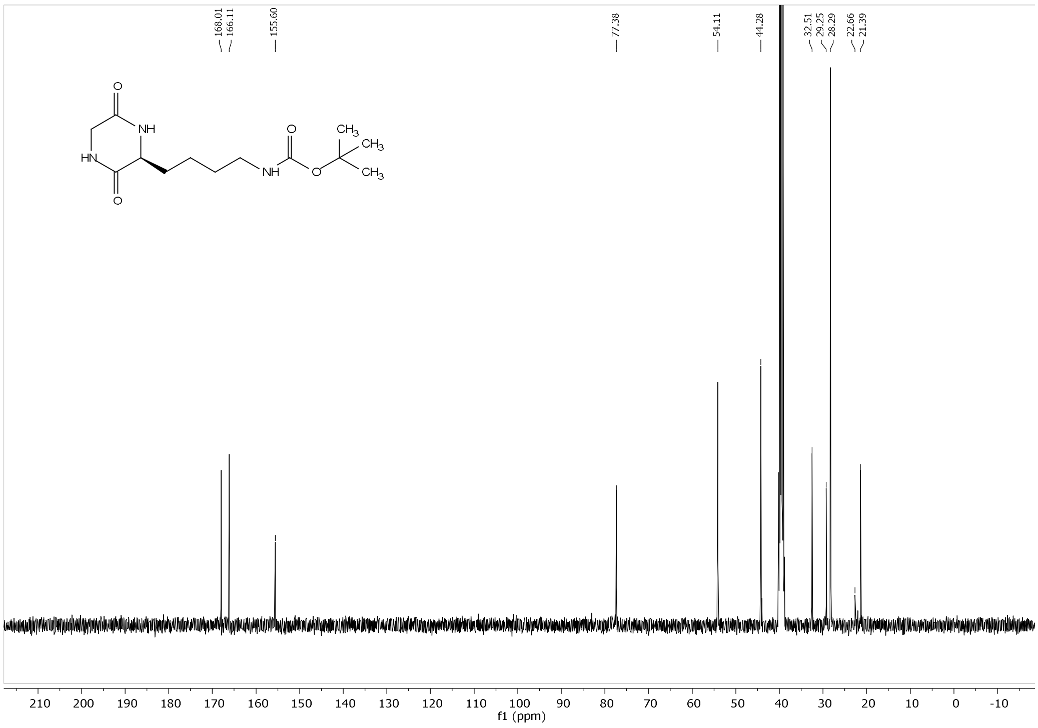

*Figure S 111: ^{13}C-NMR-spectrum (101 MHz, DMSO-d_6) of the compound **23**.*

Di-Boc-cyclo(Lys(Boc)-Gly) (24)

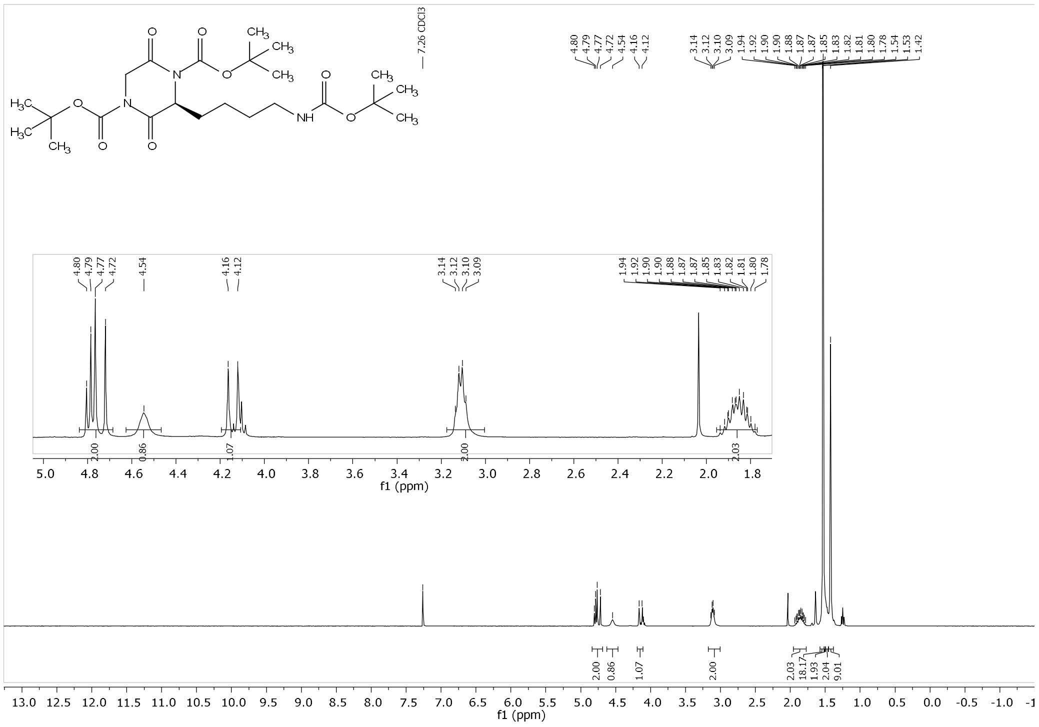

Figure S 112: ^{1}H-NMR-spectrum (400 MHz, $CDCl_3$) of the compound **24**.

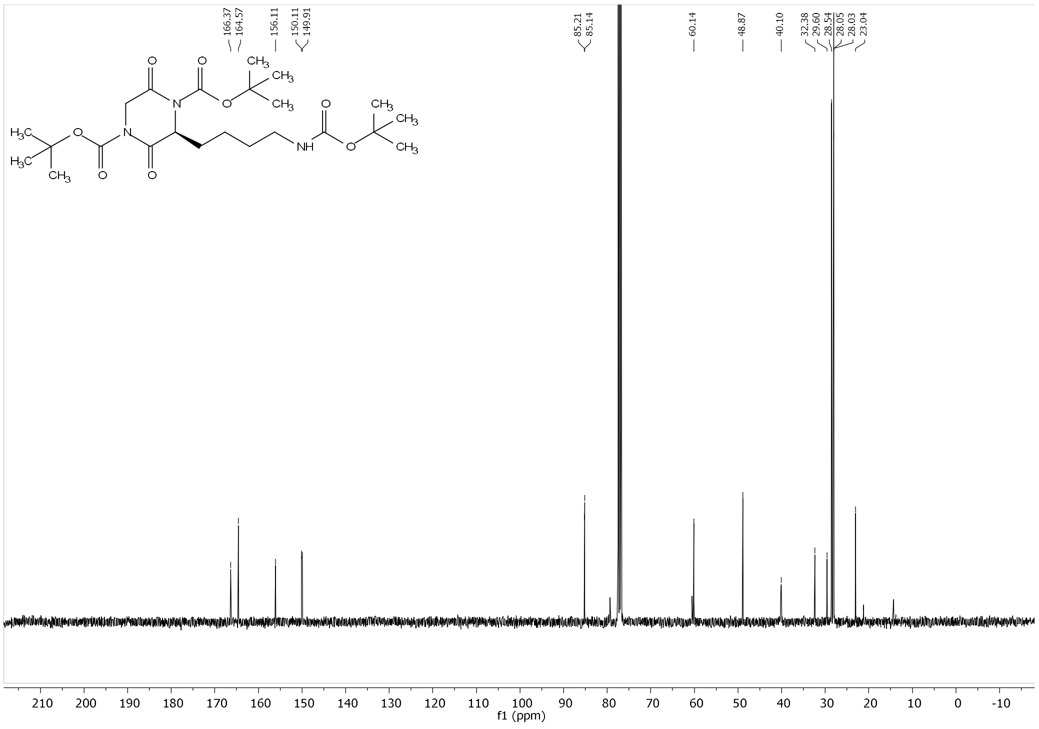

Figure S 113: ^{13}C-NMR-spectrum (101 MHz, $CDCl_3$) of the compound **24**.

Bis-(Boc-cyclo(Lys(Boc))-(2,6-difluoro-4-vinyl-azobenzene) (25)

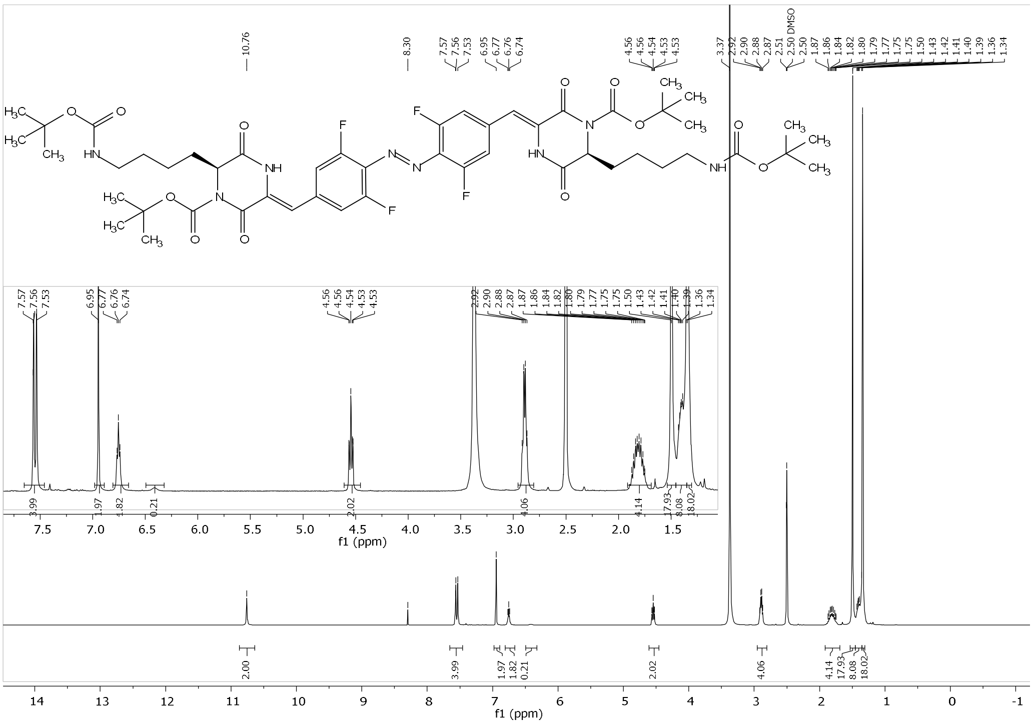

*Figure S 114: ^{1}H-NMR-spectrum (400 MHz, DMSO-d_6) of the compound **25**.*

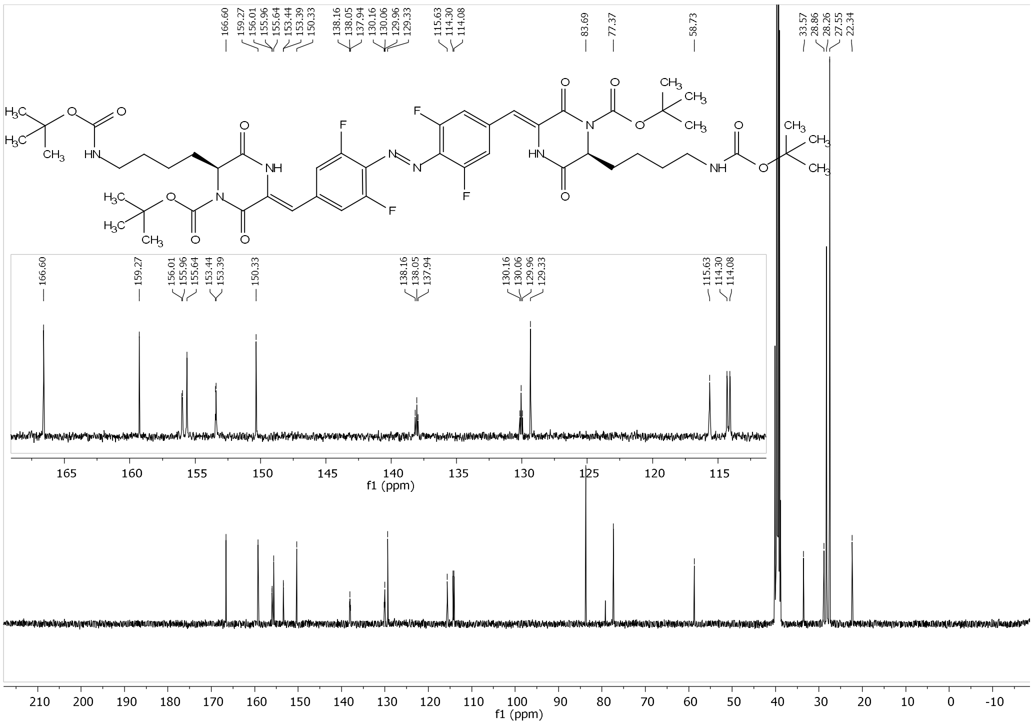

*Figure S 115: ^{13}C-NMR-spectrum (101 MHz, DMSO-d_6) of the compound **25**.*

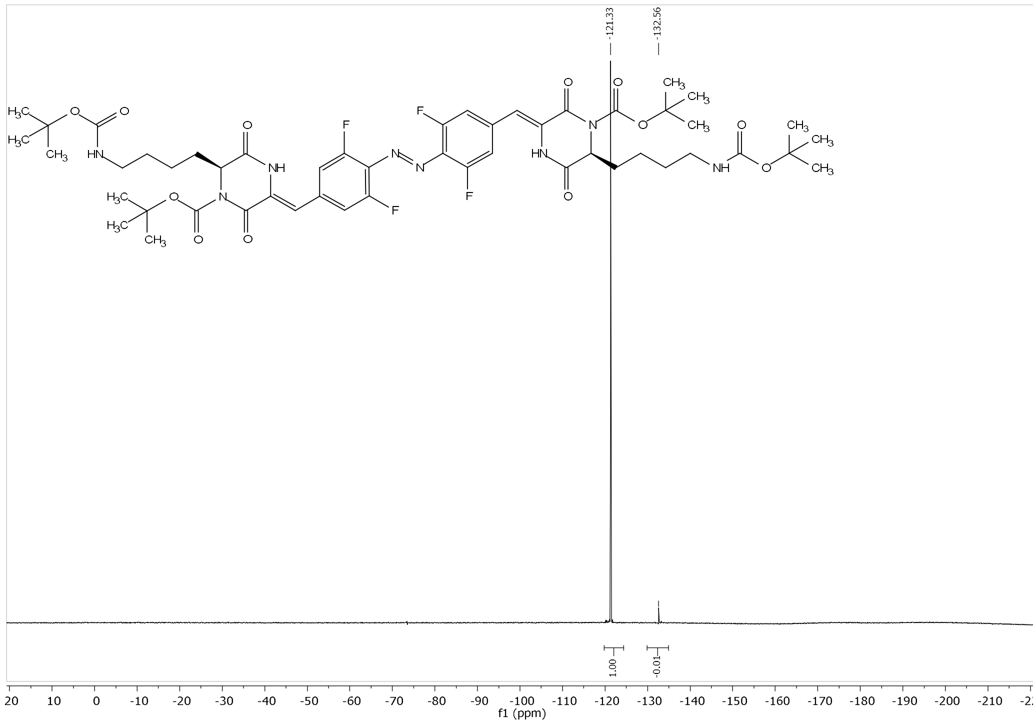

*Figure S 116: ^{19}F NMR spectrum (376 MHz, DMSO) of the compound **25.***

Bis-(cyclo(Lys)-(2,6-difluoro-4-vinyl-azobenzene) (8)

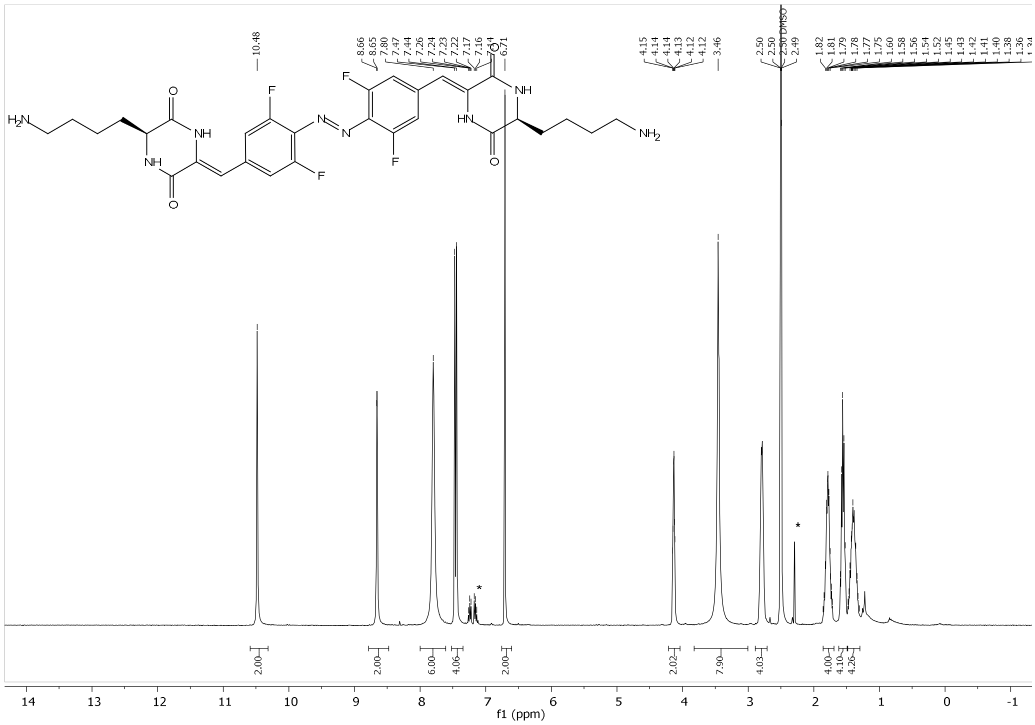

*Figure S 117: ^{1}H-NMR-spectrum (400 MHz, DMSO-d_6) of the compound **8**. *< 1%Toluene.*

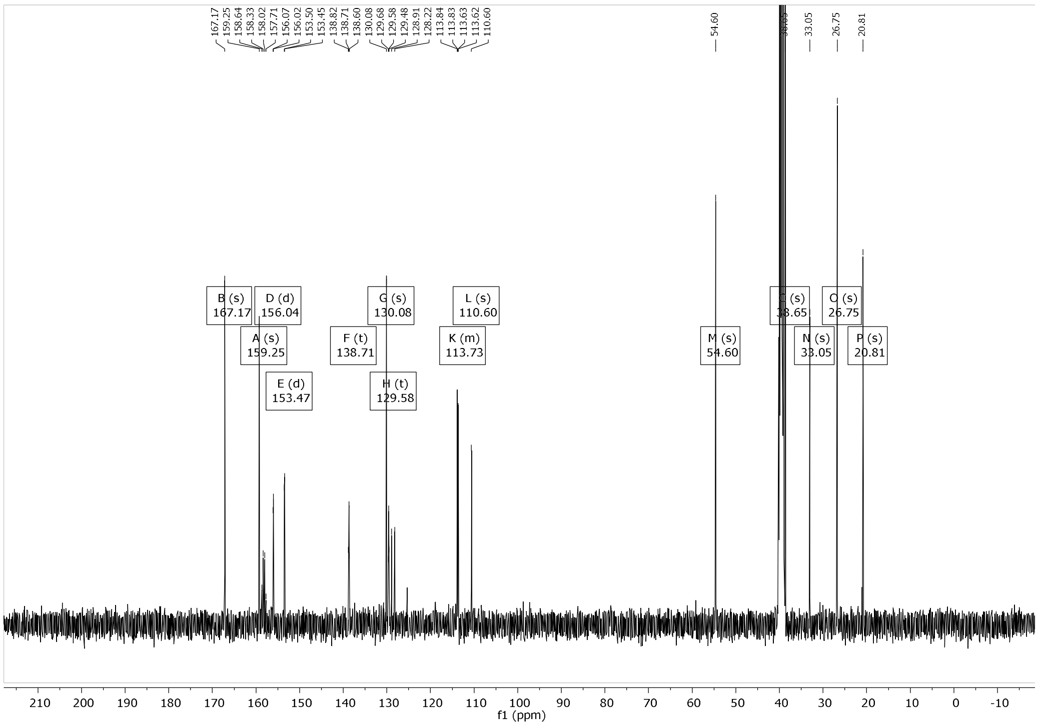

*Figure S 118: ^{13}C-NMR-spectrum (101 MHz, DMSO-d_6) of the compound **8**.*

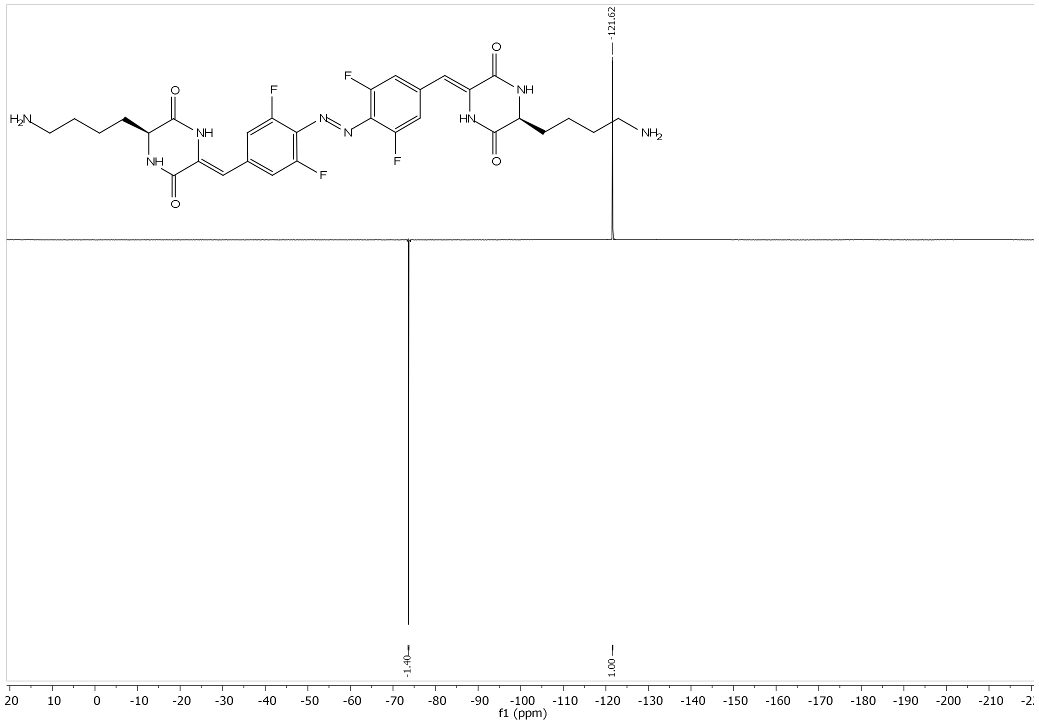

*Figure S 119: ^{19}F NMR spectrum (376 MHz, DMSO) of the compound **8.***

12. Photoisomerization Experiments

The following ^{1}H NMR spectra were recorded in *d*^{3}-MeCN on a BRUKER 300 (300 MHz).

*NMR spectra of **TFAB** after irradiation*

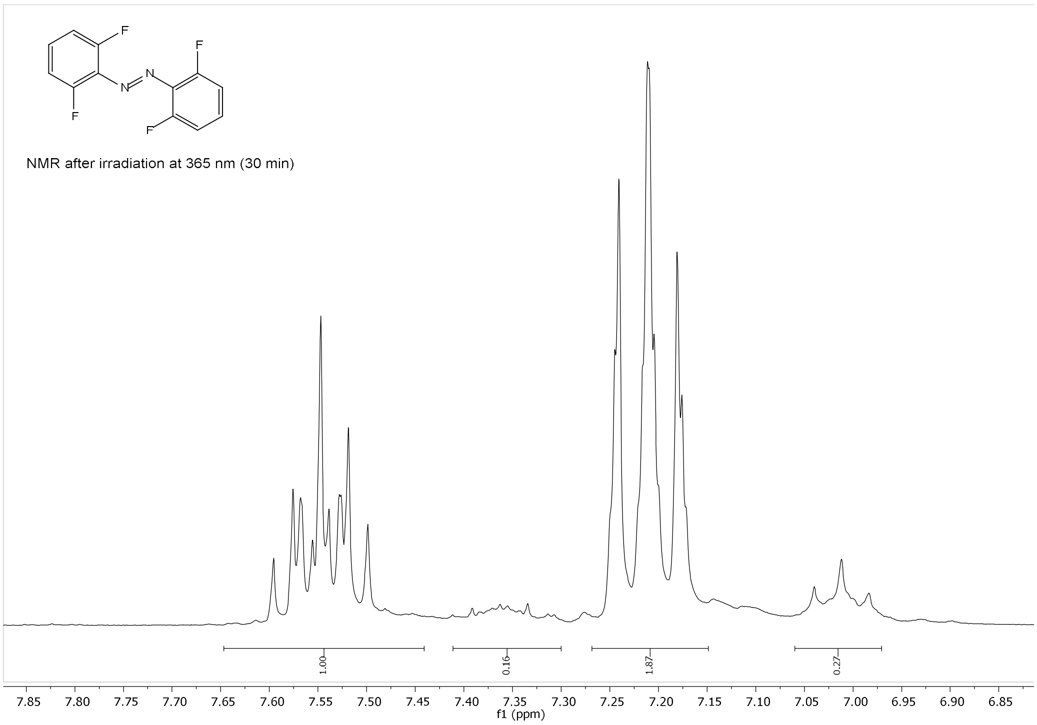

Figure S 120

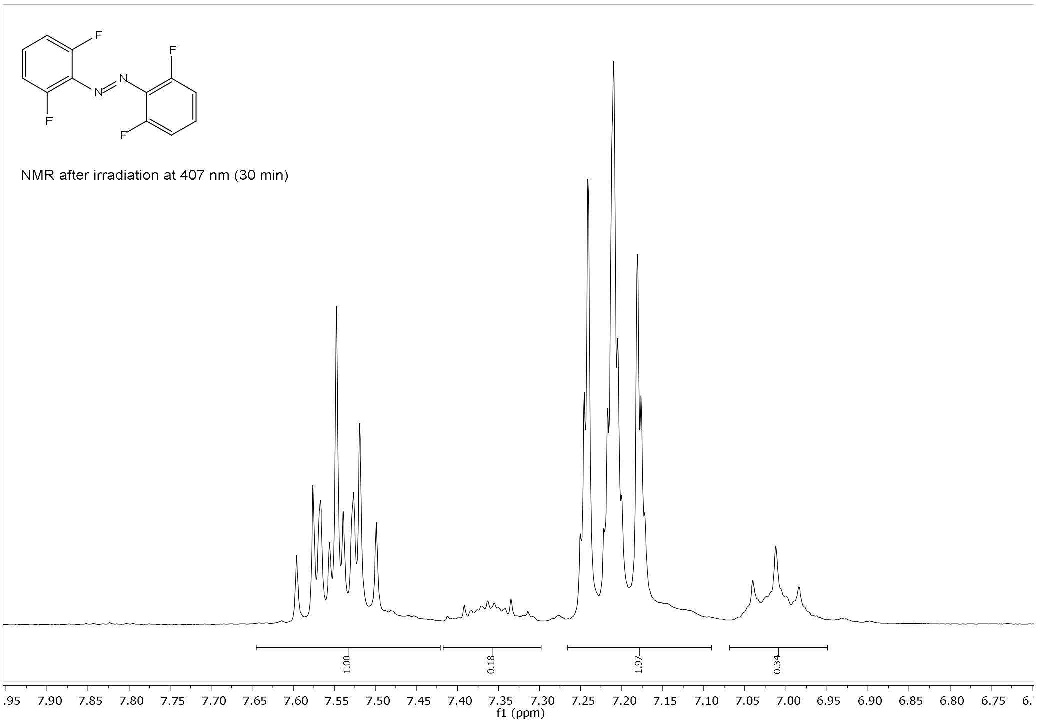

Figure S 121

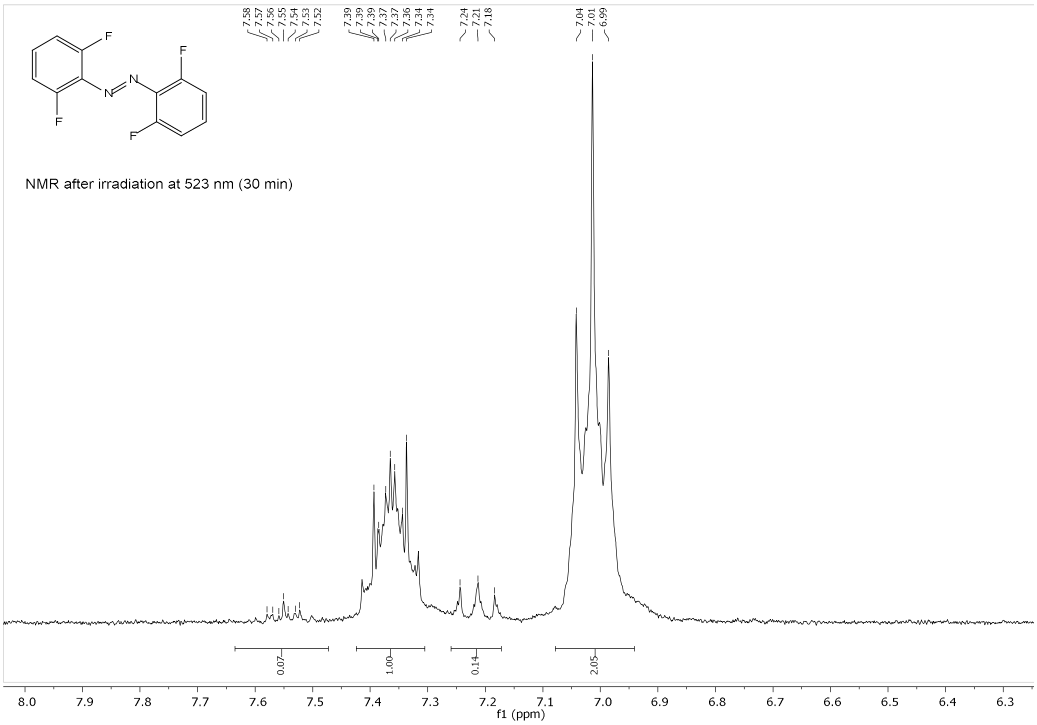

Figure S 122

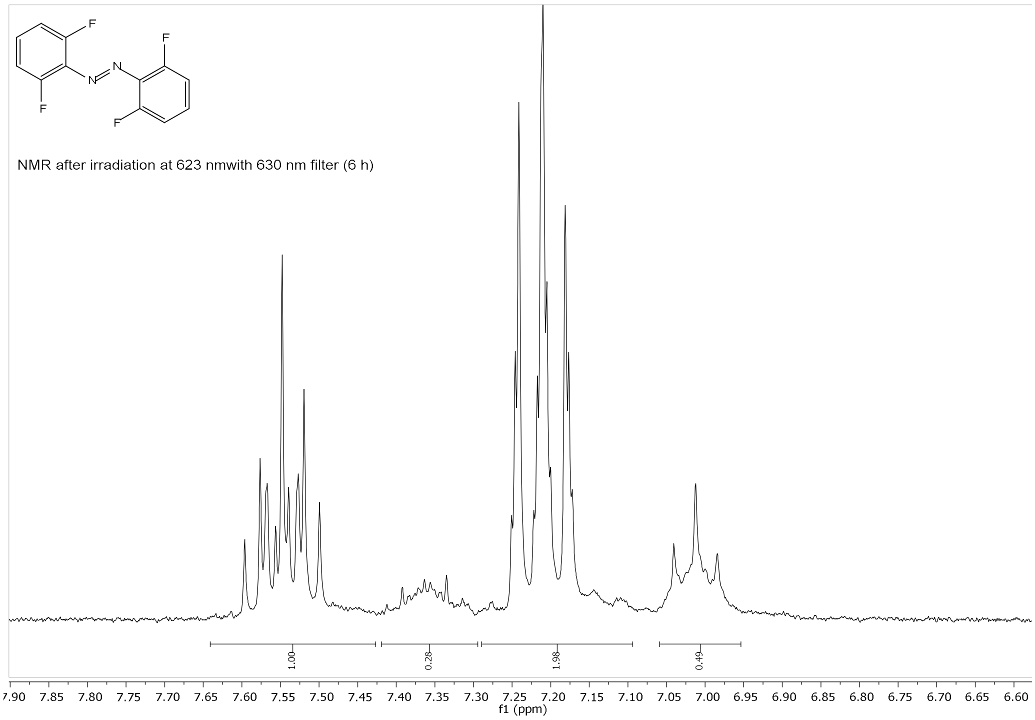

Figure S 123

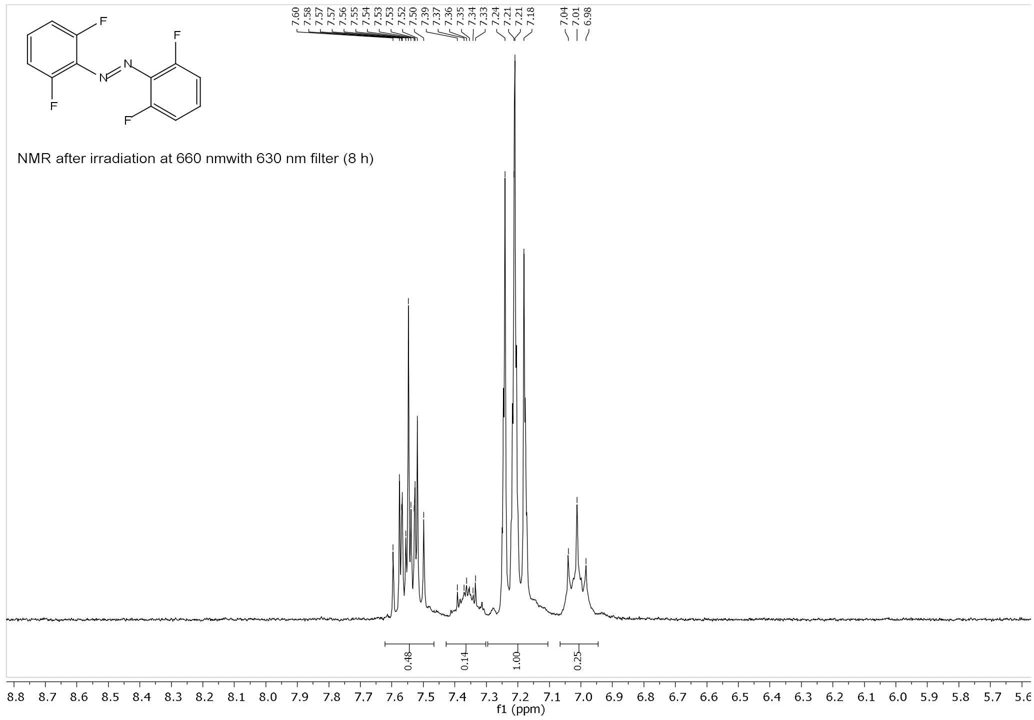

Figure S 124

*NMR spectra of **2** after irradiation*

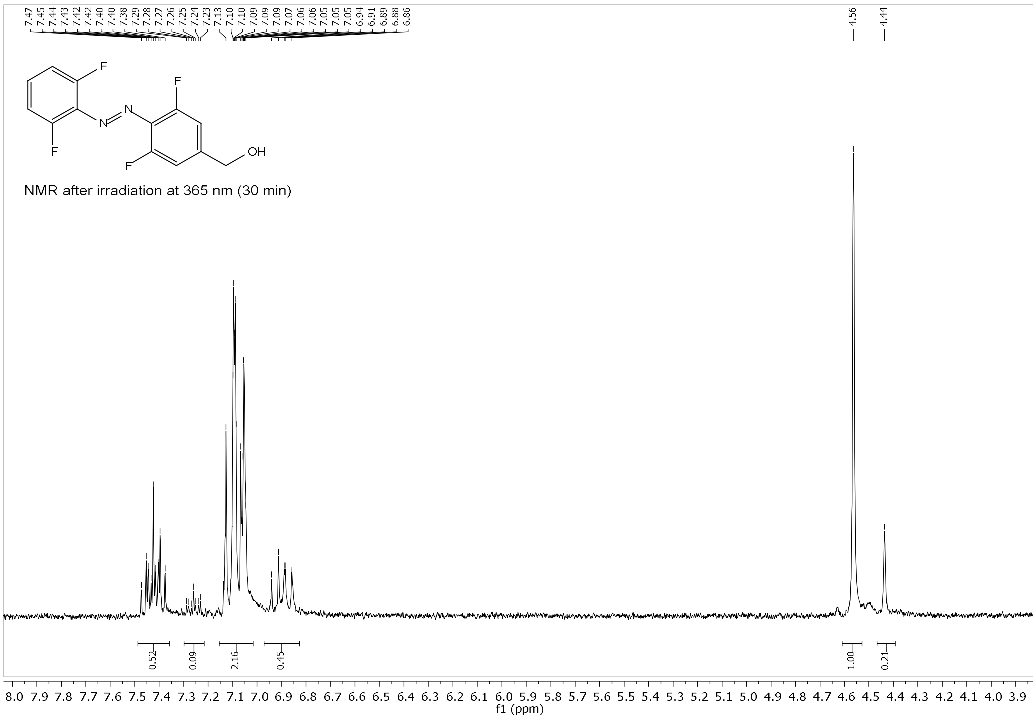

Figure S 125

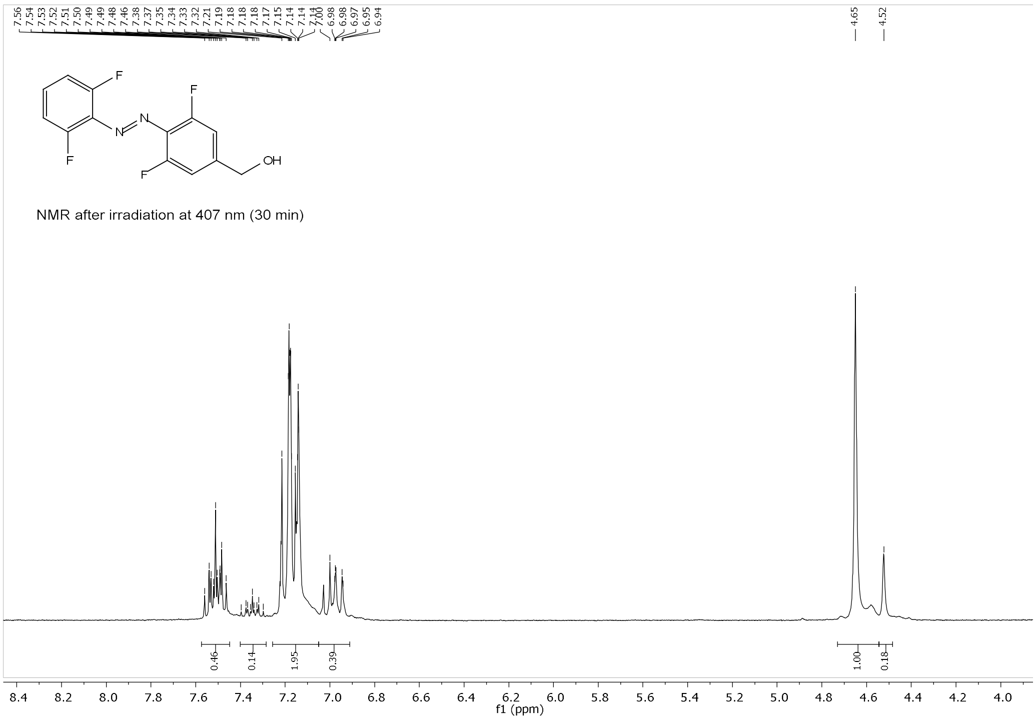

Figure S 126

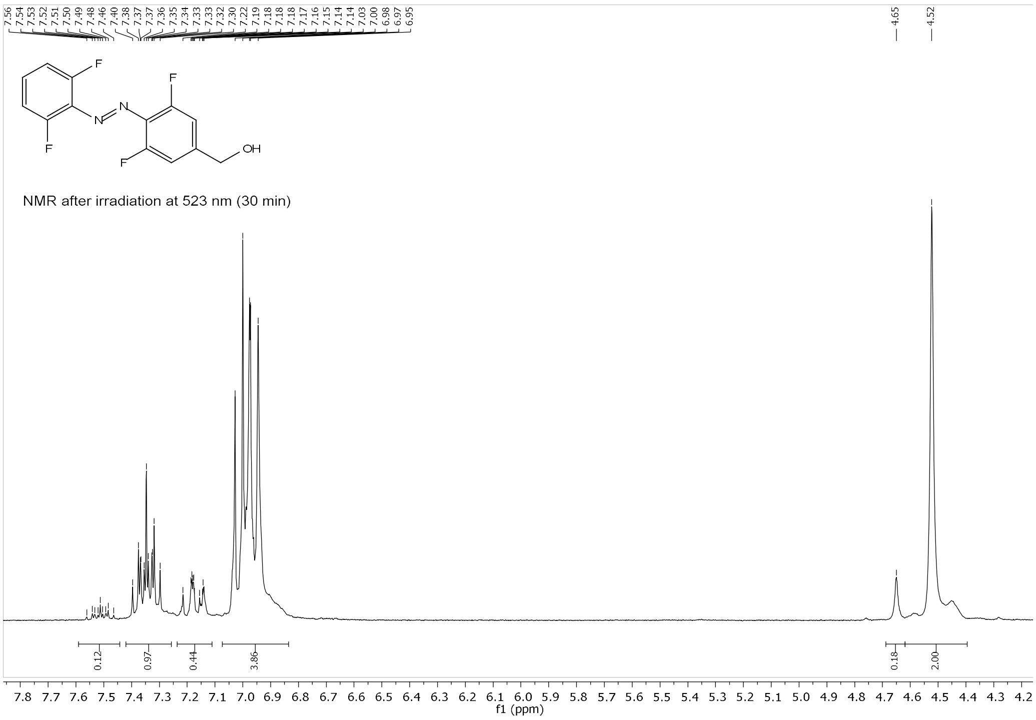

Figure S 127

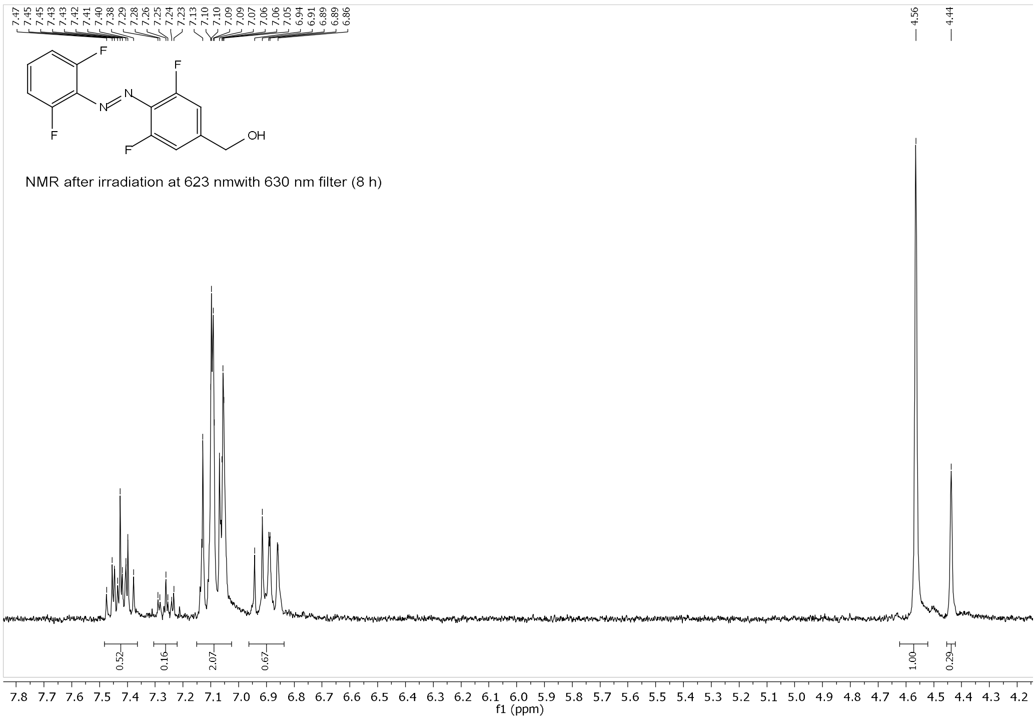

Figure S 128

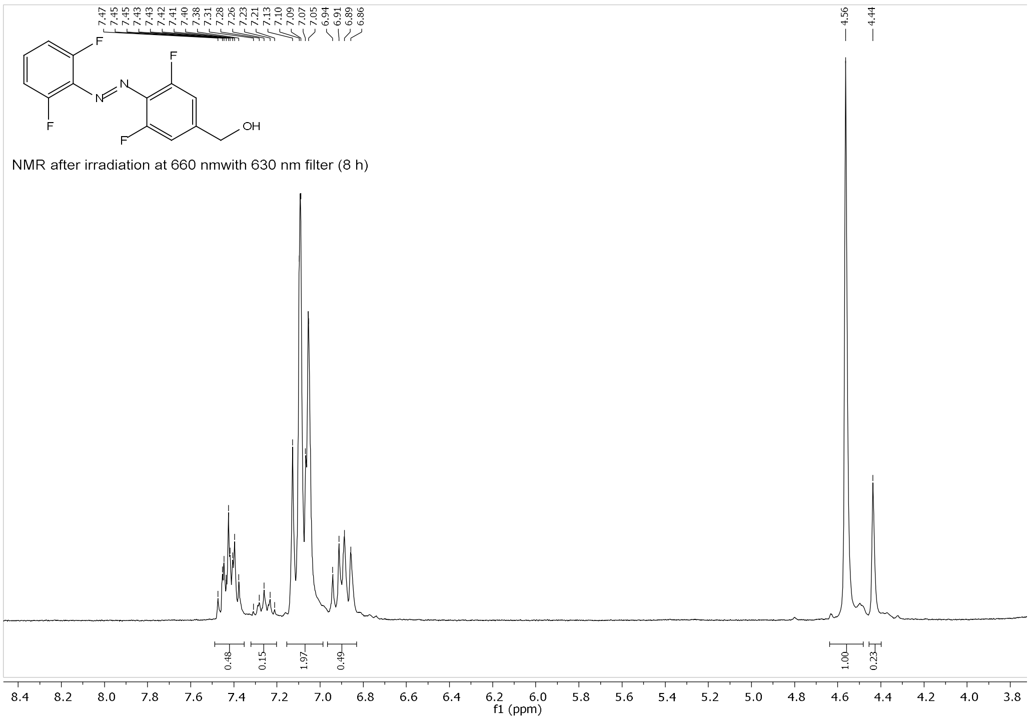

Figure S 129

*NMR spectra of **3** after irradiation*

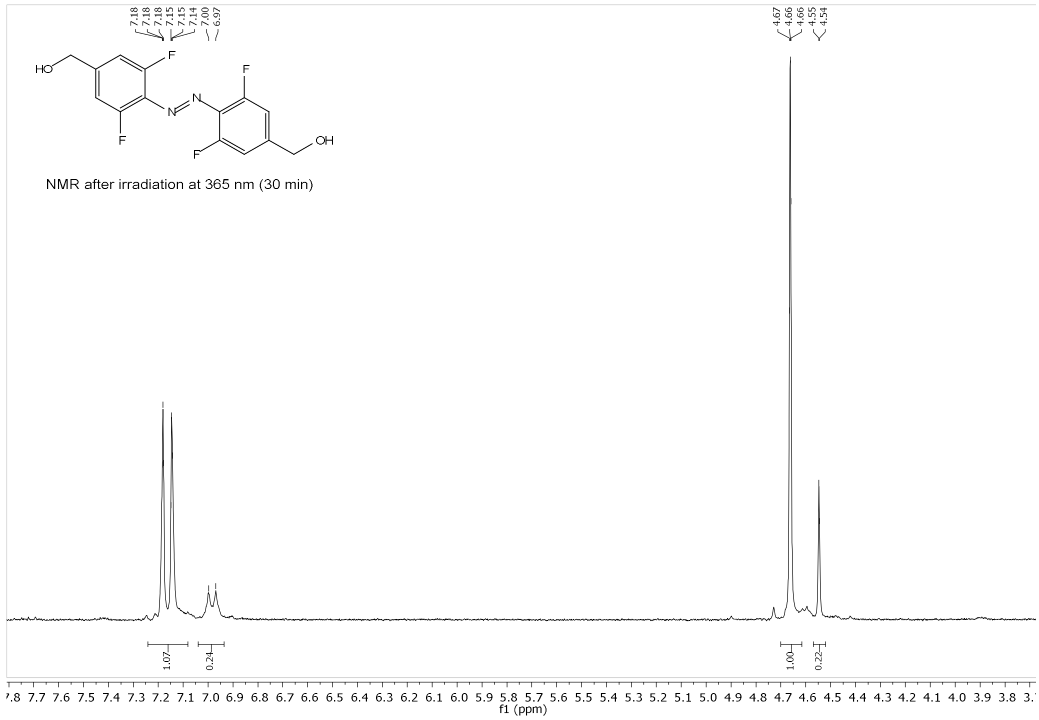

Figure S 130

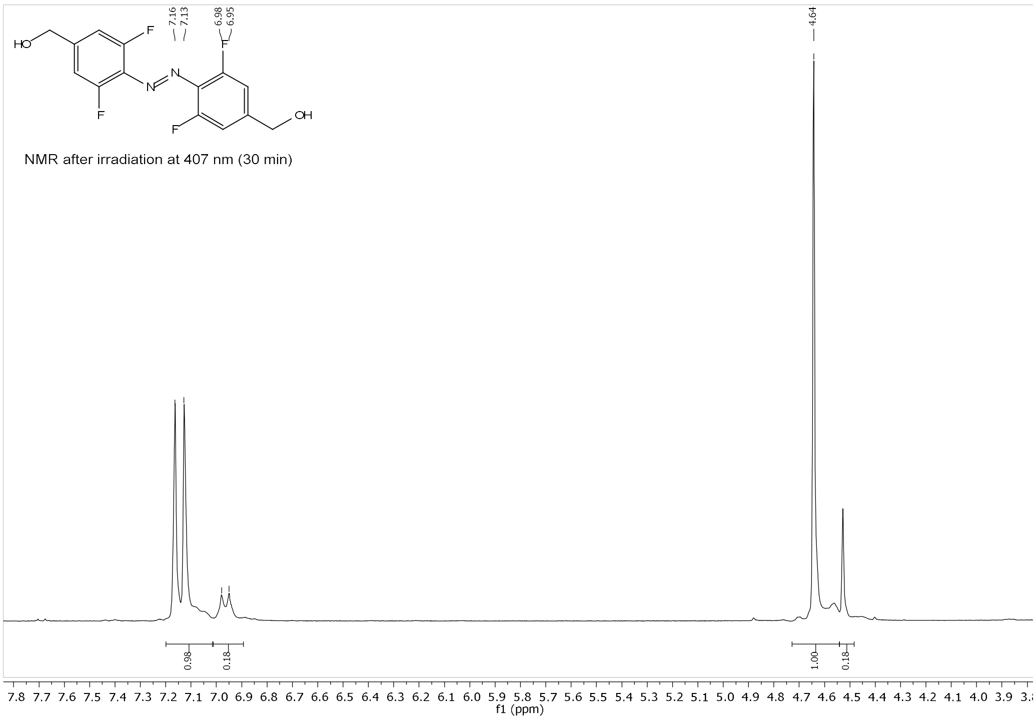

Figure S 131

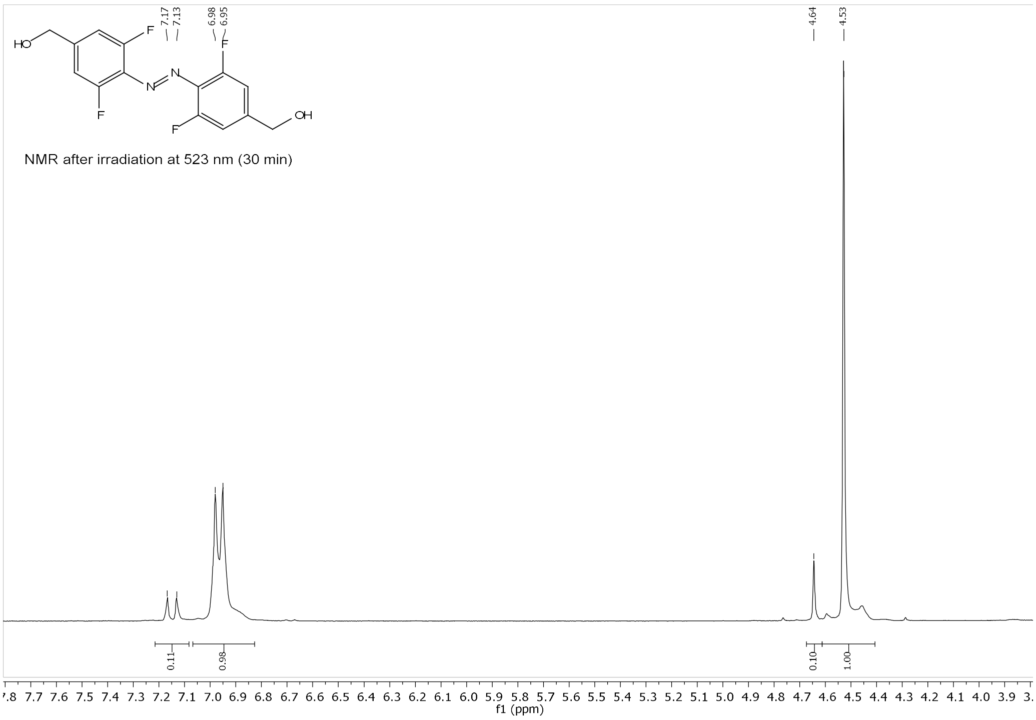

Figure S 132

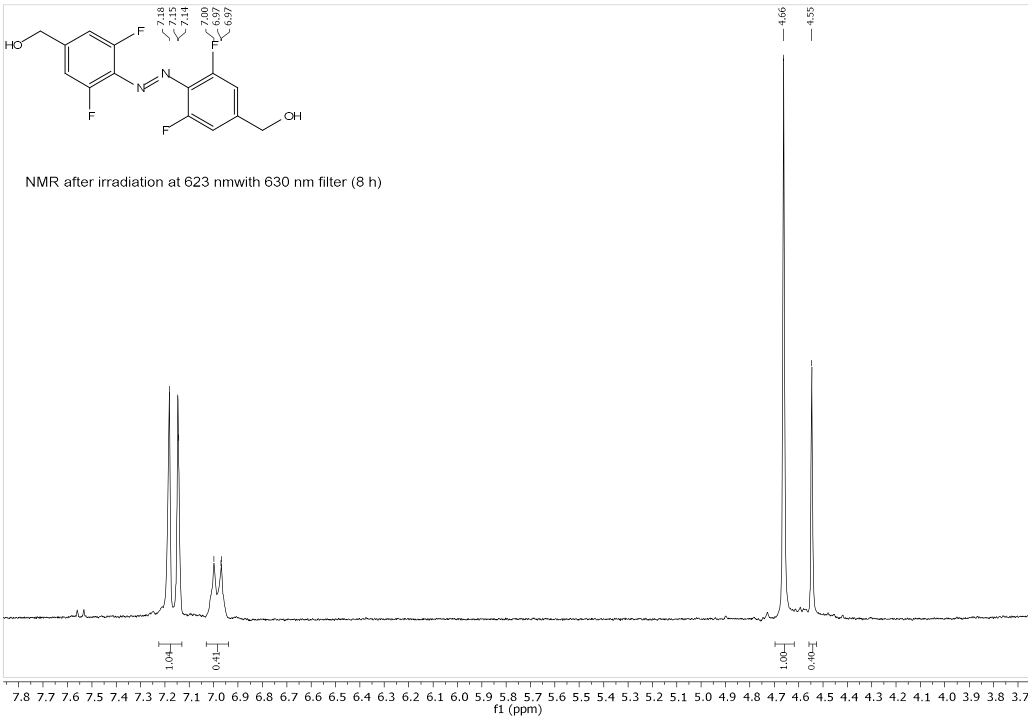

Figure S 133

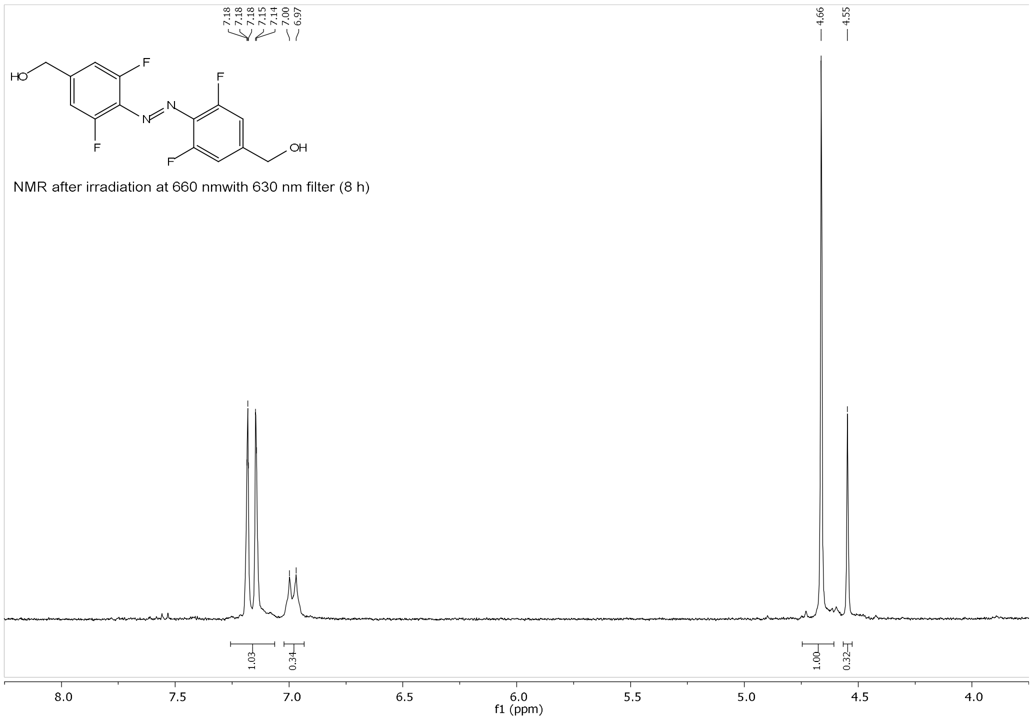

Figure S 134

*NMR spectra of **4** after irradiation*

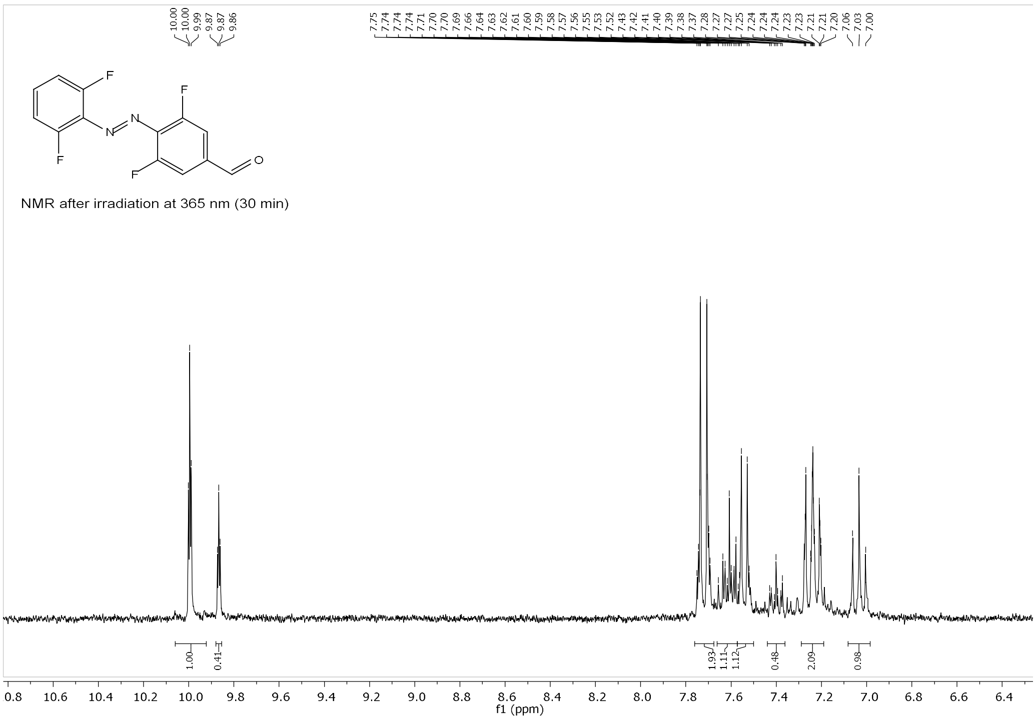

Figure S 135

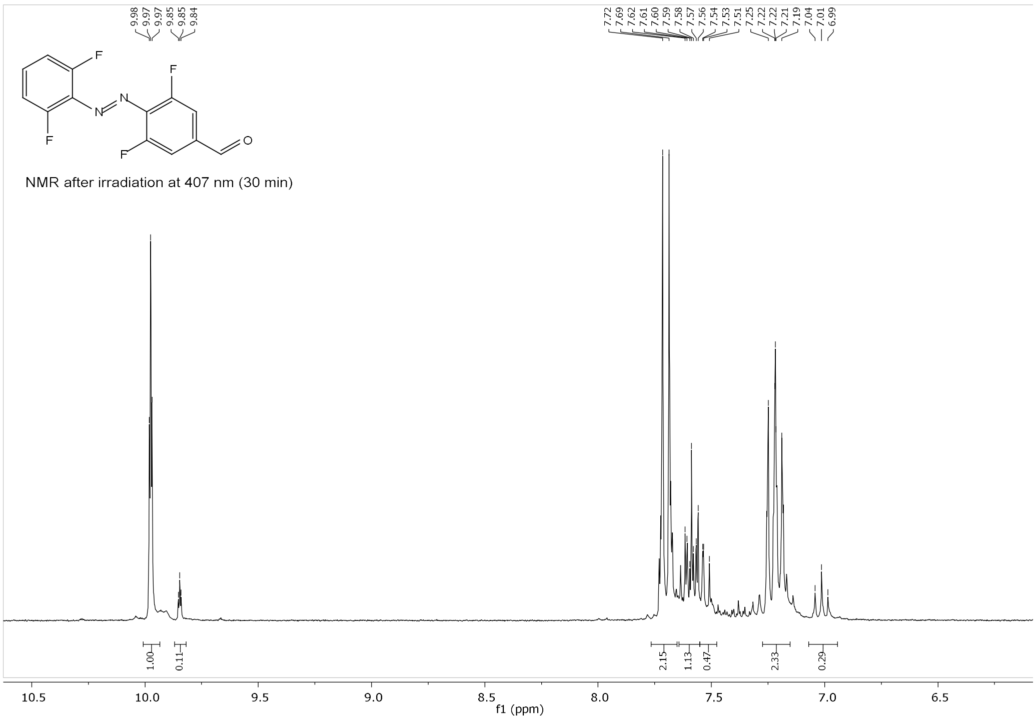

Figure S 136

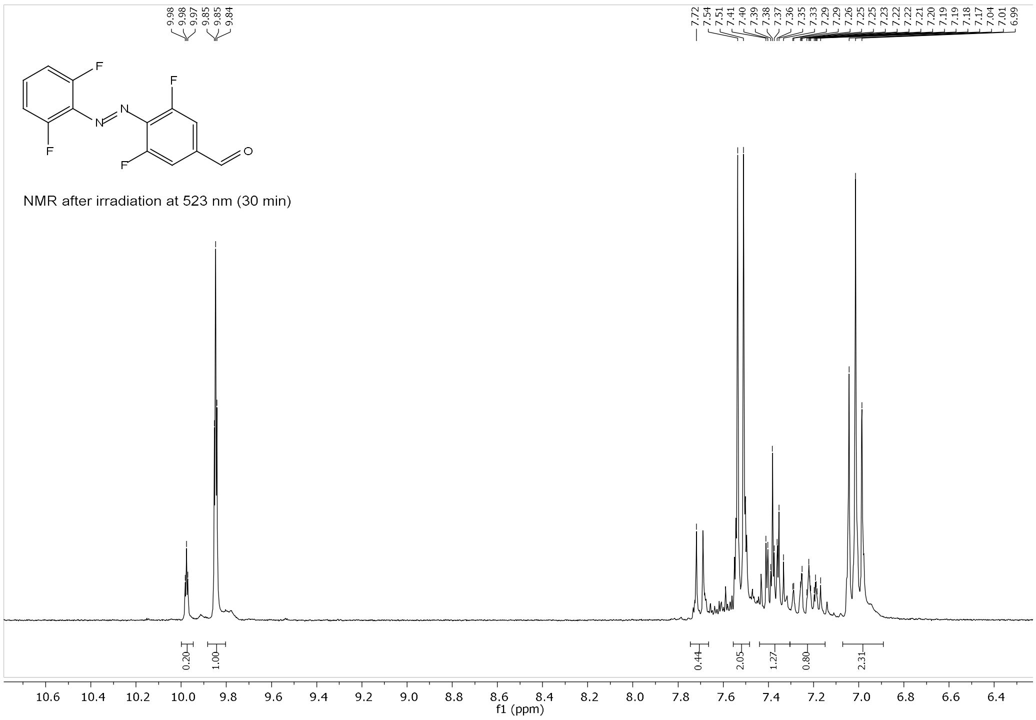

Figure S 137

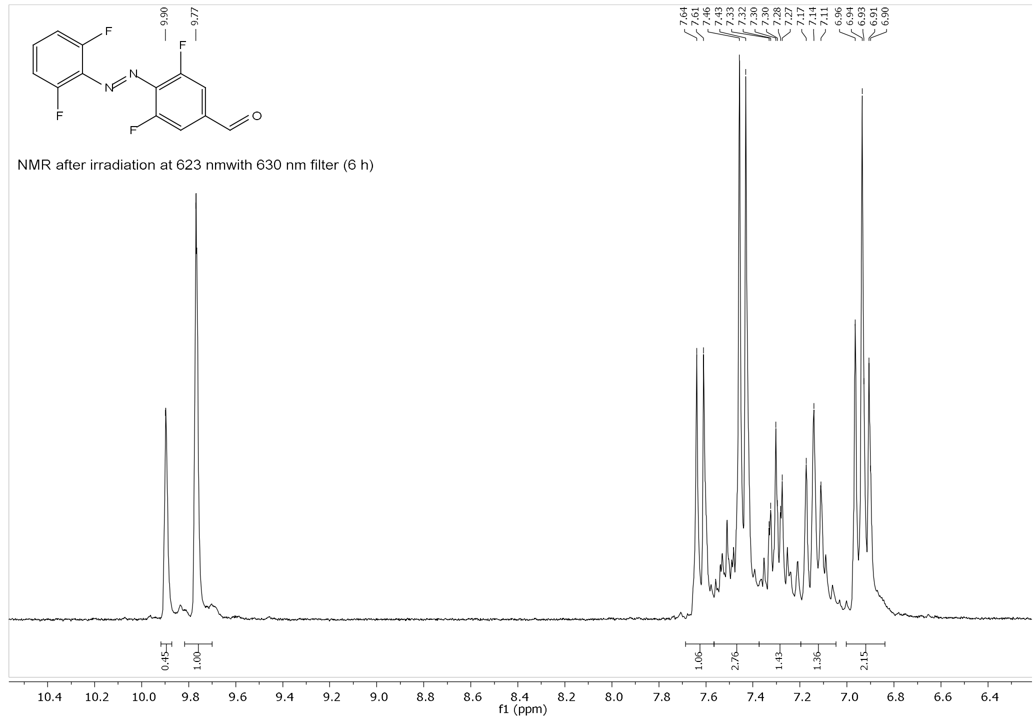

Figure S 138

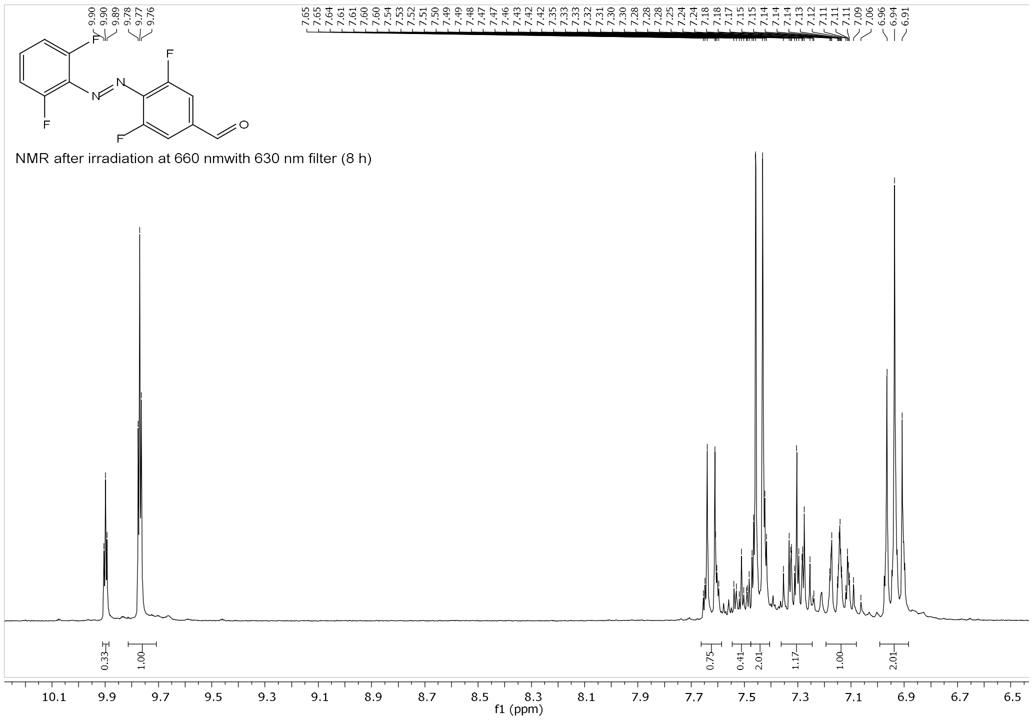

Figure S 139

*NMR spectra of **5** after irradiation*

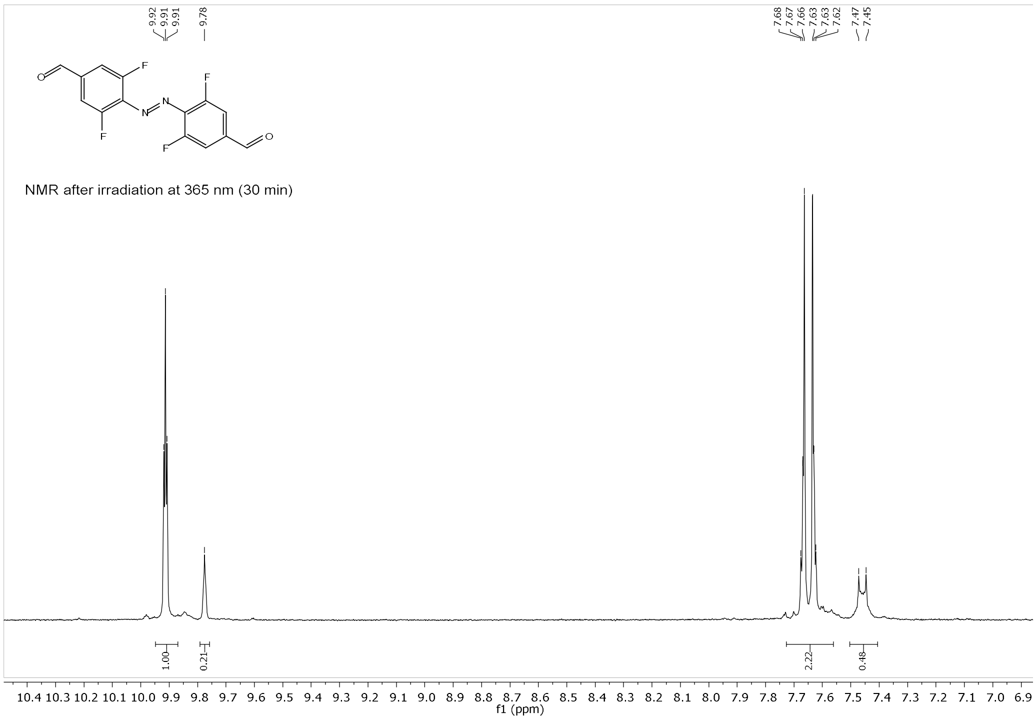

Figure S 140

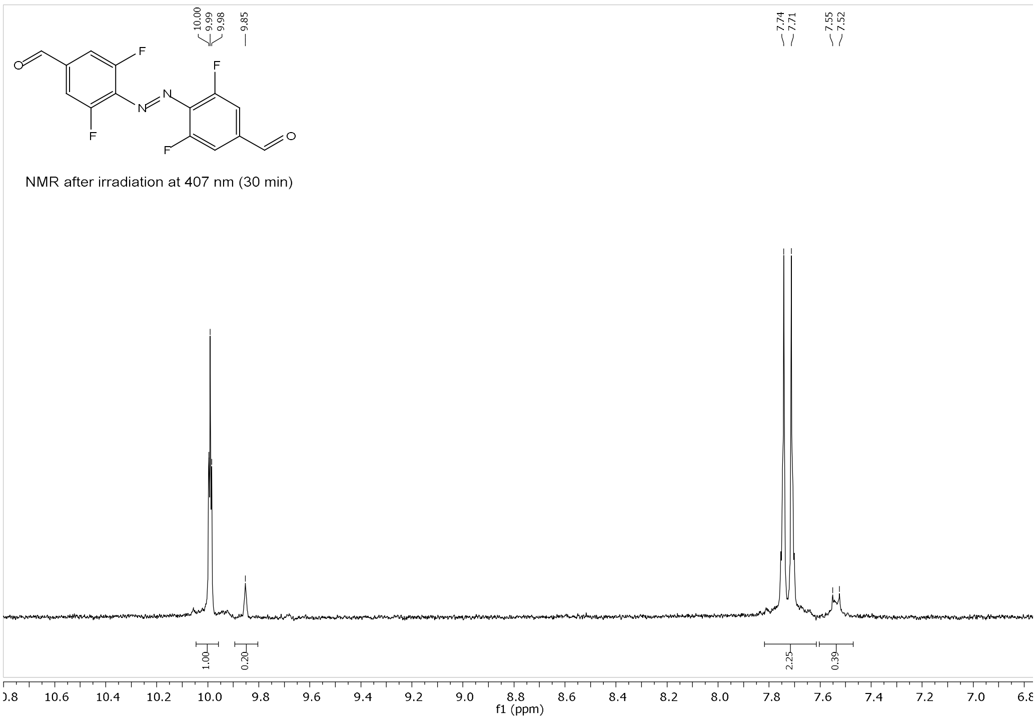

Figure S 141

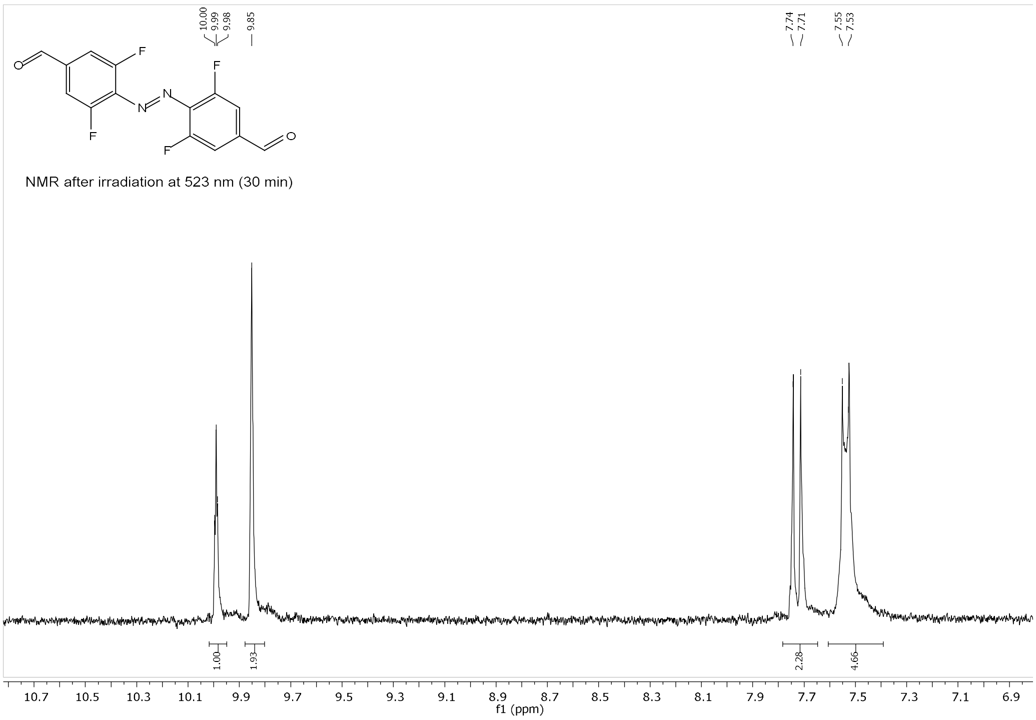

Figure S 142

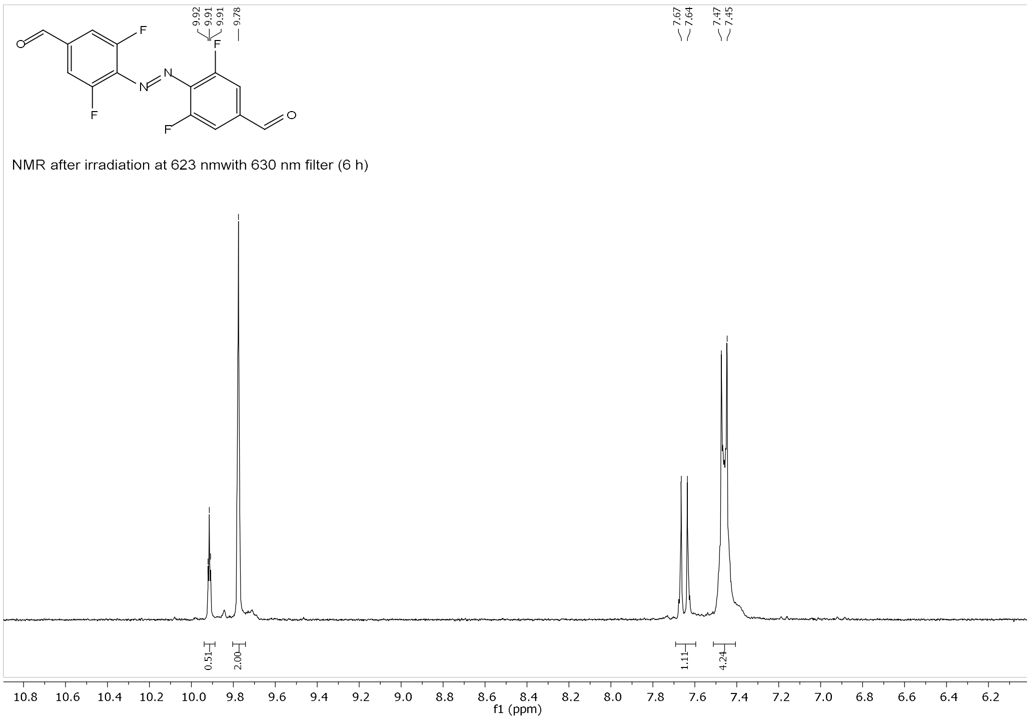
NMR after irradiation at 623 nmwith 630 nm filter (6 h)
f1 (ppm)

Figure S 143

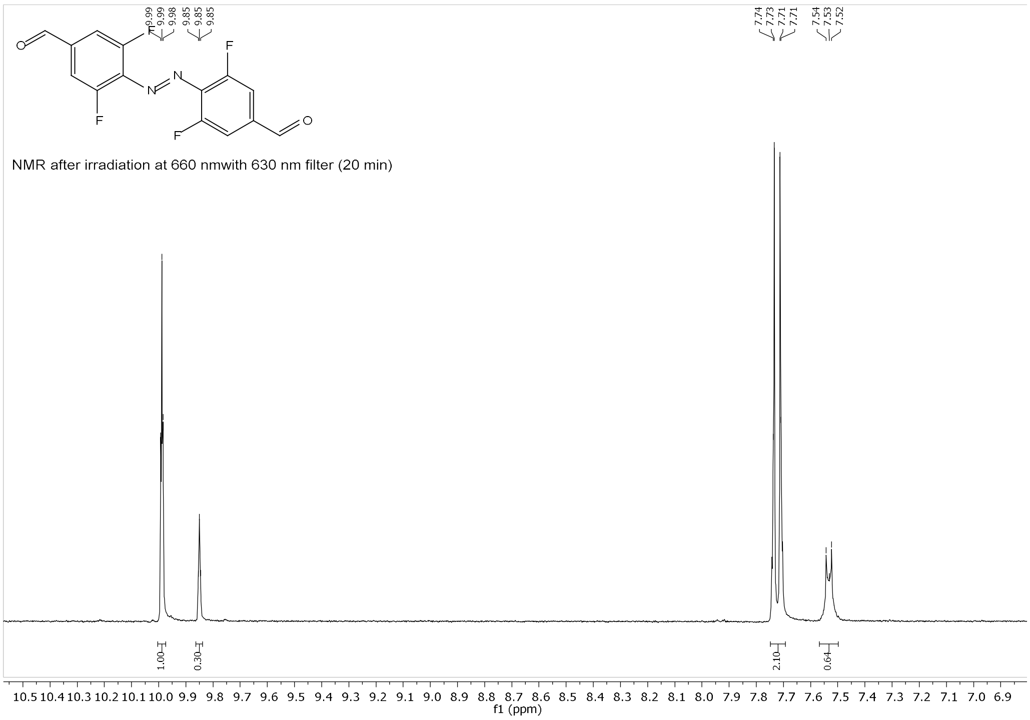
NMR after irradiation at 660 nmwith 630 nm filter (20 min)
f1 (ppm)

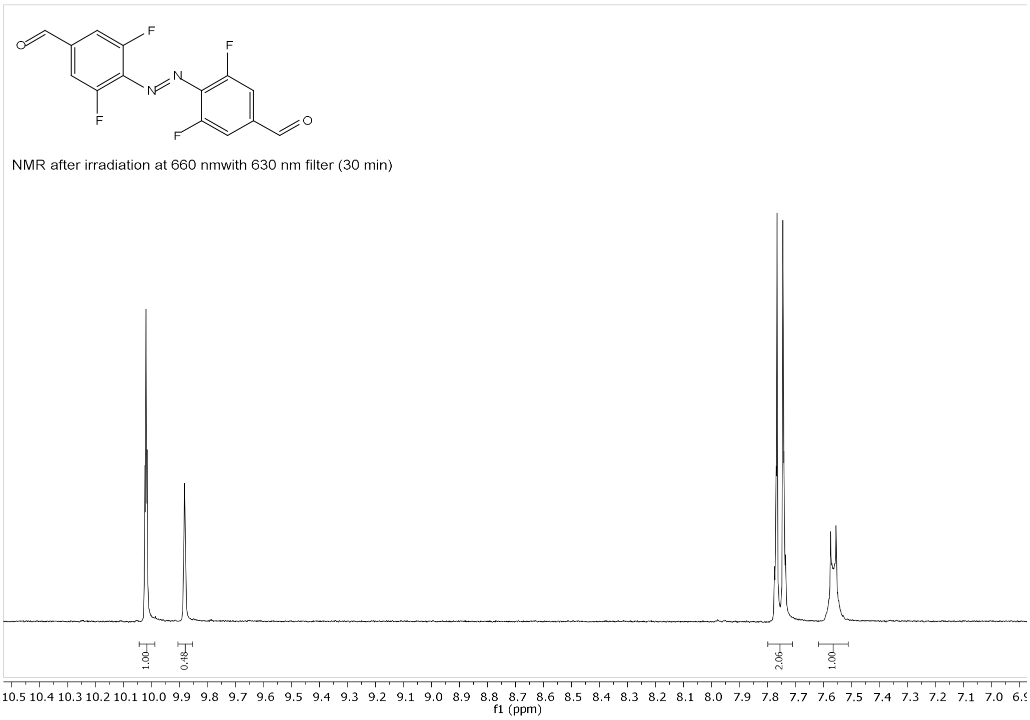
F
F
N
N
F
F
O
O
NMR after irradiation at 660 nmwith 630 nm filter (30 min)
1.00
0.48
2.06
1.00
10.5 10.4 10.3 10.2 10.1 10.0 9.9 9.8 9.7 9.6 9.5 9.4 9.3 9.2 9.1 9.0 8.9 8.8 8.7 8.6 8.5 8.4 8.3 8.2 8.1 8.0 7.9 7.8 7.7 7.6 7.5 7.4 7.3 7.2 7.1 7.0
f1 (ppm)

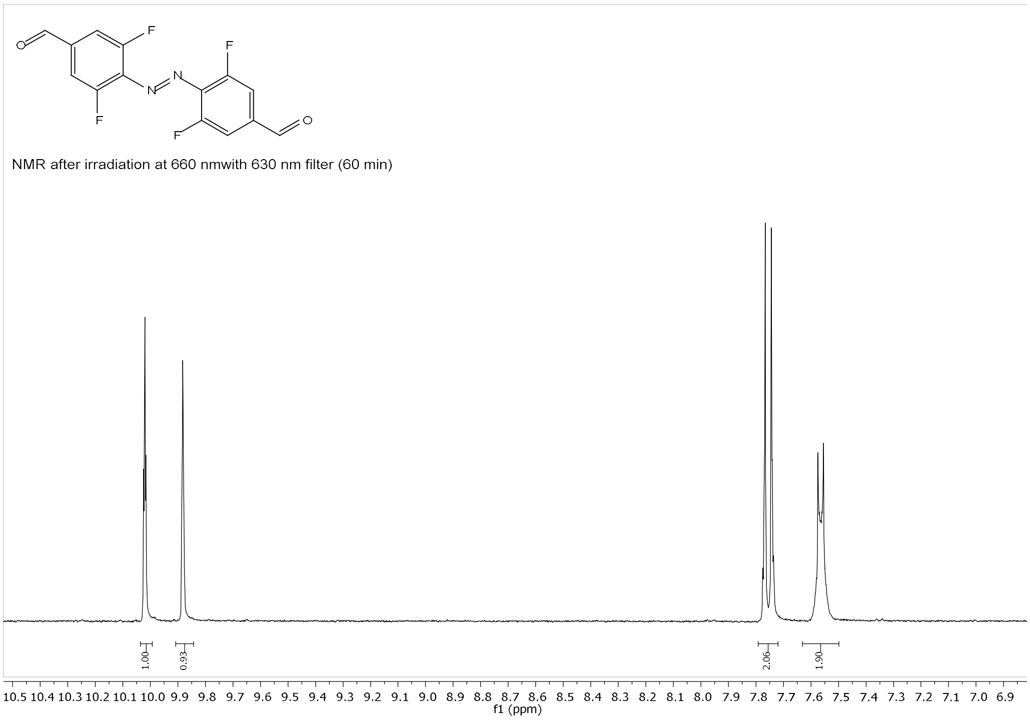
F
F
N
N
F
F
O
O
NMR after irradiation at 660 nmwith 630 nm filter (60 min)
1.00
0.93
2.06
1.90
10.5 10.4 10.3 10.2 10.1 10.0 9.9 9.8 9.7 9.6 9.5 9.4 9.3 9.2 9.1 9.0 8.9 8.8 8.7 8.6 8.5 8.4 8.3 8.2 8.1 8.0 7.9 7.8 7.7 7.6 7.5 7.4 7.3 7.2 7.1 7.0 6.9
f1 (ppm)

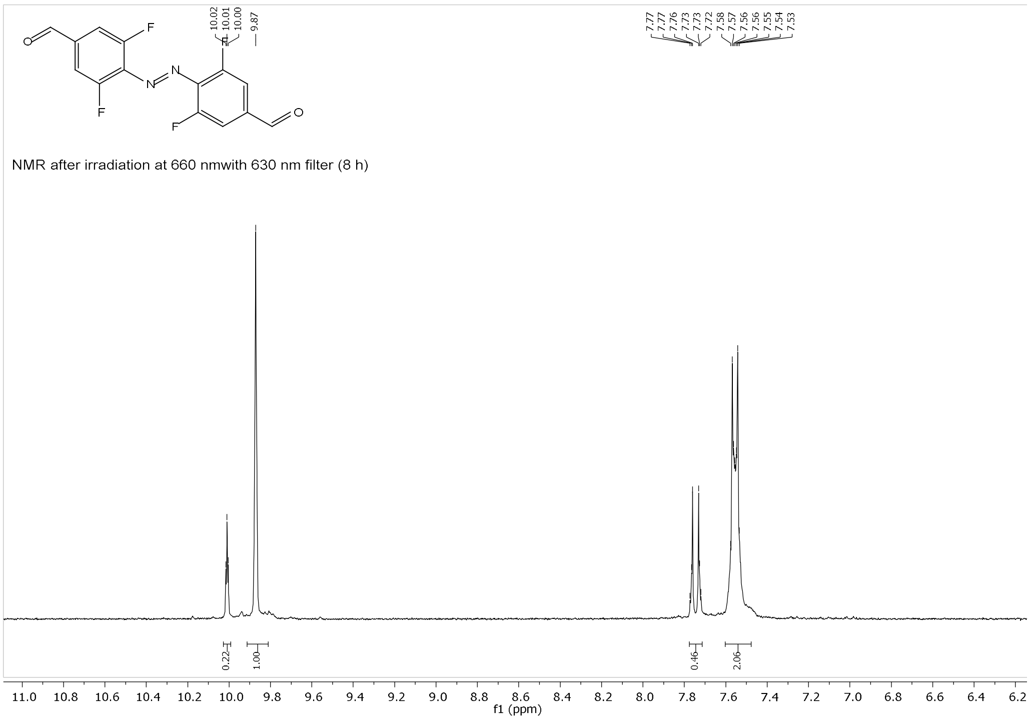

Figure S 144

*NMR spectra of **6** after irradiation*

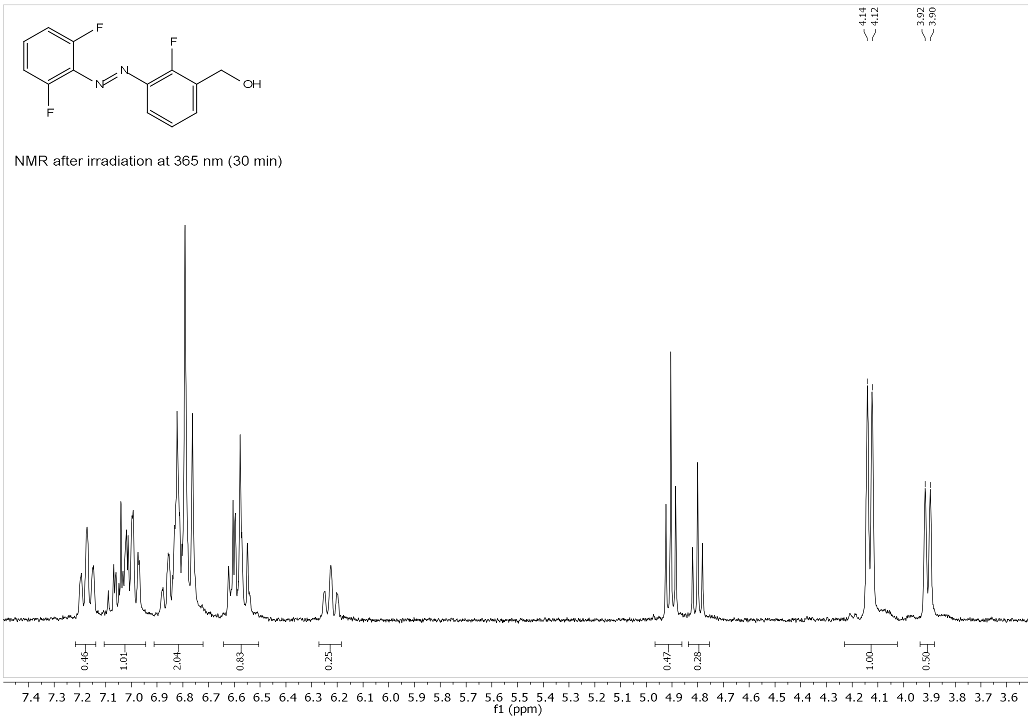

Figure S 145

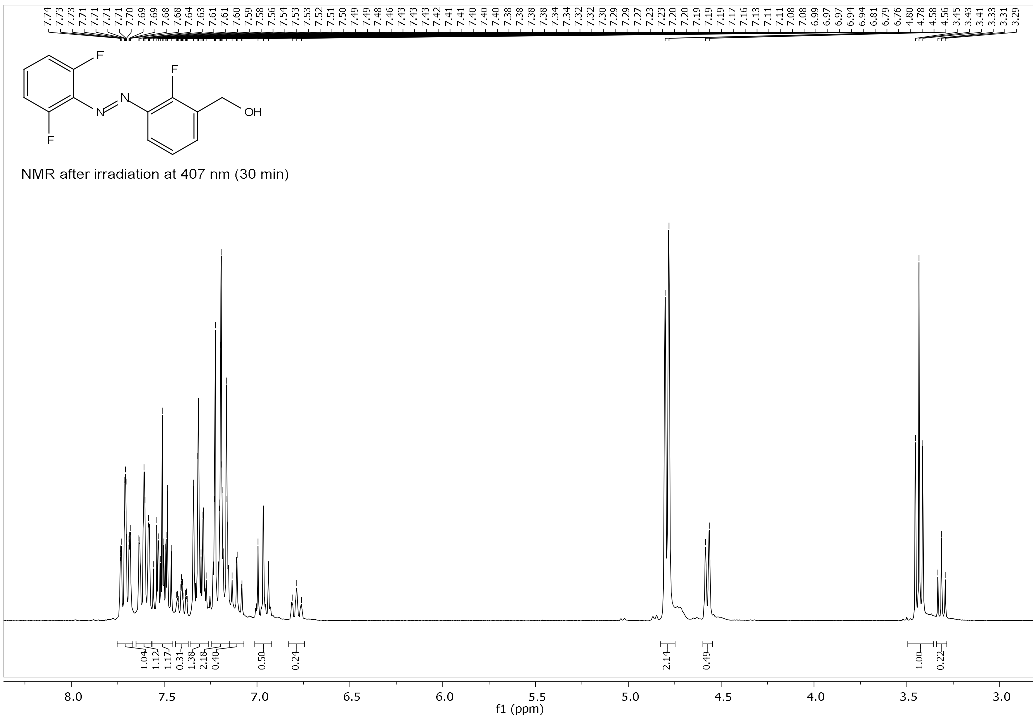

Figure S 146

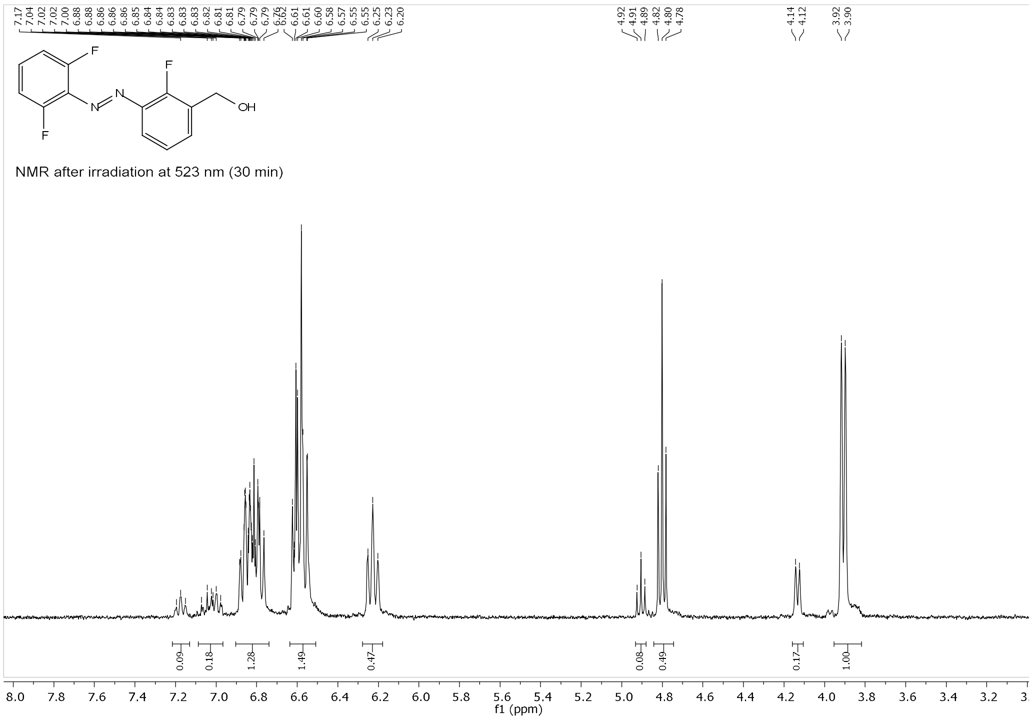

Figure S 147

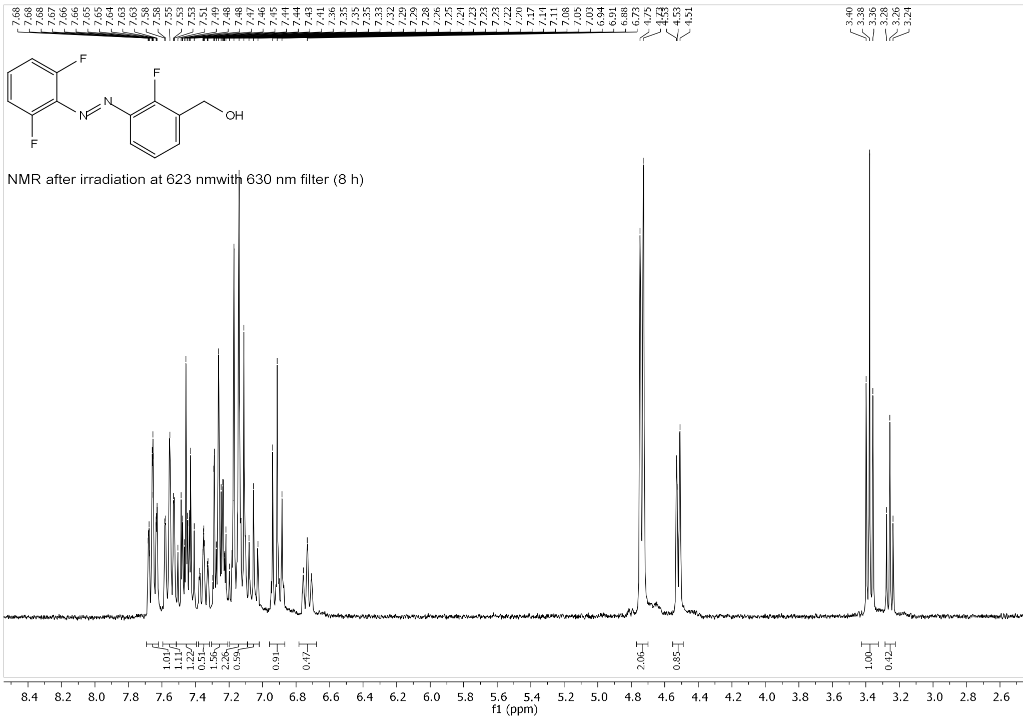

Figure S 148

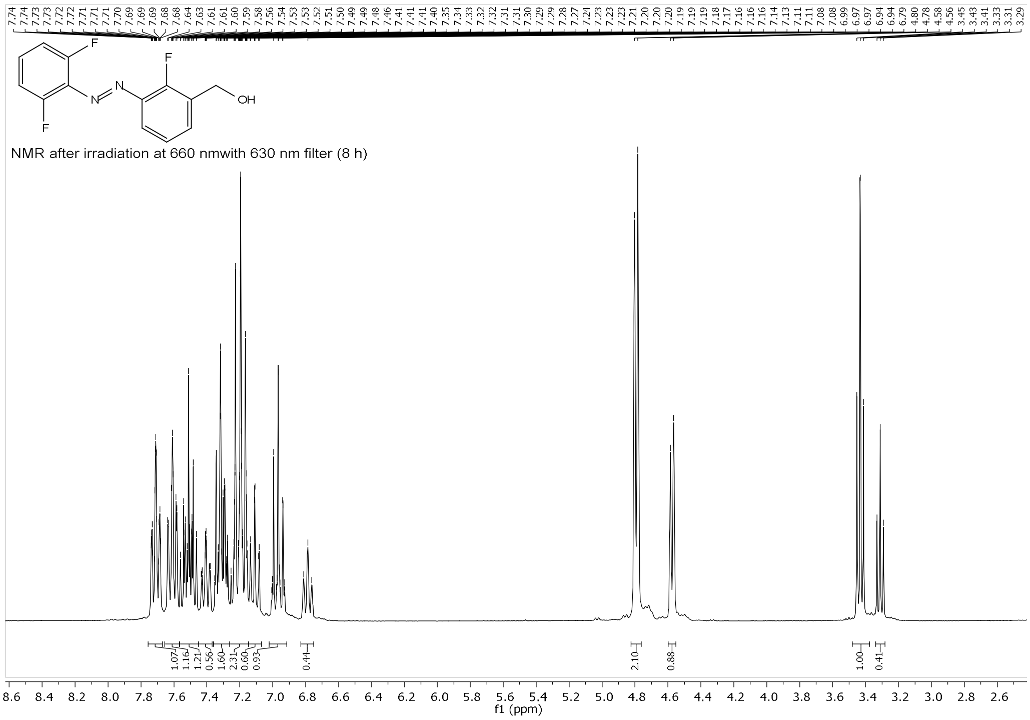

Figure S 149

*NMR spectra of **7** after irradiation*

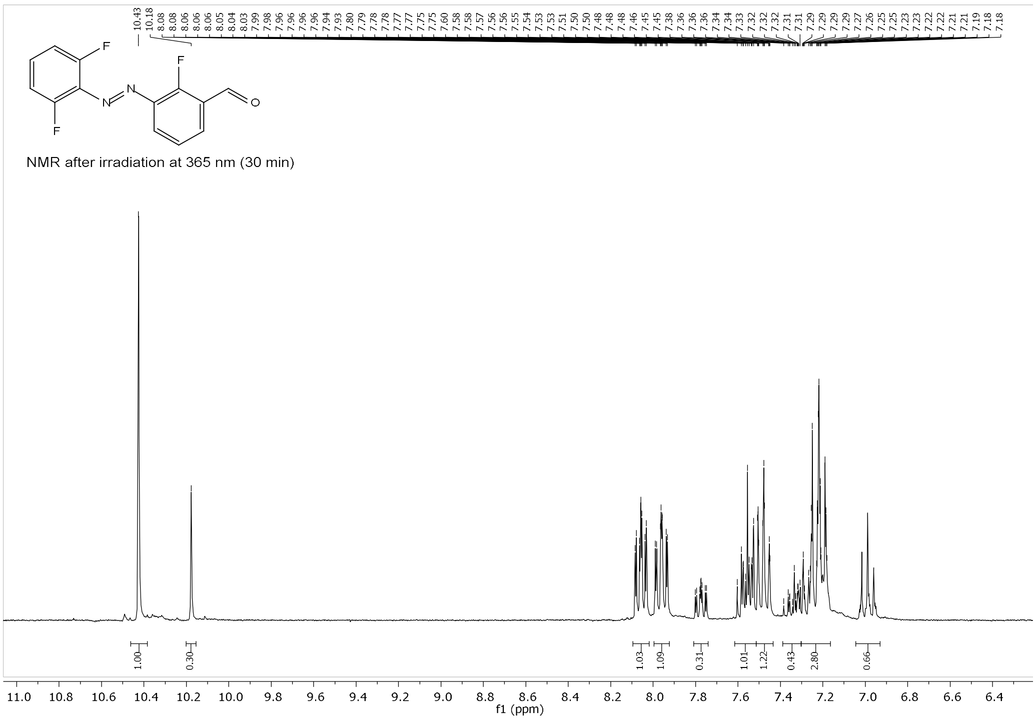

Figure S 150

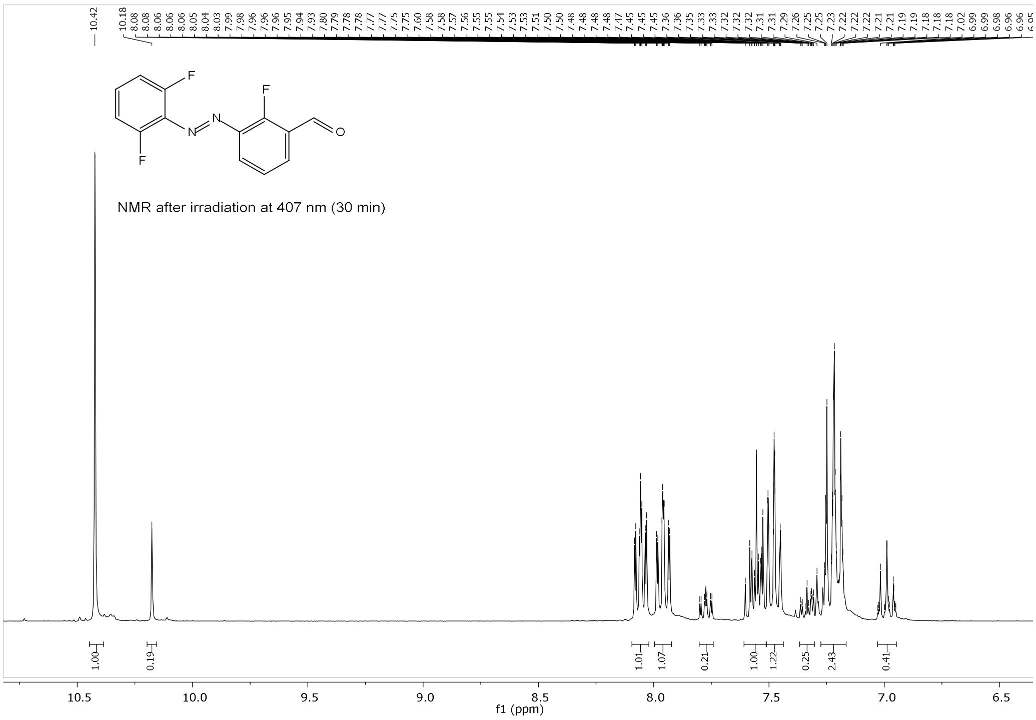

Figure S 151

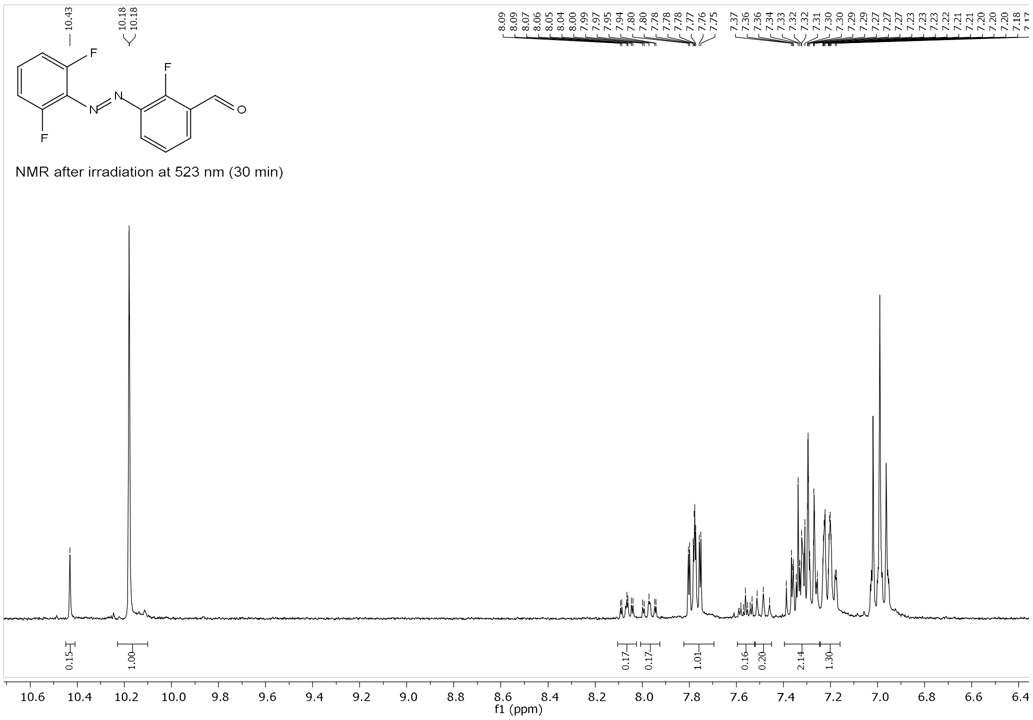

Figure S 152

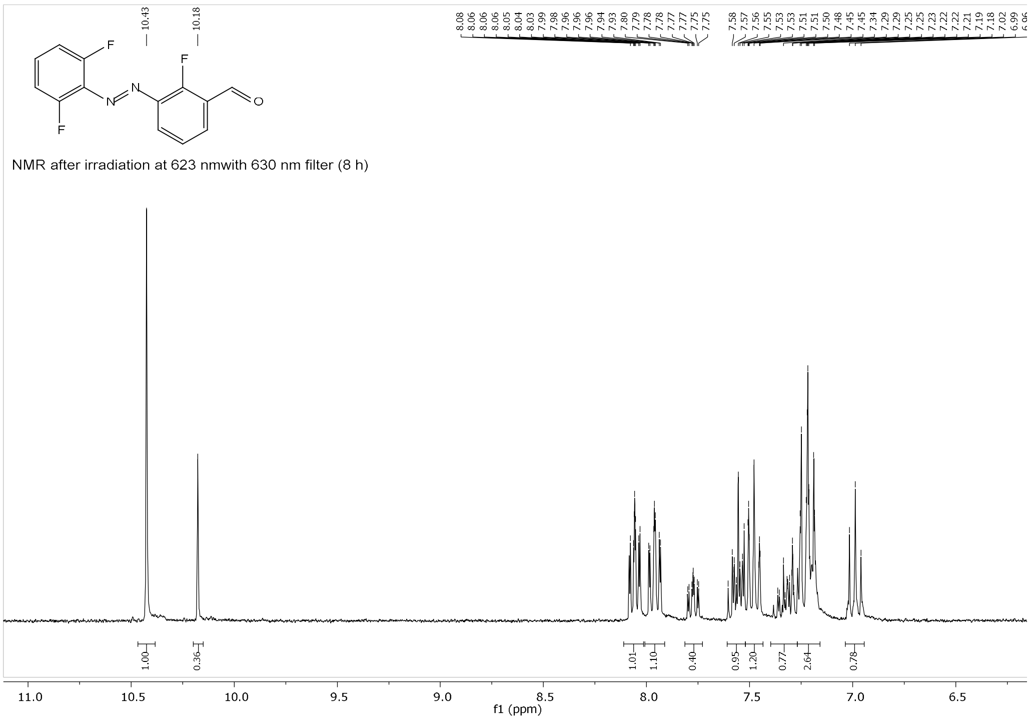

Figure S 153

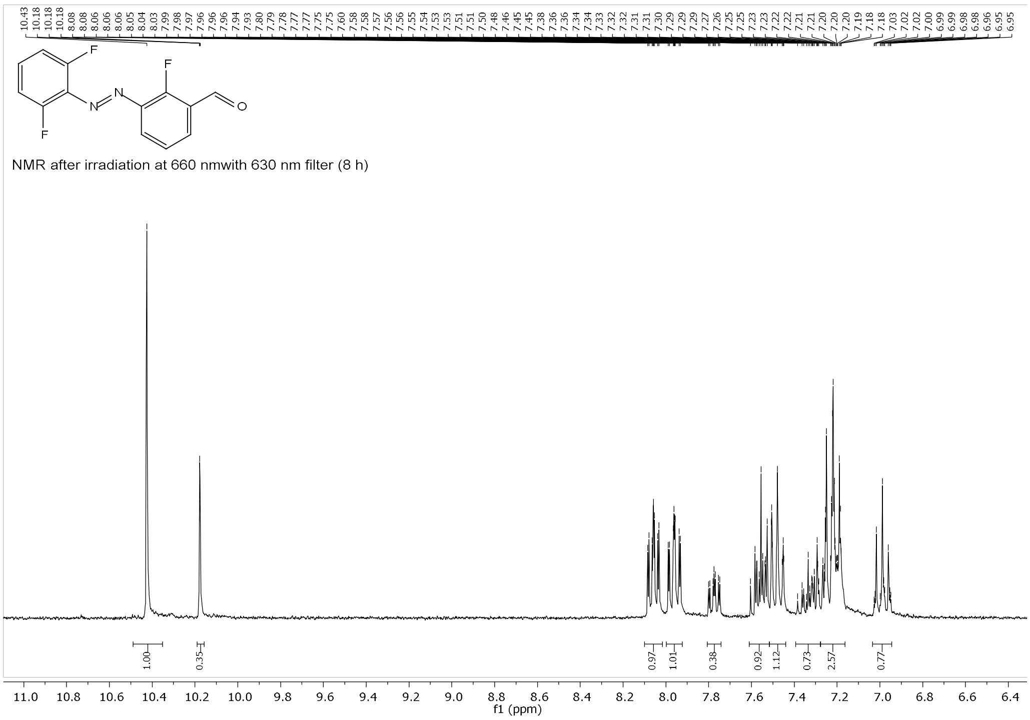

Figure S 154

The following ^{1}H NMR spectra were recorded in *DMSO* on a BRUKER 300 (300 MHz).

*NMR spectra of **8** after irradiation*

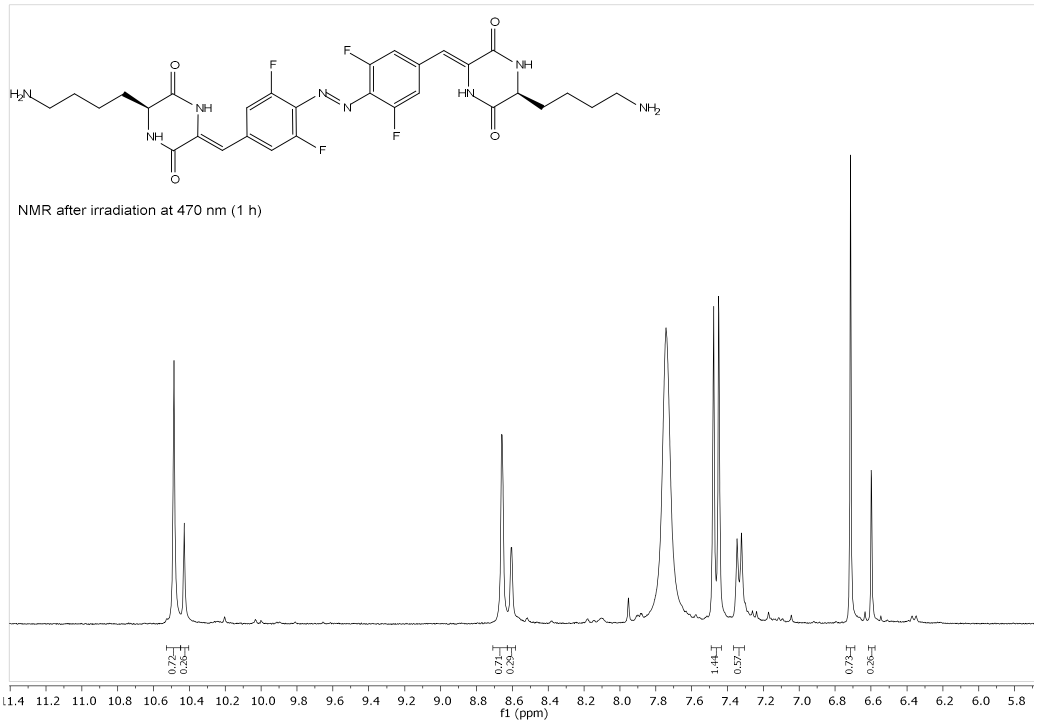

Figure S 155

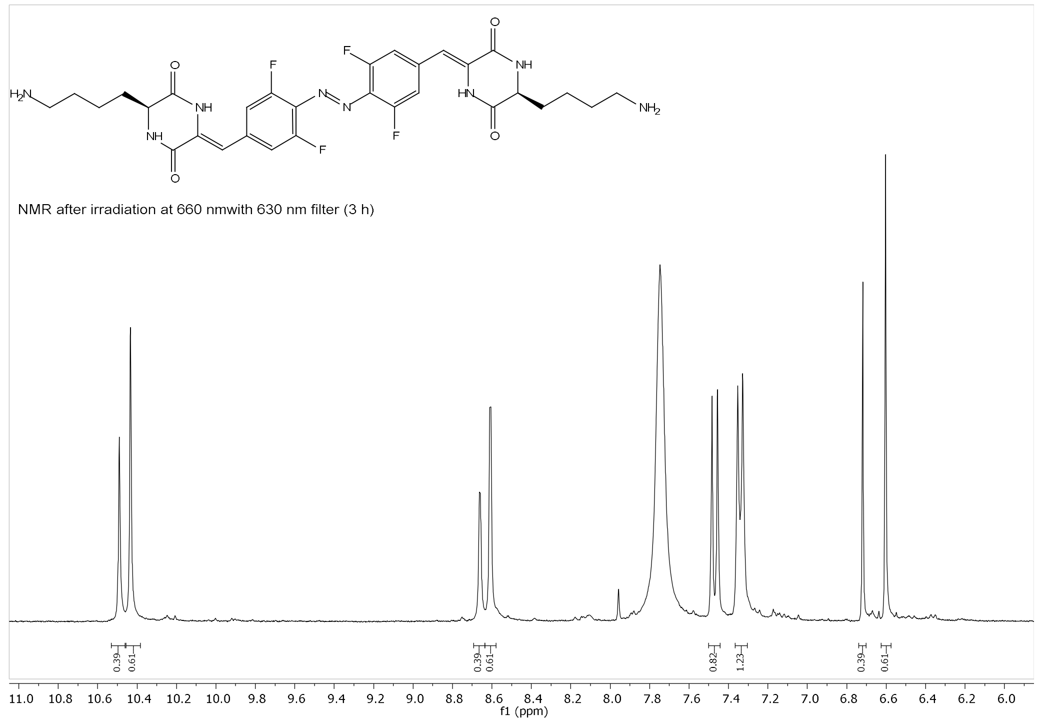

Figure S 156

13. Relative spectral power distribution of the used LED light sources

Relative spectral power distributions of the light sources used in the experiments described above have been quoted after the producer data (LED Engin, subsidiary of OSRAM).

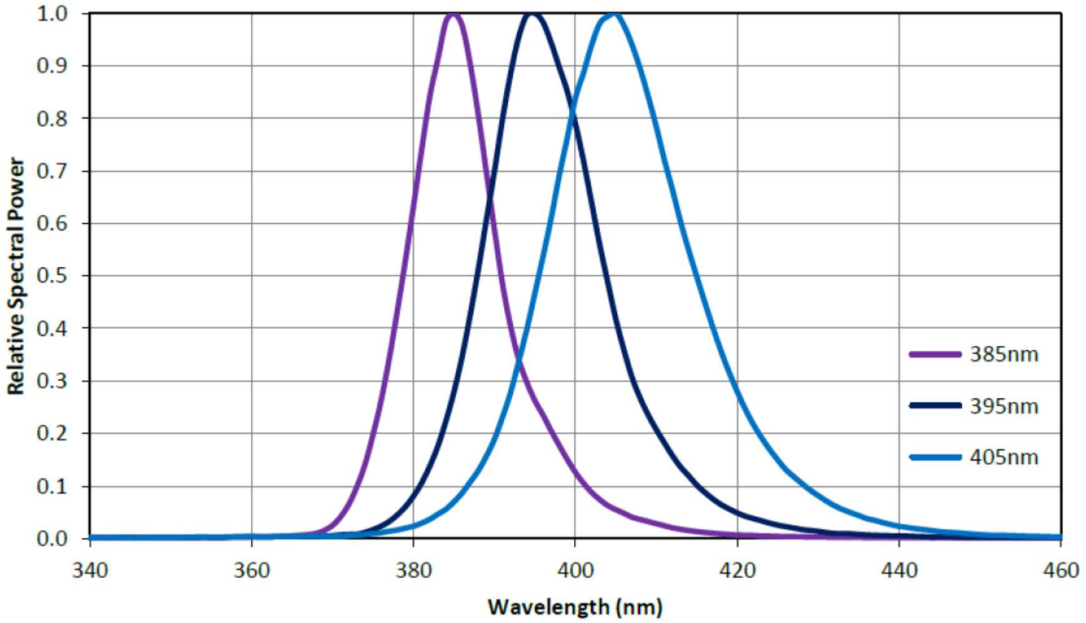

*Figure S 157. Spectral power distribution: **407 nm** (LuxiGenTM 405-410nm VIOLET LED Emitter LZ4-00UB0R, LED Engin/OSRAM)*

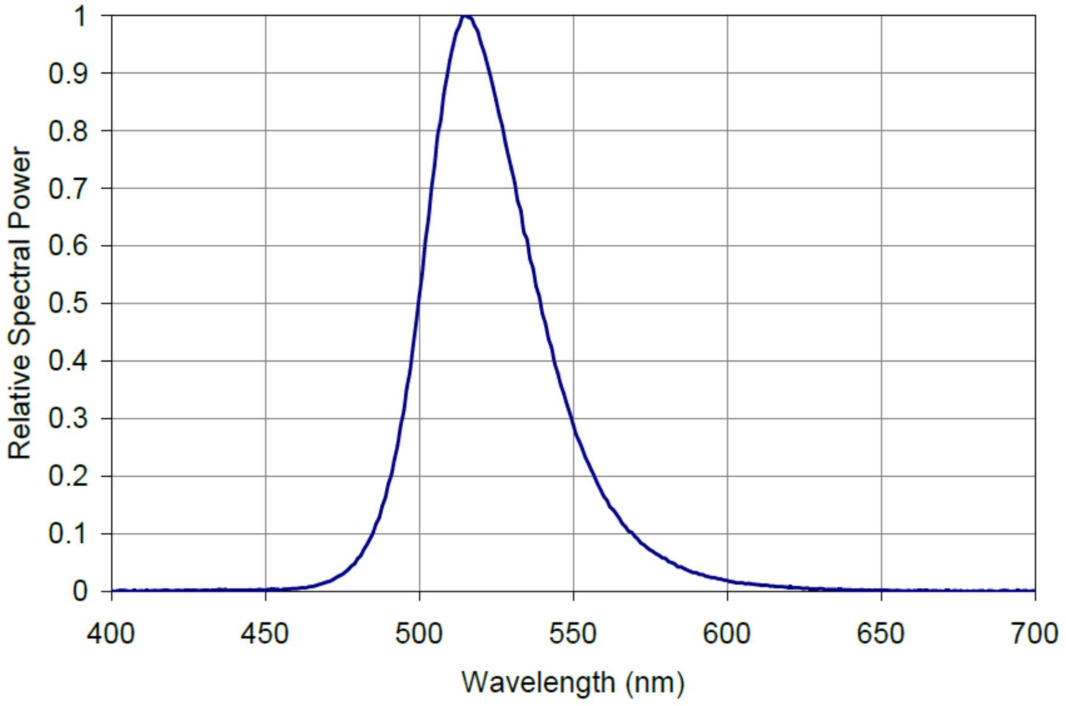

*Figure S 158. Spectral power distribution: **523 nm** (Green LED Emitter LZ4-00G108, LED Engin/OSRAM)*

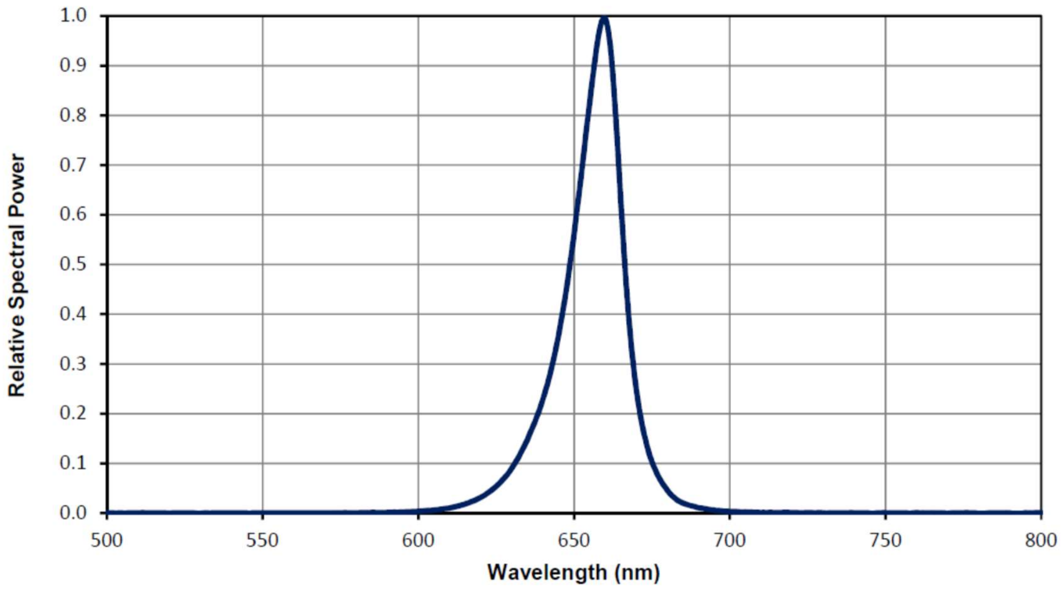

Figure S 159. Spectral power distribution: ***660 nm*** *(High Efficiency Deep Red 660nm LED Emitter LZ1-00R202 LED Engin/OSRAM).*

14. References

1. Gottlieb, H. E.; Kotlyar, V.; Nudelman, A., NMR Chemical Shifts of Common Laboratory Solvents as Trace Impurities. *The Journal of Organic Chemistry* **1997,** *62* (21), 7512-7515.

2. Bleger, D.; Schwarz, J.; Brouwer, A. M.; Hecht, S., o-Fluoroazobenzenes as readily synthesized photoswitches offering nearly quantitative two-way isomerization with visible light. *J Am Chem Soc* **2012,** *134* (51), 20597-600.

3. Knie, C.; Utecht, M.; Zhao, F.; Kulla, H.; Kovalenko, S.; Brouwer, A. M.; Saalfrank, P.; Hecht, S.; Bleger, D., ortho-Fluoroazobenzenes: visible light switches with very long-Lived Z isomers. *Chemistry* **2014,** *20* (50), 16492-501.

4. Gorska, K.; Manicardi, A.; Barluenga, S.; Winssinger, N., DNA-templated release of functional molecules with an azide-reduction-triggered immolative linker. *Chem Commun (Camb)* **2011,** *47* (15), 4364-6.

5. Cigl, M.; Bubnov, A.; Kaspar, M.; Hampl, F.; Hamplova, V.; Pacherova, O.; Svoboda, J., Photosensitive chiral self-assembling materials: significant effects of small lateral substituents. *J Mater Chem C* **2016,** *4* (23), 5326-5333.

6. Sabbatini, P.; Wellendorph, P.; Høg, S.; Pedersen, M. H. F.; Bräuner-Osborne, H.; Martiny, L.; Frølund, B.; Clausen, R. P., Design, Synthesis, and in Vitro Pharmacology of New Radiolabeled γ-Hydroxybutyric Acid Analogues Including Photolabile Analogues with Irreversible

Binding to the High-Affinity γ-Hydroxybutyric Acid Binding Sites. *Journal of Medicinal Chemistry* **2010,** *53* (17), 6506-6510.

7. Antoine John, A.; Lin, Q., Synthesis of Azobenzenes Using N-Chlorosuccinimide and 1,8-Diazabicyclo[5.4.0]undec-7-ene (DBU). *J Org Chem* **2017,** *82* (18), 9873-9876.

8. Bateman, S. A.; Kelly, D. P.; White, J. M.; Martin, R. F., DNA Binding Compounds. VII. Synthesis, Characterization and DNA Binding Capacity of 1,2-Dicarba-closo-dodecaborane Bibenzimidazoles Related to the DNA Minor Groove Binder Hoechst 33258. *Australian Journal of Chemistry* **1999,** *52* (4).

9. Schulte, M. L. Tetra-ortho-fluoro-azobenzenes for application in smart materials and catalysis. Bachelor, The Karlsruhe Institute of Technology, Karlsruhe, 2018.

10. Knie, C.; Utecht, M.; Zhao, F.; Kulla, H.; Kovalenko, S.; Brouwer, A. M.; Saalfrank, P.; Hecht, S.; Bléger, D., ortho-Fluoroazobenzenes: Visible Light Switches with Very Long-Lived Z Isomers. *Chemistry – A European Journal* **2014,** *20* (50), 16492-16501.

11. Frisch, M. J.; Trucks, G. W.; Schlegel, H. B.; Scuseria, G. E.; Robb, M. A.; Cheeseman, J. R.; Scalmani, G.; Barone, V.; Petersson, G. A.; Nakatsuji, H.; Li, X.; Caricato, M.; Marenich, A. V.; Bloino, J.; Janesko, B. G.; Gomperts, R.; Mennucci, B.; Hratchian, H. P.; Ortiz, J. V.; Izmaylov, A. F.; Sonnenberg, J. L.; Williams; Ding, F.; Lipparini, F.; Egidi, F.; Goings, J.; Peng, B.; Petrone, A.; Henderson, T.; Ranasinghe, D.; Zakrzewski, V. G.; Gao, J.; Rega, N.; Zheng, G.; Liang, W.; Hada, M.; Ehara, M.; Toyota, K.; Fukuda, R.; Hasegawa, J.; Ishida, M.; Nakajima, T.; Honda, Y.; Kitao, O.; Nakai, H.; Vreven, T.; Throssell, K.; Montgomery Jr., J. A.; Peralta, J. E.; Ogliaro, F.; Bearpark, M. J.; Heyd, J. J.; Brothers, E. N.; Kudin, K. N.; Staroverov, V. N.; Keith, T. A.; Kobayashi, R.; Normand, J.; Raghavachari, K.; Rendell, A. P.; Burant, J. C.; Iyengar, S. S.; Tomasi, J.; Cossi, M.; Millam, J. M.; Klene, M.; Adamo, C.; Cammi, R.; Ochterski, J. W.; Martin, R. L.; Morokuma, K.; Farkas, O.; Foresman, J. B.; Fox, D. J. *Gaussian 16 Rev. C.01*, Wallingford, CT, 2016.

12. Becke, A. D., Density-functional thermochemistry. III. The role of exact exchange. *The Journal of Chemical Physics* **1993,** *98* (7), 5648-5652.

13. McLean, A. D.; Chandler, G. S., Contracted Gaussian basis sets for molecular calculations. I. Second row atoms, Z=11–18. *The Journal of Chemical Physics* **1980,** *72* (10), 5639-5648.

14. Krishnan, R.; Binkley, J. S.; Seeger, R.; Pople, J. A., Self-consistent molecular orbital methods. XX. A basis set for correlated wave functions. *The Journal of Chemical Physics* **1980,** *72* (1), 650-654.

15. Miertuš, S.; Scrocco, E.; Tomasi, J., Electrostatic interaction of a solute with a continuum. A direct utilizaion of AB initio molecular potentials for the prevision of solvent effects. *Chemical Physics* **1981,** *55* (1), 117-129.

16. Casida, M. E.; Salahub, D. R., Asymptotic correction approach to improving approximate exchange–correlation potentials: Time-dependent density-functional theory calculations of molecular excitation spectra. *The Journal of Chemical Physics* **2000,** *113* (20), 8918-8935.

17. Karcher, J. W. Synthesis of novel photoswitchable small molecules for photomodulation of biopolymers. Master, The Karlsruhe Institute of Technology, Karlsruhe, 2015.

18. Bantle, T. Solid–phase synthesis of antisense agents based on PNA oligonucleotide analogues for in vivo photomodulation of zebrafish embryos' development. Master, The Karlsruhe Institute of Technology, Karlsruhe, 2017.

19. Kirchner, S. Photochromic 2,5-diketopiperazines and peptides for photomodulation of biological systems with visible light. Master, Heidelberg University, Heidelberg, 2018.

20. Y. Yamazaki, K. Tanaka, B. Nicholson, G. Deyanat-Yazdi, B. Potts, T. Yoshida, A. Oda, T. Kitagawa, S. Orikasa, Y. Kiso, H. Yasui, M. Akamatsu, T. Chinen, T. Usui, Y. Shinozaki, F. Yakushiji, B. R. Miller, S. Neuteboom, M. Palladino, K. Kanoh, G. K. Lloyd, Y. Hayashi, *Journal of Medicinal Chemistry* **2012**, *55*, 1056-1071.

21 David B. Konrad, Gökcen Savasci, Lars Allmendinger, Dirk Trauner, Christian Ochsenfeld, and Ahmed M. Ali Computational Design and Synthesis of a Deeply Red-Shifted and Bistable Azobenzene *Journal of the American Chemical Society* **2020**, *142*, *14*, 6538–6547.

22 Nadja A. Simeth, Alfredo Bellisario, Stefano Crespi, Maurizio Fagnoni, and Burkhard König Substituent Effects on 3-Arylazoindole Photoswitches *Journal of Organic Chemistry* **2019**, *84*, *11*, 6565–6575

23 Grimme, S.; Antony, J.; Ehrlich, S.; Krieg, H., A consistent and accurate ab initio parametrization of density functional dispersion correction (DFT-D) for the 94 elements H-Pu. *J. Chem. Phys.* **2010**, *132*, 154104.

24 Perdew, J. P.; Burke, K.; Ernzerhof, M., Generalized Gradient Approximation Made Simple. *Phys. Rev. Lett.* **1996**, *77*, 3865-3868.

25 Weigend, F., Accurate Coulomb-fitting basis sets for H to Rn. *Phys. Chem. Chem. Phys.* **2006**, *8*, 1057-1065.

26 Weigend, F.; Häser, M.; Patzelt, H.; Ahlrichs, R., RI-MP2: optimized auxiliary basis sets and demonstration of efficiency. *Chem. Phys. Lett.* **1998**, *294*, 143-152.

27 A. Maus, C. Hertlein, K. Saalwächter, A Robust Proton NMR Method to Investigate Hard/Soft Ratios, Crystallinity, and Component Mobility in Polymers. *Macromol. Chem. Phys.* **2006**, *207*, 1150.

28 S. Meiboom, D. Gill, Modified Spin-Echo Method for Measuring Nuclear Relaxation Times. *Rev. Sci. Instrum.* **1958**, *29*, 688.

29 T. Gullion, D. B. Baker, M. S. Conradi, New, compensated Carr-Purcell sequences. *J. Magn. Reson.* **1990**, *89*, 479.
